# Permeabilisation of the Outer Membrane of *Escherichia coli* for Enhanced Transport of Complex Molecules

**DOI:** 10.1111/1751-7915.70122

**Published:** 2025-03-09

**Authors:** Ivan Casas‐Rodrigo, Tobias Vornholt, Kathrin Castiglione, Tania Michelle Roberts, Markus Jeschek, Thomas R. Ward, Sven Panke

**Affiliations:** ^1^ Department of Biosystems Science and Engineering ETH Zurich Basel Switzerland; ^2^ Department of Chemistry University of Basel Basel Switzerland; ^3^ Institute of Bioprocess Engineering Friedrich‐Alexander‐Universität Erlangen‐Nürnberg Erlangen Germany; ^4^ Institute of Microbiology University of Regensburg Regensburg Germany; ^5^ National Centre of Competence in Research (NCCR) Molecular Systems Engineering Basel Switzerland

**Keywords:** cell envelope, OMP, outer membrane, permeability, pore

## Abstract

The bacterial envelope plays a critical role in maintaining essential cellular functions by selectively regulating import and export. The selectivity of this envelope can restrict the utilisation of externally provided compounds, thereby restricting the functional space of cellular engineering. This study systematically investigates the potential of large pore outer membrane proteins (OMPs) to enhance outer membrane permeability for diverse challenging compounds. We focus on the general porin OmpF, which facilitates the diffusion of water and small molecules, and specific OMP transporters FhuA and FepA, which mediate the translocation of small hydrophilic compounds. Through comprehensive characterisation, we evaluate the effects of recombinant expression of OMPs and engineered variants for small and hydrophilic compounds, aromatic molecules and bulky molecules and apply our findings to address two critical contemporary transport challenges: the uptake of large metal‐containing cofactors for artificial metalloenzymes and non‐permeant fluorescent Halo‐ligands for in vivo protein labelling. Notably, we demonstrate significant improvements in ArM‐catalysis and labelling. This study provides a practical guide for designing experiments that include outer‐membrane‐transport‐limiting steps. This study highlights the potential of engineered OMPs to overcome the limitations imposed by the cell envelope, enabling the incorporation of complex molecules and expanding the frontiers of cellular engineering.

## Graphical abstract

Access to synthetic compounds in the bacterial cytoplasm is hindered by the selective cell envelope, particularly in Gram‐negative bacteria like *Escherichia coli* . This study systematically compares three outer membrane proteins (OMPs) and their engineered variants, highlighting the potential of OMP engineering for the permeabilisation of the outer membrane.

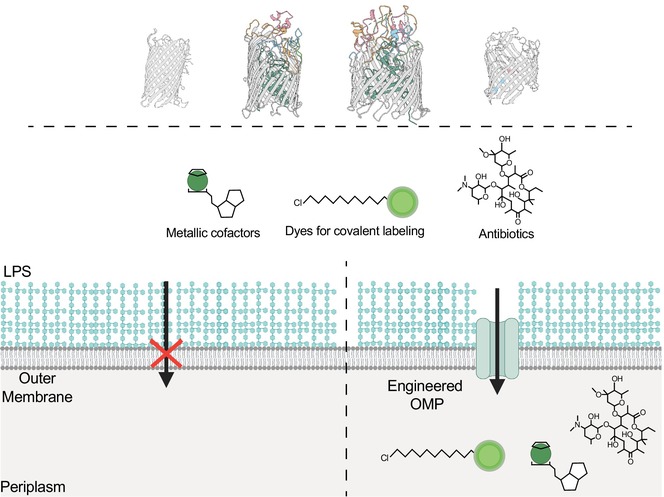

## 1 Introduction

The introduction of synthetic biology marked a shift in research focus from understanding natural organisms and their molecular processes to designing novel organisms with features not found in nature (Elowitz and Lim 2010) while applying engineering principles. This has expanded the boundaries of what biological systems can be engineered to do or even what they are made of (Cameron et al. 2014; Kobayashi et al. 2004; Martin et al. 2003; Dunkelmann et al. 2024; Marlière et al. 2011). Often, the expansion of function requires access to non‐canonical molecules in the cell, e.g., when implementing new genetic codes (Richardson et al. 2017; Fredens et al. 2019), expanding the use of existing or engineered pathways (Katsuyama et al. 2007), or integrating novel chemical strategies into cells via novel cofactors (Jeschek, Reuter, et al. 2016). However, this access is often difficult to ensure.

The cellular envelope controls such access of extracellular compounds to the interior of cells. In Gram‐negative bacteria, the selectivity resides in an external outer membrane and an inner or cytoplasmic membrane, which demarcates the periplasmic space between them (Silhavy et al. 2010). The outer membrane is a phospholipid bilayer with embedded lipopolysaccharides, lipoproteins and outer membrane proteins (OMPs) (Sun et al. 2022). It serves as a physical and mechanical barrier that selectively controls the transport of specific molecules into the cell, thereby enabling the metabolic processes of the cell, maintaining homeostasis (Shrivastava et al. 2017; Vergalli et al. 2020) and supporting essential biological functions such as cell envelope structure, self‐recognition, signalling and virulence (Aoki et al. 1979, 2008) and protection (Nikaido and Vaara 1985).

Numerous strategies have been proposed to enhance the permeability of the outer membrane through physical or chemical treatments of bacteria, which render the membrane temporarily permeable to a wide array of molecules. The different permeabilisation strategies included the addition of organic solvents, elevation of salt concentrations, rapid temperature fluctuations or incubation with specific buffers or biologically active compounds such as rhamnolipids or peptides (Irvin et al. 1981; Hancock 2003; Vaara 1992; Skerlavaj et al. 1990; Ibrahim et al. 2001; Sotirova et al. 2008; Chen 2007). As a readout, permeabilisation markers were employed, such as aromatic p‐nitrophenyl phosphate or N‐phenyl‐1‐naphtylamine (NPN) and larger antibiotic molecules like erythromycin.

Additionally, genetic interventions have been explored to increase outer membrane permeability. Notably, modifications to the lipopolysaccharide composition of the outer membrane showed increased permeability for molecules such as the cephalosporin nitrocefin and modified tetrapeptides (Ni and Chen 2004). Also, the intracellular production of viral proteins led to an enhanced uptake of small molecules (Birkeland 1994; Patel et al. 2014). Still, all these strategies have serious limitations due to the harsh conditions required, which can compromise cell viability, and only a limited range of molecules can permeate the membrane, typically favouring specific types of compounds (Chen 2007).

An alternative approach for permeabilising the outer membrane consists of the engineering of OMPs. OMPs are integral membrane proteins composed of β‐barrel channels of 8 to 24 β‐strands (Vergalli et al. 2020) and play, among other functions, a crucial role in facilitating nutrient transport across the cell membrane through a central channel (Nikaido and Vaara 1985). Within the group of OMP transporters, two subgroups can be distinguished based on the specificity of their transport functions. The first subgroup consists of general porins, typically trimeric β‐barrels with relatively little specificity as to which molecules they let pass by passive diffusion (Schirmer 1998). A good example is the well‐studied OmpF, which allows the diffusion of molecules of up to 600 Da (Cowan et al. 1995).

The second subgroup comprises specific OMP transporters, responsible for the translocation of specific hydrophilic molecules such as sugars, vitamins or nucleosides into the cell (Aoki et al. 1979). These specific OMPs typically adopt a monomeric β‐barrel structure, and they possess a specific binding site, obstructing access to the channel, with an affinity for the target compound (Nikaido and Vaara 1985). Notably, they have a larger pore diameter than general OMPs, rendering them potentially promising candidates for permeabilising the outer membrane to larger, bulkier compounds. Furthermore, the absence of a quaternary structure facilitates their engineering (Odokonyero et al. 2014).

OMPs have been a prominent target for engineering the permeability of the outer membrane, mostly around the general OMP OmpF and reduced‐selectivity variants of the specific OMP FhuA (Phale et al. 2001; Jeanteur et al. 1994; Lout et al. 1996; Saint et al. 1996; Ruff et al. 2016; Bertelmann et al. 2022; Cohen‐Khait et al. 2021; Cowan et al. 1992; Kobayashi and Nakae 1985). However, it remains difficult to develop a rational strategy on how to improve transport for different molecules, as the available data set on different specific OMPs is small, and a systematic comparison of various OMPs and different molecules is lacking. In this study, we systematically compared the permeabilisation capabilities of three different OMPs and their respective mutated variants across a set of compounds with diverse characteristics. Our objective was not to enhance the uptake of a specific compound but rather to improve the overall permeability of the outer membrane to a broad range of molecules. We further demonstrated the potential of this increased permeability in facilitating periplasmic‐based applications that are currently limited by the inherent impermeability of the outer membrane.

## 2 Experimental Procedures

### 2.1 Chemicals and Reagents

Unless stated otherwise, chemicals were obtained from Sigma‐Aldrich (Sigma‐Aldrich, Burlington, MA, USA). Primers were synthesised by Sigma‐Aldrich, and enzymes for molecular cloning purposes were obtained from New England Biolabs (New England Biolabs, Ipswich, MA, USA).

### 2.2 Common Equipment

For DNA amplification through PCR and cloning of plasmids through Gibson assembly reactions, a peqSTAR 96x thermocycler (PEQLAB, Erlangen, Germany). The resulting reaction mixture was used to transform *E. coli* DH5α cells by heat‐shock using a Bioshake IQ thermoshaker (QINSTRUMENTS, Jena, Germany). Cell cultures were incubated at the indicated temperature and shaking conditions in an ISF1‐X shaking incubator (Kuehner, Birsfelden, Germany). The biomass of these cultures, when needed, was determined by measuring their optical density at 600 nm (OD_600_) with an Eppendorf Biophotometer D30 (Eppendorf, Hamburg, Germany). An Eppendorf Centrifuge 5810R was used for centrifugation steps.

### 2.3 Strains

All the strains used in this study, along with their genotypes, can be found in Table S10.

Deletion of the malE gene to develop the BL21 (DE3) ∆malE strain was performed via λ red recombineering (Datsenko and Wanner 2000). First, the kanamycin resistance expression cassette surrounded by FRT sites from plasmid pKD4 was amplified using primers IC22JUN05 and IC22JUN06 (Table S11), which contain 50 bp of the target gene flanking regions as overhangs. The PCR product was purified by gel electrophoresis in 1% agarose, cutting the band and subsequently performing a gel extraction purification using the Zymoclean Gel DNA Recovery Kit (Zymo Research, Freiburg im Breisgau, Germany).

BL21(DE3) electrocompetent cells were transformed with plasmid pKD46 and spread on an LB + agar plate supplemented with carbenicillin (100 mg·L^−1^). After overnight incubation at 30°C, a 5 mL LB culture supplemented with carbenicillin (100 mg·L^−1^) was inoculated from a single colony on an agar plate and incubated overnight again at 30°C, 200 rpm. The following day, the culture was diluted 1:100 in new LB + carb media and incubated at 30°C, 200 rpm, until OD_600_ reached 0.5–0.6. Then, L‐arabinose was added to a final concentration of 15 mM and incubated at 30°C, 200 rpm for 2 h. Cells were subsequently put on ice and centrifuged three times (3220 rcf, 10 min, 4°C), resuspending them in 5 mL of sterile ice‐cold water for the first two centrifugations and in 50 μL for the last one. The purified PCR product from the kanamycin resistance expression cassette amplification was added to the 50 μL of electrocompetent cells and subsequently electroporated. Cells were recovered for 1 h at 37°C and plated on an LB + agar plate supplemented with kanamycin (50 mg·L^−1^). After overnight incubation at 37°C, several colonies were streaked on new LB + Kan plates and were again incubated at 37°C overnight. A colony from each of the resulting streaks was used to carry out colony PCRs to confirm the deletion of malE using IC22JUN03 and IC22JUN04 primers.

After confirmation of the malE deletion, the kanamycin resistance expression cassette was removed following a similar procedure. Cells were transformed with the plasmid pCP20 encoding the FLP recombinase and plated on LB supplemented with carbenicillin (100 mg·L^−1^) at 30°C. After overnight incubation at 30°C, several colonies were used for colony PCR to confirm the deletion of the kanamycin resistance expression cassette using IC22JUN03 and IC22JUN04 primers. Simultaneously, these colonies were streaked by duplicate on LB + agar plates and on LB + agar plates supplemented with carbenicillin (100 mg·L^−1^) and were incubated at 37°C overnight until no growth could be observed in the presence of carbenicillin, which indicates the loss of the pCP20 plasmid.

### 2.4 Plasmid Construction

All plasmids used in this study were constructed using the pSEVA architecture (Martínez‐García et al. 2014) and are listed in Table S12. The genes encoding the OMPs were amplified through PCR using the primers listed in Table S11, using as a template already existing plasmids or synthetic double‐stranded DNA sequences in the case of FepA ∆C128/∆L, FepA ∆C/∆4L and cloned into the previously linearised vector pSEVA291 with kanamycin antibiotic resistance and pBR322‐ROP origin of replication (Martínez‐García et al. 2014). Except for the ArMs‐assay (see below), OMPs are under the control of a T7 promoter and harbour a T7 terminator, and these sequences were added as overhangs for the corresponding forward and reverse primers, respectively.

For the ArMs‐based whole‐cell biocatalysis assay, the vector used was pSEVA361 with chloramphenicol antibiotic resistance and p15A origin of replication (Martínez‐García et al. 2014). Gene expression for the OMPs was under the control of the rhamnose inducible promoter P_*rhaBAD*_ and terminated by a T1 terminator, already present in the pSEVA vector used for the assembly of the coding sequence (CDS) of the OMPs. Thus, the final PCR product was formed by the corresponding CDS flanked by regions homologous to the P_*rhaBAD*_ promoter in 5′ and to the T1 terminator in 3′. To produce the periplasmic Sav, a pSEVA291 vector with kanamycin antibiotic resistance and a pBR322‐ROP origin of replication was used (Martínez‐García et al. 2014). The Sav gene was under the control of the T7 promoter and the T7 terminator. The DNA sequence of the plasmids and the protein sequence of the OMPs used in this study can be found in Data S1 and S2.

PCR products were purified by gel electrophoresis in 1% agarose, cutting the band and subsequently performing a gel extraction purification using the Zymoclean Gel DNA Recovery Kit (Zymo Research, Freiburg im Breisgau, Germany). An aliquot of 100 ng of linearised pSEVA291 was incubated with a 2‐fold molar excess of the corresponding PCR fragment, 15 μL of an in‐house Gibson assembly mixture (Gibson et al. 2009) and incubated at 50°C for 1 h. The resulting reaction mixture was used to transform *E. coli* DH5α by heat‐shock. Transformed cells were recovered in 1 mL of Luria‐Bertani (LB) medium at 37°C for 1 h and plated on LB + agar medium supplemented with kanamycin (50 mg·L^−1^). Plasmids were isolated from the resulting colonies using the ZR Plasmid Miniprep—Classic kit (Zymo Research, Freiburg im Breisgau, Germany) and Sanger sequenced (Microsynth, Balgach, Switzerland).

### 2.5 Cell Cultivation

*Escherichia* *coli* strains carrying a plasmid for protein overproduction were pre‐cultivated in 5 mL of LB medium supplemented with kanamycin (50 mg·L^−1^) in 15 mL culture tubes at 37°C, 200 rpm, overnight. When the cultivated strains carried a second plasmid (to overproduce MBP:HaloTag), the media were supplemented with 1% (w/v) glucose to prevent the premature production of OMP. The saturated pre‐cultures were diluted 1:100 in new fresh LB medium supplemented with kanamycin (50 mg·L^−1^) and isopropyl‐β‐D‐thiogalactopyranoside (IPTG, 50 μM) and incubated again in 96‐deep well plates (1 mL) until their OD_600_ reached 0.4–0.6. The resulting samples were subsequently processed as indicated to assess cell permeability to small hydrophilic compounds, NPN or erythromycin, to quantify the levels of cellular MBP:HaloTag and to assess cell permeability to HaloTag dyes.

### 2.6 Assessing Outer Membrane Permeability to Small Hydrophilic Compounds

BL21 ∆ABCF (Meuskens et al. 2017) cell cultures were prepared as described above and diluted to a final OD_600_ of 0.05 into 800 μL of fresh LB medium supplemented with kanamycin (50 mg·L^−1^) and IPTG (50 μM). These diluted cultures were transferred into a FlowerPlate (MTP‐48‐B) (Beckman Coulter, Baesweiler, Germany) and incubated inside a BioLector (Beckman Coulter, Baesweiler, Germany) at 37°C and 800 rpm. Biomass was measured every 10 min for 20 h using an excitation wavelength of 620 nm. The logarithmic growth curves of the biomass were analysed using an in‐house Python linear regression script generated to determine the slope of the curve that corresponds to the maximum specific growth rate (*μ*_max_). This script is publicly available on GitLab (https://git.bsse.ethz.ch/BPL_Panke/max‐specific‐growth‐rate‐calculator). Manual adjustments were made to the non‐overproducing control strain (−), as well as to the strains overproducing GFP, OmpF and OmpF ∆ (Figure S2) due to their complex growth behaviour, which did not follow the typical sigmoid growth curve.

### 2.7 Assessing Outer Membrane Permeability to NPN

To test the effect of OMP expression on outer membrane permeability to NPN, 1 mL cultures of BL21(DE3) cells expressing OMPs were grown as described in the protocol, were pelleted (3220 rcf, 10 min) and resuspended in 1 mL of phosphate buffer saline (PBS). The optical density of the cultures was measured and subsequently adjusted to an OD_600_ of 0.2 by diluting them in PBS. Afterwards, 200 μL of each culture was incubated with 10 μM N‐phenyl‐1‐naphthylamine (NPN) (from a 1 mM stock prepared in ethanol) or with ethanol alone for 30 min at 20°C, 300 rpm. After staining, cell suspensions were kept on ice and washed three times by centrifugation at 3220 rcf at 4°C for 10 min, followed by resuspension in 200 μL of fresh PBS buffer. The resulting samples were analysed on a BD LSR Fortessa SORP (BD Biosciences, Franklin Lakes, NJ, USA) using a 355 nm laser and a 450/50 nm filter for analysis of the emitted fluorescence signal.

### 2.8 Assessing Outer Membrane Permeability to Erythromycin

A modified 2‐fold broth dilution method was used to determine the susceptibility of *E. coli* expressing the different OMPs to erythromycin. Cultures of a volume of 1 mL of BL21(DE3) cells expressing OMPs were grown as described in the cultivation protocol. The optical density of the cultures was measured. Cultures were diluted to an OD_600_ of 0.005 into fresh LB medium supplemented with kanamycin (50 mg·L^−1^), IPTG (50 μM) and increasing concentrations of erythromycin in a 2‐fold series (from 1 to 1000 mg·L^−1^), also adding samples with no erythromycin as a control. Cultures of 1 mL were incubated in a 96‐deep well plate at 37°C, 300 rpm, for 18 h. Afterwards, endpoint measurements of their OD_600_ were performed using a Tecan Infinite M1000 (Tecan, Maennedorf, Switzerland) after transferring 200 μL from the incubated 96‐deep well plate into a standard 96‐deep well plate. The MIC was the lowest concentration at which the OD_600_ was smaller than 0.05 determined as the mean from independent triplicates that were not allowed to be more than one dilution apart.

### 2.9 Assessing Outer Membrane Permeability to Metal Cofactors for Biocatalysis With ArMs

To test the effect of OMP expression on whole‐cell biocatalysis with ArMs, the pores were co‐produced with a previously engineered streptavidin variant (Sav S112M K121R) in *E. coli* BL21‐Gold(DE3). Streptavidin was expressed using the T7 promoter and targeted to the periplasm using an N‐terminal *ompA* secretion signal (Jeschek, Panke, et al. 2016). The OMPs tested were expressed using the rhamnose promoter. To conduct the assay, 96‐deepwell plates were filled with 500 μL of LB supplemented with kanamycin (50 mg·L^−1^) and chloramphenicol (34 mg·L^−1^) per well. Cultures were inoculated from single colonies on agar plates and grown overnight at 37°C and 300 rpm. Aliquots of 20 μL of culture were used to inoculate expression cultures in 1 mL LB with kanamycin and chloramphenicol. These cultures were grown at 37°C and 300 rpm for 1.5 h. At this point, the plates were placed at room temperature for 20 min, and subsequently, streptavidin and OMP expression were induced by the addition of 50 μM IPTG and 0.2% (w/v) L‐rhamnose. Expression was carried out at 20°C and 300 rpm for 20 h. Subsequently, the OD_600_ of the cultures was determined in a Tecan Infinite M1000 (Tecan, Maenedorf, Switzerland) using 50 μL samples diluted with an equal volume of PBS. Afterwards, the plates were centrifuged (3220 rcf, 15°C, 10 min). The supernatant was discarded, and the pellets were resuspended in 400 μL of incubation buffer containing the biotinylated ruthenium cyclopentadienyl (Biot‐HQ)CpRu cofactor (1 μM (Biot‐HQ)CpRu) in 50 mM 2‐(N‐morpholino)ethanesulfonic acid (MES), 0.9% NaCl (pH 6.1). Cells were incubated with the cofactor for 1 h at 15°C and 300 rpm. Afterwards, plates were centrifuged (2000 rcf, 15°C, 10 min), the supernatant was discarded, and the pellets were resuspended in 500 μL of incubation buffer lacking the cofactor to remove unbound cofactor. Following another centrifugation step, cell pellets were resuspended in 200 μL reaction buffer (100 μM allyl carbamate coumarin in 50 mM MES, 0.9% NaCl, pH 6.1). Reactions were performed at 37°C and 300 rpm for 20 h. The product concentration was quantified by measuring the fluorescence intensity with a Tecan Infinite M1000 at an excitation of 394 nm and an emission of 460 nm, and the values were normalised by dividing by the OD_600_ of the respective culture.

### 2.10 Quantification of MBP‐HaloTag Production by Western Blot

To quantify the MBP‐HaloTag production levels, 1 mL cultures of BL21(DE3) ∆*malE* cells producing OMPs were grown as described in the cultivation protocol, adding chloramphenicol (34 mg·L^−1^) and 0.2% (w/v) L‐rhamnose to induce MBP‐HaloTag overproduction. The optical density of the cultures was measured. Then biomass was adjusted by transferring a volume equivalent to 200 μL divided by OD_600_ of each sample into new tubes, and cells were pelleted (3220 rcf, 10 min). Pellets were frozen at −20°C and thawed before resuspending them in 40 μL of Tris‐MOPS‐SDS Running Buffer (Genscript Biotech, Piscataway, NJ, USA) and 10 μL of 5× Laemmli buffer. Resuspended samples were incubated at 95°C for 10 min, and SDS‐PAGE electrophoresis was carried out using an ExpressPlus PAGE Gel (Genscript Biotech, Piscataway, NJ, USA). After electrophoresis, proteins were transferred onto an Amersham Protran nitrocellulose membrane at 250 mA for 45 min and then stained with 0.2% (w/v) Ponceau S solution for a reversible detection of protein bands. After blotting, the nitrocellulose membrane was blocked with a blocking buffer (5% (w/v) non‐fat dry milk in 1× PBS with 0.15% Tween 20) for 1 h. After removing the blocking solution, the membrane was incubated with a primary anti‐MBP polyclonal antibody (1:500) (Thermo Fisher Scientific, Waltham, MA, USA) at 4°C overnight. The membrane was washed three times with PBS for 5 min and then incubated with the VRDye 490 Donkey anti‐Rabbit IgG secondary antibody (1:10,000) (LI‐COR Biosciences, Lincoln, NE, USA) for 1 h at room temperature. This secondary antibody is conjugated to the VRDye 490 fluorophore, which allows for the detection and quantification of the fluorescent signal using LI‐COR imaging systems. The membrane was washed four times with PBS for 5 min, and fluorescent signal was analysed using Odyssey CLx Imaging System (LI‐COR Biosciences, Lincoln, NE, USA). Band intensities were analysed using Image Studio Software (LI‐COR Biosciences, Lincoln, NE, USA).

### 2.11 Assessing Outer Membrane Permeability to HaloTag Dyes by Flow Cytometry

To test the effect of OMP expression on outer membrane permeability to HaloTag dyes, 1 mL cultures of BL21(DE3) ∆*malE* cells producing OMPs were grown using a modified version of the cultivation protocol previously described. In this case, the saturated pre‐cultures were diluted 1:100 in new fresh LB medium supplemented with kanamycin (50 mg·L^−1^), chloramphenicol (34 mg·L^−1^) and IPTG (50 μM final concentration) to induce the production of OMP, and 0.2% (w/v) L‐rhamnose to induce the production of MBP:HaloTag. Cultures were subsequently incubated again in 96‐deep well plates (1 mL) until their OD_600_ reached 0.4–0.6. Cells were pelleted (3220 rcf, 10 min) and resuspended in 1 mL of phosphate buffer saline (PBS). The optical density of the cultures was measured and subsequently adjusted to an OD_600_ of 0.2 by diluting them in PBS. Afterwards, 200 μL of each culture was incubated with 1 μM of HaloTag TMR, HaloTag JF646 or HaloTag AF488 (Promega, Madison, WI, USA) (from a 1 mM stock prepared in DMSO) or with DMSO alone for 30 min at 20°C, 300 rpm. After staining, cell suspensions were always kept on ice and washed three times by centrifugation at 3220 rcf at 4°C for 10 min, followed by resuspension in 100 μL of fresh PBS buffer. The resulting samples were analysed on a BD LSR Fortessa SORP (BD Biosciences, Franklin Lakes, NJ, USA) by using a 561 nm laser and a 686/15 nm filter, a 640 nm laser and a 670/14 nm filter and a 488 nm laser and a 530/30 nm filter for the analysis of the emitted fluorescence signal by HaloTag TMR, HaloTag JF646 and HaloTag AF488, respectively.

### 2.12 Assessing Outer Membrane Permeability to HaloTag Dyes by Confocal Microscopy

To test the effect of OMP expression on outer membrane permeability to HaloTag dyes, 1 mL cultures of BL21(DE3) ∆*malE* cells producing OMPs were grown using a modified version of the cultivation protocol previously described. In this case, the saturated pre‐cultures were diluted 1:100 in new fresh LB medium supplemented with kanamycin (50 mg·L^−1^), chloramphenicol (34 mg·L^−1^) and IPTG (50 μM) to induce the production of OMP, and 0.2% (w/v) L‐rhamnose to induce the production of MBP:HaloTag. Cultures were subsequently incubated again in 96‐deep well plates (1 mL) until their OD_600_ reached 0.4–0.6.

Cells were pelleted (3220 rcf, 10 min) and resuspended in 1 mL of phosphate buffer saline (PBS). The optical density of the cultures was measured and subsequently adjusted to an OD_600_ of 0.2 by diluting them in PBS. Afterwards, 200 μL of each culture was incubated with 1 μM of HaloTag AF488 (Promega, Madison, WI, USA) (from a 1 mM stock prepared in DMSO) and 10 μg mL^−1^ of 4′,6‐diamidino‐2‐phenylindole (DAPI) for 30 min at 20°C, 300. After staining, cell suspensions were always kept on ice and washed three times by centrifugation at 3220 rcf at 4°C for 10 min, followed by resuspension in 100 μL of fresh PBS buffer. Samples were analysed on a Leica SP8 Falcon (Leica Microsystems, Wetzlar, Germany) with a 40x objective, using a 405 and 488 nm lasers to excite DAPI and HaloTag‐AF488, respectively. Acquired images were analysed using Fiji software. Calculated integrated densities for both DAPI and HaloTag‐AF488 signals were used for quantification purposes.

### 2.13 Data Analysis and Figure Design

**FIGURE 1:**
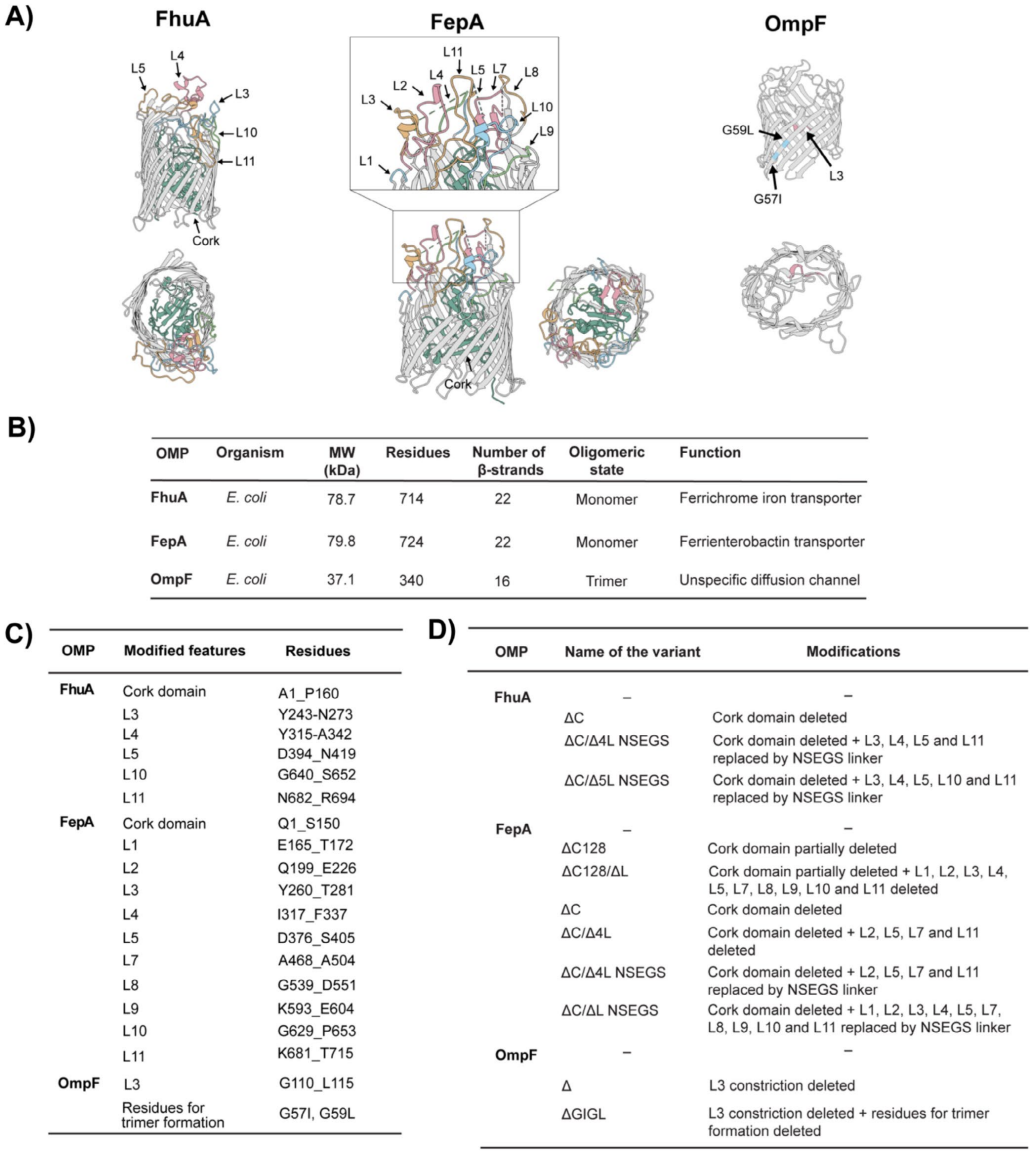
Outer membrane proteins were investigated in this study. (A) Ribbon diagrams (side and top view) of the structure of the OMPs used in this study. The domains that were modified to obtain the corresponding variants are annotated and highlighted in colour. The deleted cork domains are highlighted in green and the deleted loops are highlighted in different colours. Diagrams were generated using the structural data available in Protein Data Bank OmpF (PDB:2ZFG) (Yamashita et al. 2008), FhuA (PDB:1BY3) (Locher et al. 1998) and FepA (PDB:1FEP) (Buchanan et al. 1999). (B) Table indicating the OMPs assessed in this study and their major structural features. (C) Table summarising the structural features of the wildtype OMPs that were subsequently modified. (D) Summary of modified structural features.

The diagrams depicted in Figure 1 were generated using UCSF Chimera (Pettersen et al. 2004) and the structural data available in the Protein Data Bank for OmpF (PDB:2ZFG) (Yamashita et al. 2008), FhuA (PDB:1BY3) (Locher et al. 1998) and FepA (PDB:1FEP) (Buchanan et al. 1999).

Diameters of the channels of the OMPs, minimal diameters of NPN, erythromycin, (Biot‐HQ)CpRu metallic cofactor, HaloTag‐JF656, HaloTAg‐TMR and HaloTag‐AF488 were estimated with PyMOL (Schrödinger, New York, NY, USA).

Flow cytometry data corresponding to NPN and HaloTag dyes permeabilisation assays was analysed using FlowJo software (FlowJo, Ashland, OR, USA). All the plots shown as well as the statistical analysis carried out in this study were performed using Prism 9 (GraphPad Software, Boston, MA, USA).

BioRender (BioRender, Toronto, Canada) was used for Figures 3, 4, 5, 6.

## 3 Results

### 3.1 Construction of a Set of OMP Variants

An extended set of OMP candidates (Figure 1 and Table S1) was selected and engineered based on previously reported data on their structure and permeabilisation potential. First, we included the specific OMP FhuA (714 amino acid residues, 78.7 kDa [Coulton et al. 1986]), a β‐barrel protein with 22 β‐sheets and a globular ‘cork’ domain that blocks its channel. This cork domain is responsible for the specificity of FhuA, allowing only the import of iron in complex with ferrichrome (740 Da) upon interaction with this domain. Besides the wild‐type protein, we included the corkless variant FhuA ∆C (554 amino acid residues, 61.8 kDa) that harbours the deletion ∆A1‐P160, corresponding to the cork domain in the N‐terminus that would otherwise block the channel and interact with ferric hydroxamate to facilitate its uptake (Ruff et al. 2016; Braun et al. 1999). This deletion transforms FhuA into a passive diffusion channel that was successfully used to increase outer membrane permeabilisation to aromatics, terpenes (Ruff et al. 2016) and steroid compounds (Bertelmann et al. 2022). We also included the variant FhuA ∆C/∆4L NSEGS (465 amino acid residues, 51.6 kDa), which has not only the cork domain removed but also features shortened extracellular loops L3, 4, 5 and 11, obtained by substituting the original sequence stretches with an NSEGS pentapeptide linker connecting adjacent β‐strands (Mohammad et al. 2011). The shortening of these extracellular loops, which are predicted to occlude the inside of the FhuA channel once the cork domain is removed, might help to increase the free space inside the lumen of the channel (Mohammad et al. 2011). This FhuA ∆C/∆4L NSEGS variant was found before to sensitise *E. coli* cells to several bulky antibiotics, such as erythromycin (Krishnamoorthy et al. 2016). Similarly, Wolfe et al. (2016) developed a further engineered FhuA variant, referred to in this study as FhuA ∆C/∆5 L NSEGS (455 amino acid residues, 50.6 kDa), in which the extracellular loop 10 was also replaced by an NSEGS linker. Although there is no data available on the in vivo permeabilisation capabilities of FhuA ∆C/∆5 L NSEGS, its structural similarity to FhuA ∆C/∆4L NSEGS and its reported improved conductance (Wolfe et al. 2016) suggest that FhuA ∆C/∆5 L NSEGS could be a promising candidate variant to permeabilise the OM.

As a second specific OMP, we included FepA (724 amino acid residues, 79.8 kDa), which is structurally and functionally similar to FhuA. It is also a monomeric β‐barrel composed of 22 β‐strands responsible for the uptake of iron conjugated with enterobactin (719 Da). FepA has been shown to facilitate the uptake of large molecules, including antibiotics conjugated to enterobactin (Zheng et al. 2012), bacteriocin colicin B with 55 kDa (Devanathan and Postle 2007), as well as the uptake of DNA conjugated to colicin B (Cohen‐Khait et al. 2021). While engineered variants of FepA lacking the N‐terminal cork domain have been described (Vakharia and Postle 2002; Scott et al. 2001), to the best of our knowledge, no in vivo data on membrane permeabilisation has been reported in the literature. Hence, we designed a number of variants in analogy to those designed for FhuA. First, we took a conservative approach for a variant in which the cork domain was partially deleted (∆Q1_P128), to try to unblock the pore channel while avoiding the misfolding of the extracellular loops (Mohammad et al. 2011). The resulting variant is referred to as FepA ∆C128 (596 amino acid residues, 65.8 kDa). This variant was further engineered by shortening 10 of the 11 extracellular loops (leaving L6 untouched, which natively contains only 3 amino acid residues), retaining two non‐β‐strand loop residues per terminus to connect the adjacent β‐strands and generating short extracellular loops, each consisting of only four residues, as described previously for FhuA (Fioroni et al. 2014). This variant is referred to as FepA ∆C128/∆L (407 amino acid residues, 45.2 kDa).

In a second approach, we removed the entire cork domain (∆Q1_S150) to obtain a fully corkless FepA variant (Vakharia and Postle 2002; Scott et al. 2001) referred to here as FepA ∆C (574 amino acid residues, 63.7 kDa). FepA ∆C was further modified by shortening the four largest extracellular loops (L2, L5, L7, and L11), leading to FepA ∆C/∆4L (460 amino acid residues, 52.1 kDa), or all extracellular loops except for L6, leading to FepA ∆C/∆L. The plasmid encoding the equivalent FepA ∆C/∆L construct proved to be genetically unstable in vivo by rapidly attracting multiple mutations, so we ultimately discarded this variant. Next, the shortened loops were replaced by NSEGS linkers as before (Mohammad et al. 2011), leading to FepA ∆C/∆4L NSEGS (464 amino acid residues, 52.1 kDa) and FepA ∆C/∆L NSEGS (394 amino acid residues, 44.2 kDa).

Besides these specific OMPs, we also included a family of variants of the general OMP OmpF (340 amino acid residues, 37.1 kDa), specifically the previously reported variant OmpF ∆ (334 amino acid residues, 36.4 kDa), in which a region of the loop L3, usually forming an α‐helix inside the constriction region, was deleted (∆G110_L115) (Karasawa et al. 2022), and OmpF ∆ with two substitutions, G57I and G59L, resulting in OmpF ∆ GI/GL. These residues are critical for protein–protein interaction during trimer formation, and their substitution renders OmpF more likely to exist in monomeric form (Naveed et al. 2012). The genes encoding all the aforementioned OMP variants were recombinantly expressed using plasmid‐based expression systems under the control of the indicated inducible systems. Consequently, all *E. coli* strains used in this study retained their endogenous OMP repertoire alongside the engineered OMPs unless otherwise indicated.

### 3.2 Expression of OMP Variants and Effect on Outer Membrane Permeability to Small Hydrophilic Molecules

Some, but not all, of the variants employed in our study were expressed in *E. coli* before. Also, the specific setup of an expression system can have implications for functional expression. To ensure the functionality of the low selectivity OMP variants and the parent OMPs after recombinant expression in *E. coli* , we expressed the corresponding genes in pSEVA291 plasmids (Kan/pBR322‐ROP) (Martínez‐García et al. 2014) from the T7 promoter and tested for improved growth after expression in strain BL21 ∆ABCF (Karasawa et al. 2022). This strain, whose genetic background is BL21 Gold (DE3), lacks the genes encoding four of the most common unspecific OMPs (OmpA, OmpC, OmpF and LamB), and it is known to display a reduced specific growth rate because of that (Meuskens et al. 2017). We reasoned that upon functional expression, the increased availability of functional unspecific pores in the outer membrane would improve growth due to an enhanced influx of mostly hydrophilic nutrients (Naveed et al. 2012). To compare the performance of the different OMP variants, time‐course growth experiments were carried out to determine the maximum specific growth rate (*μ*_max_) of the different strains overproducing each of the variants. Negative effects resulting from the additional burden of recombinant overproduction could potentially obscure the benefits in nutrient uptake conferred by these OMPs and, as such, we also included a BL21 ∆ABCF strain overproducing GFP in the cytoplasm as a control (Figures S1 and S2).

**FIGURE 2:**
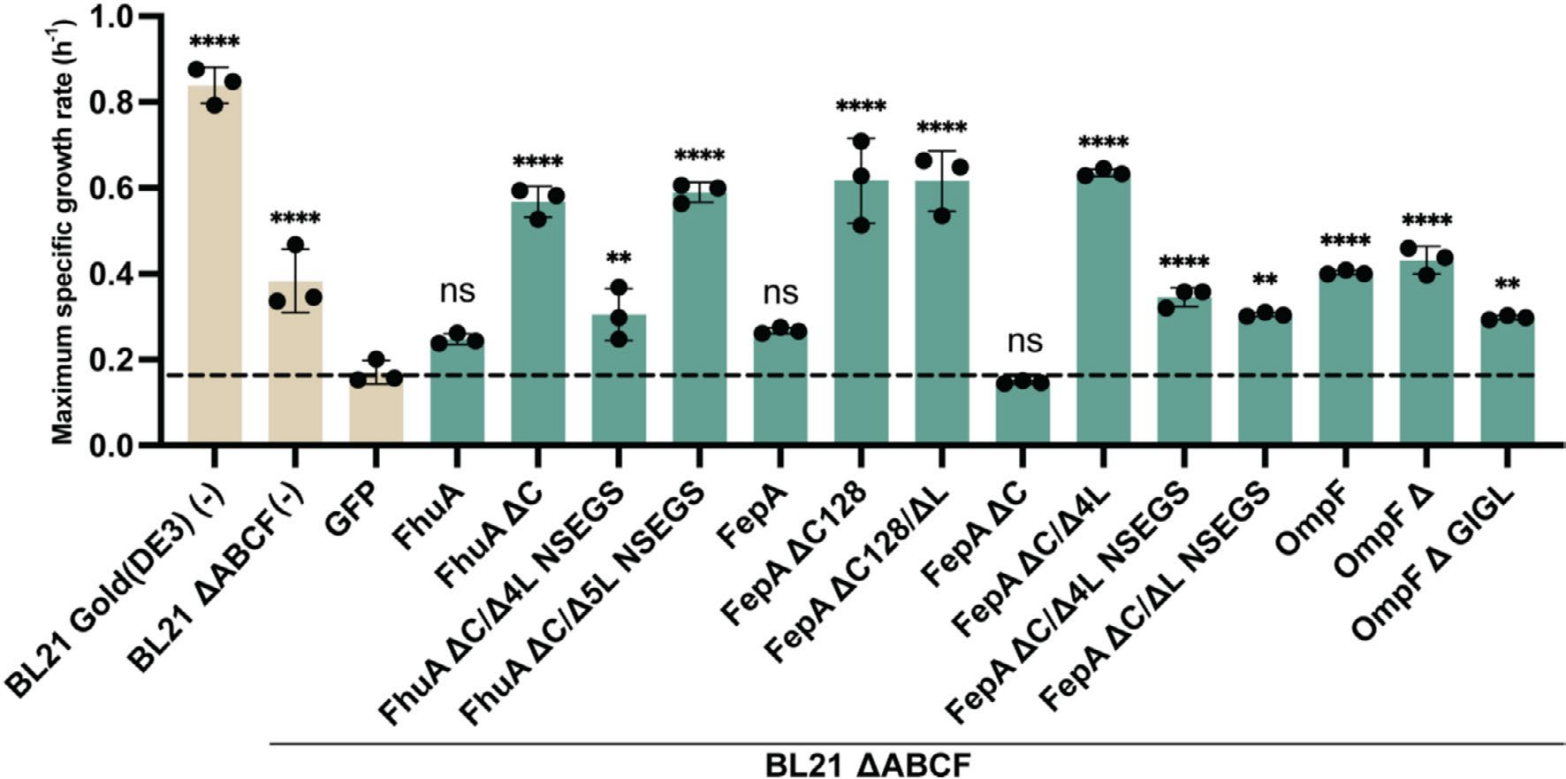
OMP variant production in BL21 ∆ABCF and its effects on outer membrane permeability to small hydrophilic molecules. Maximum specific growth rates of *Escherichia coli* BL21 Gold not overexpressing a protein (for comparison, first entry on the left) and BL21 ∆ABCF either expressing no recombinant protein (second entry) or a variety of control proteins (GFP) or OMPs and variants. Data show the average and standard deviation of three biological replicates grown in an LB medium at 37°C. The original growth curves and the numerical data can be found in Figures S1 and S2. An ANOVA with a significance level *α* = 0.05 was carried out to determine statistically significant differences in comparison to the GFP population. *P* values > 0.05 are indicated with ‘ns’, *p* values ≤ 0.01 are indicated with ‘**’ and *p* values ≤ 0.0001 are indicated with ‘****’.

As expected, BL21 ∆ABCF exhibited approximately a 2‐fold decrease in its *μ*_max_ compared to its parent BL21 Gold (DE3) (Figure 2) when grown on an LB medium. Also as expected, overexpression of GFP reduced *μ*_max_ further. Notably, the overproduction of all analysed OMP variants except for FepA ∆C leads to a significant increase in *μ*_max_, suggesting that these expressed OMP variants are functional, including the new variants as explained above, and indeed facilitate access to nutrients from the medium to the periplasm.

The absence of an effect of FepA ∆C expression on the specific growth rate might be an indication of non‐functional expression, but we consider this very unlikely, most importantly because in later experiments this variant also showed clear effects in terms of facilitating transport across the outer membrane (see below). Also, the protein has been functionally produced in *E. coli* before (Scott et al. 2001). Interestingly, the expression of FepA variants without the cork domain and with shortened loops showed an effect on maximum specific growth rate. This could imply that the natural extracellular loops of FepA, when folded into the channel, are a significant factor in hindering the passage of small hydrophilic molecules to the *E. coli* periplasm. Also, a 3.6‐fold increase in *μ*_max_ is observed for both FepA ∆C128 and FepA ∆C128/∆L variants, suggesting the partial removal of the cork domain already allows the passive diffusion of small hydrophilic compounds through the channel while preventing the extracellular loops from occluding it, as it might occur when the cork domain is completely removed (Mohammad et al. 2011).

Looking at the effect that the expression of the unmodified OMPs has on the specific growth rate, the results for FhuA, FepA (no observable effect due to the remaining selectivity) and OmpF (increase of specific growth rate because of improved permeability of the outer membrane) are consistent with expectation.

### 3.3 Effect of OMP Variants Overproduction on the Permeability of the Outer Membrane to Aromatic Hydrophobic Molecules

**FIGURE 3:**
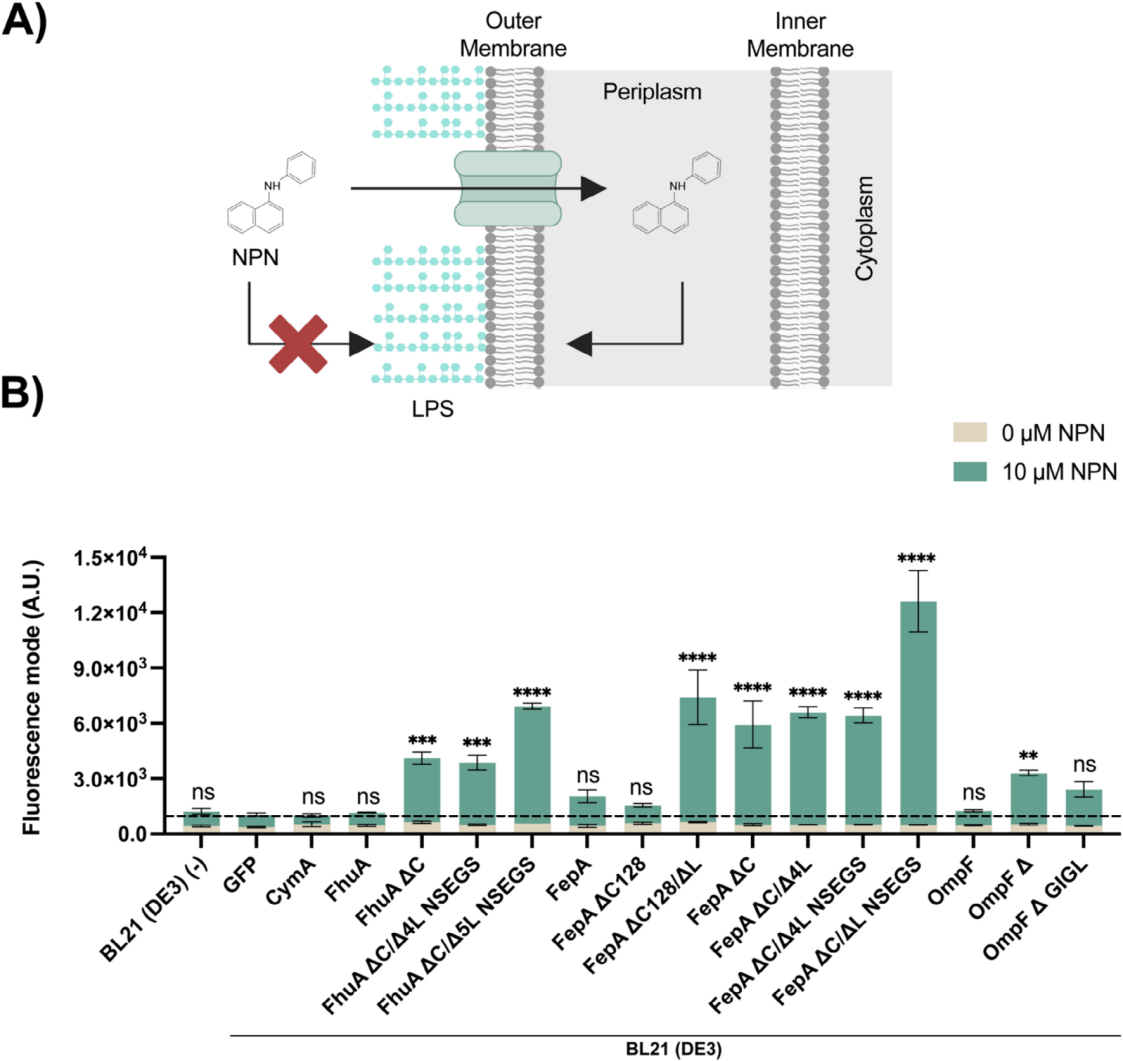
Effect of OMP expression on *Escherichia coli* outer membrane permeability to aromatic hydrophobic molecules. (A) Schematic representation of the principle of the NPN import assay. For details, see text. (B) Flow cytometry analysis of OM permeability to NPN. Fluorescence mode of OMP overproducing strains and controls after incubation without or with 10 μM NPN. First entry: Data for BL21(DE3) containing a plasmid without a gene for overexpression. All data to the right refer to overexpression of the mentioned protein in strain BL21 (DE3). Data shown corresponds to the average and standard deviation of three biological replicates. An ANOVA with a significance level *α* = 0.05 test was carried out to determine statistically significant differences in comparison to a strain that recombinantly produces GFP instead of an OMP. *P* values > 0.05 are indicated with 'ns', *p* values ≤ 0.01 are indicated with '**', *p* values ≤ 0.001 are indicated with '***' and *p* values ≤ 0.0001 are indicated with '****'. The fluorescence histograms and detailed fluorescence data corresponding to the geometric mean, median and mode can be found in Figure S3 and Table S2.

We next investigated the permeabilisation capabilities of the different OMP variants by testing their ability to enhance outer membrane permeability for NPN, which is an aromatic hydrophobic compound (219 Da) that exhibits fluorescence upon intercalation into a phospholipid layer. Since the extracellular side of *E. coli*'s outer membrane is packed with lipopolysaccharides (LPS), NPN can only interact with the phospholipids of the outer membrane if it reaches the periplasm and binds to the inner leaflet of the outer membrane, which makes NPN a standard probe for assessing the permeability of the outer membrane in Gram‐negative bacteria (Helander and Mattila‐Sandholm 2000). We used it as a proxy for access to aromatic compounds to the periplasm in response to the expression of the different OMP variants (Figure 3A).

We examined the ability of our set of OMPs and OMP variants to facilitate the passage of NPN through the outer membrane in BL21(DE3) cells. The overproduction of most of the engineered variants, with the exception of FepA ∆C128 and OmpF ∆GIGL, resulted in a significant increase in intracellular NPN fluorescence signal (Figure 3B). The strains overexpressing variants FhuA ∆C/∆5L NSEGS, FepA ∆C128/∆L, FepA ∆C, FepA ∆C/∆4L, FepA ∆C/∆4L NSEGS and FepA ∆C/∆4L NSEGS showed the highest signal increases, with improvements between 9‐ and 18.0‐fold. The improvements obtained from overproducing FhuA ∆C, FhuA ∆C/∆4L NSEGS and OmpF ∆ were also significant but smaller (between 4‐ and 5‐fold). Note that FepA ∆C also led to an improved NPN‐derived signal, supporting the hypothesis that the previous result obtained for permeabilisation to small hydrophilic molecules in strain BL21 ∆ABCF was not due to a lack of functional expression.

Overproduction of the parent OMPs does not lead to an NPN‐derived signal increase, which again is consistent with expectation. For the specific OMPs, their specificity remains intact, and for OmpF, the physicochemical properties of NPN might lead to its exclusion despite the fact that its molecular weight (219 Da) is far below the generally accepted size limit for OmpF. Notably, *E. coli* BL21, which contains the natural gene for OmpF in its chromosome, is impermeable to NPN, as the first entry in Figure 3B shows.

As before for small hydrophilic media components, we find again a correlation between the increase in NPN signal and whether a parent OMP has been engineered or not. In addition, there seems to be a stronger correlation between the NPN signal and the extent of engineering in the OMP variants. Specifically, OMPs with more extensive engineering, such as FhuA ∆C/∆5L NSEGS and FepA ∆C/∆L NSEGS, exhibited the highest fluorescence signal increases compared to the other variants of the same OMP. This suggests that these engineered OMPs are more effective in permeabilising the *E. coli* outer membrane for NPN, either because of different levels of functionally expressed proteins or because the mutations in the OMP variants actually enlarge the free space within their channels (without biasing the pore against the aromatic nature of NPN).

### 3.4 Examination of the OMPs Overproduction Effect on *E. coli* Permeability to Complex Molecules

**FIGURE 4:**
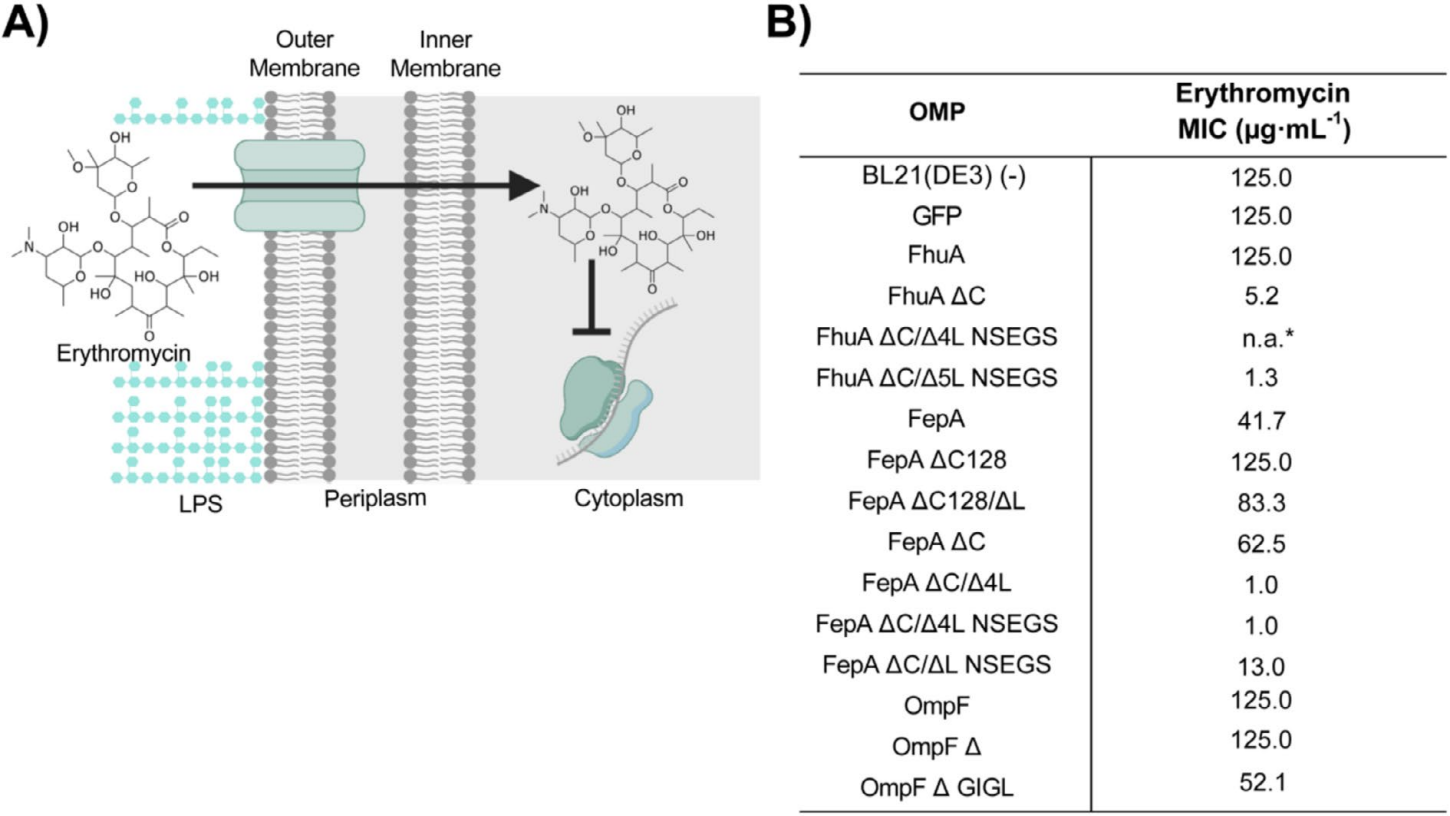
Effect of OMP production on outer membrane permeability to bulky hydrophobic molecules. (A) Schematic representation of the principles of the assay exploiting erythromycin‐based blocking of translation. (B) MICs of strains overproducing the indicated OMP and controls. (−) represents the MIC obtained for strain BL21(DE3) containing a plasmid without a gene for overexpression. Data show the average of three biological replicates grown in LB medium at 37°C. Detailed data is available in Figure S4 and Table S3. *n.a., not available (see text for details).

We next wanted to compare the effect of the different OMP variants on the permeabilisation of the outer membrane to a bulky hydrophobic compound, such as the antibiotic erythromycin (734 Da). As an assay, we tested whether the susceptibility of *E. coli* to this antimicrobial agent was increased as a possible result of outer membrane permeabilisation, and thus determined minimum inhibitory concentrations (MICs) as a read out (Krishnamoorthy et al. 2016). As a macrolide antibiotic, erythromycin inhibits bacterial protein synthesis when it is transported inside the cell. While it is capable of freely diffusing across the inner membrane, the outer membrane serves as the primary barrier to its entry into the cell (Capobianco and Goldman 1994). Consequently, the greater the permeability of the outer membrane is, the lower the concentration required to inhibit bacterial growth should result (Figure 4A) (Krishnamoorthy et al. 2016; Muheim et al. 2017).

In agreement with previous studies (Krishnamoorthy et al. 2016), the MIC of *E. coli* cells not overproducing any protein or overproducing GFP is between 125 and 62.5 μg mL^−1^ (Table S3). Again, the overproduction of most engineered OMP variants showed a clear effect consistent with enhanced outer membrane permeabilisation for erythromycin compared to the OMP parents (Figure 4B, Figure S4). Specifically, FhuA ∆C, FhuA ∆C/5 L NSEGS, FepA ∆C/∆4L, FepA ∆C/∆4L NSEGS, FepA ∆C/∆L NSEGS and OmpF ∆ GIGL exhibited lower MICs, showing fold reductions between 1.5 and 125, in comparison to the GFP‐overproducing control. These results are also consistent with an earlier study conducted for FhuA variants (Krishnamoorthy et al. 2016). It should be noted that strains overproducing FhuA ΔC/∆4L NSEGS could not be evaluated in this study due to consistent inactivation of the expression construct.

Unexpectedly, strains overproducing FepA showed a lower MIC to erythromycin than the engineered variants FepA ∆C128, FepA ∆C128/∆L and FepA ∆C, but not lower than the FepA variants with complete cork removal and shortened extracellular loops. This might suggest that already native FepA allows erythromycin to pass through the outer membrane.

Moreover, the increase in the MIC for FepA ∆C128/∆L and FepA ∆C compared to the FepA‐overproducing strain could be due to the conformational changes that the extracellular loops may undergo after the (partial) deletion of the cork domain. These conformational changes might block the pore channel, as suggested previously for FhuA (Mohammad et al. 2011). Consistent with this hypothesis, FepA ∆C128/∆L, with shortened loops, showed a lower MIC for erythromycin than FepA ∆C128, as dothe FepA variants with the cork domain completely removed, the strains overproducing variants with shortened extracellular loops (FepA ∆C/∆4L, FepA ∆C/∆4L NSEGS and FepA ∆C/∆L NSEGS) over strains producing variants with extracellular loops untouched (FepA ∆C).

In summary, the MIC data is consistent with improved permeability of the outer membrane for FhuA variants with removed cork‐domain and FepA variants with removed cork domain and shortened loops. Notably, the effect of the OmpF variant overexpression was only mild, suggesting that erythromycin might not pass the central channel effectively if only OmpF is expressed as a recombinant unspecific OMP. The increased performance of the engineered FhuA and FepA variants, as measured by enhanced sensitivity to a bulky antibiotic, is noteworthy.

After these three initial comparisons for outer membrane permeabilisation towards small hydrophilic, aromatic and bulky hydrophobic molecules, we summarised the obtained results in Table 1 and Table S4. The systematic comparison suggests four particularly promising OMP variants with respect to outer membrane permeabilisation, namely FhuA ∆C, FhuA ∆C/∆5L NSEGS, FepA ∆C/∆4L and FepA ∆C/∆L NSEGS. We continued with them to test the effect of their overexpression on two well‐established bioengineering applications that can benefit from outer membrane permeabilisation. Additionally, we retained the two mutated variants going back to the general OMP OmpF for comparison.

**TABLE 1:** Summary of the permeabilisation effects of OMP variant overproduction.

Overproduced protein	Growth of BL21 ∆ABCF	NPN import to periplasm in BL21 (DE3)	Reduction to erythromycin MIC in BL21 (DE3)
GFP	−	−	−
FhuA	−	−	−
**FhuA ∆C**	**+++**	**+**	**+++**
FhuA ∆C/∆4L NSEGS	+	−	−
**FhuA ∆C/∆5 L NSEGS**	**+++**	**++**	**+++**
FepA	−	−	++
FepA ∆C128	+++	−	−
FepA ∆C128/∆L	+++	++	+
FepA ∆C	−	+	++
**FepA ∆C/∆4L**	**+++**	**+**	**+++**
FepA ∆C/∆4L NSEGS	++	+	+++
**FepA ∆C/∆L NSEGS**	**+**	**+++**	**+++**
OmpF	++	−	−
**OmpF ∆**	**++**	**+**	−
**OmpF ∆ GIGL**	**+**	**+**	**++**

*Note:* Rating depends on the effect size of overproducing a specific OMP variant when compared to the GFP overproducing control in per cent. GFP overproducing strain controls were taken as baseline or 0% results, and the best result among all tested variants was taken as 100% improvement. Three ‘+’: Above or equal to 75%. Two ‘+’: Above or equal to 50%. One ‘+’: Above or equal to 25%. ‘−’: Below 25%. OMP variants in bold were advanced for further investigation.

### 3.5 Effect of OMP Variant Production on Whole‐Cell Biocatalysis

**FIGURE 5:**
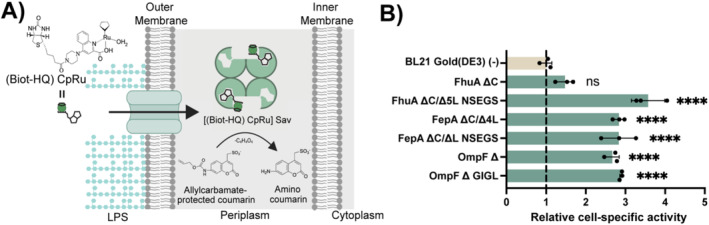
Effect of OMP production on ArMs‐based whole‐cell biocatalysis. (A) Schematic representation of the ArMs‐based whole‐cell biocatalysis reaction to deallylate the protected coumarin compound in the *Escherichia coli* periplasm. (B) Cell‐specific activity of the BL21 Gold (DE3) strains producing Sav S112M K121R and the indicated OMP variant, normalised to the activity of the strain producing the same Sav variant but no OMP variant (7.7E+04 A.U.), indicated as ‘(−)’. Data shown correspond to the average and the standard deviation of three biological replicates. Individual replicates are represented by circles. The dashed line represents the levels of activity shown by the control without OMP expression. An ANOVA with a significance level *α* = 0.05 test was carried out to determine statistically significant differences in comparison to the strain producing the same Sav variant but no OMP being overproduced. *P* values > 0.05 are indicated with 'ns' and *p* values ≤ 0.0001 are indicated with '****'. Detailed absolute and normalised cell‐specific activity values can also be found in Table S5.

We wanted to test whether the permeabilisation effects that we had observed in the three previous assays also extended to a measurable benefit for periplasm‐based applications. One such application is the introduction of novel enzyme cofactors into biochemistry to enable novel biocatalysis (Rosati and Roelfes 2010; Lewis 2013; Markel et al. 2019; Davis and Ward 2019). One promising route to achieve such an introduction is the periplasmic expression of a scaffold protein (e.g., streptavidin), which then receives a biotinylated external cofactor and assembles to artificial metalloenzymes (ArM) in the periplasm (Hassan et al. 2019; Nödling et al. 2018; Liang et al. 2019). Since the import of artificial cofactors into the cytoplasm of bacteria typically leads to only small activities (Reynolds et al. 2017; Huang et al. 2021), such periplasmic expression strategies are a valuable alternative strategy, for example, in directed evolution campaigns. However, they still suffer from the passage of the outer membrane as a bottleneck (Jeschek, Reuter, et al. 2016; Liang et al. 2019; Himiyama and Okamoto 2020; Vornholt et al. 2021). We used the de‐allylation of an allylcarbamate‐protected coumarin (357 Da) by a biotinylated ruthenium cyclopentadienyl (Biot‐HQ)CpRu‐streptavidin complex as a model reaction (Völker and Meggers 2017; Völker et al. 2014). The biotinylated cofactor has a size of 670 Da, and as the scaffold, we used an engineered variant of streptavidin (Sav S112M K121R) directed towards the periplasm (Figure 5A), which has been shown to increase the deallylation activity compared to its unmodified parent (Vornholt et al. 2021).

For this experiment, the expression setup was adapted. Specifically, the T7 promoter was used for optimal periplasmic streptavidin expression, while the production of OMP variants was controlled by the rhamnose inducible promoter P_*rhaBAD*_. After adaptation, these strains were then grown with the recombinant proteins expressed, incubated with the catalyst, washed to remove excess catalyst and then incubated with the substrate to determine activity. Product formation was determined by taking advantage of the fluorescence of the de‐allylated product of the ArM‐catalysed reaction.

Interestingly, all OMP‐overproducing strains except FhuA ∆C exhibited significantly higher cell‐specific activity for de‐allylation compared to the strain without OMP overproduction (Figure 5B), with a maximum effect for the overexpression of FhuA ∆C/∆5 L NSEGS, for which the strain showed a 3.6‐fold increase in comparison to a non‐overproducing strain. Co‐expression with streptavidin of FepA ∆C/∆4L, FepA ∆C/∆L NSEGS, but also OmpF ∆ and OmpF ∆GIGL, led to lesser, but still notable, increases in fluorescence (above 2.6‐fold) (Table S5). Clearly, the co‐expression of engineered OMPs with the ArM scaffold led to higher cell‐specific de‐allylation activity.

### 3.6 Effect of OMP Production on Covalent Labelling of Proteins

Another application that frequently suffers from limited transport across the cell envelope is fluorescent labelling of cell constituents, for example, specific proteins by covalent attachment of a fluorescent label to a tag on the protein of interest. One such archetypical labelling system is the HaloTag system, in which a modified dehalogenase (the tag) is fused to the target protein and facilitates the covalent attachment to itself of a fluorescent chloroalkane substrate (Los et al. 2008). However, typically the size of substrates for labelling is too large to reach the periplasm without problems. To investigate whether the co‐expression of an engineered OMP can improve periplasmic labelling, we selected the periplasmic maltose‐binding protein (MBP) as a model protein and extended it at its C‐terminus by a HaloTag. The MBP:HaloTag fusion protein was co‐produced with the indicated OMP variants in a BL21(DE3) ∆*malE* strain, from which the gene encoding wildtype MBP had been deleted. We first investigated, by Western blotting, the amount of Halo‐tagged MBP produced when co‐expressed with the different engineered OMPs (Figure S5) to ensure that a possible absence of an effect was not due to a lack of any MBP:HaloTag presence. Curiously, cells overproducing OmpF ∆ GIGL exhibited no detectable MBP:HaloTag protein, so we excluded this strain from further experiments. For all the remaining strains, a positive band corresponding to the MBP:HaloTag fusion protein could be observed. Then, we conducted labelling experiments with three different fluorescent HaloTag dyes (HaloTag‐TMR (636 Da), HaloTag‐JF646 (816 Da) and HaloTag‐AF488 (999 Da)). For this, the expression of OMPs and tagged MBP was induced for 3 h, cells were washed and incubated with different dyes, washed again to remove excess dye, and then analysed by flow cytometry. The results for each dye across the suite of strains co‐expressing the different engineered OMPs were then normalised for the corresponding MBP:HaloTag production levels previously determined by Western blotting.

**FIGURE 6:**
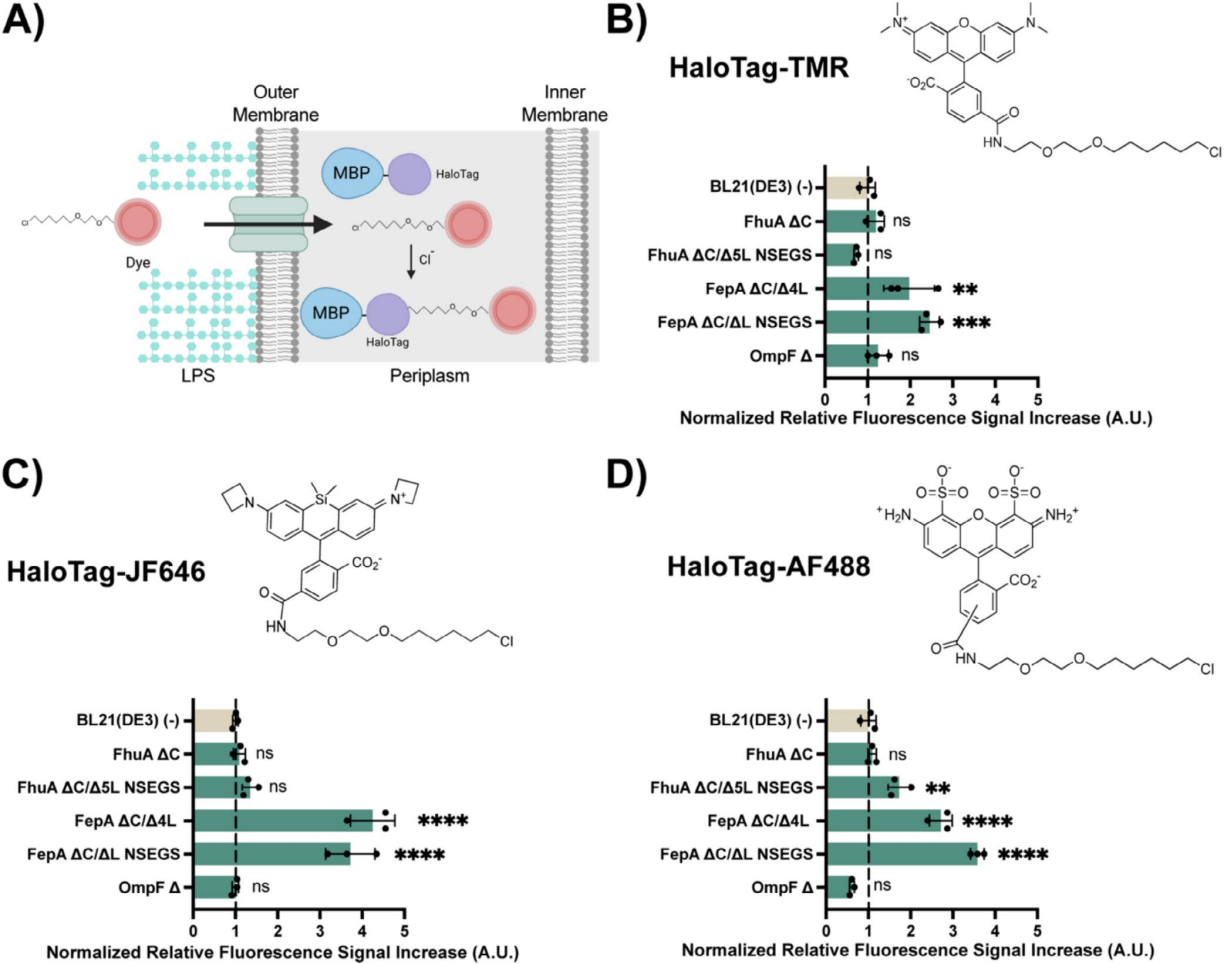
Flow cytometry analysis of the effect of OMP production on HaloTag protein labelling. (A) Schematic representation of the HaloTag labelling method to fluorescently label the periplasmic fusion protein MBP:Halo. (B–D) Increase in HaloTag‐TMR (636 Da), HaloTag‐JF646 (816 Da) and HaloTag‐AF488 (999 Da) fluorescence signal of the BL21(DE3) strains producing the MBP:Halo periplasmic fusion protein and the indicated OMP, normalised to the fluorescence signal of the strain producing the same MBP:Halo periplasmic protein but no OMP ‘(−)’. Data shown correspond to the average and the standard deviation of three independent biological replicates. Individual replicates are represented by filled black circles. Dashed lines represent the normalised level of fluorescence shown by the control without OMP expression. An ANOVA with a significance level *α* = 0.05 test was carried out to determine statistically significant differences in comparison to the population overproducing no OMP (−). *P* values > 0.05 are indicated with 'ns', *p* values ≤ 0.01 are indicated with '**', *p* values ≤ 0.001 are indicated with '***' and *p* values ≤ 0.0001 are indicated with '****'. The full data set can be found in Tables S6–S8.

The obtained results indicate again that the overproduction of specific OMPs improves the labelling results for all three tested dyes, although to different extents depending on the dye and the OMP variant (Figure 6, Figure S6). Specifically, FepA‐derived OMP variants consistently led to improvements in the extent of labelling, while, with the exception of FhuA ∆C/∆5L NSEGS and HaloTag‐AF488, FhuA or OmpF‐derived variants showed no significant improvement. Also, improvements were most notable for the dye of intermediate size, HaloTag‐JF646. As the differences in size and physicochemical properties between HaloTag‐TMR and HaloTag‐JF646 were moderate, this might point towards a size limit in the central channel of FepA‐derived variants around the extent of the aromatic triannular ring system.

**FIGURE 7:**
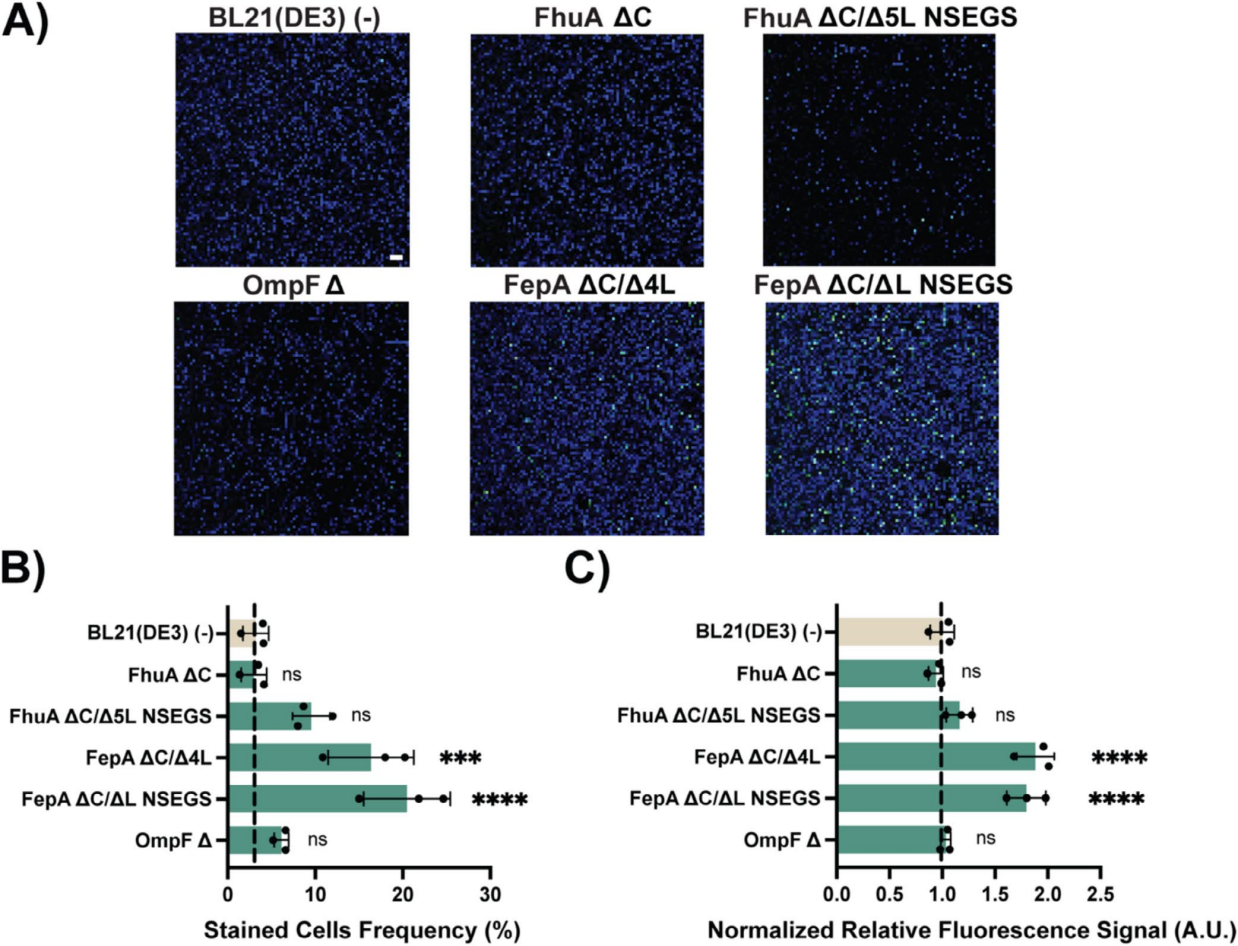
Microscopy analysis of the effect of OMP variant production on labelling with HaloTag‐AF488. (A) Representative overlay images obtained with confocal microscopy of BL21(DE3) cells overproducing periplasmic MBP:Halo and the indicated OMP variant stained with DAPI and HaloTag‐AF488. Scale bar, 10 μm. The corresponding original images for the DAPI and HaloTag‐AF488 channel can be found in Figure S7. (B) Percentage of observed cells stained with HaloTag‐AF488 observed with confocal microscopy technique. (C) Increase in HaloTag‐AF488 fluorescence signal of BL21(DE3) strains co‐producing the MBP:Halo periplasmic fusion protein and the indicated OMP. This experiment was performed with independent biological triplicates (*n* = 3) with at least 5000 counted cells. Data shown correspond to the average and the standard deviation of the HaloTag‐AF488 signal normalised to their relative amount of MBP:HaloTag. Individual replicates are represented by circles. Dashed lines represent the levels of fluorescence shown by the control without OMP expression. An ANOVA with a significance level *α* = 0.05 test was carried out to determine statistically significant differences in comparison to the population overproducing no OMP (−). *P* values > 0.05 are indicated with 'ns', *p* values ≤ 0.001 are indicated with '***' and *p* values ≤ 0.0001 are indicated with '****'. The full data set and the split channel pictures can be found in Table S9 and Figure S7.

As labelling experiments are often evaluated by microscopy, we also conducted one such experiment. Generally speaking, anionic compounds such as HaloTag‐AF488 are expected to pass the outer membrane less than other dyes since they are expected to be repelled by the negative charges present in the LPS component of the outer membrane (Kipper et al. 2016). Therefore, we selected this dye to illustrate the effect of OMP variant co‐expression on labelling by fluorescence microscopy (Figure 7). We stained cells coproducing MBP:HaloTag and the indicated OMP variants with HaloTag‐AF488 and 4′,6‐diamidino‐2‐phenylindole (DAPI), a membrane‐permeant dye used in this assay to locate the *E. coli* cells. Consistent with the results obtained in the flow cytometry assay, the overproduction of FepA ∆C/∆4L and FepA ∆C/∆L NSEGS showed the best improvement of labelling results in the microscopy assay. On the one hand, the fraction of labelled cells increased from 3.2% to 16.4% and 20.5%, respectively (Figure 7B). On the other hand, co‐expression of FepA ∆C/∆4 L and FepA ∆C/∆L NSEGS significantly enhanced the intracellular HaloTag‐AF488 signal among the stained cells, resulting in a 1.9‐ and 1.8‐fold increase, respectively (Figure 7C).

## 4 Discussion

The outer membrane of *E. coli* functions as a selective barrier for molecular transport, a critical attribute for the correct functioning of the cell. This work focuses on systematically comparing native and engineered OMPs that have been used or suggested in the literature to support the passage of such molecules across the outer membrane. With respect to the performance of monomeric OMP variants, a general trend was observed in that engineered OMPs showed stronger permeabilisation effects than their native counterparts. An exception was the lower permeabilisation of FepA ∆C128 and FepA ∆C for erythromycin uptake, hinting indirectly at the importance of extramembrane loops, as discussed above. Notably, removing the extracellular loops that occlude the channel after the removal of the cork (Mohammad et al. 2011) in engineered FepA variants enabled erythromycin diffusion through the channel.

Moreover, engineered FepA variants show higher permeabilisation effects compared to engineered FhuA variants for NPN and erythromycin, indicating a potentially higher permeabilisation capacity for FepA despite their similar functional and structural characteristics, being both ferric siderophore transporters composed of 22 β‐strands and with an estimated channel diameter of 30 Å (Usher et al. 2001). Also, when we tested the most permeabilising OMP variants in two specific applications, the import of a metal‐containing cofactor for ArM‐based whole‐cell biocatalysis and fluorescent labels for protein labelling, we found a superior performance of strains producing FepA‐derived variants over strains producing FhuA‐derived variants.

More generally speaking, our results for FhuA and FepA variants on increased cell‐specific activity in periplasmic ArM‐biocatalysis results are consistent with previous studies, which reported improved catalytic activities for ArM‐based whole‐cell biocatalysis when increasing outer membrane permeability or using more permeant compounds (Liu et al. 2022; Jeschek, Reuter, et al. 2016; Chordia et al. 2021). Furthermore, our results are also consistent with earlier experiments in which *E. coli* native ChuA transporter (not included in this study due to the lack of crystallographic and structural data) was overproduced (Reynolds et al. 2017), or when the *hug* operon from *Plesiomonas shigelloides* was used to enable the engineering of new ArM‐based biocatalytic pathways inside the cell (Huang et al. 2021).

Interestingly, the expression of FepA and FhuA‐derived variants also facilitated periplasmic protein labelling, and also here following along the trend of the degree of engineering, showing FepA‐derived variants a higher effect than FhuA‐derived variants. Periplasmic labelling in live bacteria is often hindered by the restricted permeability of the bacterial envelope to organic dyes (Yang and Weisshaar 2018; Ongwae et al. 2023). We demonstrated that the incorporation of OMP transporters could enhance to some extent the permeability of the outer membrane. Interestingly, the largest improvement was found for the compound with the highest molecular weight (HaloTag‐AF488, 999 Da) but with the smallest predicted molecular diameter (approximately 11 Å diameter), compared to HaloTag‐TMR (636 Da, approx. 16 Å) and HaloTag‐JF646 (816 Da and approx. 15 Å) (Tansila et al. 2007).

Finally, it should be noted that variants of the one general OMP in this study, derived from OmpF, did not perform similarly well in the different experiments. Apparently, the size limit of the central OmpF channel and the restrictions on the physicochemical nature of the diffusing compound are more restrictive than for the variants of the specific OMPs with reduced selectivity.

The reported results make it tempting to conclude that the FepA variants ∆C/∆4L and ∆C/∆L NSEGS, with the cork domain removed and the extramembrane loops shortened, seem to provide the least resistance to the passage of a variety of compounds, and therefore represent a good choice to start future permeabilisation efforts. While we certainly argue that our results point in this direction, we also should mention factors that limit the generality of this statement. It is important to note that we prioritised the overall effect of expression of the OMP variants over detailing protein‐specific activities (requiring quantification of active proteins) except for the effect of expression on a BL21 strain lacking the most important OMPs (Figure 2). We reasoned that from a practical point of view, the combined effect of variant expression, construct stability and overall performance is the critical factor in determining which plasmid construct is optimal for specific applications. Therefore, while we cannot disentangle which specific factor leads to the superior performance of FepA‐derived OMP variants, we can clearly state that in terms of effect for a variety of applications, the FepA variants ∆C/∆4L and ∆C/∆L NSEGS are the best candidates.

To address the potential impact of expression levels on permeabilisation capabilities, we employed the rhamnose‐inducible promoter P_*rhaBAD*_ to titrate OMP expression levels. The results demonstrated clear differences between conditions with and without induction of OMP expression; however, no significant differences were observed across varying induction levels for the same OMP (Figures S8 and S9). This suggests that the structural features and channel dimensions of the OMPs play a more pivotal role in determining their permeabilisation capabilities than the abundance of the proteins in the membrane. Additionally, at induction levels above 0.2% (w/v), a detrimental effect was observed for FepA ∆C/∆L NSEGS, likely due to the saturation of the SecYEG translocation system from excessive OMP production (Schlegel et al. 2012). Previous studies on OM permeabilisation have highlighted the detrimental effects on cell growth and viability caused by the leakage of periplasmic proteins such as RNAses and β‐lactamases (Kastenhofer et al. 2021). While it is unlikely that such large proteins would leak through the OMP variants studied here due to the dimensions of their channels, we observed a general trend in which strains producing permeabilising OMPs showed impaired growth. These observed toxic effects could be attributed to the leakage of certain unidentified small periplasmic components, but they could also result from the cell burden associated with the production of membrane proteins, which can strain the cellular machinery involved in protein production, periplasmic translocation and folding (Schlegel et al. 2012).

It should be mentioned that despite the improvements to the results, overexpression of OMP variants did not lead to homogeneous results when single cells were investigated, as shown for the labelling experiments (Figure 7, see also the quite broad signal distribution during flow cytometry as reflected in the large standard deviations of the mean values reported in Tables S6–S9). Put differently, we cannot exclude that rather than a somewhat uniform increase in the permeability of each cell in an experiment, we are looking at results that are aggregates of different performance values per cell. In this context, is it, for example, interesting to see that one major effect of the overexpression of FepA variants ∆C/∆4L and ∆C/∆L NSEGS was that the fraction of labelled cells increased, but only to a maximum of around 20% (Figure 7b).

This illustrates that there are clearly more factors that still remain to be optimised before genetically encoded permeabilisation of the outer membrane reaches its full potential. However, from a practical point of view, we argue that we provide here a very useful comparison of permeabilisation strategies that suggest that expressing the new highly engineered OMP variants FepA ∆C/∆4L and FepA ∆C/∆L NSEGS provides the best experimental outcome across a wide range of different experiments.

## Author Contributions

**Ivan Casas‐Rodrigo:** writing – original draft, writing – review and editing, visualization, validation, conceptualization, investigation, methodology, formal analysis, data curation. **Tobias Vornholt:** writing – review and editing, methodology, investigation. **Kathrin Castiglione:** writing – review and editing, supervision, conceptualization, resources. **Tania Michelle Roberts:** writing – review and editing, writing – original draft, supervision, project administration, investigation, formal analysis, conceptualization. **Markus Jeschek:** writing – review and editing, supervision, resources, conceptualization. **Thomas R. Ward:** conceptualization, writing – review and editing, supervision. **Sven Panke:** writing – original draft, writing – review and editing, supervision, conceptualization, project administration, funding acquisition.

## Conflicts of Interest

The authors declare no conflicts of interest.

## Supporting information


Data S1

## Data Availability

The data that support the findings of this study are available from the corresponding author upon reasonable request.
