# Supplementary material for: Permeabilisation of the Outer Membrane of Escherichia coli for Enhanced Transport of Complex Molecules
Source: Microb Biotechnol. 2025 Mar 9;18(3):e70122. doi: 10.1111/1751-7915.70122 (PMC11891017; doi:10.1111/1751-7915.70122)
Supplement: Supplementary file 1 — Data S1 [file MBT2-18-e70122-s001.pdf]

Supplementary Material

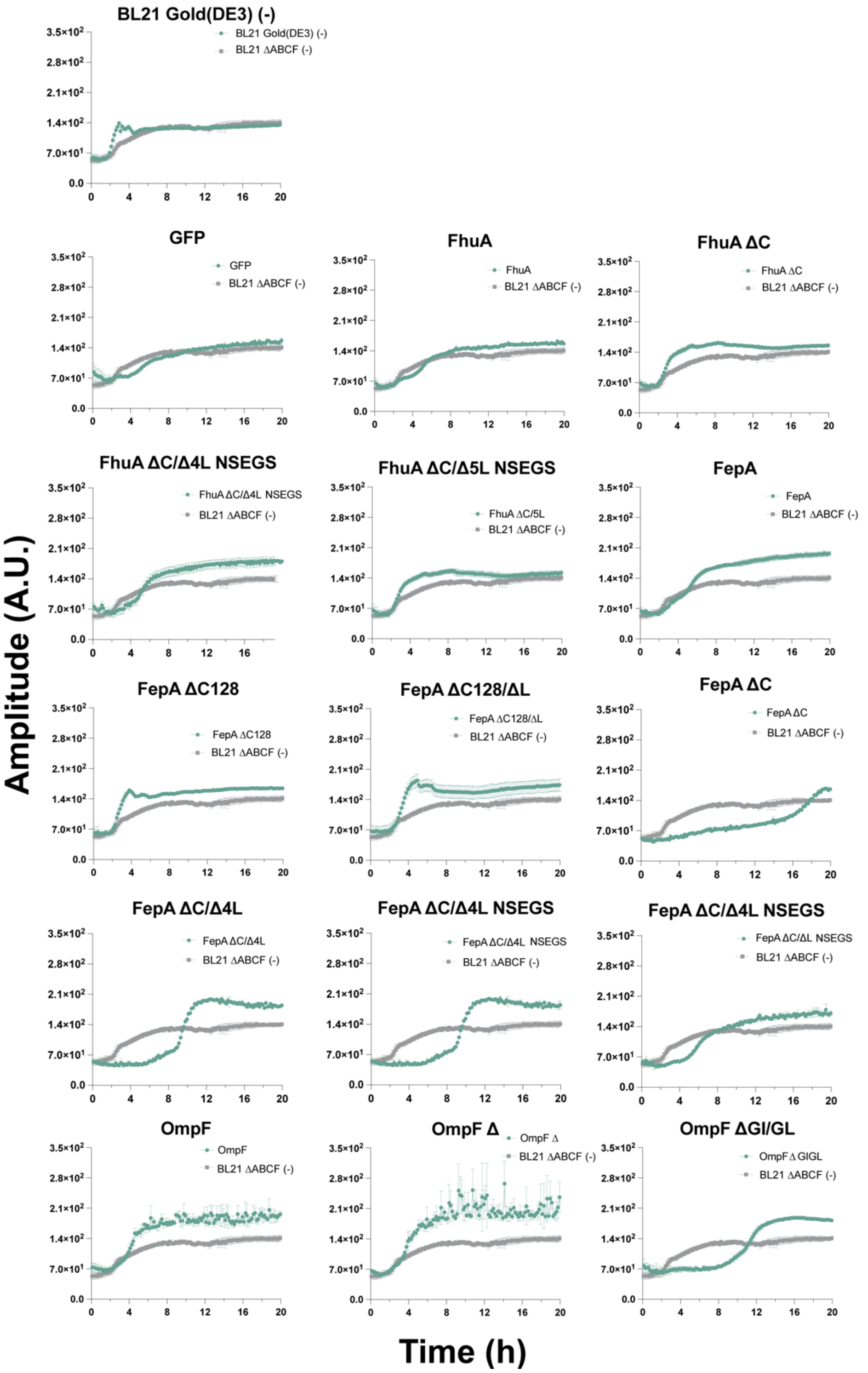

Supplementary Figure 1. **Growth behavior of BL21ΔABCF overproducing the indicated proteins.** Independent triplicates of BL21ΔABCF strains producing none (grey) or the indicated protein (green) were seeded in a deep-well plate and incubated at 37°C in LB. Growth was monitored as a function of backscatter and the amplitude is plotted. Growth of BL21ΔABCF in grey and the parental strain, BL21 Gold (DE3) carrying a plasmid with no OMP (-), are shown for comparison for the negative controls. The GFP expressing strain serves as an indicator of the effect of metabolic burden on the growth of BL21ΔABCF.

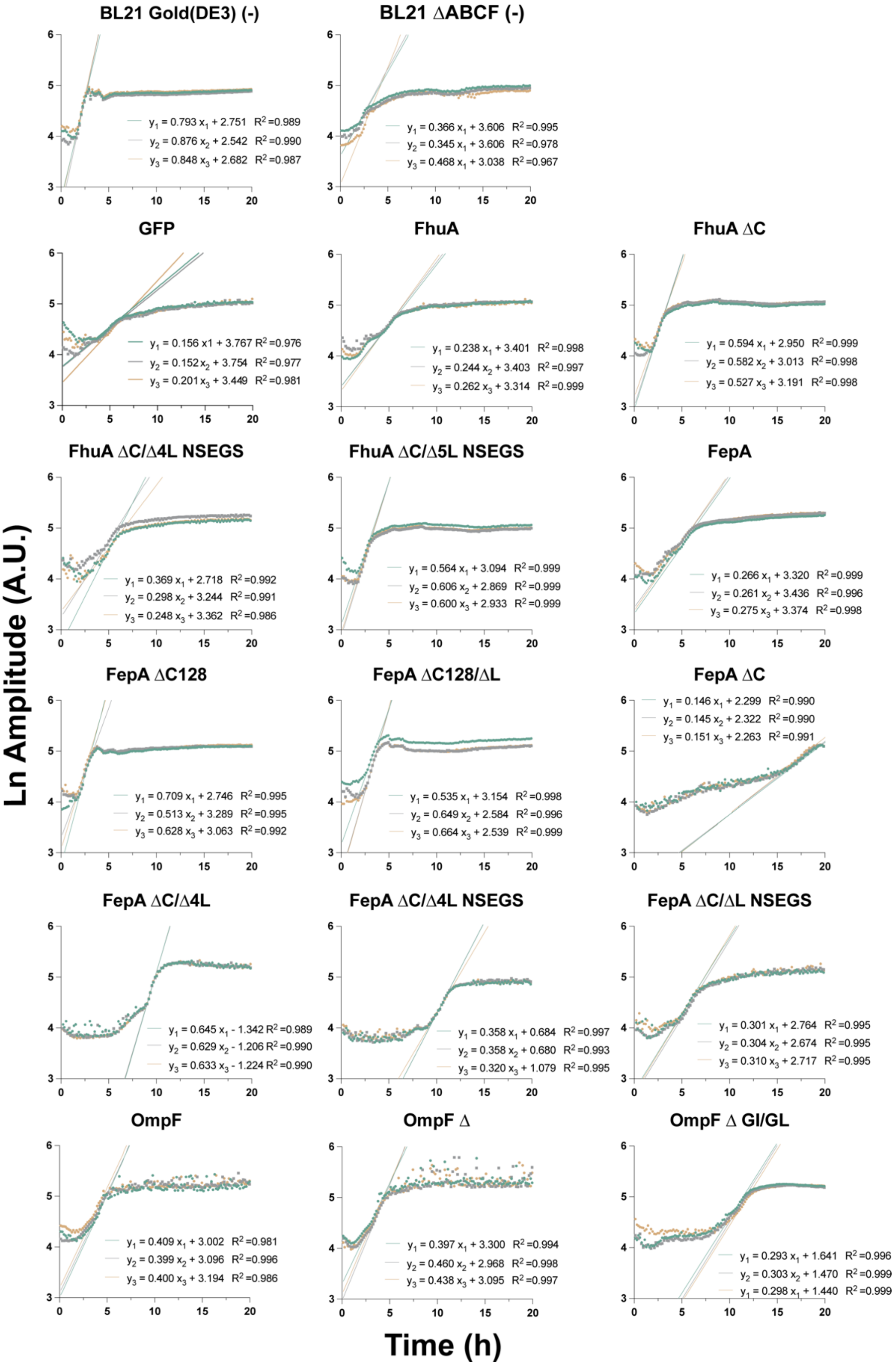

Supplementary Figure 2. **Calculation of maximum specific growth rates of the BL21ΔABCF strains overproducing the indicated proteins.** Independent triplicates of BL21ΔABCF strains producing none (-) or the indicated protein were seeded in a deep-well plate and incubated at 37°C in LB. Growth was monitored as a function of backscatter and the amplitude is plotted. The natural logarithmic values of the growth are plotted and were used to determine the maximum specific growth rate for each replicate. BL21 Gold (DE3) strain used for comparison for the negative controls. The GFP expressing strain serves as an indicator of the effect of metabolic burden on the growth of BL21ΔABCF.

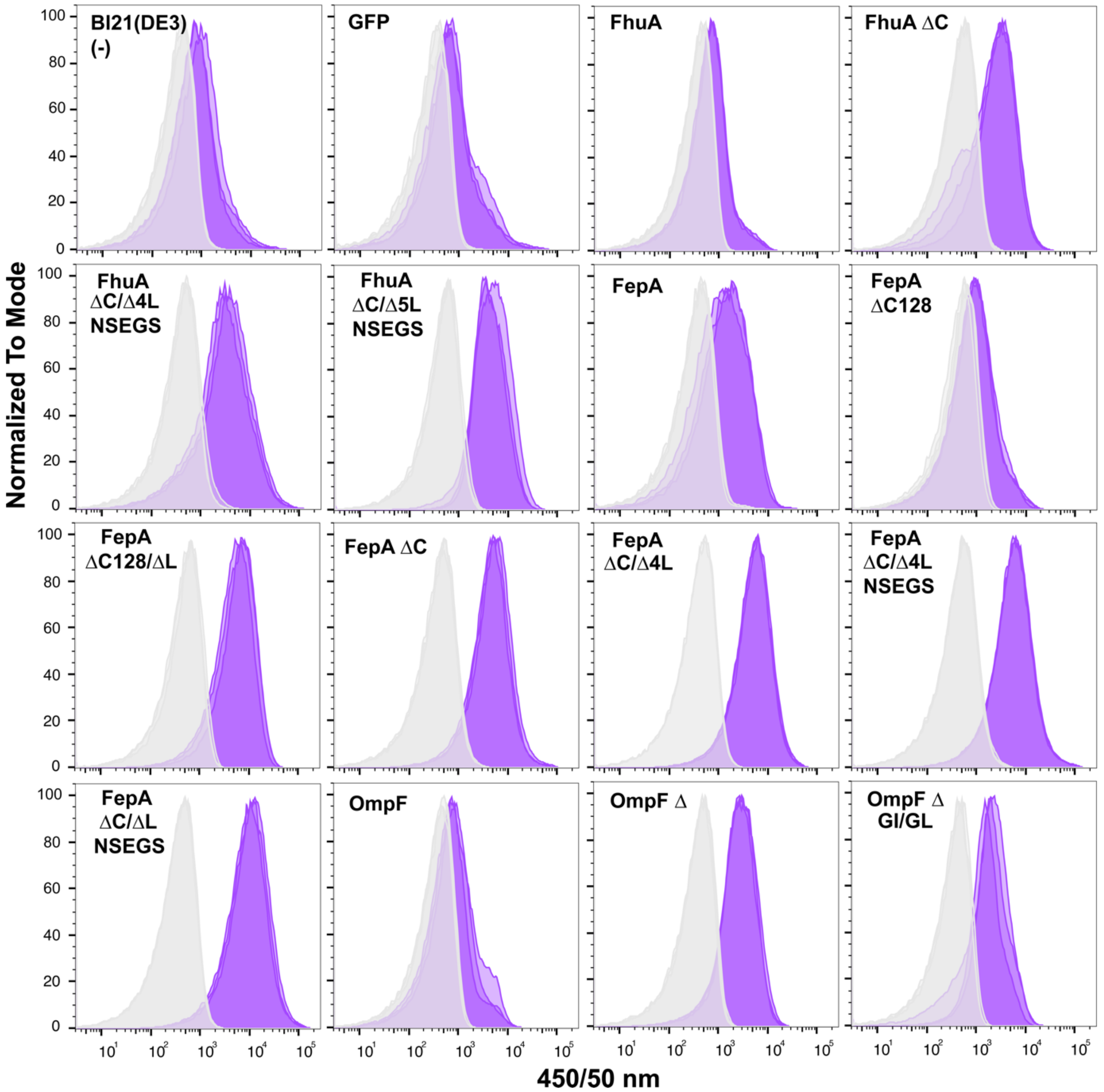

Supplementary Figure 3. Flow cytometry analysis of the effect of OMP overproduction on NPN import. BL21 (DE3) strains overproducing no protein (-) or the indicated protein were grown and incubated with (purple) or without (grey) 10 µM NPN, washed, resuspended in PBS and fluorescence (ex 355 nm, em 450/50 nm) was analyzed via flow cytometry. This experiment was performed with independent biological triplicates (n=3) with at least 80000 single events. The cell count of the populations displayed in the histograms was normalized to their corresponding mode for visualization purposes.

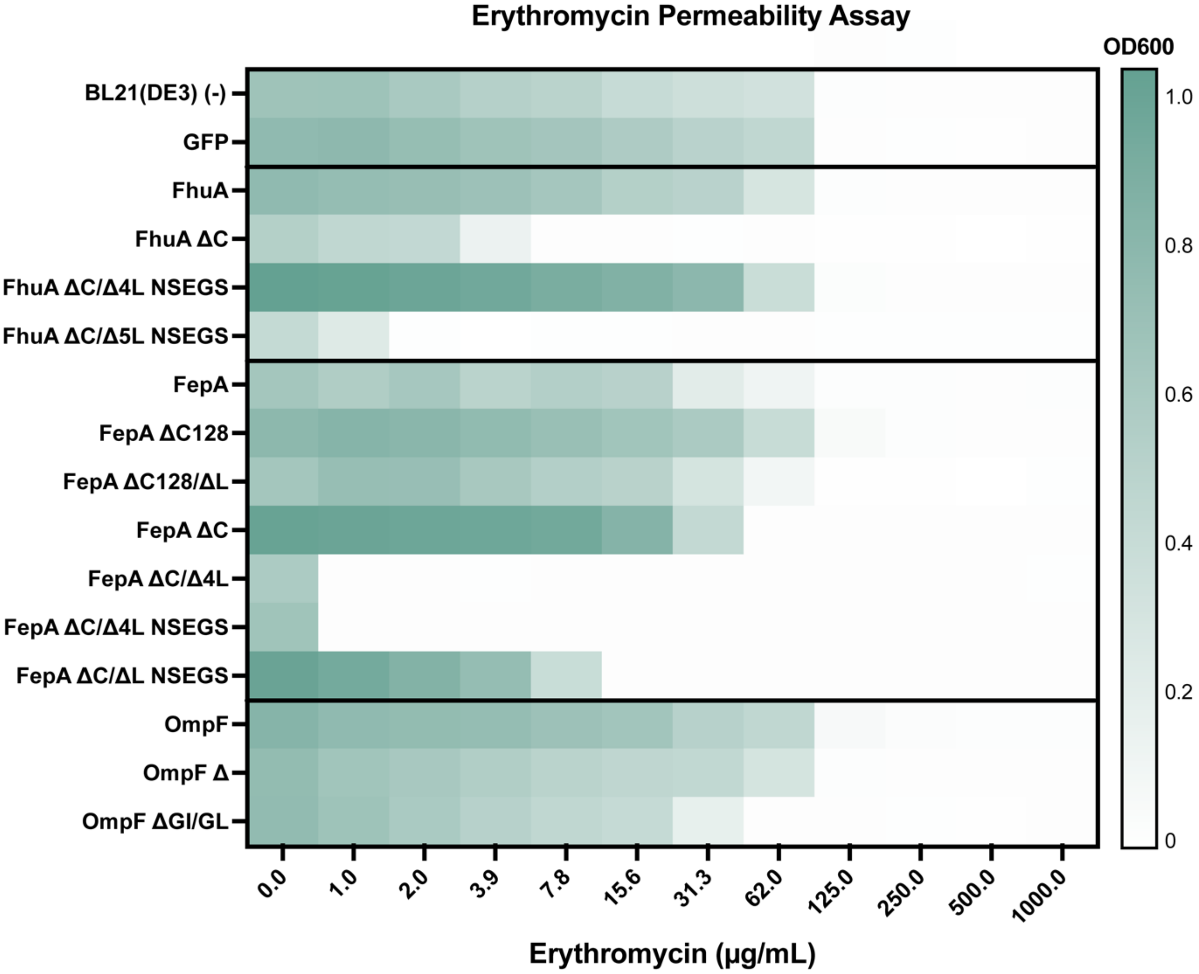

Supplementary Figure 4. Erythromycin permeability assay. BL21 (DE3) strains overproducing no protein (-) or the indicated protein were grown in LB supplemented with 50 mg·L^{-1} of kanamycin, 50 µM IPTG and the indicated concentration of erythromycin. This experiment was performed with independent biological triplicates (n=3).Heat map graph displays the OD_{600} values observed for the OMP overproducing strains and the indicated erythromycin concentrations after 18 h of incubation at 37°C. The data shown for FhuA ΔC/Δ4L NSEGS correspond to cell cultures that possessed a disrupted version of the OMP gene when analyzing after the experiment.

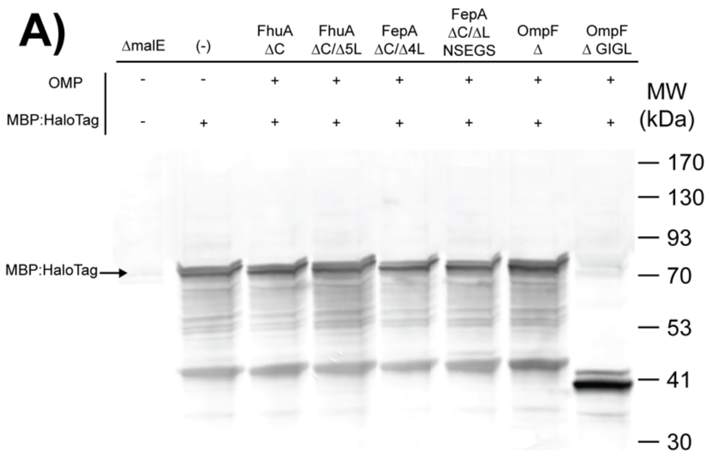

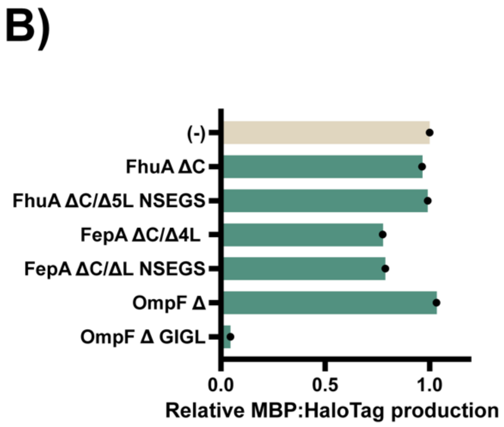

Supplementary Figure 5. MBP:HaloTag production in BL21(DE3) Δ*malE* overproducing different OMPs. A) Western Blot for MBP:HaloTag quantification. Cell lysates were electrophoresed by A 4-20% SDS-PAGE electrophoresis was carried out with the cell lysates of the indicated strains and transferred on to nitrocellulose membrane before treating them with a primary anti-MBP antibody and secondary fluorescent antibody for detection. The expected sizes for MBP, HaloTag and the MBP:HaloTag fusion protein are 43.4, 33.5 and 78.7 kDa respectively. B) Quantification of cellular MBP:HaloTag normalized to the MBP:HaloTag present in the BL21(DE3) Δ*malE* strain overproducing MBP:HaloTag but no OMP.

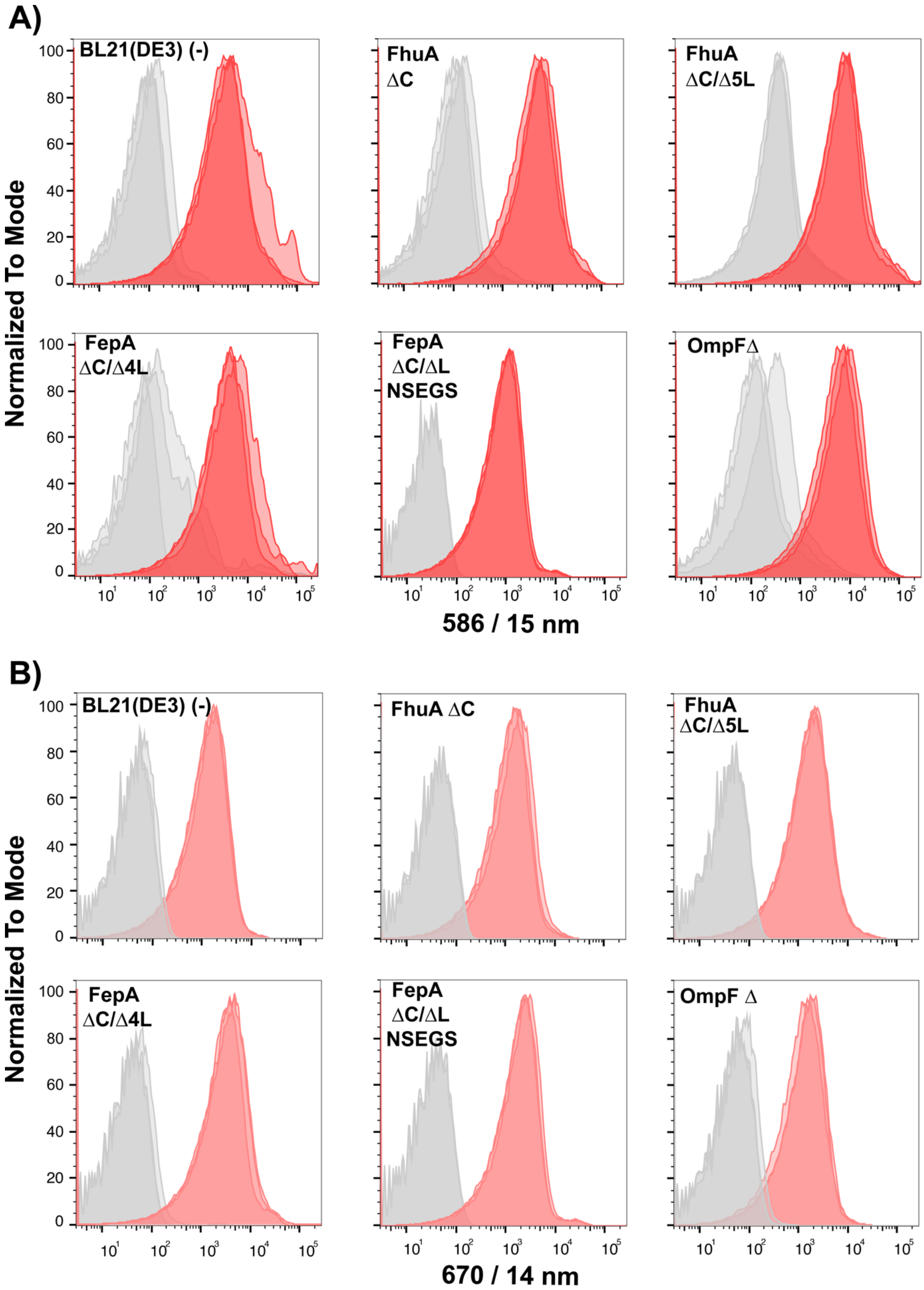

Supplementary Figure 6. Caption in next page.

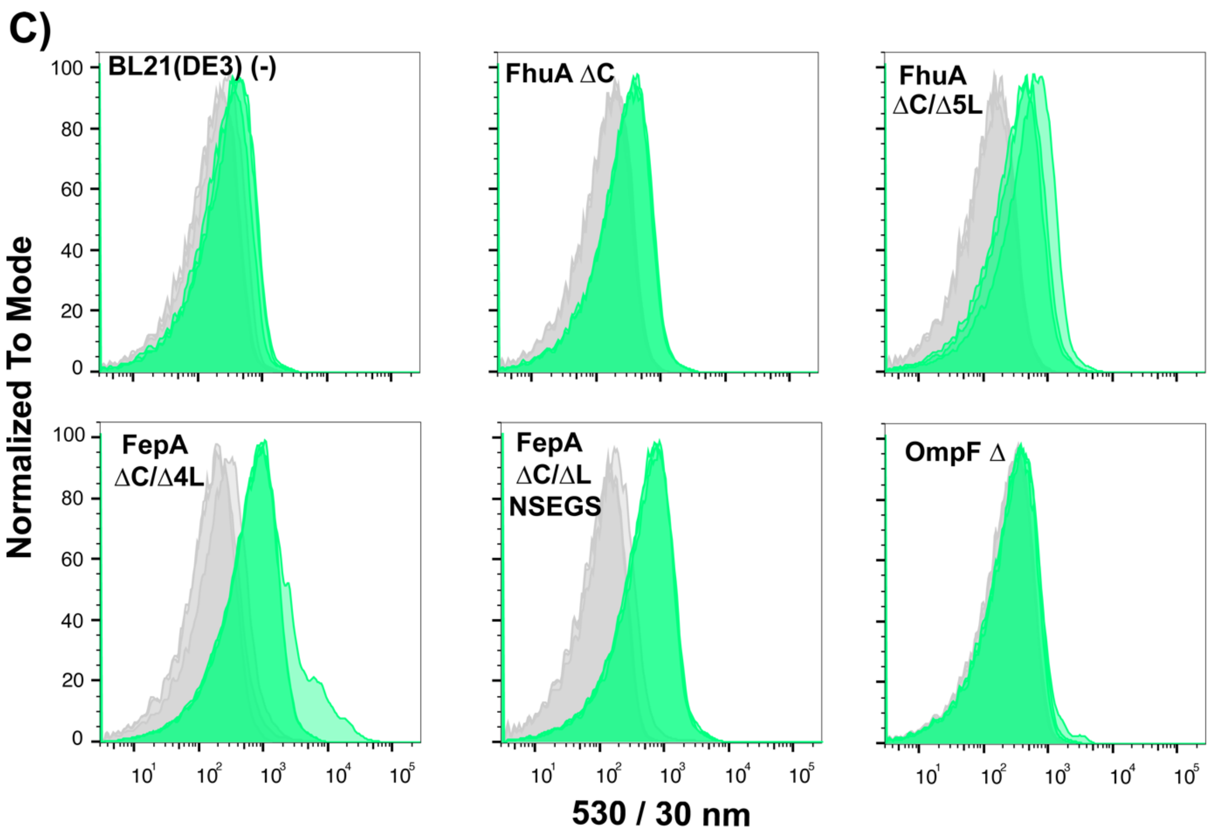

Supplementary Figure 6. Flow cytometry analysis of the effect of OMP overproduction on HaloTag dyes import. BL21 (DE3) strains overproducing the MBP:HaloTag fusion protein, and no protein (-) or the indicated protein were grown and incubated with (color) or without (grey) 1 µM of HaloTag TMR (A), HaloTag JF646(B) or HaloTag AF488 (C), washed, resuspended in PBS and fluorescence was analyzed via flow cytometry (ex 561nm, em 686/15nm; ex 640nm, em 670/14nm; ex 488nm, em 530/30nm). This experiment was performed with independent biological triplicates (n=3) with at least 80000 single events. The cell count of the populations displayed in the histograms was normalized to their corresponding mode for visualization purposes.

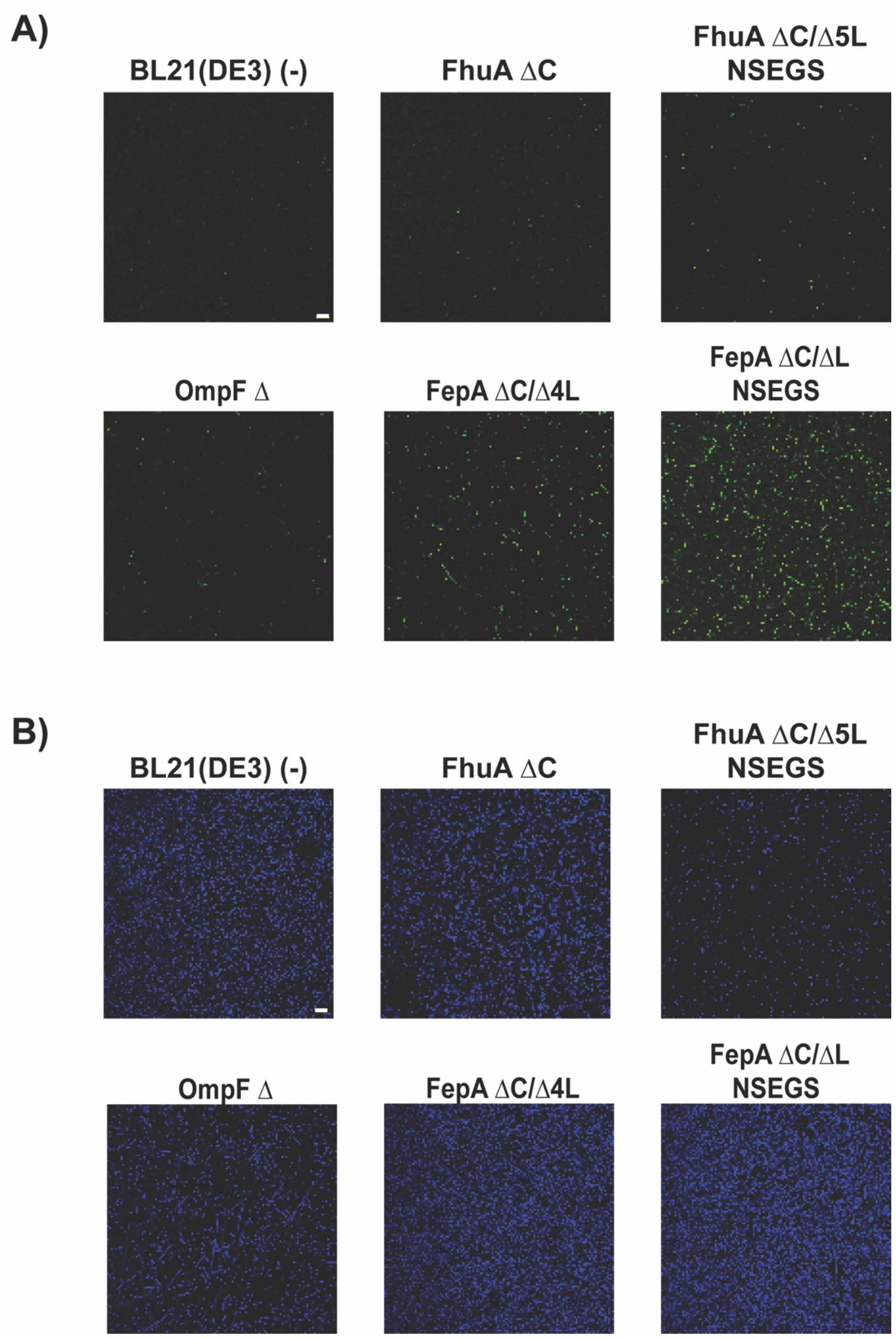

Supplementary Figure 7. Channel-split microscopy images of the effect of OMP production on HaloTag protein labeling with HaloTag-AF488. BL21 (DE3) strains overproducing the MBP:HaloTag fusion protein, and no protein (-) or the indicated protein were grown incubated with (color) or without (grey) 1 µM of HaloTag TMR (A), HaloTag JF646(B) or HaloTag AF488 (C), washed, resuspended in PBS and fluorescence was analyzed via microscopy. A) Images obtained with confocal microscopy of BL21(DE3) cells overproducing periplasmic MBP:Halo and the indicated OMP stained with DAPI and HaloTag-AF488 for the AF-488 channel. Scale bar, 10 µm. B) Images obtained with confocal microscopy of BL21(DE3) cells overproducing periplasmic MBP:Halo and the indicated OMP stained with DAPI and HaloTag-AF488 for the DAPI channel. Scale bar, 10 µm.

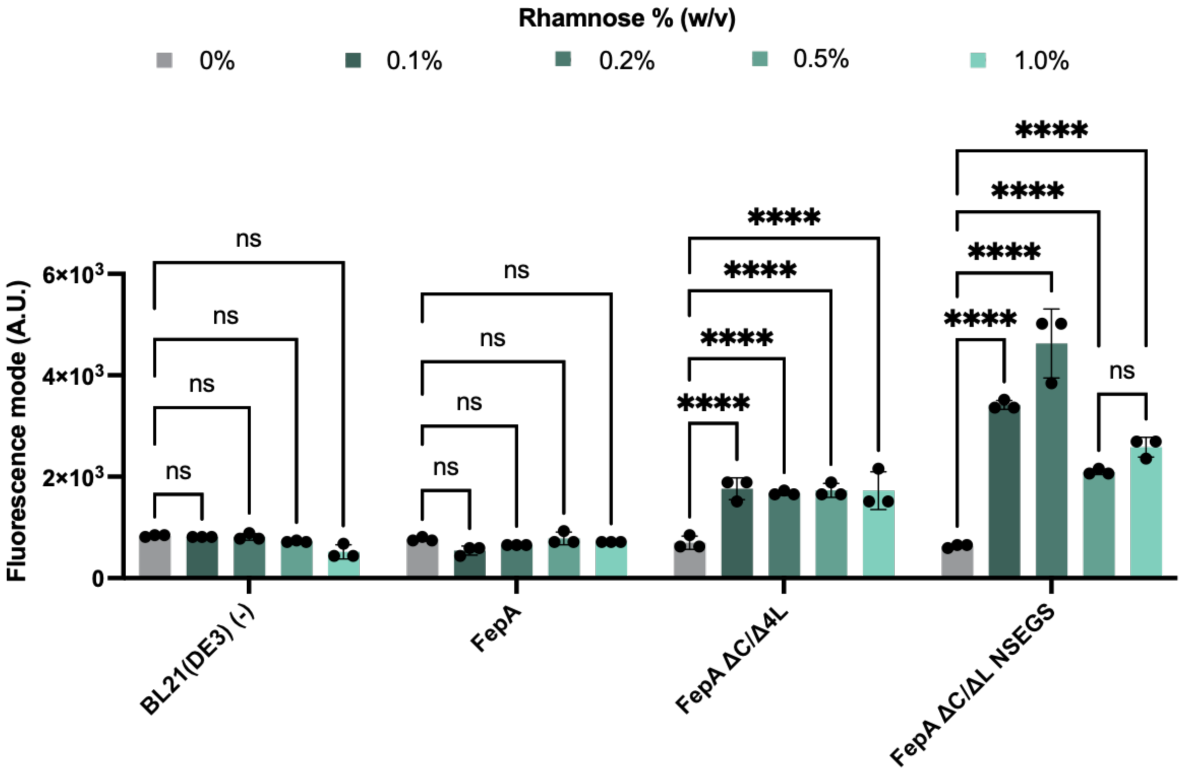

Supplementary Figure 8. Flow cytometry analysis of the effect of different expression induction levels on NPN import. Fluorescence mode of OMP overproducing strains and controls after induction with different concentrations of L-rhamnose. BL21 (DE3) strains overproducing no protein (-) or the indicated OMP, which gene expression was regulated by the rhamnose-inducible promorter P_{RhaBAD}, were grown and incubated with 0, 0.1, 0.2, 0.5 or 1.0% (w/v) L-Rhamnose. After wash and resuspension in PBS, cells were incubated with (purple) or without (grey) 10 µM NPN, washed and resuspended in PBS again. Cell fluorescence (ex 355 nm, em 450/50 nm) was analyzed via flow cytometry. This experiment was performed with independent biological triplicates (n=3) with at least 80000 single events. The values displayed here correspond to the statistical mode of the indicated population.

Supplementary Table 1. Complete set of OMPs and their different engineered versions assessed in this study.

OMP	Version	Structure mutated	Reference
**OmpF**	Wild type		Rosenbusch et al. 1992
	Δ	ΔG109_L114	Saint et al. 1996
	ΔGIGL	ΔG109_L114 + Residues for trimer formation	This study
**FhuA**	Wild type		Coulton et al. 1986
	ΔC	ΔA1_P160	Mohammad et al. 2011
	ΔC/Δ4L NSEGS	ΔA1_P160<br>ΔY243-N273::NSEGS<br>ΔY315-A342::NSEGS<br>ΔD394_N419::NSEGS<br>ΔN682_R694::NSEGS	Mohammad et al. 2011
	ΔC/Δ5L NSEGS	ΔA1_P160<br>ΔY243-N273::NSEGS<br>ΔY315-A342::NSEGS<br>ΔD394_N419::NSEGS<br>ΔG640_S652::NSEGS<br>ΔN682_R694::NSEGS	Wolfe et al. 2016
**FepA**	Wild type		Lundrigan and Kadner 1986
	ΔC	ΔQ1_S150	This study
	ΔC128	ΔQ1_P128	This study
	ΔC128/ΔL	ΔQ1_P128<br>ΔK167_E169<br>ΔD201_G224<br>ΔG262_D279<br>ΔE319_Q335<br>ΔS378_P403<br>ΔS470_L502<br>ΔV541_G549<br>ΔT595_I602<br>ΔQ631_I651<br>ΔL683_P715	This study
**FepA**	ΔC/Δ4L	ΔQ1_S150<br>ΔD201_G224<br>ΔS378_P403<br>ΔS470_L502<br>ΔL683_P715	This study
	ΔC/Δ4L NSEGS	ΔQ1_S150<br>ΔQ199_E226::NSEGS<br>ΔD376_S405::NSEGS<br>ΔA468_A504::NSEGS<br>ΔK681_T715::NSEGS	This study
	ΔC/ΔL NSEGS	ΔQ1_P128<br>ΔE165_T172::NSEGS<br>ΔQ199_E226::NSEGS<br>ΔY260_T281::NSEGS<br>ΔI317_F337::NSEGS<br>ΔD376_S405::NSEGS<br>ΔA468_A504::NSEGS<br>ΔG539_D551::NSEGS<br>ΔK593_E604::NSEGS<br>ΔG629_P653::NSEGS<br>ΔK681_T715::NSEGS	This study

Supplementary Table 2. **Analysis of the permeability of *E. coli* strains overproducing OMPs to the aromatic hydrophobic molecule NPN.** BL21 (DE3) strains overproducing no protein (-) or the indicated protein were grown and incubated with (purple) or without (grey) 10 µM NPN, washed, resuspended in PBS and fluorescence (ex 355 nm, em 450/50 nm) was analyzed via flow cytometry. This experiment was performed with independent biological triplicates (n=3) with at least 80000 single events. The values shown correspond to the average and standard deviation of the triplicates for the fluorescence mean, median and mode of the studied populations.

		Fluorescence signal (A.U.)		
**Overproduced protein**	**NPN (µM)**	**Mean**	**Median**	**Mode**
(-)	0	373.0 ± 11.4	316.3 ± 10.0	437.3 ± 49.9
GFP	0	327.0 ± 7.5	275.3 ± 7.1	371.7 ± 39.3
FhuA	0	391.3 ± 6.1	348.7 ± 6.4	472.0 ± 65.2
FhuA ΔC	0	512.7 ± 18.8	435.0 ± 13.0	641.7 ± 60.7
FhuA ΔC/Δ4L NSEGS	0	494.7 ± 49.9	405.0 ± 30.3	499.0 ± 44.5
FhuA ΔC/Δ5L NSEGS	0	549.7 ± 39.8	478.7 ± 33.1	631.3 ± 43.4
FepA	0	436.3 ± 23.7	338.7 ± 16.9	441.3 ± 86.3
FepA ΔC128	0	532.7 ± 55.6	469.3 ± 50.9	587.7 ± 55.4
FepA ΔC128/ΔL	0	575.7 ± 46.1	504.7 ± 42.5	650.0 ± 29.0
FepA ΔC	0	462.0 ± 16.6	383.7 ± 14.4	507.3 ± 57.6
FepA ΔC/Δ4L	0	887.3 ± 753.2	745.7 ± 623.8	968.7 ± 796.2
FepA ΔC/Δ4L NSEGS	0	551.0 ± 21.1	447.0 ± 9.8	512.7 ± 12.7
FepA ΔC/ΔL NSEGS	0	418.7 ± 8.4	355.3 ± 5.5	498.0 ± 22.0
OmpF	0	406 ± 19.0	359.7 ± 16.2	476.7 ± 37.5
OmpF Δ	0	429 ± 22.0	379.0 ± 17.5	529.7 ± 48.1
OmpF Δ GIGL	0	395 ± 26.9	352.7 ± 24.9	442.3 ± 21.9

		Fluorescence signal (A.U.)		
**Overproduced protein**	**NPN (µM)**	**Mean**	**Median**	**Mode**
(-)	10	1354.0 ± 276.4	737.7 ± 127.6	796.3 ± 149.5
GFP	10	1394.3 ± 136.0	612.3 ± 30.9	673.7 ± 92.0
FhuA	10	918.3 ± 36.3	623.0 ± 21.9	699.7 ± 17.9
FhuA ΔC	10	3144.7 ± 439.7	2323.0 ± 422.2	3475.3 ± 327.5
FhuA ΔC/Δ4L NSEGS	10	5306.3 ± 688.8	3140.0 ± 481.1	6363.0 ± 164.5
FhuA ΔC/Δ5L NSEGS	10	5717.0 ± 581.9	4324.0 ± 391.9	4173.3 ± 1136.8
FepA	10	2068.0 ± 103.5	1267.7 ± 175.7	1607.3 ± 344.1
FepA ΔC128	10	1334.0 ± 20.9	840.3 ± 8.0	973.3 ± 111.0
FepA ΔC128/ΔL	10	7027.0 ± 825.5	5790.0 ± 757.7	6760.7 ± 1470.6
FepA ΔC	10	6357.0 ± 624.3	4753.7 ± 458.2	5421.0 ± 1277.4
FepA ΔC/Δ4L	10	6604.3 ± 332.8	5217.0 ± 235.7	6090.3 ± 307.7
FepA ΔC/Δ4L NSEGS	10	7964.3 ± 176.7	5568.7 ± 128.3	5916.7 ± 397.0
FepA ΔC/ΔL NSEGS	10	12867.0 ± 1239.3	9628.3 ± 954.4	12109.3 ± 1661.7
OmpF	10	1151.7 ± 138.0	700.3 ± 54.2	788.7 ± 54.1
OmpF Δ	10	3300.0 ± 141.6	2643.0 ± 74.0	2777.3 ± 144.3
OmpF Δ GIGL	10	2373.7 ± 373.1	1818.3 ± 316.3	1978.7 ± 429.4

Supplementary Table 3. **Values of the replicates of the erythromycin MIC assay.** BL21 (DE3) strains overproducing no protein (-) or the indicated protein were grown in media with varying concentration of erythromycin ranging from 1 mg/mL to 1 µg/mL. The values for each of the independent biological triplicates (n=3), is indicated. The data shown for FhuA ΔC/Δ4L NSEGS correspond to cell cultures with disrupted version of the OMP gene.

Erythromycin minimal growth inhibitory concentration (µg/ml)

Overproduced protein	Rep.1	Rep.2	Rep.3	Mean
(-)	125.0	125.0	125.0	125.0
GFP	125.0	125.0	125.0	125.0
FhuA	125.0	125.0	125.0	125.0
FhuA ΔC	3.9	3.9	7.8	5.2
FhuA ΔC/Δ4L NSEGS	125.0	125.0	125.0	125.0
FhuA ΔC/Δ5L NSEGS	1.0	2.0	1.0	1.3
FepA	31.3	62.5	31.3	41.7
FepA ΔC128	125.0	125.0	125.0	125.0
FepA ΔC128/ΔL	62.5	125.0	62.5	83.3
FepA ΔC	62.5	62.5	62.5	62.5
FepA ΔC/Δ4L	1.0	1.0	1.0	1.0
FepA ΔC/Δ4L NSEGS	1.0	1.0	1.0	1.0
FepA ΔC/ΔL NSEGS	15.6	15.6	7.8	13.0
OmpF	125.0	125.0	125.0	125.0
OmpF Δ	125.0	125.0	125.0	125.0
OmpF Δ GIGL	62.5	62.5	31.3	52.1

Supplementary Table 4. **Summary of the data obtained for the analysis of the effect of OMP production on growth, on outer membrane permeability to NPN and on *E. coli* sensitivity to erythromycin.** Data was normalized using the GFP overproducing strain as reference.

Overproduced protein	Growth of BL21 ΔABCF		NPN import to periplasm		Reduduction in erythromycin MIC	
	μ_{max}(h^{-1})	Norm.	Fluoresence mode (A.U.)	Norm.	MIC (µg·mL^{-1})	Norm.
GFP	0.15	1.00	674	1.00	125.0	1.00
FhuA	0.25	1.67	700	1.04	125.0	1.00
FhuA ΔC	0.57	3.80	3475	5.16	5.2	0.04
FhuA ΔC/Δ4L NSEGS	0.31	2.07	3380	5.01	125.0	1.00
FhuA ΔC/Δ5L NSEGS	0.59	3.93	6363	9.44	1.3	0.01
FepA	0.27	1.80	1607	2.38	31.3	0.33
FepA ΔC128	0.62	4.13	973	1.44	125.0	1.00
FepA ΔC128/ΔL	0.62	4.13	6761	10.03	83.3	0.67
FepA ΔC	0.15	1.00	5421	8.04	62.5	0.50
FepA ΔC/Δ4L	0.64	4.27	6090	9.04	1.0	0.01
FepA ΔC/Δ4L NSEGS	0.35	2.33	5917	8.78	1.0	0.01
FepA ΔC/ΔL NSEGS	0.31	2.07	12109	17.97	13.0	0.10
OmpF	0.40	2.67	789	1.17	125.0	1.00
OmpF Δ	0.43	2.87	2777	4.12	125.0	1.00
OmpF Δ GIGL	0.30	2.00	1978	2.93	52.1	0.42

Supplementary Table 5. **Values of the replicates of the coumarin de-allylation in *E. coli* periplasm.** BL21 Gold (DE3) strains overproducing Sav S112M K121R, and no protein (-) or the indicated protein were grown and incubated with 1 µM of the biotinylated ruthenium cyclopentadienyl (Biot-HQ)CpRu cofactor for 1h, washed, and then incubated with 100 µM allyl carbamate. The product concentration was quantified by measuring the fluorescence intensity with a Tecan Infinite® M1000 at an excitation of 394 nm and an emission of 460 nm and the values were normalized by dividing by the OD_{600} of the respective culture. This experiment was performed with independent biological triplicates (n=3) and the values shown correspond to their average and standard deviation.

	Coumarin Fluorescence	
**OMP**	**Fluorescence/OD600 (A.U.)**	**Normalized Fluorescence/OD600 (A.U.)**
(-)	7.73E+04 ± 1.12E+04	1.00E+00 ± 1.45E-01
FhuA ΔC	1.14E+05 ± 1.77E+04	1.48E+00 ± 2.29E-01
FhuA ΔC/Δ5L NSEGS	2.76E+05 ± 3.24E+04	3.57E+00 ± 4.20E-01
FepA ΔC/Δ4L	2.35E+05 ± 1.24E+04	2.83E+00 ± 1.50E-01
FepA ΔC/ΔL NSEGS	2.34E+05 ± 3.63E+04	2.83E+00 ± 4.39E-01
OmpF Δ	2.06E+05 ± 1.34E+04	2.67E+00 ± 1.74E-01
OmpF Δ GIGL	2.24E+05 ± 2.86E+03	2.90E+00 ± 3.70E-02

Supplementary Table 6. **Analysis of the permeability of *E. coli* strains overproducing OMPs to HaloTag TMR dye.** BL21 (DE3) strains overproducing the MBP:HaloTag fusion protein, and no protein (-) or the indicated protein were grown incubated with (color) or without (grey) 1 µM of HaloTag TMR, washed, resuspended in PBS and fluorescence was analyzed via flow cytometry. This experiment was performed with independent biological triplicates (n=3) with at least 5000 counted cells This experiment was performed with independent biological triplicates (n=3) with at least 80000 single events. The average and standard deviation of the fluorescence means, medians and modes are indicated.

		Fluorescence signal TMR (561 nm, 586/15 nm)		
**Sample**	**OMP**	**Mean**	**Median**	**Mode**
**DMSO**	(-)	1.17E+02 ± 3.48E+01	8.60E+01 ± 2.09E+01	1.20E+02 ± 2.73E+01
	FhuA ΔC	1.59E+02 ± 5.42E+01	1.03E+02 ± 2.60E+01	1.43E+02 ± 2.52E+01
	FhuA ΔC/Δ5L	6.25E+02 ± 4.51E+01	3.33E+02 ± 2.54E+01	3.82E+02 ± 2.83E+01
	FepA ΔC/Δ4L	1.03E+03 ± 8.47E+02	1.14E+02 ± 5..69E+01	1.04E+02 ± 3.54E+01
	FepA ΔC/ΔL NSEGS	2.95E+01 ± 1.53E-01	2.46E+01 ± 4.62E-01	1.94E+01 ± 0.00E+00
	OmpF Δ	3.88E+02 ± 1.92E+02	1.69E+02 ± 9.99E+01	2.15E+02 ± 1.45E+02
**TMR (561 nm, 586/15 nm)**	(-)	7.46E+03 ± 3.94E+03	3.64E+03 ± 1.03E+03	4.00E+03 ± 7.08E+02
	FhuA ΔC	7.13E+03 ± 4.09E+02	4.64E+03 ± 6.58E+02	5.46E+03 ± 9.25E+02
	FhuA ΔC/Δ5L	1.17E+04 ± 1.32E+03	6.94E+03 ± 6.08E+02	9.23E+03 ± 6.19E+02
	FepA ΔC/Δ4L	7.33E+03 ± 3.26E+03	4.00E+03 ± 1.04E+03	5.32E+03 ± 1.61E+03
	FepA ΔC/ΔL NSEGS	1.14E+03 ± 7.61E+01	8.37E+02 ± 5.84E+01	1.25E+03 ± 1.18E+02
	OmpF Δ	8.57E+03 ± 1.37E+03	6.22E+03 ± 1.16E+03	9.20E+03 ± 1.85E+03

Supplementary Table 7. **Analysis of the permeability of *E. coli* strains overproducing OMPs to HaloTag JF646 dye.** BL21 (DE3) strains overproducing the MBP:HaloTag fusion protein, and no protein (-) or the indicated protein were grown incubated with (color) or without (grey) 1 µM of HaloTag JF646, washed, resuspended in PBS and fluorescence was analyzed via flow cytometry. This experiment was performed with independent biological triplicates (n=3) with at least 5000 counted cells This experiment was performed with independent biological triplicates (n=3) with at least 80000 single events. The average and standard deviation of the fluorescence means, medians and modes are indicated.

		Fluorescence signal JF646 (640nm, 670/14nm)		
**Sample**	**OMP**	**Mean**	**Median**	**Mode**
**DMSO**	(-)	5.48E+01 ± 4.50E+00	4.37E+01 ± 3.98E+00	5.49E+01 ± 1.34E+01
	FhuA ΔC	4.64E+01 ± 1.56E+00	3.79E+01 ± 1.93E+00	4.72E+01 ± 8.70E-15
	FhuA ΔC/Δ5L	4.67E+01 ± 9.64E-01	3.79E+01 ± 1.10E+00	4.72E+01 ± 8.70E-15
	FepA ΔC/Δ4L	7.61E+01 ± 5.71E+01	3.82E+01 ± 4.79E+00	4.01E+01 ± 2.36E+01
	FepA ΔC/ΔL NSEGS	4.26E+01 ± 2.00E-01	7.50E+01 ± 5.77E-01	2.65E+01 ± 0.00E+00
	OmpF Δ	5.37E+01 ± 2.49E+00	4.28E+01 ± 9.00E-01	4.72E+01 ± 8.70E-15
**JF646 (640nm, 670/14nm)**	(-)	1.70E+03 ± 7.88E+01	1.28E+03 ± 7.70E+01	1.86E+03 ± 1.25E+02
	FhuA ΔC	1.75E+03 ± 2.57E+02	1.29E+03 ± 1.84E+02	1.69E+03 ± 2.24E+02
	FhuA ΔC/Δ5L	2.54E+03 ± 2.42E+01	1.66E+03 ± 4.14E+01	2.14E+03 ± 2.96E+02
	FepA ΔC/Δ4L	4.49E+03 ± 4.81E+02	3.11E+03 ± 3.26E+02	4.48E+03 ± 5.53E+02
	FepA ΔC/ΔL NSEGS	2.59E+03 ± 1.72E+02	1.87E+03 ± 1.55E+02	2.64E+03 ± 4.15E+02
	OmpF Δ	1.79E+03 ± 1.59E+02	1.33E+03 ± 1.46E+02	1.66E+03 ± 1.24E+02

Supplementary Table 8. **Analysis of the permeability of *E. coli* strains overproducing OMPs to HaloTag AF488 dye.** BL21 (DE3) strains overproducing the MBP:HaloTag fusion protein, and no protein (-) or the indicated protein were grown incubated with (color) or without (grey) 1 µM of HaloTag AF488, washed, resuspended in PBS and fluorescence was analyzed via flow cytometry. This experiment was performed with independent biological triplicates (n=3) with at least 5000 counted cells This experiment was performed with independent biological triplicates (n=3) with at least 80000 single events. The average and standard deviation of the fluorescence means, medians and modes are indicated.

		Fluorescence signal AF488 (488 nm, 530/30 nm)		
**Sample**	**OMP**	**Mean**	**Median**	**Mode**
**DMSO**	(-)	2.21E+02 ± 2.40E+01	1.80E+02 ± 2.22E+01	2.24E+02 ± 0.00E+00
	FhuA ΔC	1.73E+02 ± 4.04E+00	1.41E+02 ± 4.58E+00	2.24E+02 ± 0.00E+00
	FhuA ΔC/Δ5L	1.54E+02 ± 2.52E+00	1.23E+02 ± 1.73E+00	1.68E+02 ± 2.48E+01
	FepA ΔC/Δ4L	2.57E+02 ± 7.00E+01	1.83E+02 ± 4.04E+01	2.45E+02 ± 7.82E+01
	FepA ΔC/ΔL NSEGS	1.57E+02 ± 4.65E+01	1.17E+02 ± 1.76E+01	1.48E+02 ± 1.96E+01
	OmpF Δ	2.82E+02 ± 4.04E+00	2.33E+02 ± 2.65E+00	3.39E+02 ± 1.73E+01
**AF488 (488 nm, 530/30 nm)**	(-)	3.60E+02 ± 3.40E+01	2.92E+02 ± 2.97E+01	3.97E+02 ± 7.02E+01
	FhuA ΔC	3.57E+02 ± 8.08E+00	2.83E+02 ± 8.62E+00	4.18E+02 ± 3.70E+01
	FhuA ΔC/Δ5L	5.15E+02 ± 1.25E+02	3.98E+02 ± 1.03E+02	5.08E+02 ± 7.49E+01
	FepA ΔC/Δ4L	1.28E+03 ± 9.27E+01	7.27E+02 ± 1.05E+02	9.16E+02 ± 9.12E+01
	FepA ΔC/ΔL NSEGS	6.86E+02 ± 3.00E+01	5.26E+02 ± 1.81E+01	7.43E+02 ± 3.30E+01
	OmpF Δ	3.61E+02 ± 3.69E+01	2.84E+02 ± 1.46E+01	3.82E+02 ± 3.40E+01

Supplementary Table 9. **Microscopy analysis of the permeability of *E. coli* strains overproducing OMPs to HaloTag AF488 dye.** BL21 (DE3) strains overproducing the MBP:HaloTag fusion protein, and no protein (-) or the indicated protein were grown incubated with (color) or without (grey) 1 µM of HaloTag AF488, washed, resuspended in PBS and fluorescence was analyzed via microscopy. This experiment was performed with independent biological triplicates (n=3) with at least 5000 counted cells. The mean, integrated density and raw integrated density of the fluorescence signal of the triplicates are indicated.

OMP	HaloTag AF488-stained cells (%)	Mean	IntDen	Raw IntDen
(-)	3.20E+00 ± 1.47E+00	2.48E+01 ± 2.71E+00	3.80E+01 ± 1.12E+01	1.17E+03 ± 3.46E+02
FhuA ΔC	2.99E+00 ± 1.46E+00	2.25E+01 ± 1.59E+00	3.38E+01 ± 8.83E+00	1.04E+03 ± 2.72E+02
FhuA ΔC/Δ5L	9.55E+00 ± 2.13E+00	2.86E+01 ± 3.21E+00	4.85E+01 ± 1.08E+01	1.49E+03 ± 3.31E+02
FepA ΔC/Δ4L	1.64E+01 ± 4.90E+00	3.63E+01 ± 3.45E+00	1.40E+02 ± 3.68E+01	4.31E+03 ± 1.13E+03
FepA ΔC/ΔL NSEGS	2.05E+01 ± 4.94E+00	3.52.E+01 ± 3.68E+00	1.82E+02 ± 5.46E+01	5.59E+03 ± 1.68E+03
OmpF Δ	6.14E+00 ± 8.20E-01	2.66E+01 ± 1.22E+00	6.30E+01 ± 1.08E+01	1.94E+03 ± 3.33E+02

Supplementary Table 10. **List of *E. coli* strains used in this study.**

Strain	Genotype	Source
DH5α	F– φ80lacZΔ M15 Δ (lacZYA-argF) U169 recA1 endA1 hsdR17 (rK– mK+) phoA supE44 λ- thi–1 gyrA96 relA1	Thermo Fisher Scientific
NEB® Turbo	F' proA+B+ lacIq ΔlacZM15 / fhuA2 Δ(lac-proAB) glnV galK16 galE15 R(zgb-210::Tn10)TetS endA1 thi-1 Δ(hsdS-mcrB)5	New England Biolabs
BL21(DE3)	F– ompT hsdSB (rB–, mB–) gal dcm (DE3)	Merck
BL21(DE3) ΔmalE	F– ompT hsdSB (rB–, mB–) gal dcm (DE3) ΔmalE	This study
BL21 Gold(DE3)	B F– ompT hsdS(rB– mB–) dcm+ Tetr gal λ(DE3) endA Hte	Agilent Technologies
BL21 ΔABCF	B F– ompT hsdS(rB– mB–) dcm+ Tetr gal λ(DE3) endA Hte ΔompA ΔompC ΔompF ΔlamB	Meuskens et al. 2017

Supplementary Table 11. **List of primers used in this study.**

Name	DNA sequence (5’-3’)	Description
IC18NOV01	GCCTAGGCCGCGGCCGCGCGTAATACGACTCACTATAGGGGAATTGTGAGCGGATAACAATTCCCCTCTAGAAATAATTTTGTTTAACTTTAAGAAGGAGATATACATATGGCGCGTTCCAAAACTGC	Amplification of FhuA ΔC For and T7 addition
IC18NOV02	CCAGTCACGACGCGGCCGCACAAAAAACCCCTCAAGACCCG	Amplification of FhuA ΔC Rev
IC18NOV14	GCCTAGGCCGCGGCCGCGCGTAATACGACTCACTATAGGGGAATTGTGAGCGGATAACAATTCCCCTCTAGAAATAATTTTGTTTAACTTTAAGAAGGAGATATACATATGAACAAGAAGATTCATTCCCTG	Amplification of FepA For and T7 addition
IC18NOV15	CCAGTCACGACGCGGCCGCACAAAAAACCCCTCAAGACCCGTTTAGAGGCCCCAAGGGGTTATGCTAGTTATTGCTCAGCGGTCAGAAGTGGGTGTTTACGC	Amplification of FepA For
IC19JAN07	GCCGTTCAGCAGTAATAAGG	Sequencing of FhuA
IC19JAN08	ATCTGGCACGTAAATACGTC	Sequencing of FhuA
IC19JAN09	GCACGTGGCGTAGAAATCG	Sequencing of FhuA
IC19JAN10	GCCTAGGCCGCGGCCGCGCGTAATACGACTCACTATAGGGGAATTGTGAGCGGATAACAATTCCCCTCTAGAAATAATTTTGTTTAACTTTAAGAAGGAGATATACATATGGCGCGTTCCAAAACTG	Amplification of FhuA For and T7 addition
IC19JAN11	GAAGTCGAAATCGGTATTCACCGGATTGTACAGATTGAGCAGTG	Amplification of FhuA Rev
IC19MAR05	GCGGAAAAGGACCCTTGTC	Amplification of CamR cassette For
IC19MAR06	CCAATAATTACGATTTAAATGATCGG	Amplification of CamR cassette Rev
IC19APR07	GCCTAGGCCGCGGCCGCGCGTAATACGACTCACTATAGGGGAATTGTGAGCGGATAACAATTCCCCTCTAGAAATAATTTTGTTTAACTTTAAGAAGGAGATATACATATGGCTCGCTCCAAAACGG	Amplification of FhuA ΔC/Δ5L NSEGS and T7 addition For
IC19APR08	CCAGTCACGACGCGGCCGCACAAAAAACCCCTCAAGACCCGTTTAGAGGCCCCAAGGGGTTATGCTAGTTATTGCTCAGCGGTTAAAAACGAAAGGTTGCGGTG	Amplification of FhuA ΔC/Δ5L NSEGS Rev
IC19APR09	TAATACGACTCACTATAGGG	Sequencing of FhuA ΔC/Δ5L NSEGS
IC19APR10	TAGTTATTGCTCAGCGGTGG	Sequencing of FhuA ΔC/Δ5L NSEGS
IC19MAY01	TGCGGCCGCGTCGTGACTG	Amplification of pSEVA empty vectors For
IC19MAY02	CGCGCGGCCGCGGCCTAG	amplification of pSEVA empty vectors Rev
IC19SEPT03	TGATGCCTTTAATTAAAGGAATTCCGTCTCAGGAGTAATACGACTCACTATAGGGGAATTGTGAGCGGATAACAATTCCCCTCTAGAAATAATTTTGTTTAACTTTAAGAAGGAGATATACATATGCGTAAAGGCGAAGAG	Amplification of GFP and addition of T7 For

Name	DNA sequence (5'-3')	Description
IC19SEPT04	GTTTTCCCAGTCACGACAAGCTTCGTCTCATGACAGCGCAAAAAACCCCTCAAGACCCGTTTAGAGGCCCCAAGGGGTTATGCTAGTTATTGCTCAGCGGTCATTTGTACAGTTCATCCATAC	Amplification of GFP Rev
IC19OCT03	AGGCTGGCCGTAGGCCGGCCGGGCAGGATAGGTGAAGTAG	Amplification of pSEVA 36X plasmid For
IC19OCT04	CCTAGACAGCTGGGCGCGCCGGCGCGCCGGATATATTC	Amplification of pSEVA 36X plasmid Rev
IC20JAN02	TTAATTGGACCGCGGTCCGC	Amplification of KanR cassette For
IC20JAN03	CCTTGTATTACTGTTTATGTAAGCAGACAG	Amplification of KanR cassette Rev
IC20JAN04	ACATAAACAGTAATACAAGGTTCAAATATGTATCCGCTCATG	Amplification of AmpR cassette For
IC20JAN05	GCGGACCGCGGTCCAATTAATTACCAATGTTTAATCAGGCTC	Amplification of AmpR cassette Rev
IC20JUL01	TTTAAGAAGGAGATATACATATGAACAAGAAGATTCATTCCCTGGCCTTGTTGGTCAATCTGGGGATTTATGGGGTAGCGCAGGCAGCAGCTGCGCGTTATGGC	Amplification of FepA ΔC128 For
IC20JUL02	ATGTATATCTCCTTCTTAAAGTTAAACAAAATTATTTC	Amplification of FepA ΔC128 Rev
IC20JUL03	TTTAAGAAGGAGATATACATATGAACAAGAAGATTCATTCCCTGGCCTTGTTGGTCAATCTGGGGATTTATGGGGTAGCGCAGGCAGCCGCCGCTCGTTATGG	Amplification of FepA ΔC128/ΔL For
IC20OCT01	CTGACCACCTTTACCCCTAATC	Amplification of pRha promoter system For
IC20OCT02	CATCCGGCAATCAATGCCTG	Amplification of pRha promoter system Rev
IC21JUL03	TTTAAGAAGGAGATATACATATGATGAAGCGCAATATTCTGG	Amplification of OmpF For
IC21JUL04	TGCTAGTTATTGCTCAGCGGTTAGAACTGGTAAACGATACC	Amplification of OmpF Rev
IC21SEP01	ATGTATATCTCCTTCTTAAAGTTAAACAAAATTATTTCTAGAGG	Amplification pSEVA 29X backbone Rev
IC21SEP02	GTTTAACTTTAAGAAGGAGATATACATATG	Amplification of OmpF For
IC21SEP03	GCCGCCTAGGCCGCGGCCGCTCAAAAGTGAGTGTTGACGC	Amplification of FepA ΔC128/ΔL Rev
IC21SEP04	GCCGCCTAGGCCGCGGCCGCTTAAAAACGAAAGGTTGCGGTG	Amplification of FhuA ΔC/Δ5L Rev
IC21SEP05	GCCGCCTAGGCCGCGGCCGCTTAGAAACGGAAGGTTGCG	Amplification of FhuA ΔC rev
IC21SEP06	AATTCCTTTAATTAAAGGCATC	Amplification pSEVA 29X with pBAD Rev
IC21SEP07	CCTCTAGAAATAATTTTGTTTAAC	Amplification pSEVA 29X with pBAD For
IC20OCT01	CTGACCACCTTTACCCCTAATC	Amplification of pRha promoter system For
IC20OCT02	CATCCGGCAATCAATGCCTG	Amplification of pRha promoter system Rev

Name	DNA sequence (5’-3’)	Description
IC21SEP08	GATGCCTTTAATTAAAGGAATTTTATGACAACTTGACGGCTACAT C	Amplification of pBAD promoter system For
IC21SEP09	GTTAAACAAAATTATTTCTAGAGGCAGTAGAGAGTTGCGATAAA AAGC	Amplification of pBAD promoter system Rev
IC22MAR01	AGGGTACCTCTAGAAATAATTTTGTTTAACTTTAAGAAGGAGATA TACATCTCGAGCCAACCACTGAG	Amplification of HALO domain For
IC22MAR02	AAGACTAGTCGAGCGGATAACAATTTCACACAGGAGGCCGTTA GTGGTGATGGTGATGATG	Amplification of HALO domain Rev
IC22MAR03	CGGCCTCCTGTGTGAAATTG	Amplification of pSEVA 36X backbone for MBP:Halo expression For
IC22MAR04	ATGTATATCTCCTTCTTAAAGTTAAACAAAATTATTTCTAGAGG	Amplification of pSEVA 36X for MBP:Halo expression Rev
IC22MAR05	ATGTCCAGACCTGCAGGC	Amplification of pSEVA 36Xwith pBAD prom For
IC22MAR06	ATGTATATCTCCTTCTTAAAGTTAAACAAAATTATTTCTAG	Amplification of pSEVA 36Xwith pBAD prom Rev
IC22MAR07	CTAGAAATAATTTTGTTTAACTTTAAGAAGGAGATATACATATGGC GCGTTCCAAAACTG	Amplification of FhuA to clone it with pBAD promoter For
IC22MAR08	TTGATGCCTCTAGAGCTTGCATGCCTGCAGGTCTGGACATTTAGA AACGGAAGGTTGCGG	Amplification of FhuA to clone it with pBAD promoter Rev
IC22MAR09	CTAGAAATAATTTTGTTTAACTTTAAGAAGGAGATATACATATGGC TCGCTCCAAAACGG	Amplification of FhuA ΔC/Δ5L NSEGS to clone it with pBAD promoter For
IC22MAR10	TTGATGCCTCTAGAGCTTGCATGCCTGCAGGTCTGGACATTTAAA AACGAAAGGTTGCGGTG	Amplification of FhuA ΔC/Δ5L NSEGS to clone it with pBAD promoter Rev
IC22MAR11	CTAGAAATAATTTTGTTTAACTTTAAGAAGGAGATATACATATGAA CAAGAAGATTCATTCCCTG	Amplification of FepA to clone it with pBAD promoter For
IC22MAR12	TTGATGCCTCTAGAGCTTGCATGCCTGCAGGTCTGGACATTCAG AAGTGGGTGTTTACGC	Amplification of FepA to clone it with pBAD promoter For
IC22MAR13	TTGATGCCTCTAGAGCTTGCATGCCTGCAGGTCTGGACATTCAAA AGTGAGTGTTGACGC	Amplification of FepA ΔC128/ΔL to clone it with pBAD promoter Rev
IC22MAR18	CTAGAAATAATTTTGTTTAACTTTAAGAAGGAGATATACATATGCG TAAAGGCGAAGAGCT	Amplification of GFP to clone it with pBAD promoter For
IC22MAR19	TTGATGCCTCTAGAGCTTGCATGCCTGCAGGTCTGGACATTCATT TGTACAGTTCATCCATACCATG	Amplification of GFP to clone it with pBAD promoter Rev
IC22MAR20	CTAGAAATAATTTTGTTTAACTTTAAGAAGGAGATATACATATGAT GAAGCGCAATATTCT	Amplification of Omp to clone it with pBAD promoter For

Name	DNA sequence (5’-3’)	Description
IC22MAR21	TTGATGCCTCTAGAGCTTGCATGCCTGCAGGTCTGGACATTTAGAACTGGTAAACGATAC	Amplification of Omp to clone it with pBAD promoter Rev
IC22MAR22	CTAGAAATAATTTTGTTTAACTTTAAGAAGGAGATATACATATGAAAAAGAGCACTCTGGC	Amplification of Omp Δ to clone it with pBAD promoter For
IC22MAR23	TTGATGCCTCTAGAGCTTGCATGCCTGCAGGTCTGGACATGAACTGGTAAACGATACCC	Amplification of Omp Δ to clone it with pBAD promoter Rev
IC22JUN01	TCCTCAGTGGTTGGCTCGAGCTTGGTGATACGAGTCTGC	Amplification of pSEVA 36X with pRha For
IC22JUN02	CAATTTCACACAGGAGGCCGCTTGGTGATACGAGTCTGCG	Amplification of MBP TU for 3' fusion with Halo
IC22JUN03	CGCGTCTTATCAGGCCT	Amplification of 5' malE and its flanking regions for colony PCR For
IC22JUN04	GAGGTTGCCGTATAAAGAAAC	Amplification of 5' malE and its flanking regions for colony PCR For
IC22JUN05	AGTCCGTTTAGGTGTTTTCACGAGCACTTCACCAACAAGGACCATAGATTCATATGAATATCCTCCTTAGTTCCTATTC	Amplification of KanR casette from pKD4 with malE flanking regions Rev
IC22JUN06	GTTTTGTAGGCCGGACAAGGCGTTCACGCCGCATCCGGCATTTCACAGCAGTGTAGGCTGGAGCTGCTTC	Amplification of KanR casette from pKD4 with malE flanking regions For
IC23JUN10	TATACCGTTGTGGATGCACTGGTTC	Addition of L10 to FhuA ΔC/Δ5L to clone FhuA ΔC/Δ4L For
IC23JUN11	AGTGCATCCACAACGGTATAACTTCCCACTTTAAAGGAGTTAGCCGGATCACCATAACTGGAGCCGGTATAGCGACCACCGGTG	Addition of L10 to FhuA ΔC/Δ5L to clone FhuA ΔC/Δ4L Rev
IC23SEPT01	ATGAAAATAAAAACAGGTGCACG	Amplification of malE for colony PCR For
IC23SEPT02	TTACTTGGTGATACGAGTCTG	Amplification of malE for colony PCR Rev

Supplementary Table 11. **List of plasmids used in this study.**

Plasmid	Description	Reference
pT7-FhuA	pT7-FhuA cloned in pSEVA291 (pBR322-ROP, Kan^R)	This study
pT7-FhuA ΔC	pT7-FhuA ΔC cloned in pSEVA291 (pBR322-ROP, Kan^R)	This study
pT7-FhuA ΔC/Δ4L NSEGS	pT7-FhuA ΔC/Δ4L cloned in pSEVA291 (pBR322-ROP, Kan^R)	This study
pT7-FhuA ΔC/Δ5L NESGS	pT7-FhuA ΔC/Δ5L cloned in pSEVA291 (pBR322-ROP, Kan^R)	This study
pT7-FepA	pT7-FepA cloned in pSEVA291 (pBR322-ROP, Kan^R)	This study
pT7-FepA ΔC128	pT7-FepA ΔC128 cloned in pSEVA291 (pBR322-ROP, Kan^R)	This study
pT7-FepA ΔC128/ΔL	pT7-FepA ΔC128/ΔL cloned in pSEVA291 (pBR322-ROP, Kan^R)	This study
pT7-FepA ΔC	pT7-FepA ΔC cloned in pSEVA291 (pBR322-ROP, Kan^R)	This study
pT7-FepA ΔC/Δ4L	pT7-FepA ΔC/Δ4L cloned in pSEVA291 (pBR322-ROP, Kan^R)	This study
pT7-FepA ΔC/Δ4L NSEGS	pT7-FepA ΔC/Δ4L NSEGS cloned in pSEVA291 (pBR322-ROP, Kan^R)	This study
pT7-FepA ΔC/ΔL NSEGS	pT7-FepA ΔC/ΔL NSEGS cloned in pSEVA291 (pBR322-ROP, Kan^R)	This study
pT7-OmpF	pT7-OmpF cloned in pSEVA291 (pBR322-ROP, Kan^R)	This study
pT7-OmpF Δ	pT7-OmpF Δ cloned in pSEVA291 (pBR322-ROP, Kan^R)	This study
pT7-OmpF Δ GIGL	pT7-OmpF Δ GIGL cloned in pSEVA291 (pBR322-ROP, Kan^R)	This study
prhaBAD-FhuA ΔC	prhaBAD-FhuA ΔC cloned in pSEVA371 (pSC101, Cam^R)	This study
prhaBAD-FhuA ΔC/Δ5L NSEGS	prhaBAD-FhuA ΔC/Δ5L NSEGS cloned in pSEVA371 (pSC101, Cam^R)	This study
prhaBAD-FepA ΔC/Δ4L	prhaBAD-FepA ΔC/Δ4L cloned in pSEVA371 (pSC101, Cam^R)	This study
prhaBAD-FepA ΔC/ΔL NSEGS	prhaBAD-FepA ΔC/ΔL NSEGS cloned in pSEVA371 (pSC101, Cam^R)	This study
prhaBAD-OmpF Δ	prhaBAD-OmpF Δ cloned in pSEVA371 (pSC101, Cam^R)	This study
prhaBAD-OmpF Δ GIGL	prhaBAD-OmpF Δ GIGL cloned in pSEVA371 (pSC101, Cam^R)	This study
pSAV S112M K121R	pT7-OmpA-SAV S112M K121R cloned in pSEVA 291 (pBR322-ROP, Kan^R)	Vornholt et al. [70]
MBP-HaloTag	prhaBAD-MBP-HaloTagL cloned in pSEVA361 (p15A, Cam^R)	This study
pKD4	FRT-*kan*-FRT (R6K, Kan^R, $Carb^R$)	Datsenko and Wanner[82]
pKD46	araC-P_{BAD}-gam-beta-exo (pSC101 (ts), $Carb^R$)	Datsenko and Wanner[82]
pCP20	*flp* expression. (pSC101 (ts), Cam^R, $Carb^R$)	Datsenko and Wanner[82]

Supplementary Table 12. Effect of different expression induction levels on on outer membrane permeability to bulky hydrophobic molecules. MICs of strains overproducing the indicated OMP and controls when incubated with the indicated concentrations of rhamnose. (-) represents the MIC obtained for strain BL21(DE3) containing a plasmid without gene for overexpression. BL21 (DE3) strains overproducing no protein (-) or the indicated OMP were grown in LB supplemented with 50 mg·L-1 of kanamycin, 50 µM IPTG, the indicated concentrations of rhamnose, and varying concentrations of erythromycin ranging from 1 mg/mL to 0 mg/mL. After 18 h of incubation at 37°C, cell density was analyzed by measuring the OD600 of the samples. This experiment was performed with independent biological triplicates (n=3), and the value displayed here corresponds to the average of the three replicates.

**OMP**	**Erythromycin MIC (µg·mL-1)**				
BL21(DE3)(-)	125	125	125	125	125
FepA	125	125	125	125	125
FepA ΔC/Δ4L	125	15.6	15.6	15.6	15.6
FepA ΔC/ΔL NSEGS	125	15.6	15.6	15.6	15.6
	**0**	**0.1**	**0.2**	**0.5**	**1**
	**Rhamnose % (w/v)**				

Supplementary Data 1. DNA sequences of the plasmids used in this study:

pSEVA291

TTAATTAAAGCGGATAACAATTTCACACAGGAGGCCGCCTAGGCCGCGGCCGCGCGAATTCGAGCTCGGTACCCGGGGATCCTCTAGAGTCGACCTGCAGGCATGCAAGCTTGCGGCCGCGTC
GTGACTGGGAAAACCCTGGCGACTAGTCTTGGACTCCTGTTGATAGATCCAGTAATGACCTCAGAACTCCATCTGGATTTGTTCAGAACGCTCGGTTGCCGCCGGGCGTTTTTTATTGGTGAGA
ATCCAGGGGTCCCCAATAATTACGATTTAAATTTGTGTCTCAAAATCTCTGATGTTACATTGCACAAGATAAAAATATATCATCATGAACAATAAAACTGTCTGCTTACATAAACAGTAATACAAG
GGGTGTTATGAGCCATATTCAGCGTGAAACGAGCTGTAGCCGTCCGCGTCTGAACAGCAACATGGATGCGGATCTGTATGGCTATAAATGGGCGCGTGATAACGTGGGTCAGAGCGGCGCGA
CCATTTATCGTCTGTATGGCAAACCGGATGCGCCGGAACTGTTTCTGAAACATGGCAAAGGCAGCGTGGCGAACGATGTGACCGATGAAATGGTGCGTCTGAACTGGCTGACCGAATTTATGC
CGCTGCCGACCATTAAACATTTTATTCGCACCCCGGATGATGCGTGGCTGCTGACCACCGCGATTCCGGGCAAAACCGCGTTTCAGGTGCTGGAAGAATATCCGGATAGCGGCGAAAACATTG
TGGATGCGCTGGCCGTGTTTCTGCGTCGTCTGCATAGCATTCCGGTGTGCAACTGCCCGTTTAACAGCGATCGTGTGTTTCGTCTGGCCCAGGCGCAGAGCCGTATGAACAACGGCCTGGTGGA
TGCGAGCGATTTTGATGATGAACGTAACGGCTGGCCGGTGGAACAGGTGTGGAAAGAAATGCATAAACTGCTGCCGTTTAGCCCGGATAGCGTGGTGACCCACGGCGATTTTAGCCTGGATA
ACCTGATTTTCGATGAAGGCAAACTGATTGGCTGCATTGATGTGGGCCGTGTGGGCATTGCGGATCGTTATCAGGATCTGGCCATTCTGTGGAACTGCCTGGGCGAATTTAGCCCGAGCCTGC
AAAAACGTCTGTTTCAGAAATATGGCATTGATAATCCGGATATGAACAAACTGCAATTTCATCTGATGCTGGATGAATTTTTCTAATAATTAATTGGACCGCGGTCCGCGCGTTGTCCTTTTCCGC
TGCATAACCCTGCTTCGGGGTCATTATAGCGATTTTTTCGGTATATCCATCCTTTTTCGCACGATATACAGGATTTTGCCAAAGGGTTCGTGTAGACTTTCCTTGGTGTATCCAACGGCGTCAGCC
GGGCAGGATAGGTGAAGTAGGCCCACCCGCGAGCGGGTGTTCCTTCTTCACTGTCCCTTATTCGCACCTGGCGGTGCTCAACGGGAATCCTGCTCTGCGAGGCTGGCCGTAGGCCGGCCCCGT
AGAAAAGATCAAAGGATCTTCTTGAGATCCTTTTTTTCTGCGCGTAATCTGCTGCTTGCAAACAAAAAAACCACCGCTACCAGCGGTGGTTTGTTTGCCGGATCAAGAGCTACCAACTCTTTTTCC
GAAGGTAACTGGCTTCAGCAGAGCGCAGATACCAAATACTGTCCTTCTAGTGTAGCCGTAGTTAGGCCACCACTTCAAGAACTCTGTAGCACCGCCTACATACCTCGCTCTGCTAATCCTGTTAC
CAGTGGCTGCTGCCAGTGGCGATAAGTCGTGTCTTACCGGGTTGGACTCAAGACGATAGTTACCGGATAAGGCGCAGCGGTCGGGCTGAACGGGGGGTTCGTGCACACAGCCCAGCTTGGA
GCGAACGACCTACACCGAACTGAGATACCTACAGCGTGAGCTATGAGAAAGCGCCACGCTTCCCGAAGGGAGAAAGGCGGACAGGTATCCGGTAAGCGGCAGGGTCGGAACAGGAGAGCG
CACGAGGGAGCTTCCAGGGGGAAACGCCTGGTATCTTTATAGTCCTGTCGGGTTTCGCCACCTCTGACTTGAGCGTCGATTTTTGTGATGCTCGTCAGGGGGGCGGAGCCTATGGAAAAACGC
CAGCAACGCGGCCTTTTTACGGTTCCTGGCCTTTTGCTGGCCTTTTGCTCACATGTTCTTTCCTGCGTTATCCCCTGATTCTGTGGATAACCGTATTACCGCCTTTGAGTGAGCTGATACCGCTCGC
CGCAGCCGAACGACCGAGCGCAGCGAGTCAGTGAGCGAGGAAGCGGAAGAGCGCCTGATGCGGTATTTTCTCCTTACGCATCTGTGCGGTATTTCACACCGCATATGGTGCACTCTCAGTACA
ATCTGCTCTGATGCCGCATAGTTAAGCCAGTATACACTCCGCTATCGCTACGTGACTGGGTCATGGCTGCGCCCCGACACCCGCCAACACCCGCTGACGCGCCCTGACGGGCTTGTCTGCTCCC
GGCATCCGCTTACAGACAAGCTGTGACCGTCTCCGGGAGCTGCATGTGTCAGAGGTTTTCACCGTCATCACCGAAACGCGCGAGGCAGCTGCGGTAAAGCTCATCAGCGTGGTCGTGCAGCGA
TTCACAGATGTCTGCCTGTTCATCCGCGTCCAGCTCGTTGAGTTTCTCCAGAAGCGTTAATGTCTGGCTTCTGATAAAGCGGGCCATGTTAAGGGCGGTTTTTTCCTGTTTGGTCACTGATGCCTC
CGTGTAAGGGGGATTTCTGTTCATGGGGGTAATGATACCGATGAAACGAGAGAGGATGCTCACGATACGGGTTACTGATGATGAACATGCCCGGTTACTGGAACGTTGTGAGGGTAAACAAC
TGGCGGTATGGATGCGGCGGGGGCGCGCCCAGCTGTCTAGGGCGGCGGATTTGTCCTACTCAGGAGAGCGTTCACCGACAAACAACAGATAAAACGAAAGGCCCAGTCTTTCGACTGAGCCT
TTCGTTTTATTTGATGCCT

pT7-FhuA

TTAATTAAAGGAATTTAATACGACTCACTATAGGGGAATTGTGAGCGGATAACAATTCCCCTCTAGAAATAATTTTGTTTAACTTTAAGAAGGAGATATACATATGGCGCGTTCCAAAACTGCTC
AGCCAAAACACTCACTGCGTAAAATCGCAGTTGTAGTAGCCACAGCGGTTAGCGGCATGTCTGTTTATGCACAGGCAGCGGTTGAACCGAAAGAAGACACTATCACCGTTACCGCTGCACCTG
CGCCGCAAGAAAGCGCATGGGGGCCTGCTGCAACTATTGCGGCGCGACAGTCTGCTACCGGCACTAAAACCGATACGCCGATTCAAAAAGTGCCACAGTCTATTTCTGTTGTGACCGCCGAAG
AGATGGCGCTGCATCAGCCGAAGTCGGTAAAAGAAGCGCTTAGCTACACGCCGGGTGTCTCTGTTGGTACGCGTGGCGCATCCAACACCTATGACCACCTGATCATTCGCGGCTTTGCGGCAG
AAGGCCAAAGCCAGAATAACTATCTGAATGGCCTGAAGTTGCAGGGCAACTTCTATAACGATGCGGTCATTGACCCGTATATGCTGGAACGCGCTGAAATTATGCGTGGCCCGGTTTCCGTGCT
TTACGGTAAAAGCAGTCCTGGCGGCCTGTTGAATATGGTCAGCAAGCGTCCGACCACCGAACCGCTGAAAGAAGTTCAGTTTAAAGCCGGTACTGACAGCCTGTTCCAGACTGGTTTTGACTTT
AGCGATTCGTTGGATGATGACGGTGTTTACTCTTATCGCCTGACCGGTCTTGCGCGTTCTGCCAATGCCCAGCAGAAAGGGTCAGAAGAGCAGCGTTATGCTATTGCACCGGCGTTCACCTGGC
GTCCGGATGATAAAACCAATTTTACCTTCCTTTCTTACTTCCAGAACGAGCCGGAAACCGGTTATTACGGCTGGTTGCCGAAAGAGGGAACCGTTGAGCCGCTGCCGAACGGTAAGCGTCTGCC
GACAGACTTTAATGAAGGGGCGAAGAACAACACCTATTCTCGTAATGAGAAGATGGTCGGCTACAGCTTCGATCACGAATTTAACGACACCTTTACTGTGCGTCAGAACCTGCGCTTTGCTGAA
AACAAAACCTCGCAAAACAGCGTTTATGGTTACGGCGTCTGCTCCGATCCGGCGAATGCTTACAGCAAACAGTGTGCGGCATTAGCGCCAGCGGATAAAGGCCATTATCTGGCACGTAAATAC
GTCGTTGATGATGAGAAGCTGCAAAACTTCTCCGTTGATACCCAGTTGCAGAGCAAGTTTGCCACTGGCGATATCGACCACACCCTGCTGACCGGTGTCGACTTTATGCGTATGCGTAATGACA
TCAACGCCTGGTTTGGTTACGACGACTCTGTGCCACTGCTCAATCTGTACAATCCGGTGAATACCGATTTCGACTTCAATGCCAAAGATCCGGCAAACTCCGGCCCTTACCGCATTCTGAATAAA
CAGAAACAAACGGGCGTTTATGTTCAGGATCAGGCGCAGTGGGATAAAGTGCTGGTCACCCTAGGCGGTCGTTATGACTGGGCAGATCAAGAATCTCTTAACCGCGTTGCCGGGACGACCGA
TAAACGTGATGACAAACAGTTTACCTGGCGTGGTGGTGTTAACTACCTGTTTGATAATGGTGTAACACCTTACTTCAGCTATAGCGAATCGTTTGAACCTTCTTCGCAAGTTGGGAAGGATGGTA
ATATTTTCGCACCGTCTAAAGGTAAGCAGTATGAAGTCGGCGTGAAATATGTACCGGAAGATCGTCCGATTGTAGTTACTGGTGCCGTGTATAATCTCACTAAAACCAACAACCTGATGGCGGA
CCCTGAGGGTTCCTTCTTCTCGGTTGAAGGTGGCGAGATCCGCGCACGTGGCGTAGAAATCGAAGCGAAAGCGGCGCTGTCGGCGAGTGTTAACGTAGTCGGTTCTTATACTTACACCGATGC
GGAATACACCACCGATACTACCTATAAAGGCAATACGCCTGCACAGGTGCCAAAACACATGGCTTCGTTGTGGGCTGACTACACCTTCTTTGACGGTCCGCTTTCAGGTCTGACGCTGGGCACC
GGTGGTCGTTATACTGGCTCCAGTTATGGTGATCCGGCTAACTCCTTTAAAGTGGGAAGTTATACGGTCGTGGATGCGTTAGTACGTTATGATCTGGCGCGAGTCGGCATGGCTGGCTCCAACG
TGGCGCTGCATGTTAACAACCTGTTCGATCGTGAATACGTCGCCAGCTGCTTTAACACTTATGGCTGCTTCTGGGGCGCAGAACGTCAGGTCGTTGCAACCGCAACCTTCCGTTTCTAACCGCTG
AGCAATAACTAGCATAACCCCTTGGGGCCTCTAAACGGGTCTTGAGGGGTTTTTTGAGCTTGTCGTGACTGGGAAAACCCTGGCGACTAGTCTTGGACTCCTGTTGATAGATCCAGTAATGACC
TCAGAACTCCATCTGGATTTGTTCAGAACGCTCGGTTGCCGCCGGGCGTTTTTTATTGGTGAGAATCCAGGGGTCCCCAATAATTACGATTTAAATTTGTGTCTCAAAATCTCTGATGTTACATTG
CACAAGATAAAAATATATCATCATGAACAATAAAACTGTCTGCTTACATAAACAGTAATACAAGGGGTGTTATGAGCCATATTCAGCGTGAAACGAGCTGTAGCCGTCCGCGTCTGAACAGCAA
CATGGATGCGGATCTGTATGGCTATAAATGGGCGCGTGATAACGTGGGTCAGAGCGGCGCGACCATTTATCGTCTGTATGGCAAACCGGATGCGCCGGAACTGTTTCTGAAACATGGCAAAG
GCAGCGTGGCGAACGATGTGACCGATGAAATGGTGCGTCTGAACTGGCTGACCGAATTTATGCCGCTGCCGACCATTAAACATTTTATTCGCACCCCGGATGATGCGTGGCTGCTGACCACCG
CGATTCCGGGCAAAACCGCGTTTCAGGTGCTGGAAGAATATCCGGATAGCGGCGAAAACATTGTGGATGCGCTGGCCGTGTTTCTGCGTCGTCTGCATAGCATTCCGGTGTGCAACTGCCCGT
TTAACAGCGATCGTGTGTTTCGTCTGGCCCAGGCGCAGAGCCGTATGAACAACGGCCTGGTGGATGCGAGCGATTTTGATGATGAACGTAACGGCTGGCCGGTGGAACAGGTGTGGAAAGAA
ATGCATAAACTGCTGCCGTTTAGCCCGGATAGCGTGGTGACCCACGGCGATTTTAGCCTGGATAACCTGATTTTCGATGAAGGCAAACTGATTGGCTGCATTGATGTGGGCCGTGTGGGCATT
GCGGATCGTTATCAGGATCTGGCCATTCTGTGGAACTGCCTGGGCGAATTTAGCCCGAGCCTGCAAAAACGTCTGTTTCAGAAATATGGCATTGATAATCCGGATATGAACAAACTGCAATTTC
ATCTGATGCTGGATGAATTTTTCTAATAATTAATTGGACCGCGGTCCGCGCGTTGTCCTTTTCCGCTGCATAACCCTGCTTCGGGGTCATTATAGCGATTTTTTCGGTATATCCATCCTTTTTCGCA
CGATATACAGGATTTTGCCAAAGGGTTCGTGTAGACTTTCCTTGGTGTATCCAACGGCGTCAGCCGGGCAGGATAGGTGAAGTAGGCCCACCCGCGAGCGGGTGTTCCTTCTTCACTGTCCCTT
ATTCGCACCTGGCGGTGCTCAACGGGAATCCTGCTCTGCGAGGCTGGCCGTAGGCCGGCCCCGTAGAAAAGATCAAAGGATCTTCTTGAGATCCTTTTTTTCTGCGCGTAATCTGCTGCTTGCA
AACAAAAAAACCACCGCTACCAGCGGTGGTTTGTTTGCCGGATCAAGAGCTACCAACTCTTTTTCCGAAGGTAACTGGCTTCAGCAGAGCGCAGATACCAAATACTGTCCTTCTAGTGTAGCCG
TAGTTAGGCCACCACTTCAAGAACTCTGTAGCACCGCCTACATACCTCGCTCTGCTAATCCTGTTACCAGTGGCTGCTGCCAGTGGCGATAAGTCGTGTCTTACCGGGTTGGACTCAAGACGATA
GTTACCGGATAAGGCGCAGCGGTCGGGCTGAACGGGGGGTTCGTGCACACAGCCCAGCTTGGAGCGAACGACCTACACCGAACTGAGATACCTACAGCGTGAGCTATGAGAAAGCGCCACG
CTTCCCGAAGGGAGAAAGGCGGACAGGTATCCGGTAAGCGGCAGGGTCGGAACAGGAGAGCGCACGAGGGAGCTTCCAGGGGGAAACGCCTGGTATCTTTATAGTCCTGTCGGGTTTCGCC
ACCTCTGACTTGAGCGTCGATTTTTGTGATGCTCGTCAGGGGGGCGGAGCCTATGGAAAAACGCCAGCAACGCGGCCTTTTTACGGTTCCTGGCCTTTTGCTGGCCTTTTGCTCACATGTTCTTT
CCTGCGTTATCCCCTGATTCTGTGGATAACCGTATTACCGCCTTTGAGTGAGCTGATACCGCTCGCCGCAGCCGAACGACCGAGCGCAGCGAGTCAGTGAGCGAGGAAGCGGAAGAGCGCCTG
ATGCGGTATTTTCTCCTTACGCATCTGTGCGGTATTTCACACCGCATATGGTGCACTCTCAGTACAATCTGCTCTGATGCCGCATAGTTAAGCCAGTATACACTCCGCTATCGCTACGTGACTGGG
TCATGGCTGCGCCCCGACACCCGCCAACACCCGCTGACGCGCCCTGACGGGCTTGTCTGCTCCCGGCATCCGCTTACAGACAAGCTGTGACCGTCTCCGGGAGCTGCATGTGTCAGAGGTTTTC
ACCGTCATCACCGAAACGCGCGAGGCAGCTGCGGTAAAGCTCATCAGCGTGGTCGTGCAGCGATTCACAGATGTCTGCCTGTTCATCCGCGTCCAGCTCGTTGAGTTTCTCCAGAAGCGTTAAT
GTCTGGCTTCTGATAAAGCGGGCCATGTTAAGGGCGGTTTTTTCCTGTTTGGTCACTGATGCCTCCGTGTAAGGGGGATTTCTGTTCATGGGGGTAATGATACCGATGAAACGAGAGAGGATG
CTCACGATACGGGTTACTGATGATGAACATGCCCGGTTACTGGAACGTTGTGAGGGTAAACAACTGGCGGTATGGATGCGGCGGGGGCGCGCCCAGCTGTCTAGGGCGGCGGATTTGTCCTA
CTCAGGAGAGCGTTCACCGACAAACAACAGATAAAACGAAAGGCCCAGTCTTTCGACTGAGCCTTTCGTTTTATTTGATGCCT

pT7-FhuA ΔC

AATTCCGTCTCAGGAGTAATACGACTCACTATAGGGGAATTGTGAGCGGATAACAATTCCCCTCTAGAAATAATTTTGTTTAACTTTAAGAAGGAGATATACATATGGCGCGTTCCAAAACTGCT
CAGCCAAAACACTCACTGCGTAAAATCGCAGTTGTAGTAGCCACAGCGGTTAGCGGCATGTCTGTTTATGCACAGGCACTGAAAGAAGTTCAGTTTAAAGCCGGTACTGACAGCCTGTTCCAGA
CTGGTTTTGACTTTAGCGATTCGTTGGATGATGACGGTGTTTACTCTTATCGCCTGACCGGTCTTGCGCGTTCTGCCAATGCCCAGCAGAAAGGGTCAGAAGAGCAGCGTTATGCTATTGCACC
GGCGTTCACCTGGCGTCCGGATGATAAAACCAATTTTACCTTCCTTTCTTACTTCCAGAACGAGCCGGAAACCGGTTATTACGGCTGGTTGCCGAAAGAGGGAACCGTTGAGCCGCTGCCGAAC
GGTAAGCGTCTGCCGACAGACTTTAATGAAGGGGCGAAGAACAACACCTATTCTCGTAATGAGAAGATGGTCGGCTACAGCTTCGATCACGAATTTAACGACACCTTTACTGTGCGTCAGAAC
CTGCGCTTTGCTGAAAACAAAACCTCGCAAAACAGCGTTTATGGTTACGGCGTCTGCTCCGATCCGGCGAATGCTTACAGCAAACAGTGTGCGGCATTAGCGCCAGCGGATAAAGGCCATTATC
TGGCACGTAAATACGTCGTTGATGATGAGAAGCTGCAAAACTTCTCCGTTGATACCCAGTTGCAGAGCAAGTTTGCCACTGGCGATATCGACCACACCCTGCTGACCGGTGTCGACTTTATGCG
TATGCGTAATGACATCAACGCCTGGTTTGGTTACGACGACTCTGTGCCACTGCTCAATCTGTACAATCCGGTGAATACCGATTTCGACTTCAATGCCAAAGATCCGGCAAACTCCGGCCCTTACC
GCATTCTGAATAAACAGAAACAAACGGGCGTTTATGTTCAGGATCAGGCGCAGTGGGATAAAGTGCTGGTCACCCTAGGCGGTCGTTATGACTGGGCAGATCAAGAATCTCTTAACCGCGTTG
CCGGGACGACCGATAAACGTGATGACAAACAGTTTACCTGGCGTGGTGGTGTTAACTACCTGTTTGATAATGGTGTAACACCTTACTTCAGCTATAGCGAATCGTTTTTCCCTTCTTCGCAAGTT
GGGAAGGATGGTAATATTTTCGCACCGTCTAAAGGTAAGCAGTATGAAGTCGGCGTGAAATATGTACCGGAAGATCGTCCGATTGTAGTTACTGGTGCCGTGTATAATCTCACTAAAACCAAC
AACCTGATGGCGGACCCTGAGGGTTCCTTCTTCTCGGTTGAAGGTGGCGAGATCCGCGCACGTGGCGTAGAAATCGAAGCGAAAGCGGCGCTGTCGGCGAGTGTTAACGTAGTCGGTTCTTA
TACTTACACCGATGCGGAATACACCACCGATACTACCTATAAAGGCAATACGCCTGCACAGGTGCCAAAACACATGGCTTCGTTGTGGGCTGACTACACCTTCTTTGACGGTCCGCTTTCAGGTC
TGACGCTGGGCACCGGTGGTCGTTATACTGGCTCCAGTTATGGTGATCCGGCTAACTCCTTTAAAGTGGGAAGTTATACGGTCGTGGATGCGTTAGTACGTTATGATCTGGCGCGAGTCGGCAT
GGCTGGCTCCAACGTGGCGCTGCATGTTAACAACCTGTTCGATCGTGAATACGTCGCCAGCTGCTTTAACACTTATGGCTGCTTCTGGGGCGCAGAACGTCAGGTCGTTGCAACCGCAACCTTC
CGTTTCTAACCGCTGAGCAATAACTAGCATAACCCCTTGGGGCCTCTAAACGGGTCTTGAGGGGTTTTTTGCGCTGTCATGAGACGAAGCTTGTCGTGACTGGGAAAACCCTGGCGACTAGTCT
TGGACTCCTGTTGATAGATCCAGTAATGACCTCAGAACTCCATCTGGATTTGTTCAGAACGCTCGGTTGCCGCCGGGCGTTTTTTATTGGTGAGAATCCAGGGGTCCCCAATAATTACGATTTAA
ATTTGTGTCTCAAAATCTCTGATGTTACATTGCACAAGATAAAAATATATCATCATGAACAATAAAACTGTCTGCTTACATAAACAGTAATACAAGGGGTGTTATGAGCCATATTCAGCGTGAAA
CGAGCTGTAGCCGTCCGCGTCTGAACAGCAACATGGATGCGGATCTGTATGGCTATAAATGGGCGCGTGATAACGTGGGTCAGAGCGGCGCGACCATTTATCGTCTGTATGGCAAACCGGAT
GCGCCGGAACTGTTTCTGAAACATGGCAAAGGCAGCGTGGCGAACGATGTGACCGATGAAATGGTGCGTCTGAACTGGCTGACCGAATTTATGCCGCTGCCGACCATTAAACATTTTATTCGC
ACCCCGGATGATGCGTGGCTGCTGACCACCGCGATTCCGGGCAAAACCGCGTTTCAGGTGCTGGAAGAATATCCGGATAGCGGCGAAAACATTGTGGATGCGCTGGCCGTGTTTCTGCGTCGT
CTGCATAGCATTCCGGTGTGCAACTGCCCGTTTAACAGCGATCGTGTGTTTCGTCTGGCCCAGGCGCAGAGCCGTATGAACAACGGCCTGGTGGATGCGAGCGATTTTGATGATGAACGTAAC
GGCTGGCCGGTGGAACAGGTGTGGAAAGAAATGCATAAACTGCTGCCGTTTAGCCCGGATAGCGTGGTGACCCACGGCGATTTTAGCCTGGATAACCTGATTTTCGATGAAGGCAAACTGATT
GGCTGCATTGATGTGGGCCGTGTGGGCATTGCGGATCGTTATCAGGATCTGGCCATTCTGTGGAACTGCCTGGGCGAATTTAGCCCGAGCCTGCAAAAACGTCTGTTTCAGAAATATGGCATT
GATAATCCGGATATGAACAAACTGCAATTTCATCTGATGCTGGATGAATTTTTCTAATAATTAATTGGACCGCGGTCCGCGCGTTGTCCTTTTCCGCTGCATAACCCTGCTTCGGGGTCATTATAG
CGATTTTTTCGGTATATCCATCCTTTTTCGCACGATATACAGGATTTTGCCAAAGGGTTCGTGTAGACTTTCCTTGGTGTATCCAACGGCGTCAGCCGGGCAGGATAGGTGAAGTAGGCCCACCC
GCGAGCGGGTGTTCCTTCTTCACTGTCCCTTATTCGCACCTGGCGGTGCTCAACGGGAATCCTGCTCTGCGAGGCTGGCCGTAGGCCGGCCCCGTAGAAAAGATCAAAGGATCTTCTTGAGATC
CTTTTTTTCTGCGCGTAATCTGCTGCTTGCAAACAAAAAAACCACCGCTACCAGCGGTGGTTTGTTTGCCGGATCAAGAGCTACCAACTCTTTTTCCGAAGGTAACTGGCTTCAGCAGAGCGCAG
ATACCAAATACTGTCCTTCTAGTGTAGCCGTAGTTAGGCCACCACTTCAAGAACTCTGTAGCACCGCCTACATACCTCGCTCTGCTAATCCTGTTACCAGTGGCTGCTGCCAGTGGCGATAAGTC
GTGTCTTACCGGGTTGGACTCAAGACGATAGTTACCGGATAAGGCGCAGCGGTCGGGCTGAACGGGGGGTTCGTGCACACAGCCCAGCTTGGAGCGAACGACCTACACCGAACTGAGATACC
TACAGCGTGAGCTATGAGAAAGCGCCACGCTTCCCGAAGGGAGAAAGGCGGACAGGTATCCGGTAAGCGGCAGGGTCGGAACAGGAGAGCGCACGAGGGAGCTTCCAGGGGGAAACGCCT
GGTATCTTTATAGTCCTGTCGGGTTTCGCCACCTCTGACTTGAGCGTCGATTTTTGTGATGCTCGTCAGGGGGGCGGAGCCTATGGAAAAACGCCAGCAACGCGGCCTTTTTACGGTTCCTGGC
CTTTTGCTGGCCTTTTGCTCACATGTTCTTTCCTGCGTTATCCCCTGATTCTGTGGATAACCGTATTACCGCCTTTGAGTGAGCTGATACCGCTCGCCGCAGCCGAACGACCGAGCGCAGCGAGTC
AGTGAGCGAGGAAGCGGAAGAGCGCCTGATGCGGTATTTTCTCCTTACGCATCTGTGCGGTATTTCACACCGCATATGGTGCACTCTCAGTACAATCTGCTCTGATGCCGCATAGTTAAGCCAG
TATACACTCCGCTATCGCTACGTGACTGGGTCATGGCTGCGCCCCGACACCCGCCAACACCCGCTGACGCGCCCTGACGGGCTTGTCTGCTCCCGGCATCCGCTTACAGACAAGCTGTGACCGT
CTCCGGGAGCTGCATGTGTCAGAGGTTTTCACCGTCATCACCGAAACGCGCGAGGCAGCTGCGGTAAAGCTCATCAGCGTGGTCGTGCAGCGATTCACAGATGTCTGCCTGTTCATCCGCGTCC
AGCTCGTTGAGTTTCTCCAGAAGCGTTAATGTCTGGCTTCTGATAAAGCGGGCCATGTTAAGGGCGGTTTTTTCCTGTTTGGTCACTGATGCCTCCGTGTAAGGGGGATTTCTGTTCATGGGGGT
AATGATACCGATGAAACGAGAGAGGATGCTCACGATACGGGTTACTGATGATGAACATGCCCGGTTACTGGAACGTTGTGAGGGTAAACAACTGGCGGTATGGATGCGGCGGGGGCGCGCC
CAGCTGTCTAGGGCGGCGGATTTGTCCTACTCAGGAGAGCGTTCACCGACAAACAACAGATAAAACGAAAGGCCCAGTCTTTCGACTGAGCCTTTCGTTTTATTTGATGCCTTTAATTAAAGG

pT7-FhuA ΔC/Δ4L NSEGS

TTAATTAAAGCGGATAACAATTTCACACAGGAGGCCGCCTAGGCCGCGGCCGCGCGTAATACGACTCACTATAGGGGAATTGTGAGCGGATAACAATTCCCCTCTAGAAATAATTTTGTTTAAC
TTTAAGAAGGAGATATACATATGGCTCGCTCCAAAACGGCACAGCCTAAACATAGCCTGCGTAAAATTGCAGTTGTTGTTGCAACCGCAGTTAGCGGTATGAGCGTTTATGCACAGGCACTGAA
AGAAGTTCAGTTTAAAGCAGGCACCGATAGCCTGTTTCAGACCGGTTTTGATTTTAGCGATAGCCTGGATGATGATGGTGTTTATAGCTATCGTCTGACAGGTCTGGCTCGGAGTGCAAATGCA
CAGCAGAAAGGTAGCGAAGAACAGCGTTATGCAATTGCACCGGCATTTACCTGGCGTCCGGATGATAAAACCAATTTTACCTTTCTGAGCTATTTTCAGAATGAACCGGAAACCGGTAATAGCG
AAGGTAGCACCTATAGCCGTAATGAAAAAATGGTGGGCTATAGCTTTGATCATGAGTTTAATGATACCTTTACCGTTCGTCAGAATCTGCGTTTTGCCGAAAATAAAACCAGCCAGAATAGCGT
TTATGGTAATTCTGAAGGCAGCCGTAAATATGTTGTGGATGATGAAAAACTGCAGAATTTTAGCGTTGATACCCAGCTGCAGAGCAAATTTGCAACCGGTGATATTGATCATACCCTGCTGACC
GGTGTGGATTTTATGCGTATGCGCAATGATATTAATGCCTGGTTTGGCTATAATTCAGAAGGTAGCAGCGGTCCGTATCGTATTCTGAATAAACAGAAACAGACCGGTGTTTATGTTCAGGATC
AGGCACAGTGGGATAAAGTTCTGGTTACCCTGGGTGGTCGTTATGATTGGGCAGATCAGGAAAGCCTGAATCGCGTTGCAGGCACCACCGATAAACGTGATGATAAACAGTTTACATGGCGT
GGTGGTGTGAATTACCTGTTTGATAATGGCGTGACCCCGTATTTTAGCTATAGCGAAAGCTTTGAACCGAGCAGCCAGGTGGGTAAAGATGGCAATATTTTTGCACCGAGCAAAGGCAAACAG
TATGAAGTGGGCGTTAAATATGTTCCGGAAGATCGTCCGATTGTGGTTACCGGTGCCGTTTATAATCTGACCAAAACCAATAATCTGATGGCAGATCCGGAAGGTAGCTTTTTTAGCGTGGAAG
GTGGTGAAATTCGTGCACGTGGTGTTGAAATTGAAGCAAAAGCAGCACTGAGCGCAAGCGTTAATGTTGTTGGCAGCTATACCTATACCGATGCAGAATATACCACCGATACCACCTATAAAG
GTAATACACCGGCACAGGTTCCGAAACATATGGCAAGCCTGTGGGCAGATTATACCTTTTTTGATGGTCCGCTGTCTGGTCTGACCCTGGGCACCGGTGGTCGCTATACCGGCTCCAGTTATGG
TGATCCGGCTAACTCCTTTAAAGTGGGAAGTTATACCGTTGTGGATGCACTGGTTCGTTATGATCTGGCTCGTGTTGGTATGGCAGGTAGCAATGTTGCACTGCATGTGAATAGTGAAGGTAGC
CAGGTTGTTGCCACCGCAACCTTTCGTTTTTAACCGCTGAGCAATAACTAGCATAACCCCTTGGGGCCTCTAAACGGGTCTTGAGGGGTTTTTTGTGCGGCCGCGTCGTGACTGGGAAAACCCT
GGCGACTAGTCTTGGACTCCTGTTGATAGATCCAGTAATGACCTCAGAACTCCATCTGGATTTGTTCAGAACGCTCGGTTGCCGCCGGGCGTTTTTTATTGGTGAGAATCCAGGGGTCCCCAATA
ATTACGATTTAAATTTGTGTCTCAAAATCTCTGATGTTACATTGCACAAGATAAAAATATATCATCATGAACAATAAAACTGTCTGCTTACATAAACAGTAATACAAGGGGTGTTATGAGCCATAT
TCAGCGTGAAACGAGCTGTAGCCGTCCGCGTCTGAACAGCAACATGGATGCGGATCTGTATGGCTATAAATGGGCGCGTGATAACGTGGGTCAGAGCGGCGCGACCATTTATCGTCTGTATG
GCAAACCGGATGCGCCGGAACTGTTTCTGAAACATGGCAAAGGCAGCGTGGCGAACGATGTGACCGATGAAATGGTGCGTCTGAACTGGCTGACCGAATTTATGCCGCTGCCGACCATTAAA
CATTTTATTCGCACCCCGGATGATGCGTGGCTGCTGACCACCGCGATTCCGGGCAAAACCGCGTTTCAGGTGCTGGAAGAATATCCGGATAGCGGCGAAAACATTGTGGATGCGCTGGCCGTG
TTTCTGCGTCGTCTGCATAGCATTCCGGTGTGCAACTGCCCGTTTAACAGCGATCGTGTGTTTCGTCTGGCCCAGGCGCAGAGCCGTATGAACAACGGCCTGGTGGATGCGAGCGATTTTGATG
ATGAACGTAACGGCTGGCCGGTGGAACAGGTGTGGAAAGAAATGCATAAACTGCTGCCGTTTAGCCCGGATAGCGTGGTGACCCACGGCGATTTTAGCCTGGATAACCTGATTTTCGATGAA
GGCAAACTGATTGGCTGCATTGATGTGGGCCGTGTGGGCATTGCGGATCGTTATCAGGATCTGGCCATTCTGTGGAACTGCCTGGGCGAATTTAGCCCGAGCCTGCAAAAACGTCTGTTTCAG
AAATATGGCATTGATAATCCGGATATGAACAAACTGCAATTTCATCTGATGCTGGATGAATTTTTCTAATAATTAATTGGACCGCGGTCCGCGCGTTGTCCTTTTCCGCTGCATAACCCTGCTTCG
GGGTCATTATAGCGATTTTTTCGGTATATCCATCCTTTTTCGCACGATATACAGGATTTTGCCAAAGGGTTCGTGTAGACTTTCCTTGGTGTATCCAACGGCGTCAGCCGGGCAGGATAGGTGAA
GTAGGCCCACCCGCGAGCGGGTGTTCCTTCTTCACTGTCCCTTATTCGCACCTGGCGGTGCTCAACGGGAATCCTGCTCTGCGAGGCTGGCCGTAGGCCGGCCCCGTAGAAAAGATCAAAGGA
TCTTCTTGAGATCCTTTTTTTCTGCGCGTAATCTGCTGCTTGCAAACAAAAAAACCACCGCTACCAGCGGTGGTTTGTTTGCCGGATCAAGAGCTACCAACTCTTTTTCCGAAGGTAACTGGCTTC
AGCAGAGCGCAGATACCAAATACTGTCCTTCTAGTGTAGCCGTAGTTAGGCCACCACTTCAAGAACTCTGTAGCACCGCCTACATACCTCGCTCTGCTAATCCTGTTACCAGTGGCTGCTGCCAG
TGGCGATAAGTCGTGTCTTACCGGGTTGGACTCAAGACGATAGTTACCGGATAAGGCGCAGCGGTCGGGCTGAACGGGGGGTTCGTGCACACAGCCCAGCTTGGAGCGAACGACCTACACCG
AACTGAGATACCTACAGCGTGAGCTATGAGAAAGCGCCACGCTTCCCGAAGGGAGAAAGGCGGACAGGTATCCGGTAAGCGGCAGGGTCGGAACAGGAGAGCGCACGAGGGAGCTTCCAG
GGGGAAACGCCTGGTATCTTTATAGTCCTGTCGGGTTTCGCCACCTCTGACTTGAGCGTCGATTTTTGTGATGCTCGTCAGGGGGGCGGAGCCTATGGAAAAACGCCAGCAACGCGGCCTTTTT
ACGGTTCCTGGCCTTTTGCTGGCCTTTTGCTCACATGTTCTTTCCTGCGTTATCCCCTGATTCTGTGGATAACCGTATTACCGCCTTTGAGTGAGCTGATACCGCTCGCCGCAGCCGAACGACCGA
GCGCAGCGAGTCAGTGAGCGAGGAAGCGGAAGAGCGCCTGATGCGGTATTTTCTCCTTACGCATCTGTGCGGTATTTCACACCGCATATGGTGCACTCTCAGTACAATCTGCTCTGATGCCGCA
TAGTTAAGCCAGTATACACTCCGCTATCGCTACGTGACTGGGTCATGGCTGCGCCCCGACACCCGCCAACACCCGCTGACGCGCCCTGACGGGCTTGTCTGCTCCCGGCATCCGCTTACAGACA
AGCTGTGACCGTCTCCGGGAGCTGCATGTGTCAGAGGTTTTCACCGTCATCACCGAAACGCGCGAGGCAGCTGCGGTAAAGCTCATCAGCGTGGTCGTGCAGCGATTCACAGATGTCTGCCTG
TTCATCCGCGTCCAGCTCGTTGAGTTTCTCCAGAAGCGTTAATGTCTGGCTTCTGATAAAGCGGGCCATGTTAAGGGCGGTTTTTTCCTGTTTGGTCACTGATGCCTCCGTGTAAGGGGGATTTC
TGTTCATGGGGGTAATGATACCGATGAAACGAGAGAGGATGCTCACGATACGGGTTACTGATGATGAACATGCCCGGTTACTGGAACGTTGTGAGGGTAAACAACTGGCGGTATGGATGCGG
CGGGGGCGCGCCCAGCTGTCTAGGGCGGCGGATTTGTCCTACTCAGGAGAGCGTTCACCGACAAACAACAGATAAAACGAAAGGCCCAGTCTTTCGACTGAGCCTTTCGTTTTATTTGATGCCT

pT7-FhuA ΔC/Δ5L NSEGS

TTAATTAAAGCGGATAACAATTTCACACAGGAGGCCGCCTAGGCCGCGGCCGCGCGTAATACGACTCACTATAGGGGAATTGTGAGCGGATAACAATTCCCCTCTAGAAATAATTTTGTTTAAC
TTTAAGAAGGAGATATACATATGGCTCGCTCCAAAACGGCACAGCCTAAACATAGCCTGCGTAAAATTGCAGTTGTTGTTGCAACCGCAGTTAGCGGTATGAGCGTTTATGCACAGGCACTGAA
AGAAGTTCAGTTTAAAGCAGGCACCGATAGCCTGTTTCAGACCGGTTTTGATTTTAGCGATAGCCTGGATGATGATGGTGTTTATAGCTATCGTCTGACAGGTCTGGCTCGGAGTGCAAATGCA
CAGCAGAAAGGTAGCGAAGAACAGCGTTATGCAATTGCACCGGCATTTACCTGGCGTCCGGATGATAAAACCAATTTTACCTTTCTGAGCTATTTTCAGAATGAACCGGAAACCGGTAATAGCG
AAGGTAGCACCTATAGCCGTAATGAAAAAATGGTGGGCTATAGCTTTGATCATGAGTTTAATGATACCTTTACCGTTCGTCAGAATCTGCGTTTTGCCGAAAATAAAACCAGCCAGAATAGCGT
TTATGGTAATTCTGAAGGCAGCCGTAAATATGTTGTGGATGATGAAAAACTGCAGAATTTTAGCGTTGATACCCAGCTGCAGAGCAAATTTGCAACCGGTGATATTGATCATACCCTGCTGACC
GGTGTGGATTTTATGCGTATGCGCAATGATATTAATGCCTGGTTTGGCTATAATTCAGAAGGTAGCAGCGGTCCGTATCGTATTCTGAATAAACAGAAACAGACCGGTGTTTATGTTCAGGATC
AGGCACAGTGGGATAAAGTTCTGGTTACCCTGGGTGGTCGTTATGATTGGGCAGATCAGGAAAGCCTGAATCGCGTTGCAGGCACCACCGATAAACGTGATGATAAACAGTTTACATGGCGT
GGTGGTGTGAATTACCTGTTTGATAATGGCGTGACCCCGTATTTTAGCTATAGCGAAAGCTTTGAACCGAGCAGCCAGGTGGGTAAAGATGGCAATATTTTTGCACCGAGCAAAGGCAAACAG
TATGAAGTGGGCGTTAAATATGTTCCGGAAGATCGTCCGATTGTGGTTACCGGTGCCGTTTATAATCTGACCAAAACCAATAATCTGATGGCAGATCCGGAAGGTAGCTTTTTTAGCGTGGAAG
GTGGTGAAATTCGTGCACGTGGTGTTGAAATTGAAGCAAAAGCAGCACTGAGCGCAAGCGTTAATGTTGTTGGCAGCTATACCTATACCGATGCAGAATATACCACCGATACCACCTATAAAG
GTAATACACCGGCACAGGTTCCGAAACATATGGCAAGCCTGTGGGCAGATTATACCTTTTTTGATGGTCCGCTGTCTGGTCTGACCCTGGGCACCGGTGGTCGCTATACCAATTCTGAAGGTAG
CTATACCGTTGTGGATGCACTGGTTCGTTATGATCTGGCTCGTGTTGGTATGGCAGGTAGCAATGTTGCACTGCATGTGAATAGTGAAGGTAGCCAGGTTGTTGCCACCGCAACCTTTCGTTTTT
AACCGCTGAGCAATAACTAGCATAACCCCTTGGGGCCTCTAAACGGGTCTTGAGGGGTTTTTTGTGCGGCCGCGTCGTGACTGGGAAAACCCTGGCGACTAGTCTTGGACTCCTGTTGATAGAT
CCAGTAATGACCTCAGAACTCCATCTGGATTTGTTCAGAACGCTCGGTTGCCGCCGGGCGTTTTTTATTGGTGAGAATCCAGGGGTCCCCAATAATTACGATTTAAATTTGTGTCTCAAAATCTCT
GATGTTACATTGCACAAGATAAAAATATATCATCATGAACAATAAAACTGTCTGCTTACATAAACAGTAATACAAGGGGTGTTATGAGCCATATTCAGCGTGAAACGAGCTGTAGCCGTCCGCG
TCTGAACAGCAACATGGATGCGGATCTGTATGGCTATAAATGGGCGCGTGATAACGTGGGTCAGAGCGGCGCGACCATTTATCGTCTGTATGGCAAACCGGATGCGCCGGAACTGTTTCTGAA
ACATGGCAAAGGCAGCGTGGCGAACGATGTGACCGATGAAATGGTGCGTCTGAACTGGCTGACCGAATTTATGCCGCTGCCGACCATTAAACATTTTATTCGCACCCCGGATGATGCGTGGCT
GCTGACCACCGCGATTCCGGGCAAAACCGCGTTTCAGGTGCTGGAAGAATATCCGGATAGCGGCGAAAACATTGTGGATGCGCTGGCCGTGTTTCTGCGTCGTCTGCATAGCATTCCGGTGTG
CAACTGCCCGTTTAACAGCGATCGTGTGTTTCGTCTGGCCCAGGCGCAGAGCCGTATGAACAACGGCCTGGTGGATGCGAGCGATTTTGATGATGAACGTAACGGCTGGCCGGTGGAACAGG
TGTGGAAAGAAATGCATAAACTGCTGCCGTTTAGCCCGGATAGCGTGGTGACCCACGGCGATTTTAGCCTGGATAACCTGATTTTCGATGAAGGCAAACTGATTGGCTGCATTGATGTGGGCC
GTGTGGGCATTGCGGATCGTTATCAGGATCTGGCCATTCTGTGGAACTGCCTGGGCGAATTTAGCCCGAGCCTGCAAAAACGTCTGTTTCAGAAATATGGCATTGATAATCCGGATATGAACAA
ACTGCAATTTCATCTGATGCTGGATGAATTTTTCTAATAATTAATTGGACCGCGGTCCGCGCGTTGTCCTTTTCCGCTGCATAACCCTGCTTCGGGGTCATTATAGCGATTTTTTCGGTATATCCAT
CCTTTTTCGCACGATATACAGGATTTTGCCAAAGGGTTCGTGTAGACTTTCCTTGGTGTATCCAACGGCGTCAGCCGGGCAGGATAGGTGAAGTAGGCCCACCCGCGAGCGGGTGTTCCTTCTT
CACTGTCCCTTATTCGCACCTGGCGGTGCTCAACGGGAATCCTGCTCTGCGAGGCTGGCCGTAGGCCGGCCCCGTAGAAAAGATCAAAGGATCTTCTTGAGATCCTTTTTTTCTGCGCGTAATCT
GCTGCTTGCAAACAAAAAAACCACCGCTACCAGCGGTGGTTTGTTTGCCGGATCAAGAGCTACCAACTCTTTTTCCGAAGGTAACTGGCTTCAGCAGAGCGCAGATACCAAATACTGTCCTTCTA
GTGTAGCCGTAGTTAGGCCACCACTTCAAGAACTCTGTAGCACCGCCTACATACCTCGCTCTGCTAATCCTGTTACCAGTGGCTGCTGCCAGTGGCGATAAGTCGTGTCTTACCGGGTTGGACTC
AAGACGATAGTTACCGGATAAGGCGCAGCGGTCGGGCTGAACGGGGGGTTCGTGCACACAGCCCAGCTTGGAGCGAACGACCTACACCGAACTGAGATACCTACAGCGTGAGCTATGAGAA
AGCGCCACGCTTCCCGAAGGGAGAAAGGCGGACAGGTATCCGGTAAGCGGCAGGGTCGGAACAGGAGAGCGCACGAGGGAGCTTCCAGGGGGAAACGCCTGGTATCTTTATAGTCCTGTCG
GGTTTCGCCACCTCTGACTTGAGCGTCGATTTTTGTGATGCTCGTCAGGGGGGCGGAGCCTATGGAAAAACGCCAGCAACGCGGCCTTTTTACGGTTCCTGGCCTTTTGCTGGCCTTTTGCTCAC
ATGTTCTTTCCTGCGTTATCCCCTGATTCTGTGGATAACCGTATTACCGCCTTTGAGTGAGCTGATACCGCTCGCCGCAGCCGAACGACCGAGCGCAGCGAGTCAGTGAGCGAGGAAGCGGAA
GAGCGCCTGATGCGGTATTTTCTCCTTACGCATCTGTGCGGTATTTCACACCGCATATGGTGCACTCTCAGTACAATCTGCTCTGATGCCGCATAGTTAAGCCAGTATACACTCCGCTATCGCTAC
GTGACTGGGTCATGGCTGCGCCCCGACACCCGCCAACACCCGCTGACGCGCCCTGACGGGCTTGTCTGCTCCCGGCATCCGCTTACAGACAAGCTGTGACCGTCTCCGGGAGCTGCATGTGTC
AGAGGTTTTCACCGTCATCACCGAAACGCGCGAGGCAGCTGCGGTAAAGCTCATCAGCGTGGTCGTGCAGCGATTCACAGATGTCTGCCTGTTCATCCGCGTCCAGCTCGTTGAGTTTCTCCAG
AAGCGTTAATGTCTGGCTTCTGATAAAGCGGGCCATGTTAAGGGCGGTTTTTTCCTGTTTGGTCACTGATGCCTCCGTGTAAGGGGGATTTCTGTTCATGGGGGTAATGATACCGATGAAACGA
GAGAGGATGCTCACGATACGGGTTACTGATGATGAACATGCCCGGTTACTGGAACGTTGTGAGGGTAAACAACTGGCGGTATGGATGCGGCGGGGGCGCGCCCAGCTGTCTAGGGCGGCGG
ATTTGTCCTACTCAGGAGAGCGTTCACCGACAAACAACAGATAAAACGAAAGGCCCAGTCTTTCGACTGAGCCTTTCGTTTTATTTGATGCCT

pT7-FepA

TAATACGACTCACTATAGGGGAATTGTGAGCGGATAACAATTCCCCTCTAGAAATAATTTTGTTTAACTTTAAGAAGGAGATATACATATGAACAAGAAGATTCATTCCCTGGCCTTGTTGGTCA
ATCTGGGGATTTATGGGGTAGCGCAGGCACAAGAGCCGACCGATACTCCTGTTTCACATGACGATACTATTGTCGTTACCGCCGCCGAGCAGAACTTACAGGCGCCTGGCGTTTCGACCATCAC
CGCAGATGAAATCCGCAAAAACCCGGTTGCCCGCGATGTGTCGAAGATCATCCGTACCATGCCAGGCGTTAACCTGACCGGTAACTCCACCAGTGGTCAGCGTGGGAATAACCGACAGATTGA
TATTCGCGGTATGGGTCCGGAAAACACGCTGATTTTGATTGACGGCAAGCCGGTAAGCAGCCGTAACTCGGTGCGTCAGGGCTGGCGTGGCGAGCGCGATACCCGTGGTGATACTTCCTGGG
TGCCACCTGAAATGATTGAACGTATTGAAGTTCTGCGTGGTCCGGCAGCTGCGCGTTATGGCAACGGCGCGGCGGGCGGCGTGGTTAACATCATTACCAAAAAAGGCAGCGGCGAGTGGCAC
GGCTCCTGGGACGCATATTTCAATGCGCCAGAACATAAAGAGGAAGGTGCCACCAAACGCACTAACTTTAGCCTGACCGGTCCGCTGGGCGACGAATTCAGCTTCCGTTTGTATGGCAACCTC
GACAAAACCCAGGCTGACGCGTGGGATATCAACCAGGGCCATCAGTCCGCGCGTGCCGGAACGTATGCCACGACGTTACCAGCCGGGCGCGAAGGGGTAATCAACAAAGATATTAATGGCGT
GGTGCGCTGGGATTTCGCGCCATTGCAATCGCTGGAACTGGAAGCAGGTTACAGCCGCCAGGGTAACCTGTATGCGGGCGACACCCAGAATACCAACTCCGATTCCTATACCCGCTCGAAATA
TGGCGATGAAACCAACCGTCTGTATCGCCAGAACTACGCGCTGACCTGGAACGGTGGCTGGGATAACGGCGTGACCACCAGCAACTGGGTGCAGTACGAACACACCCGTAACTCGCGTATTCC
GGAAGGTCTGGCGGGCGGTACCGAAGGGAAATTTAACGAAAAAGCGACACAGGATTTCGTCGATATCGATCTTGATGACGTGATGCTGCACAGCGAAGTTAACCTGCCGATTGATTTCCTCGT
TAACCAGACGCTGACGCTGGGTACGGAGTGGAATCAGCAACGGATGAAGGACTTAAGTTCCAACACCCAGGCACTGACCGGAACGAATACCGGTGGCGCTATTGATGGCGTGAGTACCACCG
ACCGTAGCCCGTATTCAAAAGCAGAAATTTTCTCGCTGTTTGCCGAAAACAACATGGAGCTGACTGACAGCACCATCGTAACGCCGGGGCTGCGTTTCGATCATCACAGTATTGTCGGCAATAA
CTGGAGCCCGGCGCTGAACATATCGCAAGGTTTAGGCGATGACTTCACGCTGAAAATGGGCATCGCCCGTGCTTATAAAGCGCCGAGCCTGTACCAGACTAACCCGAACTACATTCTCTACAGT
AAAGGTCAGGGTTGCTATGCCAGCGCGGGCGGCTGCTATCTGCAAGGTAACGATGACCTGAAAGCAGAAACCAGCATCAACAAAGAGATTGGTCTGGAGTTCAAACGCGACGGGTGGCTGG
CGGGCGTCACCTGGTTCCGTAACGATTATCGCAATAAGATTGAAGCGGGCTATGTGGCTGTAGGGCAAAACGCAGTCGGCACCGATCTCTATCAGTGGGATAACGTGCCGAAAGCGGTGGTT
GAAGGTCTGGAAGGATCGTTAAACGTACCGGTTAGCGAAACGGTGATGTGGACCAATAACATCACTTATATGCTGAAGAGTGAAAACAAAACCACGGGCGACCGTTTGTCGATCATCCCGGA
GTATACGTTGAACTCAACGCTGAGCTGGCAGGCACGGGAAGATTTGTCGATGCAAACGACCTTCACCTGGTACGGCAAGCAGCAGCCGAAGAAGTACAACTATAAAGGTCAGCCAGCGGTTG
GACCGGAAACCAAAGAAATTAGTCCTTACAGCATTGTTGGCCTGAGCGCGACCTGGGATGTGACGAAGAATGTCAGTCTGACCGGCGGCGTGGACAATCTGTTCGACAAACGTTTGTGGCGT
GCGGGTAATGCCCAGACCACGGGCGATTTGGCAGGGGCCAACTATATCGCCGGTGCCGGGGCGTATACCTATAACGAGCCGGGACGTACGTGGTATATGAGCGTAAACACCCACTTCTGACC
GCTGAGCAATAACTAGCATAACCCCTTGGGGCCTCTAAACGGGTCTTGAGGGGTTTTTTGTGCGGCCGCGTCGTGACTGGGAAAACCCTGGCGACTAGTCTTGGACTCCTGTTGATAGATCCAG
TAATGACCTCAGAACTCCATCTGGATTTGTTCAGAACGCTCGGTTGCCGCCGGGCGTTTTTTATTGGTGAGAATCCAGGGGTCCCCAATAATTACGATTTAAATTTGTGTCTCAAAATCTCTGATG
TTACATTGCACAAGATAAAAATATATCATCATGAACAATAAAACTGTCTGCTTACATAAACAGTAATACAAGGGGTGTTATGAGCCATATTCAGCGTGAAACGAGCTGTAGCCGTCCGCGTCTG
AACAGCAACATGGATGCGGATCTGTATGGCTATAAATGGGCGCGTGATAACGTGGGTCAGAGCGGCGCGACCATTTATCGTCTGTATGGCAAACCGGATGCGCCGGAACTGTTTCTGAAACAT
GGCAAAGGCAGCGTGGCGAACGATGTGACCGATGAAATGGTGCGTCTGAACTGGCTGACCGAATTTATGCCGCTGCCGACCATTAAACATTTTATTCGCACCCCGGATGATGCGTGGCTGCTG
ACCACCGCGATTCCGGGCAAAACCGCGTTTCAGGTGCTGGAAGAATATCCGGATAGCGGCGAAAACATTGTGGATGCGCTGGCCGTGTTTCTGCGTCGTCTGCATAGCATTCCGGTGTGCAAC
TGCCCGTTTAACAGCGATCGTGTGTTTCGTCTGGCCCAGGCGCAGAGCCGTATGAACAACGGCCTGGTGGATGCGAGCGATTTTGATGATGAACGTAACGGCTGGCCGGTGGAACAGGTGTG
GAAAGAAATGCATAAACTGCTGCCGTTTAGCCCGGATAGCGTGGTGACCCACGGCGATTTTAGCCTGGATAACCTGATTTTCGATGAAGGCAAACTGATTGGCTGCATTGATGTGGGCCGTGT
GGGCATTGCGGATCGTTATCAGGATCTGGCCATTCTGTGGAACTGCCTGGGCGAATTTAGCCCGAGCCTGCAAAAACGTCTGTTTCAGAAATATGGCATTGATAATCCGGATATGAACAAACTG
CAATTTCATCTGATGCTGGATGAATTTTTCTAATAATTAATTGGACCGCGGTCCGCGCGTTGTCCTTTTCCGCTGCATAACCCTGCTTCGGGGTCATTATAGCGATTTTTTCGGTATATCCATCCTT
TTTCGCACGATATACAGGATTTTGCCAAAGGGTTCGTGTAGACTTTCCTTGGTGTATCCAACGGCGTCAGCCGGGCAGGATAGGTGAAGTAGGCCCACCCGCGAGCGGGTGTTCCTTCTTCACT
GTCCCTTATTCGCACCTGGCGGTGCTCAACGGGAATCCTGCTCTGCGAGGCTGGCCGTAGGCCGGCCCCGTAGAAAAGATCAAAGGATCTTCTTGAGATCCTTTTTTTCTGCGCGTAATCTGCTG
CTTGCAAACAAAAAAACCACCGCTACCAGCGGTGGTTTGTTTGCCGGATCAAGAGCTACCAACTCTTTTTCCGAAGGTAACTGGCTTCAGCAGAGCGCAGATACCAAATACTGTCCTTCTAGTGT
AGCCGTAGTTAGGCCACCACTTCAAGAACTCTGTAGCACCGCCTACATACCTCGCTCTGCTAATCCTGTTACCAGTGGCTGCTGCCAGTGGCGATAAGTCGTGTCTTACCGGGTTGGACTCAAGA
CGATAGTTACCGGATAAGGCGCAGCGGTCGGGCTGAACGGGGGGTTCGTGCACACAGCCCAGCTTGGAGCGAACGACCTACACCGAACTGAGATACCTACAGCGTGAGCTATGAGAAAGCG
CCACGCTTCCCGAAGGGAGAAAGGCGGACAGGTATCCGGTAAGCGGCAGGGTCGGAACAGGAGAGCGCACGAGGGAGCTTCCAGGGGGAAACGCCTGGTATCTTTATAGTCCTGTCGGGTT
TCGCCACCTCTGACTTGAGCGTCGATTTTTGTGATGCTCGTCAGGGGGGCGGAGCCTATGGAAAAACGCCAGCAACGCGGCCTTTTTACGGTTCCTGGCCTTTTGCTGGCCTTTTGCTCACATGT
TCTTTCCTGCGTTATCCCCTGATTCTGTGGATAACCGTATTACCGCCTTTGAGTGAGCTGATACCGCTCGCCGCAGCCGAACGACCGAGCGCAGCGAGTCAGTGAGCGAGGAAGCGGAAGAGC
GCCTGATGCGGTATTTTCTCCTTACGCATCTGTGCGGTATTTCACACCGCATATGGTGCACTCTCAGTACAATCTGCTCTGATGCCGCATAGTTAAGCCAGTATACACTCCGCTATCGCTACGTGA
CTGGGTCATGGCTGCGCCCCGACACCCGCCAACACCCGCTGACGCGCCCTGACGGGCTTGTCTGCTCCCGGCATCCGCTTACAGACAAGCTGTGACCGTCTCCGGGAGCTGCATGTGTCAGAG
GTTTTCACCGTCATCACCGAAACGCGCGAGGCAGCTGCGGTAAAGCTCATCAGCGTGGTCGTGCAGCGATTCACAGATGTCTGCCTGTTCATCCGCGTCCAGCTCGTTGAGTTTCTCCAGAAGC
GTTAATGTCTGGCTTCTGATAAAGCGGGCCATGTTAAGGGCGGTTTTTTCCTGTTTGGTCACTGATGCCTCCGTGTAAGGGGGATTTCTGTTCATGGGGGTAATGATACCGATGAAACGAGAGA
GGATGCTCACGATACGGGTTACTGATGATGAACATGCCCGGTTACTGGAACGTTGTGAGGGTAAACAACTGGCGGTATGGATGCGGCGGGGGCGCGCCCAGCTGTCTAGGGCGGCGGATTT
GTCCTACTCAGGAGAGCGTTCACCGACAAACAACAGATAAAACGAAAGGCCCAGTCTTTCGACTGAGCCTTTCGTTTTATTTGATGCCTTTAATTAAAGCGGATAACAATTTCACACAGGAGGC
CGCCTAGGCCGCGGCCGCGCG

pT7-FepA ΔC128

TAATACGACTCACTATAGGGGAATTGTGAGCGGATAACAATTCCCCTCTAGAAATAATTTTGTTTAACTTTAAGAAGGAGATATACATATGAACAAGAAGATTCATTCCCTGGCCTTGTTGGTCA
ATCTGGGGATTTATGGGGTAGCGCAGGCAGCAGCTGCGCGTTATGGCAACGGCGCGGCGGGCGGCGTGGTTAACATCATTACCAAAAAAGGCAGCGGCGAGTGGCACGGCTCCTGGGACGC
ATATTTCAATGCGCCAGAACATAAAGAGGAAGGTGCCACCAAACGCACTAACTTTAGCCTGACCGGTCCGCTGGGCGACGAATTCAGCTTCCGTTTGTATGGCAACCTCGACAAAACCCAGGCT
GACGCGTGGGATATCAACCAGGGCCATCAGTCCGCGCGTGCCGGAACGTATGCCACGACGTTACCAGCCGGGCGCGAAGGGGTAATCAACAAAGATATTAATGGCGTGGTGCGCTGGGATTT
CGCGCCATTGCAATCGCTGGAACTGGAAGCAGGTTACAGCCGCCAGGGTAACCTGTATGCGGGCGACACCCAGAATACCAACTCCGATTCCTATACCCGCTCGAAATATGGCGATGAAACCAA
CCGTCTGTATCGCCAGAACTACGCGCTGACCTGGAACGGTGGCTGGGATAACGGCGTGACCACCAGCAACTGGGTGCAGTACGAACACACCCGTAACTCGCGTATTCCGGAAGGTCTGGCGG
GCGGTACCGAAGGGAAATTTAACGAAAAAGCGACACAGGATTTCGTCGATATCGATCTTGATGACGTGATGCTGCACAGCGAAGTTAACCTGCCGATTGATTTCCTCGTTAACCAGACGCTGA
CGCTGGGTACGGAGTGGAATCAGCAACGGATGAAGGACTTAAGTTCCAACACCCAGGCACTGACCGGAACGAATACCGGTGGCGCTATTGATGGCGTGAGTACCACCGACCGTAGCCCGTAT
TCAAAAGCAGAAATTTTCTCGCTGTTTGCCGAAAACAACATGGAGCTGACTGACAGCACCATCGTAACGCCGGGGCTGCGTTTCGATCATCACAGTATTGTCGGCAATAACTGGAGCCCGGCGC
TGAACATATCGCAAGGTTTAGGCGATGACTTCACGCTGAAAATGGGCATCGCCCGTGCTTATAAAGCGCCGAGCCTGTACCAGACTAACCCGAACTACATTCTCTACAGTAAAGGTCAGGGTTG
CTATGCCAGCGCGGGCGGCTGCTATCTGCAAGGTAACGATGACCTGAAAGCAGAAACCAGCATCAACAAAGAGATTGGTCTGGAGTTCAAACGCGACGGGTGGCTGGCGGGCGTCACCTGGT
TCCGTAACGATTATCGCAATAAGATTGAAGCGGGCTATGTGGCTGTAGGGCAAAACGCAGTCGGCACCGATCTCTATCAGTGGGATAACGTGCCGAAAGCGGTGGTTGAAGGTCTGGAAGGA
TCGTTAAACGTACCGGTTAGCGAAACGGTGATGTGGACCAATAACATCACTTATATGCTGAAGAGTGAAAACAAAACCACGGGCGACCGTTTGTCGATCATCCCGGAGTATACGTTGAACTCA
ACGCTGAGCTGGCAGGCACGGGAAGATTTGTCGATGCAAACGACCTTCACCTGGTACGGCAAGCAGCAGCCGAAGAAGTACAACTATAAAGGTCAGCCAGCGGTTGGACCGGAAACCAAAG
AAATTAGTCCTTACAGCATTGTTGGCCTGAGCGCGACCTGGGATGTGACGAAGAATGTCAGTCTGACCGGCGGCGTGGACAATCTGTTCGACAAACGTTTGTGGCGTGCGGGTAATGCCCAGA
CCACGGGCGATTTGGCAGGGGCCAACTATATCGCCGGTGCCGGGGCGTATACCTATAACGAGCCGGGACGTACGTGGTATATGAGCGTAAACACCCACTTCTGACCGCTGAGCAATAACTAGC
ATAACCCCTTGGGGCCTCTAAACGGGTCTTGAGGGGTTTTTTGAGCTTGTCGTGACTGGGAAAACCCTGGCGACTAGTCTTGGACTCCTGTTGATAGATCCAGTAATGACCTCAGAACTCCATCT
GGATTTGTTCAGAACGCTCGGTTGCCGCCGGGCGTTTTTTATTGGTGAGAATCCAGGGGTCCCCAATAATTACGATTTAAATTTGTGTCTCAAAATCTCTGATGTTACATTGCACAAGATAAAAA
TATATCATCATGAACAATAAAACTGTCTGCTTACATAAACAGTAATACAAGGGGTGTTATGAGCCATATTCAGCGTGAAACGAGCTGTAGCCGTCCGCGTCTGAACAGCAACATGGATGCGGAT
CTGTATGGCTATAAATGGGCGCGTGATAACGTGGGTCAGAGCGGCGCGACCATTTATCGTCTGTATGGCAAACCGGATGCGCCGGAACTGTTTCTGAAACATGGCAAAGGCAGCGTGGCGAA
CGATGTGACCGATGAAATGGTGCGTCTGAACTGGCTGACCGAATTTATGCCGCTGCCGACCATTAAACATTTTATTCGCACCCCGGATGATGCGTGGCTGCTGACCACCGCGATTCCGGGCAAA
ACCGCGTTTCAGGTGCTGGAAGAATATCCGGATAGCGGCGAAAACATTGTGGATGCGCTGGCCGTGTTTCTGCGTCGTCTGCATAGCATTCCGGTGTGCAACTGCCCGTTTAACAGCGATCGT
GTGTTTCGTCTGGCCCAGGCGCAGAGCCGTATGAACAACGGCCTGGTGGATGCGAGCGATTTTGATGATGAACGTAACGGCTGGCCGGTGGAACAGGTGTGGAAAGAAATGCATAAACTGCT
GCCGTTTAGCCCGGATAGCGTGGTGACCCACGGCGATTTTAGCCTGGATAACCTGATTTTCGATGAAGGCAAACTGATTGGCTGCATTGATGTGGGCCGTGTGGGCATTGCGGATCGTTATCA
GGATCTGGCCATTCTGTGGAACTGCCTGGGCGAATTTAGCCCGAGCCTGCAAAAACGTCTGTTTCAGAAATATGGCATTGATAATCCGGATATGAACAAACTGCAATTTCATCTGATGCTGGAT
GAATTTTTCTAATAATTAATTGGACCGCGGTCCGCGCGTTGTCCTTTTCCGCTGCATAACCCTGCTTCGGGGTCATTATAGCGATTTTTTCGGTATATCCATCCTTTTTCGCACGATATACAGGATT
TTGCCAAAGGGTTCGTGTAGACTTTCCTTGGTGTATCCAACGGCGTCAGCCGGGCAGGATAGGTGAAGTAGGCCCACCCGCGAGCGGGTGTTCCTTCTTCACTGTCCCTTATTCGCACCTGGCG
GTGCTCAACGGGAATCCTGCTCTGCGAGGCTGGCCGTAGGCCGGCCCCGTAGAAAAGATCAAAGGATCTTCTTGAGATCCTTTTTTTCTGCGCGTAATCTGCTGCTTGCAAACAAAAAAACCAC
CGCTACCAGCGGTGGTTTGTTTGCCGGATCAAGAGCTACCAACTCTTTTTCCGAAGGTAACTGGCTTCAGCAGAGCGCAGATACCAAATACTGTCCTTCTAGTGTAGCCGTAGTTAGGCCACCAC
TTCAAGAACTCTGTAGCACCGCCTACATACCTCGCTCTGCTAATCCTGTTACCAGTGGCTGCTGCCAGTGGCGATAAGTCGTGTCTTACCGGGTTGGACTCAAGACGATAGTTACCGGATAAGG
CGCAGCGGTCGGGCTGAACGGGGGGTTCGTGCACACAGCCCAGCTTGGAGCGAACGACCTACACCGAACTGAGATACCTACAGCGTGAGCTATGAGAAAGCGCCACGCTTCCCGAAGGGAG
AAAGGCGGACAGGTATCCGGTAAGCGGCAGGGTCGGAACAGGAGAGCGCACGAGGGAGCTTCCAGGGGGAAACGCCTGGTATCTTTATAGTCCTGTCGGGTTTCGCCACCTCTGACTTGAGC
GTCGATTTTTGTGATGCTCGTCAGGGGGGCGGAGCCTATGGAAAAACGCCAGCAACGCGGCCTTTTTACGGTTCCTGGCCTTTTGCTGGCCTTTTGCTCACATGTTCTTTCCTGCGTTATCCCCTG
ATTCTGTGGATAACCGTATTACCGCCTTTGAGTGAGCTGATACCGCTCGCCGCAGCCGAACGACCGAGCGCAGCGAGTCAGTGAGCGAGGAAGCGGAAGAGCGCCTGATGCGGTATTTTCTCC
TTACGCATCTGTGCGGTATTTCACACCGCATATGGTGCACTCTCAGTACAATCTGCTCTGATGCCGCATAGTTAAGCCAGTATACACTCCGCTATCGCTACGTGACTGGGTCATGGCTGCGCCCC
GACACCCGCCAACACCCGCTGACGCGCCCTGACGGGCTTGTCTGCTCCCGGCATCCGCTTACAGACAAGCTGTGACCGTCTCCGGGAGCTGCATGTGTCAGAGGTTTTCACCGTCATCACCGAA
ACGCGCGAGGCAGCTGCGGTAAAGCTCATCAGCGTGGTCGTGCAGCGATTCACAGATGTCTGCCTGTTCATCCGCGTCCAGCTCGTTGAGTTTCTCCAGAAGCGTTAATGTCTGGCTTCTGATA
AAGCGGGCCATGTTAAGGGCGGTTTTTTCCTGTTTGGTCACTGATGCCTCCGTGTAAGGGGGATTTCTGTTCATGGGGGTAATGATACCGATGAAACGAGAGAGGATGCTCACGATACGGGTT
ACTGATGATGAACATGCCCGGTTACTGGAACGTTGTGAGGGTAAACAACTGGCGGTATGGATGCGGCGGGGGCGCGCCCAGCTGTCTAGGGCGGCGGATTTGTCCTACTCAGGAGAGCGTTC
ACCGACAAACAACAGATAAAACGAAAGGCCCAGTCTTTCGACTGAGCCTTTCGTTTTATTTGATGCCTTTAATTAAAGGAATT

pT7-FepA ΔC128/ΔL

TTAATTAAAGGAATTTAATACGACTCACTATAGGGGAATTGTGAGCGGATAACAATTCCCCTCTAGAAATAATTTTGTTTAACTTTAAGAAGGAGATATACATATGAACAAGAAGATTCATTCCC
TGGCCTTGTTGGTCAATCTGGGGATTTATGGGGTAGCGCAGGCAGCCGCCGCTCGTTATGGCAACGGGGCGGCCGGGGGAGTTGTTAATATCATCACGAAGAAGGGGTCAGGGGAATGGCA
CGGGAGCTGGGACGCGTATTTCAATGCACCGGAACATGGCGCAACTAAGCGCACAAATTTCTCCTTAACGGGGCCACTGGGAGATGAGTTCAGTTTCCGCTTGTATGGCAACCTGGATAAGAC
CCAGGCACGTGAGGGGGTGATTAATAAAGATATTAACGGTGTAGTTCGTTGGGATTTCGCACCTCTGCAATCACTGGAGTTGGAGGCCGGCTACTCACGTCAAGGAAATTTATATGCTGAAAC
GAACCGTCTTTATCGCCAAAATTACGCCCTTACATGGAACGGAGGTTGGGACAATGGCGTCACTACGTCGAACTGGGTCCAATATGAGCACACTCGCAATTCGCGTATTCCCGATTTCGTAGAC
ATCGATTTAGATGATGTAATGTTACACTCTGAAGTAAATTTGCCCATTGACTTCCTTGTGAACCAAACTTTAACTTTGGGGACTGAGTGGAACCAACAGCGTATGAAAGACCTGTACTCTAAAGC
GGAGATCTTTAGTTTATTTGCGGAAAACAACATGGAGCTGACCGATAGCACGATTGTAACTCCAGGGTTACGTTTCGACCATCACAGTATTGTAGGCAATAACTGGTCCCCGGCTTTAAACATCT
CTCAAGGCCTTGGAGACGACTTCACTTTGAAAATGGGGATCGCGCGTGCCTATAAGGCCCCTAAAGCTGAAACTTCTATCAATAAGGAGATCGGCTTAGAGTTCAAACGCGACGGGTGGTTAG
CTGGGGTCACTTGGTTCCGTAACGATTACCGTAATAAAATTGAAGCTGGATATACAGACTTGTATCAATGGGATAACGTACCAAAGGCCGTCGTTGAAGGGTTGGAGGGCTCCTTAAATGTACC
CGTTAGCGAGACGGTTATGTGGACCAATAATATCACGTACATGCTGAAGTCAGAGAACAAAACCCCGGAATATACGCTGAACAGTACTTTGTCTTGGCAGGCTCGTGAGGATCTTAGCATGCA
GACTACTTTCACGTGGTATGGCAAGTCTCCTTACTCCATCGTCGGTCTTTCAGCTACGTGGGACGTCACAAAGAACGTGTCTTTGACGGGCGGTGTAGATAACTTATTTGATAAACGCGGGCGC
ACCTGGTATATGAGCGTCAACACTCACTTTTGACCGCTGAGCAATAACTAGCATAACCCCTTGGGGCCTCTAAACGGGTCTTGAGGGGTTTTTTGAGCTTGTCGTGACTGGGAAAACCCTGGCG
ACTAGTCTTGGACTCCTGTTGATAGATCCAGTAATGACCTCAGAACTCCATCTGGATTTGTTCAGAACGCTCGGTTGCCGCCGGGCGTTTTTTATTGGTGAGAATCCAGGGGTCCCCAATAATTA
CGATTTAAATTTGTGTCTCAAAATCTCTGATGTTACATTGCACAAGATAAAAATATATCATCATGAACAATAAAACTGTCTGCTTACATAAACAGTAATACAAGGGGTGTTATGAGCCATATTCAG
CGTGAAACGAGCTGTAGCCGTCCGCGTCTGAACAGCAACATGGATGCGGATCTGTATGGCTATAAATGGGCGCGTGATAACGTGGGTCAGAGCGGCGCGACCATTTATCGTCTGTATGGCAA
ACCGGATGCGCCGGAACTGTTTCTGAAACATGGCAAAGGCAGCGTGGCGAACGATGTGACCGATGAAATGGTGCGTCTGAACTGGCTGACCGAATTTATGCCGCTGCCGACCATTAAACATTT
TATTCGCACCCCGGATGATGCGTGGCTGCTGACCACCGCGATTCCGGGCAAAACCGCGTTTCAGGTGCTGGAAGAATATCCGGATAGCGGCGAAAACATTGTGGATGCGCTGGCCGTGTTTCT
GCGTCGTCTGCATAGCATTCCGGTGTGCAACTGCCCGTTTAACAGCGATCGTGTGTTTCGTCTGGCCCAGGCGCAGAGCCGTATGAACAACGGCCTGGTGGATGCGAGCGATTTTGATGATGA
ACGTAACGGCTGGCCGGTGGAACAGGTGTGGAAAGAAATGCATAAACTGCTGCCGTTTAGCCCGGATAGCGTGGTGACCCACGGCGATTTTAGCCTGGATAACCTGATTTTCGATGAAGGCA
AACTGATTGGCTGCATTGATGTGGGCCGTGTGGGCATTGCGGATCGTTATCAGGATCTGGCCATTCTGTGGAACTGCCTGGGCGAATTTAGCCCGAGCCTGCAAAAACGTCTGTTTCAGAAATA
TGGCATTGATAATCCGGATATGAACAAACTGCAATTTCATCTGATGCTGGATGAATTTTTCTAATAATTAATTGGACCGCGGTCCGCGCGTTGTCCTTTTCCGCTGCATAACCCTGCTTCGGGGTC
ATTATAGCGATTTTTTCGGTATATCCATCCTTTTTCGCACGATATACAGGATTTTGCCAAAGGGTTCGTGTAGACTTTCCTTGGTGTATCCAACGGCGTCAGCCGGGCAGGATAGGTGAAGTAGG
CCCACCCGCGAGCGGGTGTTCCTTCTTCACTGTCCCTTATTCGCACCTGGCGGTGCTCAACGGGAATCCTGCTCTGCGAGGCTGGCCGTAGGCCGGCCCCGTAGAAAAGATCAAAGGATCTTCT
TGAGATCCTTTTTTTCTGCGCGTAATCTGCTGCTTGCAAACAAAAAAACCACCGCTACCAGCGGTGGTTTGTTTGCCGGATCAAGAGCTACCAACTCTTTTTCCGAAGGTAACTGGCTTCAGCAG
AGCGCAGATACCAAATACTGTCCTTCTAGTGTAGCCGTAGTTAGGCCACCACTTCAAGAACTCTGTAGCACCGCCTACATACCTCGCTCTGCTAATCCTGTTACCAGTGGCTGCTGCCAGTGGCG
ATAAGTCGTGTCTTACCGGGTTGGACTCAAGACGATAGTTACCGGATAAGGCGCAGCGGTCGGGCTGAACGGGGGGTTCGTGCACACAGCCCAGCTTGGAGCGAACGACCTACACCGAACTG
AGATACCTACAGCGTGAGCTATGAGAAAGCGCCACGCTTCCCGAAGGGAGAAAGGCGGACAGGTATCCGGTAAGCGGCAGGGTCGGAACAGGAGAGCGCACGAGGGAGCTTCCAGGGGGA
AACGCCTGGTATCTTTATAGTCCTGTCGGGTTTCGCCACCTCTGACTTGAGCGTCGATTTTTGTGATGCTCGTCAGGGGGGCGGAGCCTATGGAAAAACGCCAGCAACGCGGCCTTTTTACGGT
TCCTGGCCTTTTGCTGGCCTTTTGCTCACATGTTCTTTCCTGCGTTATCCCCTGATTCTGTGGATAACCGTATTACCGCCTTTGAGTGAGCTGATACCGCTCGCCGCAGCCGAACGACCGAGCGCA
GCGAGTCAGTGAGCGAGGAAGCGGAAGAGCGCCTGATGCGGTATTTTCTCCTTACGCATCTGTGCGGTATTTCACACCGCATATGGTGCACTCTCAGTACAATCTGCTCTGATGCCGCATAGTT
AAGCCAGTATACACTCCGCTATCGCTACGTGACTGGGTCATGGCTGCGCCCCGACACCCGCCAACACCCGCTGACGCGCCCTGACGGGCTTGTCTGCTCCCGGCATCCGCTTACAGACAAGCTG
TGACCGTCTCCGGGAGCTGCATGTGTCAGAGGTTTTCACCGTCATCACCGAAACGCGCGAGGCAGCTGCGGTAAAGCTCATCAGCGTGGTCGTGCAGCGATTCACAGATGTCTGCCTGTTCATC
CGCGTCCAGCTCGTTGAGTTTCTCCAGAAGCGTTAATGTCTGGCTTCTGATAAAGCGGGCCATGTTAAGGGCGGTTTTTTCCTGTTTGGTCACTGATGCCTCCGTGTAAGGGGGATTTCTGTTCA
TGGGGGTAATGATACCGATGAAACGAGAGAGGATGCTCACGATACGGGTTACTGATGATGAACATGCCCGGTTACTGGAACGTTGTGAGGGTAAACAACTGGCGGTATGGATGCGGCGGGG
GCGCGCCCAGCTGTCTAGGGCGGCGGATTTGTCCTACTCAGGAGAGCGTTCACCGACAAACAACAGATAAAACGAAAGGCCCAGTCTTTCGACTGAGCCTTTCGTTTTATTTGATGCCT

pT7-FepA ΔC

TAATACGACTCACTATAGGGGAATTGTGAGCGGATAACAATTCCCCTCTAGAAATAATTTTGTTTAACTTTAAGAAGGAGATATACATATGAACAAGAAGATTCATTCCCTGGCCTTGTTGGTCA
ATCTGGGGATTTATGGGGTAGCGCAGGCAGGCGAGTGGCACGGCTCCTGGGACGCATATTTCAATGCGCCAGAACATAAAGAGGAAGGTGCCACCAAACGCACTAACTTTAGCCTGACCGGT
CCGCTGGGCGACGAATTCAGCTTCCGTTTGTATGGCAACCTCGACAAAACCCAGGCTGACGCGTGGGATATCAACCAGGGCCATCAGTCCGCGCGTGCCGGAACGTATGCCACGACGTTACCA
GCCGGGCGCGAAGGGGTAATCAACAAAGATATTAATGGCGTGGTGCGCTGGGATTTCGCGCCATTGCAATCGCTGGAACTGGAAGCAGGTTACAGCCGCCAGGGTAACCTGTATGCGGGCG
ACACCCAGAATACCAACTCCGATTCCTATACCCGCTCGAAATATGGCGATGAAACCAACCGTCTGTATCGCCAGAACTACGCGCTGACCTGGAACGGTGGCTGGGATAACGGCGTGACCACCA
GCAACTGGGTGCAGTACGAACACACCCGTAACTCGCGTATTCCGGAAGGTCTGGCGGGCGGTACCGAAGGGAAATTTAACGAAAAAGCGACACAGGATTTCGTCGATATCGATCTTGATGAC
GTGATGCTGCACAGCGAAGTTAACCTGCCGATTGATTTCCTCGTTAACCAGACGCTGACGCTGGGTACGGAGTGGAATCAGCAACGGATGAAGGACTTAAGTTCCAACACCCAGGCACTGACC
GGAACGAATACCGGTGGCGCTATTGATGGCGTGAGTACCACCGACCGTAGCCCGTATTCAAAAGCAGAAATTTTCTCGCTGTTTGCCGAAAACAACATGGAGCTGACTGACAGCACCATCGTA
ACGCCGGGGCTGCGTTTCGATCATCACAGTATTGTCGGCAATAACTGGAGCCCGGCGCTGAACATATCGCAAGGTTTAGGCGATGACTTCACGCTGAAAATGGGCATCGCCCGTGCTTATAAA
GCGCCGAGCCTGTACCAGACTAACCCGAACTACATTCTCTACAGTAAAGGTCAGGGTTGCTATGCCAGCGCGGGCGGCTGCTATCTGCAAGGTAACGATGACCTGAAAGCAGAAACCAGCATC
AACAAAGAGATTGGTCTGGAGTTCAAACGCGACGGGTGGCTGGCGGGCGTCACCTGGTTCCGTAACGATTATCGCAATAAGATTGAAGCGGGCTATGTGGCTGTAGGGCAAAACGCAGTCGG
CACCGATCTCTATCAGTGGGATAACGTGCCGAAAGCGGTGGTTGAAGGTCTGGAAGGATCGTTAAACGTACCGGTTAGCGAAACGGTGATGTGGACCAATAACATCACTTATATGCTGAAGA
GTGAAAACAAAACCACGGGCGACCGTTTGTCGATCATCCCGGAGTATACGTTGAACTCAACGCTGAGCTGGCAGGCACGGGAAGATTTGTCGATGCAAACGACCTTCACCTGGTACGGCAAGC
AGCAGCCGAAGAAGTACAACTATAAAGGTCAGCCAGCGGTTGGACCGGAAACCAAAGAAATTAGTCCTTACAGCATTGTTGGCCTGAGCGCGACCTGGGATGTGACGAAGAATGTCAGTCTG
ACCGGCGGCGTGGACAATCTGTTCGACAAACGTTTGTGGCGTGCGGGTAATGCCCAGACCACGGGCGATTTGGCAGGGGCCAACTATATCGCCGGTGCCGGGGCGTATACCTATAACGAGCC
GGGACGTACGTGGTATATGAGCGTAAACACCCACTTCTGACCGCTGAGCAATAACTAGCATAACCCCTTGGGGCCTCTAAACGGGTCTTGAGGGGTTTTTTGAGCTTGTCGTGACTGGGAAAA
CCCTGGCGACTAGTCTTGGACTCCTGTTGATAGATCCAGTAATGACCTCAGAACTCCATCTGGATTTGTTCAGAACGCTCGGTTGCCGCCGGGCGTTTTTTATTGGTGAGAATCCAGGGGTCCCC
AATAATTACGATTTAAATTTGTGTCTCAAAATCTCTGATGTTACATTGCACAAGATAAAAATATATCATCATGAACAATAAAACTGTCTGCTTACATAAACAGTAATACAAGGGGTGTTATGAGCC
ATATTCAGCGTGAAACGAGCTGTAGCCGTCCGCGTCTGAACAGCAACATGGATGCGGATCTGTATGGCTATAAATGGGCGCGTGATAACGTGGGTCAGAGCGGCGCGACCATTTATCGTCTGT
ATGGCAAACCGGATGCGCCGGAACTGTTTCTGAAACATGGCAAAGGCAGCGTGGCGAACGATGTGACCGATGAAATGGTGCGTCTGAACTGGCTGACCGAATTTATGCCGCTGCCGACCATT
AAACATTTTATTCGCACCCCGGATGATGCGTGGCTGCTGACCACCGCGATTCCGGGCAAAACCGCGTTTCAGGTGCTGGAAGAATATCCGGATAGCGGCGAAAACATTGTGGATGCGCTGGCC
GTGTTTCTGCGTCGTCTGCATAGCATTCCGGTGTGCAACTGCCCGTTTAACAGCGATCGTGTGTTTCGTCTGGCCCAGGCGCAGAGCCGTATGAACAACGGCCTGGTGGATGCGAGCGATTTTG
ATGATGAACGTAACGGCTGGCCGGTGGAACAGGTGTGGAAAGAAATGCATAAACTGCTGCCGTTTAGCCCGGATAGCGTGGTGACCCACGGCGATTTTAGCCTGGATAACCTGATTTTCGATG
AAGGCAAACTGATTGGCTGCATTGATGTGGGCCGTGTGGGCATTGCGGATCGTTATCAGGATCTGGCCATTCTGTGGAACTGCCTGGGCGAATTTAGCCCGAGCCTGCAAAAACGTCTGTTTC
AGAAATATGGCATTGATAATCCGGATATGAACAAACTGCAATTTCATCTGATGCTGGATGAATTTTTCTAATAATTAATTGGACCGCGGTCCGCGCGTTGTCCTTTTCCGCTGCATAACCCTGCTT
CGGGGTCATTATAGCGATTTTTTCGGTATATCCATCCTTTTTCGCACGATATACAGGATTTTGCCAAAGGGTTCGTGTAGACTTTCCTTGGTGTATCCAACGGCGTCAGCCGGGCAGGATAGGTG
AAGTAGGCCCACCCGCGAGCGGGTGTTCCTTCTTCACTGTCCCTTATTCGCACCTGGCGGTGCTCAACGGGAATCCTGCTCTGCGAGGCTGGCCGTAGGCCGGCCCCGTAGAAAAGATCAAAG
GATCTTCTTGAGATCCTTTTTTTCTGCGCGTAATCTGCTGCTTGCAAACAAAAAAACCACCGCTACCAGCGGTGGTTTGTTTGCCGGATCAAGAGCTACCAACTCTTTTTCCGAAGGTAACTGGCT
TCAGCAGAGCGCAGATACCAAATACTGTCCTTCTAGTGTAGCCGTAGTTAGGCCACCACTTCAAGAACTCTGTAGCACCGCCTACATACCTCGCTCTGCTAATCCTGTTACCAGTGGCTGCTGCC
AGTGGCGATAAGTCGTGTCTTACCGGGTTGGACTCAAGACGATAGTTACCGGATAAGGCGCAGCGGTCGGGCTGAACGGGGGGTTCGTGCACACAGCCCAGCTTGGAGCGAACGACCTACAC
CGAACTGAGATACCTACAGCGTGAGCTATGAGAAAGCGCCACGCTTCCCGAAGGGAGAAAGGCGGACAGGTATCCGGTAAGCGGCAGGGTCGGAACAGGAGAGCGCACGAGGGAGCTTCC
AGGGGGAAACGCCTGGTATCTTTATAGTCCTGTCGGGTTTCGCCACCTCTGACTTGAGCGTCGATTTTTGTGATGCTCGTCAGGGGGGCGGAGCCTATGGAAAAACGCCAGCAACGCGGCCTT
TTTACGGTTCCTGGCCTTTTGCTGGCCTTTTGCTCACATGTTCTTTCCTGCGTTATCCCCTGATTCTGTGGATAACCGTATTACCGCCTTTGAGTGAGCTGATACCGCTCGCCGCAGCCGAACGACC
GAGCGCAGCGAGTCAGTGAGCGAGGAAGCGGAAGAGCGCCTGATGCGGTATTTTCTCCTTACGCATCTGTGCGGTATTTCACACCGCATATGGTGCACTCTCAGTACAATCTGCTCTGATGCC
GCATAGTTAAGCCAGTATACACTCCGCTATCGCTACGTGACTGGGTCATGGCTGCGCCCCGACACCCGCCAACACCCGCTGACGCGCCCTGACGGGCTTGTCTGCTCCCGGCATCCGCTTACAG
ACAAGCTGTGACCGTCTCCGGGAGCTGCATGTGTCAGAGGTTTTCACCGTCATCACCGAAACGCGCGAGGCAGCTGCGGTAAAGCTCATCAGCGTGGTCGTGCAGCGATTCACAGATGTCTGC
CTGTTCATCCGCGTCCAGCTCGTTGAGTTTCTCCAGAAGCGTTAATGTCTGGCTTCTGATAAAGCGGGCCATGTTAAGGGCGGTTTTTTCCTGTTTGGTCACTGATGCCTCCGTGTAAGGGGGAT
TTCTGTTCATGGGGGTAATGATACCGATGAAACGAGAGAGGATGCTCACGATACGGGTTACTGATGATGAACATGCCCGGTTACTGGAACGTTGTGAGGGTAAACAACTGGCGGTATGGATG
CGGCGGGGGCGCGCCCAGCTGTCTAGGGCGGCGGATTTGTCCTACTCAGGAGAGCGTTCACCGACAAACAACAGATAAAACGAAAGGCCCAGTCTTTCGACTGAGCCTTTCGTTTTATTTGAT
GCCTTTAATTAAAGGAATT

pT7-FepA ΔC/Δ4L

TTAATTAAAGCGGATAACAATTTCACACAGGAGGCCGCCTAGGCCGCGGCCGCGCGTAATACGACTCACTATAGGGGAATTGTGAGCGGATAACAATTCCCCTCTAGAAATAATTTTGTTTAAC
TTTAAGAAGGAGATATACATATGAACAAGAAGATTCATTCCCTGGCCTTGTTGGTCAATCTGGGGATTTATGGGGTAGCGCAGGCAGGCGAGTGGCACGGCTCCTGGGACGCATATTTCAATG
CGCCAGAACATAAAGAGGAAGGTGCCACCAAACGCACTAACTTTAGCCTGACCGGTCCGCTGGGCGACGAATTCAGCTTCCGTTTGTATGGCAACCTCGACAAAACCCAGCGCGAAGGGGTAA
TCAACAAAGATATTAATGGCGTGGTGCGCTGGGATTTCGCGCCATTGCAATCGCTGGAACTGGAAGCAGGTTACAGCCGCCAGGGTAACCTGTATGCGGGCGACACCCAGAATACCAACTCCG
ATTCCTATACCCGCTCGAAATATGGCGATGAAACCAACCGTCTGTATCGCCAGAACTACGCGCTGACCTGGAACGGTGGCTGGGATAACGGCGTGACCACCAGCAACTGGGTGCAGTACGAAC
ACACCCGTAACTCGCGTATTCCGGAAGGTCTGGCGGGCGGTACCGAAGGGAAATTTAACGAAAAAGCGACACAGGATTTCGTCGATATCGATCTTGATGACGTGATGCTGCACAGCGAAGTTA
ACCTGCCGATTGATTTCCTCGTTAACCAGACGCTGACGCTGGGTACGGAGTGGAATCAGCAACGGATGAAGGACTTATATTCAAAAGCAGAAATTTTCTCGCTGTTTGCCGAAAACAACATGGA
GCTGACTGACAGCACCATCGTAACGCCGGGGCTGCGTTTCGATCATCACAGTATTGTCGGCAATAACTGGAGCCCGGCGCTGAACATATCGCAAGGTTTAGGCGATGACTTCACGCTGAAAAT
GGGCATCGCCCGTGCTTATAAAGCGCCGAAAGCAGAAACCAGCATCAACAAAGAGATTGGTCTGGAGTTCAAACGCGACGGGTGGCTGGCGGGCGTCACCTGGTTCCGTAACGATTATCGCA
ATAAGATTGAAGCGGGCTATGTGGCTGTAGGGCAAAACGCAGTCGGCACCGATCTCTATCAGTGGGATAACGTGCCGAAAGCGGTGGTTGAAGGTCTGGAAGGATCGTTAAACGTACCGGTT
AGCGAAACGGTGATGTGGACCAATAACATCACTTATATGCTGAAGAGTGAAAACAAAACCACGGGCGACCGTTTGTCGATCATCCCGGAGTATACGTTGAACTCAACGCTGAGCTGGCAGGCA
CGGGAAGATTTGTCGATGCAAACGACCTTCACCTGGTACGGCAAGCAGCAGCCGAAGAAGTACAACTATAAAGGTCAGCCAGCGGTTGGACCGGAAACCAAAGAAATTAGTCCTTACAGCAT
TGTTGGCCTGAGCGCGACCTGGGATGTGACGAAGAATGTCAGTCTGACCGGCGGCGTGGACAATCTGTTCGACAAACGTGGACGTACGTGGTATATGAGCGTAAACACCCACTTCTGACCGCT
GAGCAATAACTAGCATAACCCCTTGGGGCCTCTAAACGGGTCTTGAGGGGTTTTTTGTGCGGCCGCGTCGTGACTGGGAAAACCCTGGCGACTAGTCTTGGACTCCTGTTGATAGATCCAGTAA
TGACCTCAGAACTCCATCTGGATTTGTTCAGAACGCTCGGTTGCCGCCGGGCGTTTTTTATTGGTGAGAATCCAGGGGTCCCCAATAATTACGATTTAAATTTGTGTCTCAAAATCTCTGATGTTA
CATTGCACAAGATAAAAATATATCATCATGAACAATAAAACTGTCTGCTTACATAAACAGTAATACAAGGGGTGTTATGAGCCATATTCAGCGTGAAACGAGCTGTAGCCGTCCGCGTCTGAAC
AGCAACATGGATGCGGATCTGTATGGCTATAAATGGGCGCGTGATAACGTGGGTCAGAGCGGCGCGACCATTTATCGTCTGTATGGCAAACCGGATGCGCCGGAACTGTTTCTGAAACATGG
CAAAGGCAGCGTGGCGAACGATGTGACCGATGAAATGGTGCGTCTGAACTGGCTGACCGAATTTATGCCGCTGCCGACCATTAAACATTTTATTCGCACCCCGGATGATGCGTGGCTGCTGAC
CACCGCGATTCCGGGCAAAACCGCGTTTCAGGTGCTGGAAGAATATCCGGATAGCGGCGAAAACATTGTGGATGCGCTGGCCGTGTTTCTGCGTCGTCTGCATAGCATTCCGGTGTGCAACTG
CCCGTTTAACAGCGATCGTGTGTTTCGTCTGGCCCAGGCGCAGAGCCGTATGAACAACGGCCTGGTGGATGCGAGCGATTTTGATGATGAACGTAACGGCTGGCCGGTGGAACAGGTGTGGA
AAGAAATGCATAAACTGCTGCCGTTTAGCCCGGATAGCGTGGTGACCCACGGCGATTTTAGCCTGGATAACCTGATTTTCGATGAAGGCAAACTGATTGGCTGCATTGATGTGGGCCGTGTGG
GCATTGCGGATCGTTATCAGGATCTGGCCATTCTGTGGAACTGCCTGGGCGAATTTAGCCCGAGCCTGCAAAAACGTCTGTTTCAGAAATATGGCATTGATAATCCGGATATGAACAAACTGCA
ATTTCATCTGATGCTGGATGAATTTTTCTAATAATTAATTGGACCGCGGTCCGCGCGTTGTCCTTTTCCGCTGCATAACCCTGCTTCGGGGTCATTATAGCGATTTTTTCGGTATATCCATCCTTTTT
CGCACGATATACAGGATTTTGCCAAAGGGTTCGTGTAGACTTTCCTTGGTGTATCCAACGGCGTCAGCCGGGCAGGATAGGTGAAGTAGGCCCACCCGCGAGCGGGTGTTCCTTCTTCACTGTC
CCTTATTCGCACCTGGCGGTGCTCAACGGGAATCCTGCTCTGCGAGGCTGGCCGTAGGCCGGCCCCGTAGAAAAGATCAAAGGATCTTCTTGAGATCCTTTTTTTCTGCGCGTAATCTGCTGCTT
GCAAACAAAAAAACCACCGCTACCAGCGGTGGTTTGTTTGCCGGATCAAGAGCTACCAACTCTTTTTCCGAAGGTAACTGGCTTCAGCAGAGCGCAGATACCAAATACTGTCCTTCTAGTGTAG
CCGTAGTTAGGCCACCACTTCAAGAACTCTGTAGCACCGCCTACATACCTCGCTCTGCTAATCCTGTTACCAGTGGCTGCTGCCAGTGGCGATAAGTCGTGTCTTACCGGGTTGGACTCAAGACG
ATAGTTACCGGATAAGGCGCAGCGGTCGGGCTGAACGGGGGGTTCGTGCACACAGCCCAGCTTGGAGCGAACGACCTACACCGAACTGAGATACCTACAGCGTGAGCTATGAGAAAGCGCC
ACGCTTCCCGAAGGGAGAAAGGCGGACAGGTATCCGGTAAGCGGCAGGGTCGGAACAGGAGAGCGCACGAGGGAGCTTCCAGGGGGAAACGCCTGGTATCTTTATAGTCCTGTCGGGTTTC
GCCACCTCTGACTTGAGCGTCGATTTTTGTGATGCTCGTCAGGGGGGCGGAGCCTATGGAAAAACGCCAGCAACGCGGCCTTTTTACGGTTCCTGGCCTTTTGCTGGCCTTTTGCTCACATGTTC
TTTCCTGCGTTATCCCCTGATTCTGTGGATAACCGTATTACCGCCTTTGAGTGAGCTGATACCGCTCGCCGCAGCCGAACGACCGAGCGCAGCGAGTCAGTGAGCGAGGAAGCGGAAGAGCGC
CTGATGCGGTATTTTCTCCTTACGCATCTGTGCGGTATTTCACACCGCATATGGTGCACTCTCAGTACAATCTGCTCTGATGCCGCATAGTTAAGCCAGTATACACTCCGCTATCGCTACGTGACT
GGGTCATGGCTGCGCCCCGACACCCGCCAACACCCGCTGACGCGCCCTGACGGGCTTGTCTGCTCCCGGCATCCGCTTACAGACAAGCTGTGACCGTCTCCGGGAGCTGCATGTGTCAGAGGT
TTTCACCGTCATCACCGAAACGCGCGAGGCAGCTGCGGTAAAGCTCATCAGCGTGGTCGTGCAGCGATTCACAGATGTCTGCCTGTTCATCCGCGTCCAGCTCGTTGAGTTTCTCCAGAAGCGT
TAATGTCTGGCTTCTGATAAAGCGGGCCATGTTAAGGGCGGTTTTTTCCTGTTTGGTCACTGATGCCTCCGTGTAAGGGGGATTTCTGTTCATGGGGGTAATGATACCGATGAAACGAGAGAG
GATGCTCACGATACGGGTTACTGATGATGAACATGCCCGGTTACTGGAACGTTGTGAGGGTAAACAACTGGCGGTATGGATGCGGCGGGGGCGCGCCCAGCTGTCTAGGGCGGCGGATTTGT
CCTACTCAGGAGAGCGTTCACCGACAAACAACAGATAAAACGAAAGGCCCAGTCTTTCGACTGAGCCTTTCGTTTTATTTGATGCCT

pT7-FepA ΔC/Δ4L NSEGS

TTAATTAAAGCGGATAACAATTTCACACAGGAGGCCGCCTAGGCCGCGGCCGCGCGTAATACGACTCACTATAGGGGAATTGTGAGCGGATAACAATTCCCCTCTAGAAATAATTTTGTTTAAC
TTTAAGAAGGAGATATACATATGAACAAGAAGATTCATTCCCTGGCCTTGTTGGTCAATCTGGGGATTTATGGGGTAGCGCAGGCAGGCGAGTGGCACGGCTCCTGGGACGCATATTTCAATG
CGCCAGAACATAAAGAGGAAGGTGCCACCAAACGCACTAACTTTAGCCTGACCGGTCCGCTGGGCGACGAATTCAGCTTCCGTTTGTATGGCAACCTCGACAAAACCAATAGTGAAGGTAGCG
GGGTAATCAACAAAGATATTAATGGCGTGGTGCGCTGGGATTTCGCGCCATTGCAATCGCTGGAACTGGAAGCAGGTTACAGCCGCCAGGGTAACCTGTATGCGGGCGACACCCAGAATACC
AACTCCGATTCCTATACCCGCTCGAAATATGGCGATGAAACCAACCGTCTGTATCGCCAGAACTACGCGCTGACCTGGAACGGTGGCTGGGATAACGGCGTGACCACCAGCAACTGGGTGCAG
TACGAACACACCCGTAACTCGCGTATTCCGGAAGGTCTGGCGGGCGGTACCGAAGGGAAATTTAACGAAAAAGCGACACAGGATTTCGTCGATATCGATCTTGATGACGTGATGCTGCACAGC
GAAGTTAACCTGCCGATTGATTTCCTCGTTAACCAGACGCTGACGCTGGGTACGGAGTGGAATCAGCAACGGATGAAGAATAGTGAAGGTAGCAAAGCAGAAATTTTCTCGCTGTTTGCCGAA
AACAACATGGAGCTGACTGACAGCACCATCGTAACGCCGGGGCTGCGTTTCGATCATCACAGTATTGTCGGCAATAACTGGAGCCCGGCGCTGAACATATCGCAAGGTTTAGGCGATGACTTC
ACGCTGAAAATGGGCATCGCCCGTGCTTATAAAAATAGTGAAGGTAGCGAAACCAGCATCAACAAAGAGATTGGTCTGGAGTTCAAACGCGACGGGTGGCTGGCGGGCGTCACCTGGTTCCG
TAACGATTATCGCAATAAGATTGAAGCGGGCTATGTGGCTGTAGGGCAAAACGCAGTCGGCACCGATCTCTATCAGTGGGATAACGTGCCGAAAGCGGTGGTTGAAGGTCTGGAAGGATCGT
TAAACGTACCGGTTAGCGAAACGGTGATGTGGACCAATAACATCACTTATATGCTGAAGAGTGAAAACAAAACCACGGGCGACCGTTTGTCGATCATCCCGGAGTATACGTTGAACTCAACGC
TGAGCTGGCAGGCACGGGAAGATTTGTCGATGCAAACGACCTTCACCTGGTACGGCAAGCAGCAGCCGAAGAAGTACAACTATAAAGGTCAGCCAGCGGTTGGACCGGAAACCAAAGAAATT
AGTCCTTACAGCATTGTTGGCCTGAGCGCGACCTGGGATGTGACGAAGAATGTCAGTCTGACCGGCGGCGTGGACAATCTGTTCGACAATAGTGAAGGTAGCTGGTATATGAGCGTAAACACC
CACTTCTGACCGCTGAGCAATAACTAGCATAACCCCTTGGGGCCTCTAAACGGGTCTTGAGGGGTTTTTTGGAAAACCCTGGCGACTAGTCTTGGACTCCTGTTGATAGATCCAGTAATGACCTC
AGAACTCCATCTGGATTTGTTCAGAACGCTCGGTTGCCGCCGGGCGTTTTTTATTGGTGAGAATCCAGGGGTCCCCAATAATTACGATTTAAATTTGTGTCTCAAAATCTCTGATGTTACATTGCA
CAAGATAAAAATATATCATCATGAACAATAAAACTGTCTGCTTACATAAACAGTAATACAAGGGGTGTTATGAGCCATATTCAGCGTGAAACGAGCTGTAGCCGTCCGCGTCTGAACAGCAACA
TGGATGCGGATCTGTATGGCTATAAATGGGCGCGTGATAACGTGGGTCAGAGCGGCGCGACCATTTATCGTCTGTATGGCAAACCGGATGCGCCGGAACTGTTTCTGAAACATGGCAAAGGC
AGCGTGGCGAACGATGTGACCGATGAAATGGTGCGTCTGAACTGGCTGACCGAATTTATGCCGCTGCCGACCATTAAACATTTTATTCGCACCCCGGATGATGCGTGGCTGCTGACCACCGCG
ATTCCGGGCAAAACCGCGTTTCAGGTGCTGGAAGAATATCCGGATAGCGGCGAAAACATTGTGGATGCGCTGGCCGTGTTTCTGCGTCGTCTGCATAGCATTCCGGTGTGCAACTGCCCGTTTA
ACAGCGATCGTGTGTTTCGTCTGGCCCAGGCGCAGAGCCGTATGAACAACGGCCTGGTGGATGCGAGCGATTTTGATGATGAACGTAACGGCTGGCCGGTGGAACAGGTGTGGAAAGAAAT
GCATAAACTGCTGCCGTTTAGCCCGGATAGCGTGGTGACCCACGGCGATTTTAGCCTGGATAACCTGATTTTCGATGAAGGCAAACTGATTGGCTGCATTGATGTGGGCCGTGTGGGCATTGC
GGATCGTTATCAGGATCTGGCCATTCTGTGGAACTGCCTGGGCGAATTTAGCCCGAGCCTGCAAAAACGTCTGTTTCAGAAATATGGCATTGATAATCCGGATATGAACAAACTGCAATTTCAT
CTGATGCTGGATGAATTTTTCTAATAATTAATTGGACCGCGGTCCGCGCGTTGTCCTTTTCCGCTGCATAACCCTGCTTCGGGGTCATTATAGCGATTTTTTCGGTATATCCATCCTTTTTCGCACG
ATATACAGGATTTTGCCAAAGGGTTCGTGTAGACTTTCCTTGGTGTATCCAACGGCGTCAGCCGGGCAGGATAGGTGAAGTAGGCCCACCCGCGAGCGGGTGTTCCTTCTTCACTGTCCCTTAT
TCGCACCTGGCGGTGCTCAACGGGAATCCTGCTCTGCGAGGCTGGCCGTAGGCCGGCCCCGTAGAAAAGATCAAAGGATCTTCTTGAGATCCTTTTTTTCTGCGCGTAATCTGCTGCTTGCAAA
CAAAAAAACCACCGCTACCAGCGGTGGTTTGTTTGCCGGATCAAGAGCTACCAACTCTTTTTCCGAAGGTAACTGGCTTCAGCAGAGCGCAGATACCAAATACTGTCCTTCTAGTGTAGCCGTA
GTTAGGCCACCACTTCAAGAACTCTGTAGCACCGCCTACATACCTCGCTCTGCTAATCCTGTTACCAGTGGCTGCTGCCAGTGGCGATAAGTCGTGTCTTACCGGGTTGGACTCAAGACGATAGT
TACCGGATAAGGCGCAGCGGTCGGGCTGAACGGGGGGTTCGTGCACACAGCCCAGCTTGGAGCGAACGACCTACACCGAACTGAGATACCTACAGCGTGAGCTATGAGAAAGCGCCACGCT
TCCCGAAGGGAGAAAGGCGGACAGGTATCCGGTAAGCGGCAGGGTCGGAACAGGAGAGCGCACGAGGGAGCTTCCAGGGGGAAACGCCTGGTATCTTTATAGTCCTGTCGGGTTTCGCCAC
CTCTGACTTGAGCGTCGATTTTTGTGATGCTCGTCAGGGGGGCGGAGCCTATGGAAAAACGCCAGCAACGCGGCCTTTTTACGGTTCCTGGCCTTTTGCTGGCCTTTTGCTCACATGTTCTTTCC
TGCGTTATCCCCTGATTCTGTGGATAACCGTATTACCGCCTTTGAGTGAGCTGATACCGCTCGCCGCAGCCGAACGACCGAGCGCAGCGAGTCAGTGAGCGAGGAAGCGGAAGAGCGCCTGAT
GCGGTATTTTCTCCTTACGCATCTGTGCGGTATTTCACACCGCATATGGTGCACTCTCAGTACAATCTGCTCTGATGCCGCATAGTTAAGCCAGTATACACTCCGCTATCGCTACGTGACTGGGTC
ATGGCTGCGCCCCGACACCCGCCAACACCCGCTGACGCGCCCTGACGGGCTTGTCTGCTCCCGGCATCCGCTTACAGACAAGCTGTGACCGTCTCCGGGAGCTGCATGTGTCAGAGGTTTTCAC
CGTCATCACCGAAACGCGCGAGGCAGCTGCGGTAAAGCTCATCAGCGTGGTCGTGCAGCGATTCACAGATGTCTGCCTGTTCATCCGCGTCCAGCTCGTTGAGTTTCTCCAGAAGCGTTAATGT
CTGGCTTCTGATAAAGCGGGCCATGTTAAGGGCGGTTTTTTCCTGTTTGGTCACTGATGCCTCCGTGTAAGGGGGATTTCTGTTCATGGGGGTAATGATACCGATGAAACGAGAGAGGATGCT
CACGATACGGGTTACTGATGATGAACATGCCCGGTTACTGGAACGTTGTGAGGGTAAACAACTGGCGGTATGGATGCGGCGGGGGCGCGCCCAGCTGTCTAGGGCGGCGGATTTGTCCTACT
CAGGAGAGCGTTCACCGACAAACAACAGATAAAACGAAAGGCCCAGTCTTTCGACTGAGCCTTTCGTTTTATTTGATGCCT

pT7-FepA ΔC/ΔL NSEGS

TTAATTAAAGCGGATAACAATTTCACACAGGAGGCCGCCTAGGCCGCGGCCGCGCGTAATACGACTCACTATAGGGGAATTGTGAGCGGATAACAATTCCCCTCTAGAAATAATTTTGTTTAAC
TTTAAGAAGGAGATATACATATGAACAAGAAGATTCATTCCCTGGCCTTGTTGGTCAATCTGGGGATTTATGGGGTAGCGCAGGCAGGCGAGTGGCACGGCTCCTGGGACGCATATTTCAATG
CGCCAAATAGTGAAGGTAGCACCAAACGCACTAACTTTAGCCTGACCGGTCCGCTGGGCGACGAATTCAGCTTCCGTTTGTATGGCAACCTCGACAAAACCAATAGTGAAGGTAGCGGGGTAA
TCAACAAAGATATTAATGGCGTGGTGCGCTGGGATTTCGCGCCATTGCAATCGCTGGAACTGGAAGCAGGTTACAGCCGCCAGGGTAACCTGAATAGTGAAGGTAGCAACCGTCTGTATCGCC
AGAACTACGCGCTGACCTGGAACGGTGGCTGGGATAACGGCGTGACCACCAGCAACTGGGTGCAGTACGAACACACCCGTAACTCGCGTAATAGTGAAGGTAGCGTCGATATCGATCTTGAT
GACGTGATGCTGCACAGCGAAGTTAACCTGCCGATTGATTTCCTCGTTAACCAGACGCTGACGCTGGGTACGGAGTGGAATCAGCAACGGATGAAGAATAGTGAAGGTAGCAAAGCAGAAAT
TTTCTCGCTGTTTGCCGAAAACAACATGGAGCTGACTGACAGCACCATCGTAACGCCGGGGCTGCGTTTCGATCATCACAGTATTGTCGGCAATAACTGGAGCCCGGCGCTGAACATATCGCAA
GGTTTAGGCGATGACTTCACGCTGAAAATGGGCATCGCCCGTGCTTATAAAAATAGTGAAGGTAGCGAAACCAGCATCAACAAAGAGATTGGTCTGGAGTTCAAACGCGACGGGTGGCTGGC
GGGCGTCACCTGGTTCCGTAACGATTATCGCAATAAGATTGAAGCGAATAGTGAAGGTAGCCTCTATCAGTGGGATAACGTGCCGAAAGCGGTGGTTGAAGGTCTGGAAGGATCGTTAAACG
TACCGGTTAGCGAAACGGTGATGTGGACCAATAACATCACTTATATGCTGAAGAGTGAAAACAATAGTGAAGGTAGCTATACGTTGAACTCAACGCTGAGCTGGCAGGCACGGGAAGATTTG
TCGATGCAAACGACCTTCACCTGGTACAATAGTGAAGGTAGCTACAGCATTGTTGGCCTGAGCGCGACCTGGGATGTGACGAAGAATGTCAGTCTGACCGGCGGCGTGGACAATCTGTTCGAC
AATAGTGAAGGTAGCTGGTATATGAGCGTAAACACCCACTTCTGACCGCTGAGCAATAACTAGCATAACCCCTTGGGGCCTCTAAACGGGTCTTGAGGGGTTTTTTGTGCGGCCGCGTCGTGAC
TGGGAAAACCCTGGCGACTAGTCTTGGACTCCTGTTGATAGATCCAGTAATGACCTCAGAACTCCATCTGGATTTGTTCAGAACGCTCGGTTGCCGCCGGGCGTTTTTTATTGGTGAGAATCCA
GGGGTCCCCAATAATTACGATTTAAATTTGTGTCTCAAAATCTCTGATGTTACATTGCACAAGATAAAAATATATCATCATGAACAATAAAACTGTCTGCTTACATAAACAGTAATACAAGGGGT
GTTATGAGCCATATTCAGCGTGAAACGAGCTGTAGCCGTCCGCGTCTGAACAGCAACATGGATGCGGATCTGTATGGCTATAAATGGGCGCGTGATAACGTGGGTCAGAGCGGCGCGACCAT
TTATCGTCTGTATGGCAAACCGGATGCGCCGGAACTGTTTCTGAAACATGGCAAAGGCAGCGTGGCGAACGATGTGACCGATGAAATGGTGCGTCTGAACTGGCTGACCGAATTTATGCCGCT
GCCGACCATTAAACATTTTATTCGCACCCCGGATGATGCGTGGCTGCTGACCACCGCGATTCCGGGCAAAACCGCGTTTCAGGTGCTGGAAGAATATCCGGATAGCGGCGAAAACATTGTGGA
TGCGCTGGCCGTGTTTCTGCGTCGTCTGCATAGCATTCCGGTGTGCAACTGCCCGTTTAACAGCGATCGTGTGTTTCGTCTGGCCCAGGCGCAGAGCCGTATGAACAACGGCCTGGTGGATGCG
AGCGATTTTGATGATGAACGTAACGGCTGGCCGGTGGAACAGGTGTGGAAAGAAATGCATAAACTGCTGCCGTTTAGCCCGGATAGCGTGGTGACCCACGGCGATTTTAGCCTGGATAACCT
GATTTTCGATGAAGGCAAACTGATTGGCTGCATTGATGTGGGCCGTGTGGGCATTGCGGATCGTTATCAGGATCTGGCCATTCTGTGGAACTGCCTGGGCGAATTTAGCCCGAGCCTGCAAAA
ACGTCTGTTTCAGAAATATGGCATTGATAATCCGGATATGAACAAACTGCAATTTCATCTGATGCTGGATGAATTTTTCTAATAATTAATTGGACCGCGGTCCGCGCGTTGTCCTTTTCCGCTGCA
TAACCCTGCTTCGGGGTCATTATAGCGATTTTTTCGGTATATCCATCCTTTTTCGCACGATATACAGGATTTTGCCAAAGGGTTCGTGTAGACTTTCCTTGGTGTATCCAACGGCGTCAGCCGGGC
AGGATAGGTGAAGTAGGCCCACCCGCGAGCGGGTGTTCCTTCTTCACTGTCCCTTATTCGCACCTGGCGGTGCTCAACGGGAATCCTGCTCTGCGAGGCTGGCCGTAGGCCGGCCCCGTAGAA
AAGATCAAAGGATCTTCTTGAGATCCTTTTTTTCTGCGCGTAATCTGCTGCTTGCAAACAAAAAAACCACCGCTACCAGCGGTGGTTTGTTTGCCGGATCAAGAGCTACCAACTCTTTTTCCGAAG
GTAACTGGCTTCAGCAGAGCGCAGATACCAAATACTGTCCTTCTAGTGTAGCCGTAGTTAGGCCACCACTTCAAGAACTCTGTAGCACCGCCTACATACCTCGCTCTGCTAATCCTGTTACCAGT
GGCTGCTGCCAGTGGCGATAAGTCGTGTCTTACCGGGTTGGACTCAAGACGATAGTTACCGGATAAGGCGCAGCGGTCGGGCTGAACGGGGGGTTCGTGCACACAGCCCAGCTTGGAGCGA
ACGACCTACACCGAACTGAGATACCTACAGCGTGAGCTATGAGAAAGCGCCACGCTTCCCGAAGGGAGAAAGGCGGACAGGTATCCGGTAAGCGGCAGGGTCGGAACAGGAGAGCGCACG
AGGGAGCTTCCAGGGGGAAACGCCTGGTATCTTTATAGTCCTGTCGGGTTTCGCCACCTCTGACTTGAGCGTCGATTTTTGTGATGCTCGTCAGGGGGGCGGAGCCTATGGAAAAACGCCAGC
AACGCGGCCTTTTTACGGTTCCTGGCCTTTTGCTGGCCTTTTGCTCACATGTTCTTTCCTGCGTTATCCCCTGATTCTGTGGATAACCGTATTACCGCCTTTGAGTGAGCTGATACCGCTCGCCGCA
GCCGAACGACCGAGCGCAGCGAGTCAGTGAGCGAGGAAGCGGAAGAGCGCCTGATGCGGTATTTTCTCCTTACGCATCTGTGCGGTATTTCACACCGCATATGGTGCACTCTCAGTACAATCT
GCTCTGATGCCGCATAGTTAAGCCAGTATACACTCCGCTATCGCTACGTGACTGGGTCATGGCTGCGCCCCGACACCCGCCAACACCCGCTGACGCGCCCTGACGGGCTTGTCTGCTCCCGGCA
TCCGCTTACAGACAAGCTGTGACCGTCTCCGGGAGCTGCATGTGTCAGAGGTTTTCACCGTCATCACCGAAACGCGCGAGGCAGCTGCGGTAAAGCTCATCAGCGTGGTCGTGCAGCGATTCA
CAGATGTCTGCCTGTTCATCCGCGTCCAGCTCGTTGAGTTTCTCCAGAAGCGTTAATGTCTGGCTTCTGATAAAGCGGGCCATGTTAAGGGCGGTTTTTTCCTGTTTGGTCACTGATGCCTCCGT
GTAAGGGGGATTTCTGTTCATGGGGGTAATGATACCGATGAAACGAGAGAGGATGCTCACGATACGGGTTACTGATGATGAACATGCCCGGTTACTGGAACGTTGTGAGGGTAAACAACTGG
CGGTATGGATGCGGCGGGGGCGCGCCCAGCTGTCTAGGGCGGCGGATTTGTCCTACTCAGGAGAGCGTTCACCGACAAACAACAGATAAAACGAAAGGCCCAGTCTTTCGACTGAGCCTTTC
GTTTTATTTGATGCCT

pT7-OmpF

TTAATTAAAGGAATTTAATACGACTCACTATAGGGGAATTGTGAGCGGATAACAATTCCCCTCTAGAAATAATTTTGTTTAACTTTAAGAAGGAGATATACATATGATGAAGCGCAATATTCTGG
CAGTGATCGTCCCTGCTCTGTTAGTAGCAGGTACTGCAAACGCTGCAGAAATCTATAACAAAGATGGCAACAAAGTAGATCTGTACGGTAAAGCTGTCGGTCTGCATTATTTCTCCAAGGGTAA
CGGTGAAAACAGTTACGGTGGCAATGGCGACATGACCTATGCCCGTCTTGGTTTTAAAGGGGAAACTCAAATCAATTCCGATCTGACCGGTTATGGTCAGTGGGAATATAACTTCCAGGGTAA
CAACTCTGAAGGCGCTGACGCTCAAACTGGTAACAAAACGCGTCTGGCATTCGCGGGTCTTAAATACGCTGACGTTGGTTCTTTCGATTACGGCCGTAACTACGGTGTGGTTTATGATGCACTG
GGTTACACCGATATGCTGCCTGAGTTTGGCGGTGACACGGCATACAGCGATGACTTCTTCGTTGGTCGTGTTGGCGGCGTTGCTACCTATCGTAACTCCAACTTCTTTGGTCTGGTTGATGGCCT
GAACTTCGCTGTTCAGTACCTGGGTAAAAACGAGCGTGACACTGCACGCCGCTCTAACGGCGACGGTGTTGGCGGTTCTATCAGCTACGAATACGAAGGCTTTGGTATCGTTGGTGCTTATGGT
GCAGCTGACCGTACCAACCTGCAAGAAGCTCAACCTCTTGGCAACGGTAAAAAAGCTGAACAGTGGGCTACTGGTCTGAAGTACGACGCGAACAACATCTACCTGGCAGCGAACTACGGTGA
AACCCGTAACGCTACGCCGATCACTAATAAATTTACAAACACCAGCGGCTTCGCCAACAAAACGCAAGACGTTCTGTTAGTTGCGCAATACCAGTTCGATTTCGGTCTGCGTCCGTCCATCGCTT
ACACCAAATCTAAAGCGAAAGACGTAGAAGGTATCGGTGATGTTGATCTGGTGAACTACTTTGAAGTGGGTGCAACCTACTACTTCAACAAAAACATGTCCACCTATGTTGACTACATCATCAA
CCAGATCGATTCTGACAACAAACTGGGCGTAGGTTCAGACGACACCGTTGCTGTGGGTATCGTTTACCAGTTCTAACCGCTGAGCAATAACTAGCATAACCCCTTGGGGCCTCTAAACGGGTCT
TGAGGGGTTTTTTGAGCTTGTCGTGACTGGGAAAACCCTGGCGACTAGTCTTGGACTCCTGTTGATAGATCCAGTAATGACCTCAGAACTCCATCTGGATTTGTTCAGAACGCTCGGTTGCCGCC
GGGCGTTTTTTATTGGTGAGAATCCAGGGGTCCCCAATAATTACGATTTAAATTTGTGTCTCAAAATCTCTGATGTTACATTGCACAAGATAAAAATATATCATCATGAACAATAAAACTGTCTGC
TTACATAAACAGTAATACAAGGGGTGTTATGAGCCATATTCAGCGTGAAACGAGCTGTAGCCGTCCGCGTCTGAACAGCAACATGGATGCGGATCTGTATGGCTATAAATGGGCGCGTGATAA
CGTGGGTCAGAGCGGCGCGACCATTTATCGTCTGTATGGCAAACCGGATGCGCCGGAACTGTTTCTGAAACATGGCAAAGGCAGCGTGGCGAACGATGTGACCGATGAAATGGTGCGTCTGA
ACTGGCTGACCGAATTTATGCCGCTGCCGACCATTAAACATTTTATTCGCACCCCGGATGATGCGTGGCTGCTGACCACCGCGATTCCGGGCAAAACCGCGTTTCAGGTGCTGGAAGAATATCC
GGATAGCGGCGAAAACATTGTGGATGCGCTGGCCGTGTTTCTGCGTCGTCTGCATAGCATTCCGGTGTGCAACTGCCCGTTTAACAGCGATCGTGTGTTTCGTCTGGCCCAGGCGCAGAGCCG
TATGAACAACGGCCTGGTGGATGCGAGCGATTTTGATGATGAACGTAACGGCTGGCCGGTGGAACAGGTGTGGAAAGAAATGCATAAACTGCTGCCGTTTAGCCCGGATAGCGTGGTGACCC
ACGGCGATTTTAGCCTGGATAACCTGATTTTCGATGAAGGCAAACTGATTGGCTGCATTGATGTGGGCCGTGTGGGCATTGCGGATCGTTATCAGGATCTGGCCATTCTGTGGAACTGCCTGG
GCGAATTTAGCCCGAGCCTGCAAAAACGTCTGTTTCAGAAATATGGCATTGATAATCCGGATATGAACAAACTGCAATTTCATCTGATGCTGGATGAATTTTTCTAATAATTAATTGGACCGCGG
TCCGCGCGTTGTCCTTTTCCGCTGCATAACCCTGCTTCGGGGTCATTATAGCGATTTTTTCGGTATATCCATCCTTTTTCGCACGATATACAGGATTTTGCCAAAGGGTTCGTGTAGACTTTCCTTG
GTGTATCCAACGGCGTCAGCCGGGCAGGATAGGTGAAGTAGGCCCACCCGCGAGCGGGTGTTCCTTCTTCACTGTCCCTTATTCGCACCTGGCGGTGCTCAACGGGAATCCTGCTCTGCGAGG
CTGGCCGTAGGCCGGCCCCGTAGAAAAGATCAAAGGATCTTCTTGAGATCCTTTTTTTCTGCGCGTAATCTGCTGCTTGCAAACAAAAAAACCACCGCTACCAGCGGTGGTTTGTTTGCCGGATC
AAGAGCTACCAACTCTTTTTCCGAAGGTAACTGGCTTCAGCAGAGCGCAGATACCAAATACTGTCCTTCTAGTGTAGCCGTAGTTAGGCCACCACTTCAAGAACTCTGTAGCACCGCCTACATAC
CTCGCTCTGCTAATCCTGTTACCAGTGGCTGCTGCCAGTGGCGATAAGTCGTGTCTTACCGGGTTGGACTCAAGACGATAGTTACCGGATAAGGCGCAGCGGTCGGGCTGAACGGGGGGTTCG
TGCACACAGCCCAGCTTGGAGCGAACGACCTACACCGAACTGAGATACCTACAGCGTGAGCTATGAGAAAGCGCCACGCTTCCCGAAGGGAGAAAGGCGGACAGGTATCCGGTAAGCGGCA
GGGTCGGAACAGGAGAGCGCACGAGGGAGCTTCCAGGGGGAAACGCCTGGTATCTTTATAGTCCTGTCGGGTTTCGCCACCTCTGACTTGAGCGTCGATTTTTGTGATGCTCGTCAGGGGGG
CGGAGCCTATGGAAAAACGCCAGCAACGCGGCCTTTTTACGGTTCCTGGCCTTTTGCTGGCCTTTTGCTCACATGTTCTTTCCTGCGTTATCCCCTGATTCTGTGGATAACCGTATTACCGCCTTT
GAGTGAGCTGATACCGCTCGCCGCAGCCGAACGACCGAGCGCAGCGAGTCAGTGAGCGAGGAAGCGGAAGAGCGCCTGATGCGGTATTTTCTCCTTACGCATCTGTGCGGTATTTCACACCG
CATATGGTGCACTCTCAGTACAATCTGCTCTGATGCCGCATAGTTAAGCCAGTATACACTCCGCTATCGCTACGTGACTGGGTCATGGCTGCGCCCCGACACCCGCCAACACCCGCTGACGCGCC
CTGACGGGCTTGTCTGCTCCCGGCATCCGCTTACAGACAAGCTGTGACCGTCTCCGGGAGCTGCATGTGTCAGAGGTTTTCACCGTCATCACCGAAACGCGCGAGGCAGCTGCGGTAAAGCTC
ATCAGCGTGGTCGTGCAGCGATTCACAGATGTCTGCCTGTTCATCCGCGTCCAGCTCGTTGAGTTTCTCCAGAAGCGTTAATGTCTGGCTTCTGATAAAGCGGGCCATGTTAAGGGCGGTTTTTT
CCTGTTTGGTCACTGATGCCTCCGTGTAAGGGGGATTTCTGTTCATGGGGGTAATGATACCGATGAAACGAGAGAGGATGCTCACGATACGGGTTACTGATGATGAACATGCCCGGTTACTGG
AACGTTGTGAGGGTAAACAACTGGCGGTATGGATGCGGCGGGGGCGCGCCCAGCTGTCTAGGGCGGCGGATTTGTCCTACTCAGGAGAGCGTTCACCGACAAACAACAGATAAAACGAAAG
GCCCAGTCTTTCGACTGAGCCTTTCGTTTTATTTGATGCCT

pT7-OmpF Δ

AATTTAATACGACTCACTATAGGGGAATTGTGAGCGGATAACAATTCCCCTCTAGAAATAATTTTGTTTAACTTTAAGAAGGAGATATACATATGAAAAAGAGCACTCTGGCATTAGTGGTGAT
GGGCATTGTGGCATCTGCATCTGTACAGGCTGCAGAAATCTATAACAAAGATGGCAACAAAGTAGATCTGTACGGTAAAGCTGTTGGTCTGCATTATTTTTCCAAGGGTAACGGTGAAAACAG
TTACGGTGGCAATGGCGACATGACCTATGCCCGTCTTGGTTTTAAAGGGGAAACTCAAATCAATTCCGATCTGACCGGTTATGGTCAGTGGGAATATAACTTCCAGGGTAACAACTCTGAAGGC
GCTGACGCTCAAACTGGTAACAAAACGCGTCTGGCATTCGCGGGTCTTAAATACGCTGACGTTGGTTCTTTCGATTACGGCCGTAACTACGGTGTGGTTTATGATGCACTGGGTTACACCGATA
TGCTGCCAGAATTTGGTGGTGATACTGCATACAGCGATGACTTCTTCGTTGGTCGTGTTGGCGGCGTTGCTACCTATCGTAACTCCAACTTCTTTGGTCTGGTTGATGGCCTGAACTTCGCTGTTC
AGTACCTGGGTAAAAACGAGCGTGACACTGCACGCCGTTCTAACGGCGACGGTGTTGGCGGTTCTATCAGCTACGAATACGAAGGCTTTGGTATCGTTGGTGCTTATGGTGCAGCTGACCGTA
CCAACCTGCAAGAAGCTCAACCTCTTGGCAACGGTAAAAAAGCTGAACAGTGGGCTACTGGTCTGAAGTACGACGCGAACAACATCTACCTGGCAGCGAACTACGGTGAAACCCGTAACGCTA
CGCCGATCACTAATAAATTTACAAACACCAGCGGCTTCGCCAACAAAACGCAAGACGTTCTGTTAGTTGCGCAATACCAGTTCGATTTCGGTCTGCGTCCGTCCATCGCTTACACCAAATCTAAA
GCGAAAGACGTAGAAGGTATCGGTGATGTTGATCTGGTGAACTACTTTGAAGTGGGCGCAACCTACTACTTCAACAAAAACATGTCCACCTATGTTGACTACATCATCAACCAGATCGATTCTG
ACAACAAACTGGGCGTAGGTTCAGACGACACCGTTGCTGTGGGTATCGTTTACCAGTTCTAAAAGCTTGCGGCCGCACTCGAGCACCACCACCACCACCACTGAGATCCGGCTGCTAACAAAGC
CCGAAAGGAAGCTGAGTTGGCTGCTGCCACCGCTGAGCAATAACTAGCATAACCCCTTGGGGCCTCTAAACGGGTCTTGAGGGGTTTTTTGCTGAAAGGAGGAACTATATCCGACTAGTCTTG
GACTCCTGTTGATAGATCCAGTAATGACCTCAGAACTCCATCTGGATTTGTTCAGAACGCTCGGTTGCCGCCGGGCGTTTTTTATTGGTGAGAATCCAGGGGTCCCCAATAATTACGATTTAAAT
TTGTGTCTCAAAATCTCTGATGTTACATTGCACAAGATAAAAATATATCATCATGAACAATAAAACTGTCTGCTTACATAAACAGTAATACAAGGGGTGTTATGAGCCATATTCAGCGTGAAACG
AGCTGTAGCCGTCCGCGTCTGAACAGCAACATGGATGCGGATCTGTATGGCTATAAATGGGCGCGTGATAACGTGGGTCAGAGCGGCGCGACCATTTATCGTCTGTATGGCAAACCGGATGC
GCCGGAACTGTTTCTGAAACATGGCAAAGGCAGCGTGGCGAACGATGTGACCGATGAAATGGTGCGTCTGAACTGGCTGACCGAATTTATGCCGCTGCCGACCATTAAACATTTTATTCGCAC
CCCGGATGATGCGTGGCTGCTGACCACCGCGATTCCGGGCAAAACCGCGTTTCAGGTGCTGGAAGAATATCCGGATAGCGGCGAAAACATTGTGGATGCGCTGGCCGTGTTTCTGCGTCGTCT
GCATAGCATTCCGGTGTGCAACTGCCCGTTTAACAGCGATCGTGTGTTTCGTCTGGCCCAGGCGCAGAGCCGTATGAACAACGGCCTGGTGGATGCGAGCGATTTTGATGATGAACGTAACGG
CTGGCCGGTGGAACAGGTGTGGAAAGAAATGCATAAACTGCTGCCGTTTAGCCCGGATAGCGTGGTGACCCACGGCGATTTTAGCCTGGATAACCTGATTTTCGATGAAGGCAAACTGATTGG
CTGCATTGATGTGGGCCGTGTGGGCATTGCGGATCGTTATCAGGATCTGGCCATTCTGTGGAACTGCCTGGGCGAATTTAGCCCGAGCCTGCAAAAACGTCTGTTTCAGAAATATGGCATTGAT
AATCCGGATATGAACAAACTGCAATTTCATCTGATGCTGGATGAATTTTTCTAATAATTAATTGGACCGCGGTCCGCGCGTTGTCCTTTTCCGCTGCATAACCCTGCTTCGGGGTCATTATAGCGA
TTTTTTCGGTATATCCATCCTTTTTCGCACGATATACAGGATTTTGCCAAAGGGTTCGTGTAGACTTTCCTTGGTGTATCCAACGGCGTCAGCCGGGCAGGATAGGTGAAGTAGGCCCACCCGCG
AGCGGGTGTTCCTTCTTCACTGTCCCTTATTCGCACCTGGCGGTGCTCAACGGGAATCCTGCTCTGCGAGGCTGGCCGTAGGCCGGCCCCGTAGAAAAGATCAAAGGATCTTCTTGAGATCCTT
TTTTTCTGCGCGTAATCTGCTGCTTGCAAACAAAAAAACCACCGCTACCAGCGGTGGTTTGTTTGCCGGATCAAGAGCTACCAACTCTTTTTCCGAAGGTAACTGGCTTCAGCAGAGCGCAGATA
CCAAATACTGTCCTTCTAGTGTAGCCGTAGTTAGGCCACCACTTCAAGAACTCTGTAGCACCGCCTACATACCTCGCTCTGCTAATCCTGTTACCAGTGGCTGCTGCCAGTGGCGATAAGTCGTG
TCTTACCGGGTTGGACTCAAGACGATAGTTACCGGATAAGGCGCAGCGGTCGGGCTGAACGGGGGGTTCGTGCACACAGCCCAGCTTGGAGCGAACGACCTACACCGAACTGAGATACCTAC
AGCGTGAGCTATGAGAAAGCGCCACGCTTCCCGAAGGGAGAAAGGCGGACAGGTATCCGGTAAGCGGCAGGGTCGGAACAGGAGAGCGCACGAGGGAGCTTCCAGGGGGAAACGCCTGG
TATCTTTATAGTCCTGTCGGGTTTCGCCACCTCTGACTTGAGCGTCGATTTTTGTGATGCTCGTCAGGGGGGCGGAGCCTATGGAAAAACGCCAGCAACGCGGCCTTTTTACGGTTCCTGGCCTT
TTGCTGGCCTTTTGCTCACATGTTCTTTCCTGCGTTATCCCCTGATTCTGTGGATAACCGTATTACCGCCTTTGAGTGAGCTGATACCGCTCGCCGCAGCCGAACGACCGAGCGCAGCGAGTCAG
TGAGCGAGGAAGCGGAAGAGCGCCTGATGCGGTATTTTCTCCTTACGCATCTGTGCGGTATTTCACACCGCATATGGTGCACTCTCAGTACAATCTGCTCTGATGCCGCATAGTTAAGCCAGTA
TACACTCCGCTATCGCTACGTGACTGGGTCATGGCTGCGCCCCGACACCCGCCAACACCCGCTGACGCGCCCTGACGGGCTTGTCTGCTCCCGGCATCCGCTTACAGACAAGCTGTGACCGTCT
CCGGGAGCTGCATGTGTCAGAGGTTTTCACCGTCATCACCGAAACGCGCGAGGCAGCTGCGGTAAAGCTCATCAGCGTGGTCGTGCAGCGATTCACAGATGTCTGCCTGTTCATCCGCGTCCA
GCTCGTTGAGTTTCTCCAGAAGCGTTAATGTCTGGCTTCTGATAAAGCGGGCCATGTTAAGGGCGGTTTTTTCCTGTTTGGTCACTGATGCCTCCGTGTAAGGGGGATTTCTGTTCATGGGGGTA
ATGATACCGATGAAACGAGAGAGGATGCTCACGATACGGGTTACTGATGATGAACATGCCCGGTTACTGGAACGTTGTGAGGGTAAACAACTGGCGGTATGGATGCGGCGGGGGCGCGCCC
AGCTGTCTAGGGCGGCGGATTTGTCCTACTCAGGAGAGCGTTCACCGACAAACAACAGATAAAACGAAAGGCCCAGTCTTTCGACTGAGCCTTTCGTTTTATTTGATGCCTTTAATTAAAGG

pT7-OmpF Δ GIGL

AATTTAATACGACTCACTATAGGGGAATTGTGAGCGGATAACAATTCCCCTCTAGAAATAATTTTGTTTAACTTTAAGAAGGAGATATACATATGAAAAAGAGCACTCTGGCATTAGTGGTGAT
GGGCATTGTGGCATCTGCATCTGTACAGGCTGCAGAAATCTATAACAAAGATGGCAACAAAGTAGATCTGTACGGTAAAGCTGTTGGTCTGCATTATTTTTCCAAGGGTAACGGTGAAAACAG
TTACGGTGGCAATGGCGACATGACCTATGCCCGTCTTGGTTTTAAAGGGGAAACTCAAATCAATTCCGATCTGACCATCTATCTTCAGTGGGAATATAACTTCCAGGGTAACAACTCTGAAGGC
GCTGACGCTCAAACTGGTAACAAAACGCGTCTGGCATTCGCGGGTCTTAAATACGCTGACGTTGGTTCTTTCGATTACGGCCGTAACTACGGTGTGGTTTATGATGCACTGGGTTACACCGATA
TGCTGCCAGAATTTGGTGGTGATACTGCATACAGCGATGACTTCTTCGTTGGTCGTGTTGGCGGCGTTGCTACCTATCGTAACTCCAACTTCTTTGGTCTGGTTGATGGCCTGAACTTCGCTGTTC
AGTACCTGGGTAAAAACGAGCGTGACACTGCACGCCGTTCTAACGGCGACGGTGTTGGCGGTTCTATCAGCTACGAATACGAAGGCTTTGGTATCGTTGGTGCTTATGGTGCAGCTGACCGTA
CCAACCTGCAAGAAGCTCAACCTCTTGGCAACGGTAAAAAAGCTGAACAGTGGGCTACTGGTCTGAAGTACGACGCGAACAACATCTACCTGGCAGCGAACTACGGTGAAACCCGTAACGCTA
CGCCGATCACTAATAAATTTACAAACACCAGCGGCTTCGCCAACAAAACGCAAGACGTTCTGTTAGTTGCGCAATACCAGTTCGATTTCGGTCTGCGTCCGTCCATCGCTTACACCAAATCTAAA
GCGAAAGACGTAGAAGGTATCGGTGATGTTGATCTGGTGAACTACTTTGAAGTGGGCGCAACCTACTACTTCAACAAAAACATGTCCACCTATGTTGACTACATCATCAACCAGATCGATTCTG
ACAACAAACTGGGCGTAGGTTCAGACGACACCGTTGCTGTGGGTATCGTTTACCAGTTCTAAAAGCTTGCGGCCGCACTCGAGCACCACCACCACCACCACTGAGATCCGGCTGCTAACAAAGC
CCGAAAGGAAGCTGAGTTGGCTGCTGCCACCGCTGAGCAATAACTAGCATAACCCCTTGGGGCCTCTAAACGGGTCTTGAGGGGTTTTTTGCTGAAAGGAGGAACTATATCCGACTAGTCTTG
GACTCCTGTTGATAGATCCAGTAATGACCTCAGAACTCCATCTGGATTTGTTCAGAACGCTCGGTTGCCGCCGGGCGTTTTTTATTGGTGAGAATCCAGGGGTCCCCAATAATTACGATTTAAAT
TTGTGTCTCAAAATCTCTGATGTTACATTGCACAAGATAAAAATATATCATCATGAACAATAAAACTGTCTGCTTACATAAACAGTAATACAAGGGGTGTTATGAGCCATATTCAGCGTGAAACG
AGCTGTAGCCGTCCGCGTCTGAACAGCAACATGGATGCGGATCTGTATGGCTATAAATGGGCGCGTGATAACGTGGGTCAGAGCGGCGCGACCATTTATCGTCTGTATGGCAAACCGGATGC
GCCGGAACTGTTTCTGAAACATGGCAAAGGCAGCGTGGCGAACGATGTGACCGATGAAATGGTGCGTCTGAACTGGCTGACCGAATTTATGCCGCTGCCGACCATTAAACATTTTATTCGCAC
CCCGGATGATGCGTGGCTGCTGACCACCGCGATTCCGGGCAAAACCGCGTTTCAGGTGCTGGAAGAATATCCGGATAGCGGCGAAAACATTGTGGATGCGCTGGCCGTGTTTCTGCGTCGTCT
GCATAGCATTCCGGTGTGCAACTGCCCGTTTAACAGCGATCGTGTGTTTCGTCTGGCCCAGGCGCAGAGCCGTATGAACAACGGCCTGGTGGATGCGAGCGATTTTGATGATGAACGTAACGG
CTGGCCGGTGGAACAGGTGTGGAAAGAAATGCATAAACTGCTGCCGTTTAGCCCGGATAGCGTGGTGACCCACGGCGATTTTAGCCTGGATAACCTGATTTTCGATGAAGGCAAACTGATTGG
CTGCATTGATGTGGGCCGTGTGGGCATTGCGGATCGTTATCAGGATCTGGCCATTCTGTGGAACTGCCTGGGCGAATTTAGCCCGAGCCTGCAAAAACGTCTGTTTCAGAAATATGGCATTGAT
AATCCGGATATGAACAAACTGCAATTTCATCTGATGCTGGATGAATTTTTCTAATAATTAATTGGACCGCGGTCCGCGCGTTGTCCTTTTCCGCTGCATAACCCTGCTTCGGGGTCATTATAGCGA
TTTTTTCGGTATATCCATCCTTTTTCGCACGATATACAGGATTTTGCCAAAGGGTTCGTGTAGACTTTCCTTGGTGTATCCAACGGCGTCAGCCGGGCAGGATAGGTGAAGTAGGCCCACCCGCG
AGCGGGTGTTCCTTCTTCACTGTCCCTTATTCGCACCTGGCGGTGCTCAACGGGAATCCTGCTCTGCGAGGCTGGCCGTAGGCCGGCCCCGTAGAAAAGATCAAAGGATCTTCTTGAGATCCTT
TTTTTCTGCGCGTAATCTGCTGCTTGCAAACAAAAAAACCACCGCTACCAGCGGTGGTTTGTTTGCCGGATCAAGAGCTACCAACTCTTTTTCCGAAGGTAACTGGCTTCAGCAGAGCGCAGATA
CCAAATACTGTCCTTCTAGTGTAGCCGTAGTTAGGCCACCACTTCAAGAACTCTGTAGCACCGCCTACATACCTCGCTCTGCTAATCCTGTTACCAGTGGCTGCTGCCAGTGGCGATAAGTCGTG
TCTTACCGGGTTGGACTCAAGACGATAGTTACCGGATAAGGCGCAGCGGTCGGGCTGAACGGGGGGTTCGTGCACACAGCCCAGCTTGGAGCGAACGACCTACACCGAACTGAGATACCTAC
AGCGTGAGCTATGAGAAAGCGCCACGCTTCCCGAAGGGAGAAAGGCGGACAGGTATCCGGTAAGCGGCAGGGTCGGAACAGGAGAGCGCACGAGGGAGCTTCCAGGGGGAAACGCCTGG
TATCTTTATAGTCCTGTCGGGTTTCGCCACCTCTGACTTGAGCGTCGATTTTTGTGATGCTCGTCAGGGGGGCGGAGCCTATGGAAAAACGCCAGCAACGCGGCCTTTTTACGGTTCCTGGCCTT
TTGCTGGCCTTTTGCTCACATGTTCTTTCCTGCGTTATCCCCTGATTCTGTGGATAACCGTATTACCGCCTTTGAGTGAGCTGATACCGCTCGCCGCAGCCGAACGACCGAGCGCAGCGAGTCAG
TGAGCGAGGAAGCGGAAGAGCGCCTGATGCGGTATTTTCTCCTTACGCATCTGTGCGGTATTTCACACCGCATATGGTGCACTCTCAGTACAATCTGCTCTGATGCCGCATAGTTAAGCCAGTA
TACACTCCGCTATCGCTACGTGACTGGGTCATGGCTGCGCCCCGACACCCGCCAACACCCGCTGACGCGCCCTGACGGGCTTGTCTGCTCCCGGCATCCGCTTACAGACAAGCTGTGACCGTCT
CCGGGAGCTGCATGTGTCAGAGGTTTTCACCGTCATCACCGAAACGCGCGAGGCAGCTGCGGTAAAGCTCATCAGCGTGGTCGTGCAGCGATTCACAGATGTCTGCCTGTTCATCCGCGTCCA
GCTCGTTGAGTTTCTCCAGAAGCGTTAATGTCTGGCTTCTGATAAAGCGGGCCATGTTAAGGGCGGTTTTTTCCTGTTTGGTCACTGATGCCTCCGTGTAAGGGGGATTTCTGTTCATGGGGGTA
ATGATACCGATGAAACGAGAGAGGATGCTCACGATACGGGTTACTGATGATGAACATGCCCGGTTACTGGAACGTTGTGAGGGTAAACAACTGGCGGTATGGATGCGGCGGGGGCGCGCCC
AGCTGTCTAGGGCGGCGGATTTGTCCTACTCAGGAGAGCGTTCACCGACAAACAACAGATAAAACGAAAGGCCCAGTCTTTCGACTGAGCCTTTCGTTTTATTTGATGCCTTTAATTAAAGG

prhaBAD-FhuA ΔC

GTAACGAGAAGGTCGCGAATTCAGGCGCTTTTTAGACTGGTCGTAATGAACCTCTAGAAATAATTTTGTTTAACTTTAAGAAGGAGATATACATATGGCGCGTTCCAAAACTGCTCAGCCAAAA
CACTCACTGCGTAAAATCGCAGTTGTAGTAGCCACAGCGGTTAGCGGCATGTCTGTTTATGCACAGGCACTGAAAGAAGTTCAGTTTAAAGCCGGTACTGACAGCCTGTTCCAGACTGGTTTTG
ACTTTAGCGATTCGTTGGATGATGACGGTGTTTACTCTTATCGCCTGACCGGTCTTGCGCGTTCTGCCAATGCCCAGCAGAAAGGGTCAGAAGAGCAGCGTTATGCTATTGCACCGGCGTTCAC
CTGGCGTCCGGATGATAAAACCAATTTTACCTTCCTTTCTTACTTCCAGAACGAGCCGGAAACCGGTTATTACGGCTGGTTGCCGAAAGAGGGAACCGTTGAGCCGCTGCCGAACGGTAAGCGT
CTGCCGACAGACTTTAATGAAGGGGCGAAGAACAACACCTATTCTCGTAATGAGAAGATGGTCGGCTACAGCTTCGATCACGAATTTAACGACACCTTTACTGTGCGTCAGAACCTGCGCTTTG
CTGAAAACAAAACCTCGCAAAACAGCGTTTATGGTTACGGCGTCTGCTCCGATCCGGCGAATGCTTACAGCAAACAGTGTGCGGCATTAGCGCCAGCGGATAAAGGCCATTATCTGGCACGTA
AATACGTCGTTGATGATGAGAAGCTGCAAAACTTCTCCGTTGATACCCAGTTGCAGAGCAAGTTTGCCACTGGCGATATCGACCACACCCTGCTGACCGGTGTCGACTTTATGCGTATGCGTAA
TGACATCAACGCCTGGTTTGGTTACGACGACTCTGTGCCACTGCTCAATCTGTACAATCCGGTGAATACCGATTTCGACTTCAATGCCAAAGATCCGGCAAACTCCGGCCCTTACCGCATTCTGA
ATAAACAGAAACAAACGGGCGTTTATGTTCAGGATCAGGCGCAGTGGGATAAAGTGCTGGTCACCCTAGGCGGTCGTTATGACTGGGCAGATCAAGAATCTCTTAACCGCGTTGCCGGGACG
ACCGATAAACGTGATGACAAACAGTTTACCTGGCGTGGTGGTGTTAACTACCTGTTTGATAATGGTGTAACACCTTACTTCAGCTATAGCGAATCGTTTTTCCCTTCTTCGCAAGTTGGGAAGGA
TGGTAATATTTTCGCACCGTCTAAAGGTAAGCAGTATGAAGTCGGCGTGAAATATGTACCGGAAGATCGTCCGATTGTAGTTACTGGTGCCGTGTATAATCTCACTAAAACCAACAACCTGATG
GCGGACCCTGAGGGTTCCTTCTTCTCGGTTGAAGGTGGCGAGATCCGCGCACGTGGCGTAGAAATCGAAGCGAAAGCGGCGCTGTCGGCGAGTGTTAACGTAGTCGGTTCTTATACTTACACC
GATGCGGAATACACCACCGATACTACCTATAAAGGCAATACGCCTGCACAGGTGCCAAAACACATGGCTTCGTTGTGGGCTGACTACACCTTCTTTGACGGTCCGCTTTCAGGTCTGACGCTGG
GCACCGGTGGTCGTTATACTGGCTCCAGTTATGGTGATCCGGCTAACTCCTTTAAAGTGGGAAGTTATACGGTCGTGGATGCGTTAGTACGTTATGATCTGGCGCGAGTCGGCATGGCTGGCTC
CAACGTGGCGCTGCATGTTAACAACCTGTTCGATCGTGAATACGTCGCCAGCTGCTTTAACACTTATGGCTGCTTCTGGGGCGCAGAACGTCAGGTCGTTGCAACCGCAACCTTCCGTTTCTAAA
TGTCCAGACCTGCAGGCATGCAAGCTCTAGAGGCATCAAATAAAACGAAAGGCTCAGTCGAAAGACTGGGCCTTTCGTTTTATCTGTTGTTTGTCGGTGAACGCTCTCCTGAGTAGGACAAATC
CGCCGCCCTAGACCTAGGGTACGGGTTTTGCTGCCCGCAAACGGGCTGTTCTGGTGTTGCTAGTTTGTTATCAGAATCGCAGATCCGGCTTCAGCCGGTTTGCCGGCTGAAAGCGCTATTTCTTC
CAGAATTGCCATGATTTTTTCCCCACGGGAGGCGTCACTGGCTCCCGTGTTGTCGGCAGCTTTGATTCGATAAGCAGCATCGCCTGTTTCAGGCTGTCTATGTGTGACTGTTGAGCTGTAACAAG
TTGTCTCAGGTGTTCAATTTCATGTTCTAGTTGCTTTGTTTTACTGGTTTCACCTGTTCTATTAGGTGTTACATGCTGTTCATCTGTTACATTGTCGATCTGTTCATGGTGAACAGCTTTGAATGCAC
CAAAAACTCGTAAAAGCTCTGATGTATCTATCTTTTTTACACCGTTTTCATCTGTGCATATGGACAGTTTTCCCTTTGATATGTAACGGTGAACAGTTGTTCTACTTTTGTTTGTTAGTCTTGATGCT
TCACTGATAGATACAAGAGCCATAAGAACCTCAGATCCTTCCGTATTTAGCCAGTATGTTCTCTAGTGTGGTTCGTTGTTTTTGCGTGAGCCATGAGAACGAACCATTGAGATCATACTTACTTTG
CATGTCACTCAAAAATTTTGCCTCAAAACTGGTGAGCTGAATTTTTGCAGTTAAAGCATCGTGTAGTGTTTTTCTTAGTCCGTTATGTAGGTAGGAATCTGATGTAATGGTTGTTGGTATTTTGTC
ACCATTCATTTTTATCTGGTTGTTCTCAAGTTCGGTTACGAGATCCATTTGTCTATCTAGTTCAACTTGGAAAATCAACGTATCAGTCGGGCGGCCTCGCTTATCAACCACCAATTTCATATTGCTG
TAAGTGTTTAAATCTTTACTTATTGGTTTCAAAACCCATTGGTTAAGCCTTTTAAACTCATGGTAGTTATTTTCAAGCATTAACATGAACTTAAATTCATCAAGGCTAATCTCTATATTTGCCTTGTG
AGTTTTCTTTTGTGTTAGTTCTTTTAATAACCACTCATAAATCCTCATAGAGTATTTGTTTTCAAAAGACTTAACATGTTCCAGATTATATTTTATGAATTTTTTTAACTGGAAAAGATAAGGCAATA
TCTCTTCACTAAAAACTAATTCTAATTTTTCGCTTGAGAACTTGGCATAGTTTGTCCACTGGAAAATCTCAAAGCCTTTAACCAAAGGATTCCTGATTTCCACAGTTCTCGTCATCAGCTCTCTGGT
TGCTTTAGCTAATACACCATAAGCATTTTCCCTACTGATGTTCATCATCTGAGCGTATTGGTTATAAGTGAACGATACCGTCCGTTCTTTCCTTGTAGGGTTTTCAATCGTGGGGTTGAGTAGTGC
CACACAGCATAAAATTAGCTTGGTTTCATGCTCCGTTAAGTCATAGCGACTAATCGCTAGTTCATTTGCTTTGAAAACAACTAATTCAGACATACATCTCAATTGGTCTAGGTGATTTTAATCACT
ATACCAATTGAGATGGGCTAGTCAATGATAATTACTAGTCCTTTTCCTTTGAGTTGTGGGTATCTGTAAATTCTGCTAGACCTTTGCTGGAAAACTTGTAAATTCTGCTAGACCCTCTGTAAATTC
CGCTAGACCTTTGTGTGTTTTTTTTGTTTATATTCAAGTGGTTATAATTTATAGAATAAAGAAAGAATAAAAAAAGATAAAAAGAATAGATCCCAGCCCTGTGTATAACTCACTACTTTAGTCAGT
TCCGCAGTATTACAAAAGGATGTCGCAAACGCTGTTTGCTCCTCTACAAAACAGACCTTAAAACCCTAAAGGCTTAAGTAGCGCCGTCGCAAGCTCCGGCAAATCGCTGAATATTCCTTTTGTCT
CCGACCATCAGGCACCTGAGTCGCTGTCTTTTTCGTCACATTCAGTTCGCTGCGCTCACGGCTCTGGCAGTGAATGGGGGTAAATGGCACTACAGGCGCCTTTTATGGATTCATGCAAGGAAAC
TACCCATAATACAAGAAAAGCCCGTCACGGGCTTCTCAGGGCGTTTTATGGCGGGTCTGCTATGTGGTGCTATCTGACTTTTTGCTGTTCAGCAGTTCCTGCCCTCTGATTTTCCAGTCTGACCAC
TTCGGATTATCCCGTGACAGGTCATTCAGACTGGCTAATGCACCCAGTAAGGCAGCGGTATCATCAACAGGCTTACCCGTCTTACTGTCCCTAGTGCTTGGATTCTCACCAATAAAAAACGCCCG
GCGGCAACCGAGCGTTCTGAACAAATCCAGATGGAGTTCTGAGGTCATTACTGGATCTATCAACAGGAGTCCAAGCGAGCTCTCGAACCCCAGAGTCCCGCTATGTATCCGCTCATGAATTAAT
TCTTATTACGCCCCGCCCTGCCACTCATCGCAGTACTGTTGTAATTCATTAAGCATTCTGCCGACATGGAAGCCATCACAAACGGCATGATGAACCTGAATCGCCAGCGGCATCAGCACCTTGTC
GCCTTGCGTATAATATTTGCCCATGGTGAAAACGGGGGCGAAGAAGTTGTCCATATTGGCCACGTTTAAATCAAAACTGGTGAAACTCACCCAGGGATTGGCTGAGACGAAAAACATATTCTC
AATAAACCCTTTAGGGAAATAGGCCAGGTTTTCACCGTAACACGCCACATCTTGCGAATATATGTGTAGAAACTGCCGGAAATCGTCGTGGTATTCACTCCAGAGCGATGAAAACGTTTCAGTT
TGCTCATGGAAAACGGTGTAACAAGGGTGAACACTATCCCATATCACCAGCTCACCGTCTTTCATTGCCATACGAAATTCCGGATGAGCATTCATCAGGCGGGCAAGAATGTGAATAAAGGCC
GGATAAAACTTGTGCTTATTTTTCTTTACGGTCTTTAAAAAGGCCGTAATATCCAGCTGAACGGTCTGGTTATAGGTACATTGAGCAACTGACTGAAATGCCTCAAAATGTTCTTTACGATGCCAT
TGGGATATATCAACGGTGGTATATCCAGTGATTTTTTTCTCCATTTTAGCTTCCTTAGCTCCTGAAAATCTCGATAACTCAAAAAATACGCCCGGTAGTGATCTTATTTCATTATGGTGAAAGTTG
GAACCTCTTACGTGCCGATCAAGATCAAAGGATCTTCTTGAGATCCCCAGCTGGCAATTCCGACGTCTAAGAAACCATTATTATCATGACATTAACCTATAAAAATAGGCGTATCACGAGGCCCT
TTCGTCTTCACCTCGAGGGGATCCCACCACAATTCAGCAAATTGTGAACATCATCACGTTCATCTTTCCCTGGTTGCCAATGGCCCATTTTCCTGTCA

prhaBAD-FhuA ΔC/Δ5L

GTAACGAGAAGGTCGCGAATTCAGGCGCTTTTTAGACTGGTCGTAATGAACCTCTAGAAATAATTTTGTTTAACTTTAAGAAGGAGATATACATATGGCTCGCTCCAAAACGGCACAGCCTAAA
CATAGCCTGCGTAAAATTGCAGTTGTTGTTGCAACCGCAGTTAGCGGTATGAGCGTTTATGCACAGGCACTGAAAGAAGTTCAGTTTAAAGCAGGCACCGATAGCCTGTTTCAGACCGGTTTTG
ATTTTAGCGATAGCCTGGATGATGATGGTGTTTATAGCTATCGTCTGACAGGTCTGGCTCGGAGTGCAAATGCACAGCAGAAAGGTAGCGAAGAACAGCGTTATGCAATTGCACCGGCATTTA
CCTGGCGTCCGGATGATAAAACCAATTTTACCTTTCTGAGCTATTTTCAGAATGAACCGGAAACCGGTAATAGCGAAGGTAGCACCTATAGCCGTAATGAAAAAATGGTGGGCTATAGCTTTGA
TCATGAGTTTAATGATACCTTTACCGTTCGTCAGAATCTGCGTTTTGCCGAAAATAAAACCAGCCAGAATAGCGTTTATGGTAATTCTGAAGGCAGCCGTAAATATGTTGTGGATGATGAAAAAC
TGCAGAATTTTAGCGTTGATACCCAGCTGCAGAGCAAATTTGCAACCGGTGATATTGATCATACCCTGCTGACCGGTGTGGATTTTATGCGTATGCGCAATGATATTAATGCCTGGTTTGGCTAT
AATTCAGAAGGTAGCAGCGGTCCGTATCGTATTCTGAATAAACAGAAACAGACCGGTGTTTATGTTCAGGATCAGGCACAGTGGGATAAAGTTCTGGTTACCCTGGGTGGTCGTTATGATTGG
GCAGATCAGGAAAGCCTGAATCGCGTTGCAGGCACCACCGATAAACGTGATGATAAACAGTTTACATGGCGTGGTGGTGTGAATTACCTGTTTGATAATGGCGTGACCCCGTATTTTAGCTATA
GCGAAAGCTTTGAACCGAGCAGCCAGGTGGGTAAAGATGGCAATATTTTTGCACCGAGCAAAGGCAAACAGTATGAAGTGGGCGTTAAATATGTTCCGGAAGATCGTCCGATTGTGGTTACC
GGTGCCGTTTATAATCTGACCAAAACCAATAATCTGATGGCAGATCCGGAAGGTAGCTTTTTTAGCGTGGAAGGTGGTGAAATTCGTGCACGTGGTGTTGAAATTGAAGCAAAAGCAGCACTG
AGCGCAAGCGTTAATGTTGTTGGCAGCTATACCTATACCGATGCAGAATATACCACCGATACCACCTATAAAGGTAATACACCGGCACAGGTTCCGAAACATATGGCAAGCCTGTGGGCAGATT
ATACCTTTTTTGATGGTCCGCTGTCTGGTCTGACCCTGGGCACCGGTGGTCGCTATACCAATTCTGAAGGTAGCTATACCGTTGTGGATGCACTGGTTCGTTATGATCTGGCTCGTGTTGGTATG
GCAGGTAGCAATGTTGCACTGCATGTGAATAGTGAAGGTAGCCAGGTTGTTGCCACCGCAACCTTTCGTTTTTAAATGTCCAGACCTGCAGGCATGCAAGCTCTAGAGGCATCAAATAAAACGA
AAGGCTCAGTCGAAAGACTGGGCCTTTCGTTTTATCTGTTGTTTGTCGGTGAACGCTCTCCTGAGTAGGACAAATCCGCCGCCCTAGACCTAGGGTACGGGTTTTGCTGCCCGCAAACGGGCTG
TTCTGGTGTTGCTAGTTTGTTATCAGAATCGCAGATCCGGCTTCAGCCGGTTTGCCGGCTGAAAGCGCTATTTCTTCCAGAATTGCCATGATTTTTTCCCCACGGGAGGCGTCACTGGCTCCCGT
GTTGTCGGCAGCTTTGATTCGATAAGCAGCATCGCCTGTTTCAGGCTGTCTATGTGTGACTGTTGAGCTGTAACAAGTTGTCTCAGGTGTTCAATTTCATGTTCTAGTTGCTTTGTTTTACTGGTTT
CACCTGTTCTATTAGGTGTTACATGCTGTTCATCTGTTACATTGTCGATCTGTTCATGGTGAACAGCTTTGAATGCACCAAAAACTCGTAAAAGCTCTGATGTATCTATCTTTTTTACACCGTTTTC
ATCTGTGCATATGGACAGTTTTCCCTTTGATATGTAACGGTGAACAGTTGTTCTACTTTTGTTTGTTAGTCTTGATGCTTCACTGATAGATACAAGAGCCATAAGAACCTCAGATCCTTCCGTATTT
AGCCAGTATGTTCTCTAGTGTGGTTCGTTGTTTTTGCGTGAGCCATGAGAACGAACCATTGAGATCATACTTACTTTGCATGTCACTCAAAAATTTTGCCTCAAAACTGGTGAGCTGAATTTTTGC
AGTTAAAGCATCGTGTAGTGTTTTTCTTAGTCCGTTATGTAGGTAGGAATCTGATGTAATGGTTGTTGGTATTTTGTCACCATTCATTTTTATCTGGTTGTTCTCAAGTTCGGTTACGAGATCCATT
TGTCTATCTAGTTCAACTTGGAAAATCAACGTATCAGTCGGGCGGCCTCGCTTATCAACCACCAATTTCATATTGCTGTAAGTGTTTAAATCTTTACTTATTGGTTTCAAAACCCATTGGTTAAGCC
TTTTAAACTCATGGTAGTTATTTTCAAGCATTAACATGAACTTAAATTCATCAAGGCTAATCTCTATATTTGCCTTGTGAGTTTTCTTTTGTGTTAGTTCTTTTAATAACCACTCATAAATCCTCATAG
AGTATTTGTTTTCAAAAGACTTAACATGTTCCAGATTATATTTTATGAATTTTTTTAACTGGAAAAGATAAGGCAATATCTCTTCACTAAAAACTAATTCTAATTTTTCGCTTGAGAACTTGGCATA
GTTTGTCCACTGGAAAATCTCAAAGCCTTTAACCAAAGGATTCCTGATTTCCACAGTTCTCGTCATCAGCTCTCTGGTTGCTTTAGCTAATACACCATAAGCATTTTCCCTACTGATGTTCATCATC
TGAGCGTATTGGTTATAAGTGAACGATACCGTCCGTTCTTTCCTTGTAGGGTTTTCAATCGTGGGGTTGAGTAGTGCCACACAGCATAAAATTAGCTTGGTTTCATGCTCCGTTAAGTCATAGCG
ACTAATCGCTAGTTCATTTGCTTTGAAAACAACTAATTCAGACATACATCTCAATTGGTCTAGGTGATTTTAATCACTATACCAATTGAGATGGGCTAGTCAATGATAATTACTAGTCCTTTTCCTT
TGAGTTGTGGGTATCTGTAAATTCTGCTAGACCTTTGCTGGAAAACTTGTAAATTCTGCTAGACCCTCTGTAAATTCCGCTAGACCTTTGTGTGTTTTTTTTGTTTATATTCAAGTGGTTATAATTT
ATAGAATAAAGAAAGAATAAAAAAAGATAAAAAGAATAGATCCCAGCCCTGTGTATAACTCACTACTTTAGTCAGTTCCGCAGTATTACAAAAGGATGTCGCAAACGCTGTTTGCTCCTCTACA
AAACAGACCTTAAAACCCTAAAGGCTTAAGTAGCGCCGTCGCAAGCTCCGGCAAATCGCTGAATATTCCTTTTGTCTCCGACCATCAGGCACCTGAGTCGCTGTCTTTTTCGTCACATTCAGTTCG
CTGCGCTCACGGCTCTGGCAGTGAATGGGGGTAAATGGCACTACAGGCGCCTTTTATGGATTCATGCAAGGAAACTACCCATAATACAAGAAAAGCCCGTCACGGGCTTCTCAGGGCGTTTTA
TGGCGGGTCTGCTATGTGGTGCTATCTGACTTTTTGCTGTTCAGCAGTTCCTGCCCTCTGATTTTCCAGTCTGACCACTTCGGATTATCCCGTGACAGGTCATTCAGACTGGCTAATGCACCCAGT
AAGGCAGCGGTATCATCAACAGGCTTACCCGTCTTACTGTCCCTAGTGCTTGGATTCTCACCAATAAAAAACGCCCGGCGGCAACCGAGCGTTCTGAACAAATCCAGATGGAGTTCTGAGGTCA
TTACTGGATCTATCAACAGGAGTCCAAGCGAGCTCTCGAACCCCAGAGTCCCGCTATGTATCCGCTCATGAATTAATTCTTATTACGCCCCGCCCTGCCACTCATCGCAGTACTGTTGTAATTCAT
TAAGCATTCTGCCGACATGGAAGCCATCACAAACGGCATGATGAACCTGAATCGCCAGCGGCATCAGCACCTTGTCGCCTTGCGTATAATATTTGCCCATGGTGAAAACGGGGGCGAAGAAGT
TGTCCATATTGGCCACGTTTAAATCAAAACTGGTGAAACTCACCCAGGGATTGGCTGAGACGAAAAACATATTCTCAATAAACCCTTTAGGGAAATAGGCCAGGTTTTCACCGTAACACGCCAC
ATCTTGCGAATATATGTGTAGAAACTGCCGGAAATCGTCGTGGTATTCACTCCAGAGCGATGAAAACGTTTCAGTTTGCTCATGGAAAACGGTGTAACAAGGGTGAACACTATCCCATATCACC
AGCTCACCGTCTTTCATTGCCATACGAAATTCCGGATGAGCATTCATCAGGCGGGCAAGAATGTGAATAAAGGCCGGATAAAACTTGTGCTTATTTTTCTTTACGGTCTTTAAAAAGGCCGTAAT
ATCCAGCTGAACGGTCTGGTTATAGGTACATTGAGCAACTGACTGAAATGCCTCAAAATGTTCTTTACGATGCCATTGGGATATATCAACGGTGGTATATCCAGTGATTTTTTTCTCCATTTTAGC
TTCCTTAGCTCCTGAAAATCTCGATAACTCAAAAAATACGCCCGGTAGTGATCTTATTTCATTATGGTGAAAGTTGGAACCTCTTACGTGCCGATCAAGATCAAAGGATCTTCTTGAGATCCCCAG
CTGGCAATTCCGACGTCTAAGAAACCATTATTATCATGACATTAACCTATAAAAATAGGCGTATCACGAGGCCCTTTCGTCTTCACCTCGAGGGGATCCCACCACAATTCAGCAAATTGTGAACA
TCATCACGTTCATCTTTCCCTGGTTGCCAATGGCCCATTTTCCTGTCA

prhaBAD-FepA ΔC/Δ4L

GTAACGAGAAGGTCGCGAATTCAGGCGCTTTTTAGACTGGTCGTAATGAACCTCTAGAAATAATTTTGTTTAACTTTAAGAAGGAGATATACATATGAACAAGAAGATTCATTCCCTGGCCTTGT
TGGTCAATCTGGGGATTTATGGGGTAGCGCAGGCAGGCGAGTGGCACGGCTCCTGGGACGCATATTTCAATGCGCCAGAACATAAAGAGGAAGGTGCCACCAAACGCACTAACTTTAGCCTG
ACCGGTCCGCTGGGCGACGAATTCAGCTTCCGTTTGTATGGCAACCTCGACAAAACCCAGCGCGAAGGGGTAATCAACAAAGATATTAATGGCGTGGTGCGCTGGGATTTCGCGCCATTGCAA
TCGCTGGAACTGGAAGCAGGTTACAGCCGCCAGGGTAACCTGTATGCGGGCGACACCCAGAATACCAACTCCGATTCCTATACCCGCTCGAAATATGGCGATGAAACCAACCGTCTGTATCGC
CAGAACTACGCGCTGACCTGGAACGGTGGCTGGGATAACGGCGTGACCACCAGCAACTGGGTGCAGTACGAACACACCCGTAACTCGCGTATTCCGGAAGGTCTGGCGGGCGGTACCGAAG
GGAAATTTAACGAAAAAGCGACACAGGATTTCGTCGATATCGATCTTGATGACGTGATGCTGCACAGCGAAGTTAACCTGCCGATTGATTTCCTCGTTAACCAGACGCTGACGCTGGGTACGG
AGTGGAATCAGCAACGGATGAAGGACTTATATTCAAAAGCAGAAATTTTCTCGCTGTTTGCCGAAAACAACATGGAGCTGACTGACAGCACCATCGTAACGCCGGGGCTGCGTTTCGATCATC
ACAGTATTGTCGGCAATAACTGGAGCCCGGCGCTGAACATATCGCAAGGTTTAGGCGATGACTTCACGCTGAAAATGGGCATCGCCCGTGCTTATAAAGCGCCGAAAGCAGAAACCAGCATCA
ACAAAGAGATTGGTCTGGAGTTCAAACGCGACGGGTGGCTGGCGGGCGTCACCTGGTTCCGTAACGATTATCGCAATAAGATTGAAGCGGGCTATGTGGCTGTAGGGCAAAACGCAGTCGGC
ACCGATCTCTATCAGTGGGATAACGTGCCGAAAGCGGTGGTTGAAGGTCTGGAAGGATCGTTAAACGTACCGGTTAGCGAAACGGTGATGTGGACCAATAACATCACTTATATGCTGAAGAGT
GAAAACAAAACCACGGGCGACCGTTTGTCGATCATCCCGGAGTATACGTTGAACTCAACGCTGAGCTGGCAGGCACGGGAAGATTTGTCGATGCAAACGACCTTCACCTGGTACGGCAAGCA
GCAGCCGAAGAAGTACAACTATAAAGGTCAGCCAGCGGTTGGACCGGAAACCAAAGAAATTAGTCCTTACAGCATTGTTGGCCTGAGCGCGACCTGGGATGTGACGAAGAATGTCAGTCTGA
CCGGCGGCGTGGACAATCTGTTCGACAAACGTGGACGTACGTGGTATATGAGCGTAAACACCCACTTCTGAATGTCCAGACCTGCAGGCATGCAAGCTCTAGAGGCATCAAATAAAACGAAAG
GCTCAGTCGAAAGACTGGGCCTTTCGTTTTATCTGTTGTTTGTCGGTGAACGCTCTCCTGAGTAGGACAAATCCGCCGCCCTAGACCTAGGGTACGGGTTTTGCTGCCCGCAAACGGGCTGTTCT
GGTGTTGCTAGTTTGTTATCAGAATCGCAGATCCGGCTTCAGCCGGTTTGCCGGCTGAAAGCGCTATTTCTTCCAGAATTGCCATGATTTTTTCCCCACGGGAGGCGTCACTGGCTCCCGTGTTG
TCGGCAGCTTTGATTCGATAAGCAGCATCGCCTGTTTCAGGCTGTCTATGTGTGACTGTTGAGCTGTAACAAGTTGTCTCAGGTGTTCAATTTCATGTTCTAGTTGCTTTGTTTTACTGGTTTCACC
TGTTCTATTAGGTGTTACATGCTGTTCATCTGTTACATTGTCGATCTGTTCATGGTGAACAGCTTTGAATGCACCAAAAACTCGTAAAAGCTCTGATGTATCTATCTTTTTTACACCGTTTTCATCTG
TGCATATGGACAGTTTTCCCTTTGATATGTAACGGTGAACAGTTGTTCTACTTTTGTTTGTTAGTCTTGATGCTTCACTGATAGATACAAGAGCCATAAGAACCTCAGATCCTTCCGTATTTAGCCA
GTATGTTCTCTAGTGTGGTTCGTTGTTTTTGCGTGAGCCATGAGAACGAACCATTGAGATCATACTTACTTTGCATGTCACTCAAAAATTTTGCCTCAAAACTGGTGAGCTGAATTTTTGCAGTTA
AAGCATCGTGTAGTGTTTTTCTTAGTCCGTTATGTAGGTAGGAATCTGATGTAATGGTTGTTGGTATTTTGTCACCATTCATTTTTATCTGGTTGTTCTCAAGTTCGGTTACGAGATCCATTTGTCT
ATCTAGTTCAACTTGGAAAATCAACGTATCAGTCGGGCGGCCTCGCTTATCAACCACCAATTTCATATTGCTGTAAGTGTTTAAATCTTTACTTATTGGTTTCAAAACCCATTGGTTAAGCCTTTTA
AACTCATGGTAGTTATTTTCAAGCATTAACATGAACTTAAATTCATCAAGGCTAATCTCTATATTTGCCTTGTGAGTTTTCTTTTGTGTTAGTTCTTTTAATAACCACTCATAAATCCTCATAGAGTA
TTTGTTTTCAAAAGACTTAACATGTTCCAGATTATATTTTATGAATTTTTTTAACTGGAAAAGATAAGGCAATATCTCTTCACTAAAAACTAATTCTAATTTTTCGCTTGAGAACTTGGCATAGTTTG
TCCACTGGAAAATCTCAAAGCCTTTAACCAAAGGATTCCTGATTTCCACAGTTCTCGTCATCAGCTCTCTGGTTGCTTTAGCTAATACACCATAAGCATTTTCCCTACTGATGTTCATCATCTGAGC
GTATTGGTTATAAGTGAACGATACCGTCCGTTCTTTCCTTGTAGGGTTTTCAATCGTGGGGTTGAGTAGTGCCACACAGCATAAAATTAGCTTGGTTTCATGCTCCGTTAAGTCATAGCGACTAA
TCGCTAGTTCATTTGCTTTGAAAACAACTAATTCAGACATACATCTCAATTGGTCTAGGTGATTTTAATCACTATACCAATTGAGATGGGCTAGTCAATGATAATTACTAGTCCTTTTCCTTTGAGT
TGTGGGTATCTGTAAATTCTGCTAGACCTTTGCTGGAAAACTTGTAAATTCTGCTAGACCCTCTGTAAATTCCGCTAGACCTTTGTGTGTTTTTTTTGTTTATATTCAAGTGGTTATAATTTATAGA
ATAAAGAAAGAATAAAAAAAGATAAAAAGAATAGATCCCAGCCCTGTGTATAACTCACTACTTTAGTCAGTTCCGCAGTATTACAAAAGGATGTCGCAAACGCTGTTTGCTCCTCTACAAAACA
GACCTTAAAACCCTAAAGGCTTAAGTAGCGCCGTCGCAAGCTCCGGCAAATCGCTGAATATTCCTTTTGTCTCCGACCATCAGGCACCTGAGTCGCTGTCTTTTTCGTCACATTCAGTTCGCTGCG
CTCACGGCTCTGGCAGTGAATGGGGGTAAATGGCACTACAGGCGCCTTTTATGGATTCATGCAAGGAAACTACCCATAATACAAGAAAAGCCCGTCACGGGCTTCTCAGGGCGTTTTATGGCG
GGTCTGCTATGTGGTGCTATCTGACTTTTTGCTGTTCAGCAGTTCCTGCCCTCTGATTTTCCAGTCTGACCACTTCGGATTATCCCGTGACAGGTCATTCAGACTGGCTAATGCACCCAGTAAGGC
AGCGGTATCATCAACAGGCTTACCCGTCTTACTGTCCCTAGTGCTTGGATTCTCACCAATAAAAAACGCCCGGCGGCAACCGAGCGTTCTGAACAAATCCAGATGGAGTTCTGAGGTCATTACT
GGATCTATCAACAGGAGTCCAAGCGAGCTCTCGAACCCCAGAGTCCCGCTATGTATCCGCTCATGAATTAATTCTTATTACGCCCCGCCCTGCCACTCATCGCAGTACTGTTGTAATTCATTAAGC
ATTCTGCCGACATGGAAGCCATCACAAACGGCATGATGAACCTGAATCGCCAGCGGCATCAGCACCTTGTCGCCTTGCGTATAATATTTGCCCATGGTGAAAACGGGGGCGAAGAAGTTGTCC
ATATTGGCCACGTTTAAATCAAAACTGGTGAAACTCACCCAGGGATTGGCTGAGACGAAAAACATATTCTCAATAAACCCTTTAGGGAAATAGGCCAGGTTTTCACCGTAACACGCCACATCTT
GCGAATATATGTGTAGAAACTGCCGGAAATCGTCGTGGTATTCACTCCAGAGCGATGAAAACGTTTCAGTTTGCTCATGGAAAACGGTGTAACAAGGGTGAACACTATCCCATATCACCAGCTC
ACCGTCTTTCATTGCCATACGAAATTCCGGATGAGCATTCATCAGGCGGGCAAGAATGTGAATAAAGGCCGGATAAAACTTGTGCTTATTTTTCTTTACGGTCTTTAAAAAGGCCGTAATATCCA
GCTGAACGGTCTGGTTATAGGTACATTGAGCAACTGACTGAAATGCCTCAAAATGTTCTTTACGATGCCATTGGGATATATCAACGGTGGTATATCCAGTGATTTTTTTCTCCATTTTAGCTTCCT
TAGCTCCTGAAAATCTCGATAACTCAAAAAATACGCCCGGTAGTGATCTTATTTCATTATGGTGAAAGTTGGAACCTCTTACGTGCCGATCAAGATCAAAGGATCTTCTTGAGATCCCCAGCTGG
CAATTCCGACGTCTAAGAAACCATTATTATCATGACATTAACCTATAAAAATAGGCGTATCACGAGGCCCTTTCGTCTTCACCTCGAGGGGATCCCACCACAATTCAGCAAATTGTGAACATCAT
CACGTTCATCTTTCCCTGGTTGCCAATGGCCCATTTTCCTGTCA

prhaBAD-FepA ΔC/ΔL NSEGS

GTAACGAGAAGGTCGCGAATTCAGGCGCTTTTTAGACTGGTCGTAATGAACCTCTAGAAATAATTTTGTTTAACTTTAAGAAGGAGATATACATATGAACAAGAAGATTCATTCCCTGGCCTTGT
TGGTCAATCTGGGGATTTATGGGGTAGCGCAGGCAGGCGAGTGGCACGGCTCCTGGGACGCATATTTCAATGCGCCAAATAGTGAAGGTAGCACCAAACGCACTAACTTTAGCCTGACCGGT
CCGCTGGGCGACGAATTCAGCTTCCGTTTGTATGGCAACCTCGACAAAACCAATAGTGAAGGTAGCGGGGTAATCAACAAAGATATTAATGGCGTGGTGCGCTGGGATTTCGCGCCATTGCAA
TCGCTGGAACTGGAAGCAGGTTACAGCCGCCAGGGTAACCTGAATAGTGAAGGTAGCAACCGTCTGTATCGCCAGAACTACGCGCTGACCTGGAACGGTGGCTGGGATAACGGCGTGACCAC
CAGCAACTGGGTGCAGTACGAACACACCCGTAACTCGCGTAATAGTGAAGGTAGCGTCGATATCGATCTTGATGACGTGATGCTGCACAGCGAAGTTAACCTGCCGATTGATTTCCTCGTTAAC
CAGACGCTGACGCTGGGTACGGAGTGGAATCAGCAACGGATGAAGAATAGTGAAGGTAGCAAAGCAGAAATTTTCTCGCTGTTTGCCGAAAACAACATGGAGCTGACTGACAGCACCATCGT
AACGCCGGGGCTGCGTTTCGATCATCACAGTATTGTCGGCAATAACTGGAGCCCGGCGCTGAACATATCGCAAGGTTTAGGCGATGACTTCACGCTGAAAATGGGCATCGCCCGTGCTTATAA
AAATAGTGAAGGTAGCGAAACCAGCATCAACAAAGAGATTGGTCTGGAGTTCAAACGCGACGGGTGGCTGGCGGGCGTCACCTGGTTCCGTAACGATTATCGCAATAAGATTGAAGCGAATA
GTGAAGGTAGCCTCTATCAGTGGGATAACGTGCCGAAAGCGGTGGTTGAAGGTCTGGAAGGATCGTTAAACGTACCGGTTAGCGAAACGGTGATGTGGACCAATAACATCACTTATATGCTG
AAGAGTGAAAACAATAGTGAAGGTAGCTATACGTTGAACTCAACGCTGAGCTGGCAGGCACGGGAAGATTTGTCGATGCAAACGACCTTCACCTGGTACAATAGTGAAGGTAGCTACAGCAT
TGTTGGCCTGAGCGCGACCTGGGATGTGACGAAGAATGTCAGTCTGACCGGCGGCGTGGACAATCTGTTCGACAATAGTGAAGGTAGCTGGTATATGAGCGTAAACACCCACTTCTGAATGTC
CAGACCTGCAGGCATGCAAGCTCTAGAGGCATCAAATAAAACGAAAGGCTCAGTCGAAAGACTGGGCCTTTCGTTTTATCTGTTGTTTGTCGGTGAACGCTCTCCTGAGTAGGACAAATCCGCC
GCCCTAGACCTAGGGTACGGGTTTTGCTGCCCGCAAACGGGCTGTTCTGGTGTTGCTAGTTTGTTATCAGAATCGCAGATCCGGCTTCAGCCGGTTTGCCGGCTGAAAGCGCTATTTCTTCCAG
AATTGCCATGATTTTTTCCCCACGGGAGGCGTCACTGGCTCCCGTGTTGTCGGCAGCTTTGATTCGATAAGCAGCATCGCCTGTTTCAGGCTGTCTATGTGTGACTGTTGAGCTGTAACAAGTTG
TCTCAGGTGTTCAATTTCATGTTCTAGTTGCTTTGTTTTACTGGTTTCACCTGTTCTATTAGGTGTTACATGCTGTTCATCTGTTACATTGTCGATCTGTTCATGGTGAACAGCTTTGAATGCACCAA
AAACTCGTAAAAGCTCTGATGTATCTATCTTTTTTACACCGTTTTCATCTGTGCATATGGACAGTTTTCCCTTTGATATGTAACGGTGAACAGTTGTTCTACTTTTGTTTGTTAGTCTTGATGCTTCA
CTGATAGATACAAGAGCCATAAGAACCTCAGATCCTTCCGTATTTAGCCAGTATGTTCTCTAGTGTGGTTCGTTGTTTTTGCGTGAGCCATGAGAACGAACCATTGAGATCATACTTACTTTGCAT
GTCACTCAAAAATTTTGCCTCAAAACTGGTGAGCTGAATTTTTGCAGTTAAAGCATCGTGTAGTGTTTTTCTTAGTCCGTTATGTAGGTAGGAATCTGATGTAATGGTTGTTGGTATTTTGTCACC
ATTCATTTTTATCTGGTTGTTCTCAAGTTCGGTTACGAGATCCATTTGTCTATCTAGTTCAACTTGGAAAATCAACGTATCAGTCGGGCGGCCTCGCTTATCAACCACCAATTTCATATTGCTGTAA
GTGTTTAAATCTTTACTTATTGGTTTCAAAACCCATTGGTTAAGCCTTTTAAACTCATGGTAGTTATTTTCAAGCATTAACATGAACTTAAATTCATCAAGGCTAATCTCTATATTTGCCTTGTGAGT
TTTCTTTTGTGTTAGTTCTTTTAATAACCACTCATAAATCCTCATAGAGTATTTGTTTTCAAAAGACTTAACATGTTCCAGATTATATTTTATGAATTTTTTTAACTGGAAAAGATAAGGCAATATCT
CTTCACTAAAAACTAATTCTAATTTTTCGCTTGAGAACTTGGCATAGTTTGTCCACTGGAAAATCTCAAAGCCTTTAACCAAAGGATTCCTGATTTCCACAGTTCTCGTCATCAGCTCTCTGGTTGC
TTTAGCTAATACACCATAAGCATTTTCCCTACTGATGTTCATCATCTGAGCGTATTGGTTATAAGTGAACGATACCGTCCGTTCTTTCCTTGTAGGGTTTTCAATCGTGGGGTTGAGTAGTGCCAC
ACAGCATAAAATTAGCTTGGTTTCATGCTCCGTTAAGTCATAGCGACTAATCGCTAGTTCATTTGCTTTGAAAACAACTAATTCAGACATACATCTCAATTGGTCTAGGTGATTTTAATCACTATAC
CAATTGAGATGGGCTAGTCAATGATAATTACTAGTCCTTTTCCTTTGAGTTGTGGGTATCTGTAAATTCTGCTAGACCTTTGCTGGAAAACTTGTAAATTCTGCTAGACCCTCTGTAAATTCCGCT
AGACCTTTGTGTGTTTTTTTTGTTTATATTCAAGTGGTTATAATTTATAGAATAAAGAAAGAATAAAAAAAGATAAAAAGAATAGATCCCAGCCCTGTGTATAACTCACTACTTTAGTCAGTTCCG
CAGTATTACAAAAGGATGTCGCAAACGCTGTTTGCTCCTCTACAAAACAGACCTTAAAACCCTAAAGGCTTAAGTAGCGCCGTCGCAAGCTCCGGCAAATCGCTGAATATTCCTTTTGTCTCCGA
CCATCAGGCACCTGAGTCGCTGTCTTTTTCGTCACATTCAGTTCGCTGCGCTCACGGCTCTGGCAGTGAATGGGGGTAAATGGCACTACAGGCGCCTTTTATGGATTCATGCAAGGAAACTACC
CATAATACAAGAAAAGCCCGTCACGGGCTTCTCAGGGCGTTTTATGGCGGGTCTGCTATGTGGTGCTATCTGACTTTTTGCTGTTCAGCAGTTCCTGCCCTCTGATTTTCCAGTCTGACCACTTCG
GATTATCCCGTGACAGGTCATTCAGACTGGCTAATGCACCCAGTAAGGCAGCGGTATCATCAACAGGCTTACCCGTCTTACTGTCCCTAGTGCTTGGATTCTCACCAATAAAAAACGCCCGGCG
GCAACCGAGCGTTCTGAACAAATCCAGATGGAGTTCTGAGGTCATTACTGGATCTATCAACAGGAGTCCAAGCGAGCTCTCGAACCCCAGAGTCCCGCTATGTATCCGCTCATGAATTAATTCTT
ATTACGCCCCGCCCTGCCACTCATCGCAGTACTGTTGTAATTCATTAAGCATTCTGCCGACATGGAAGCCATCACAAACGGCATGATGAACCTGAATCGCCAGCGGCATCAGCACCTTGTCGCCT
TGCGTATAATATTTGCCCATGGTGAAAACGGGGGCGAAGAAGTTGTCCATATTGGCCACGTTTAAATCAAAACTGGTGAAACTCACCCAGGGATTGGCTGAGACGAAAAACATATTCTCAATA
AACCCTTTAGGGAAATAGGCCAGGTTTTCACCGTAACACGCCACATCTTGCGAATATATGTGTAGAAACTGCCGGAAATCGTCGTGGTATTCACTCCAGAGCGATGAAAACGTTTCAGTTTGCT
CATGGAAAACGGTGTAACAAGGGTGAACACTATCCCATATCACCAGCTCACCGTCTTTCATTGCCATACGAAATTCCGGATGAGCATTCATCAGGCGGGCAAGAATGTGAATAAAGGCCGGAT
AAAACTTGTGCTTATTTTTCTTTACGGTCTTTAAAAAGGCCGTAATATCCAGCTGAACGGTCTGGTTATAGGTACATTGAGCAACTGACTGAAATGCCTCAAAATGTTCTTTACGATGCCATTGGG
ATATATCAACGGTGGTATATCCAGTGATTTTTTTCTCCATTTTAGCTTCCTTAGCTCCTGAAAATCTCGATAACTCAAAAAATACGCCCGGTAGTGATCTTATTTCATTATGGTGAAAGTTGGAACC
TCTTACGTGCCGATCAAGATCAAAGGATCTTCTTGAGATCCCCAGCTGGCAATTCCGACGTCTAAGAAACCATTATTATCATGACATTAACCTATAAAAATAGGCGTATCACGAGGCCCTTTCGT
CTTCACCTCGAGGGGATCCCACCACAATTCAGCAAATTGTGAACATCATCACGTTCATCTTTCCCTGGTTGCCAATGGCCCATTTTCCTGTCA

prhaBAD-OmpF Δ

GTAACGAGAAGGTCGCGAATTCAGGCGCTTTTTAGACTGGTCGTAATGAACCTCTAGAAATAATTTTGTTTAACTTTAAGAAGGAGATATACATATGAAAAAGAGCACTCTGGCATTAGTGGTG
ATGGGCATTGTGGCATCTGCATCTGTACAGGCTGCAGAAATCTATAACAAAGATGGCAACAAAGTAGATCTGTACGGTAAAGCTGTTGGTCTGCATTATTTTTCCAAGGGTAACGGTGAAAACA
GTTACGGTGGCAATGGCGACATGACCTATGCCCGTCTTGGTTTTAAAGGGGAAACTCAAATCAATTCCGATCTGACCGGTTATGGTCAGTGGGAATATAACTTCCAGGGTAACAACTCTGAAGG
CGCTGACGCTCAAACTGGTAACAAAACGCGTCTGGCATTCGCGGGTCTTAAATACGCTGACGTTGGTTCTTTCGATTACGGCCGTAACTACGGTGTGGTTTATGATGCACTGCCAGAATTTGGT
GGTGATACTGCATACAGCGATGACTTCTTCGTTGGTCGTGTTGGCGGCGTTGCTACCTATCGTAACTCCAACTTCTTTGGTCTGGTTGATGGCCTGAACTTCGCTGTTCAGTACCTGGGTAAAAA
CGAGCGTGACACTGCACGCCGTTCTAACGGCGACGGTGTTGGCGGTTCTATCAGCTACGAATACGAAGGCTTTGGTATCGTTGGTGCTTATGGTGCAGCTGACCGTACCAACCTGCAAGAAGC
TCAACCTCTTGGCAACGGTAAAAAAGCTGAACAGTGGGCTACTGGTCTGAAGTACGACGCGAACAACATCTACCTGGCAGCGAACTACGGTGAAACCCGTAACGCTACGCCGATCACTAATAA
ATTTACAAACACCAGCGGCTTCGCCAACAAAACGCAAGACGTTCTGTTAGTTGCGCAATACCAGTTCGATTTCGGTCTGCGTCCGTCCATCGCTTACACCAAATCTAAAGCGAAAGACGTAGAA
GGTATCGGTGATGTTGATCTGGTGAACTACTTTGAAGTGGGCGCAACCTACTACTTCAACAAAAACATGTCCACCTATGTTGACTACATCATCAACCAGATCGATTCTGACAACAAACTGGGCGT
AGGTTCAGACGACACCGTTGCTGTGGGTATCGTTTACCAGTTCTAAATGTCCAGACCTGCAGGCATGCAAGCTCTAGAGGCATCAAATAAAACGAAAGGCTCAGTCGAAAGACTGGGCCTTTC
GTTTTATCTGTTGTTTGTCGGTGAACGCTCTCCTGAGTAGGACAAATCCGCCGCCCTAGACCTAGGGTACGGGTTTTGCTGCCCGCAAACGGGCTGTTCTGGTGTTGCTAGTTTGTTATCAGAAT
CGCAGATCCGGCTTCAGCCGGTTTGCCGGCTGAAAGCGCTATTTCTTCCAGAATTGCCATGATTTTTTCCCCACGGGAGGCGTCACTGGCTCCCGTGTTGTCGGCAGCTTTGATTCGATAAGCAG
CATCGCCTGTTTCAGGCTGTCTATGTGTGACTGTTGAGCTGTAACAAGTTGTCTCAGGTGTTCAATTTCATGTTCTAGTTGCTTTGTTTTACTGGTTTCACCTGTTCTATTAGGTGTTACATGCTGTT
CATCTGTTACATTGTCGATCTGTTCATGGTGAACAGCTTTGAATGCACCAAAAACTCGTAAAAGCTCTGATGTATCTATCTTTTTTACACCGTTTTCATCTGTGCATATGGACAGTTTTCCCTTTGA
TATGTAACGGTGAACAGTTGTTCTACTTTTGTTTGTTAGTCTTGATGCTTCACTGATAGATACAAGAGCCATAAGAACCTCAGATCCTTCCGTATTTAGCCAGTATGTTCTCTAGTGTGGTTCGTT
GTTTTTGCGTGAGCCATGAGAACGAACCATTGAGATCATACTTACTTTGCATGTCACTCAAAAATTTTGCCTCAAAACTGGTGAGCTGAATTTTTGCAGTTAAAGCATCGTGTAGTGTTTTTCTTA
GTCCGTTATGTAGGTAGGAATCTGATGTAATGGTTGTTGGTATTTTGTCACCATTCATTTTTATCTGGTTGTTCTCAAGTTCGGTTACGAGATCCATTTGTCTATCTAGTTCAACTTGGAAAATCAA
CGTATCAGTCGGGCGGCCTCGCTTATCAACCACCAATTTCATATTGCTGTAAGTGTTTAAATCTTTACTTATTGGTTTCAAAACCCATTGGTTAAGCCTTTTAAACTCATGGTAGTTATTTTCAAGC
ATTAACATGAACTTAAATTCATCAAGGCTAATCTCTATATTTGCCTTGTGAGTTTTCTTTTGTGTTAGTTCTTTTAATAACCACTCATAAATCCTCATAGAGTATTTGTTTTCAAAAGACTTAACATG
TTCCAGATTATATTTTATGAATTTTTTTAACTGGAAAAGATAAGGCAATATCTCTTCACTAAAAACTAATTCTAATTTTTCGCTTGAGAACTTGGCATAGTTTGTCCACTGGAAAATCTCAAAGCCT
TTAACCAAAGGATTCCTGATTTCCACAGTTCTCGTCATCAGCTCTCTGGTTGCTTTAGCTAATACACCATAAGCATTTTCCCTACTGATGTTCATCATCTGAGCGTATTGGTTATAAGTGAACGATA
CCGTCCGTTCTTTCCTTGTAGGGTTTTCAATCGTGGGGTTGAGTAGTGCCACACAGCATAAAATTAGCTTGGTTTCATGCTCCGTTAAGTCATAGCGACTAATCGCTAGTTCATTTGCTTTGAAAA
CAACTAATTCAGACATACATCTCAATTGGTCTAGGTGATTTTAATCACTATACCAATTGAGATGGGCTAGTCAATGATAATTACTAGTCCTTTTCCTTTGAGTTGTGGGTATCTGTAAATTCTGCTA
GACCTTTGCTGGAAAACTTGTAAATTCTGCTAGACCCTCTGTAAATTCCGCTAGACCTTTGTGTGTTTTTTTTGTTTATATTCAAGTGGTTATAATTTATAGAATAAAGAAAGAATAAAAAAAGAT
AAAAAGAATAGATCCCAGCCCTGTGTATAACTCACTACTTTAGTCAGTTCCGCAGTATTACAAAAGGATGTCGCAAACGCTGTTTGCTCCTCTACAAAACAGACCTTAAAACCCTAAAGGCTTAA
GTAGCGCCGTCGCAAGCTCCGGCAAATCGCTGAATATTCCTTTTGTCTCCGACCATCAGGCACCTGAGTCGCTGTCTTTTTCGTCACATTCAGTTCGCTGCGCTCACGGCTCTGGCAGTGAATGG
GGGTAAATGGCACTACAGGCGCCTTTTATGGATTCATGCAAGGAAACTACCCATAATACAAGAAAAGCCCGTCACGGGCTTCTCAGGGCGTTTTATGGCGGGTCTGCTATGTGGTGCTATCTGA
CTTTTTGCTGTTCAGCAGTTCCTGCCCTCTGATTTTCCAGTCTGACCACTTCGGATTATCCCGTGACAGGTCATTCAGACTGGCTAATGCACCCAGTAAGGCAGCGGTATCATCAACAGGCTTACC
CGTCTTACTGTCCCTAGTGCTTGGATTCTCACCAATAAAAAACGCCCGGCGGCAACCGAGCGTTCTGAACAAATCCAGATGGAGTTCTGAGGTCATTACTGGATCTATCAACAGGAGTCCAAGC
GAGCTCTCGAACCCCAGAGTCCCGCTATGTATCCGCTCATGAATTAATTCTTATTACGCCCCGCCCTGCCACTCATCGCAGTACTGTTGTAATTCATTAAGCATTCTGCCGACATGGAAGCCATCA
CAAACGGCATGATGAACCTGAATCGCCAGCGGCATCAGCACCTTGTCGCCTTGCGTATAATATTTGCCCATGGTGAAAACGGGGGCGAAGAAGTTGTCCATATTGGCCACGTTTAAATCAAAAC
TGGTGAAACTCACCCAGGGATTGGCTGAGACGAAAAACATATTCTCAATAAACCCTTTAGGGAAATAGGCCAGGTTTTCACCGTAACACGCCACATCTTGCGAATATATGTGTAGAAACTGCCG
GAAATCGTCGTGGTATTCACTCCAGAGCGATGAAAACGTTTCAGTTTGCTCATGGAAAACGGTGTAACAAGGGTGAACACTATCCCATATCACCAGCTCACCGTCTTTCATTGCCATACGAAATT
CCGGATGAGCATTCATCAGGCGGGCAAGAATGTGAATAAAGGCCGGATAAAACTTGTGCTTATTTTTCTTTACGGTCTTTAAAAAGGCCGTAATATCCAGCTGAACGGTCTGGTTATAGGTACA
TTGAGCAACTGACTGAAATGCCTCAAAATGTTCTTTACGATGCCATTGGGATATATCAACGGTGGTATATCCAGTGATTTTTTTCTCCATTTTAGCTTCCTTAGCTCCTGAAAATCTCGATAACTCA
AAAAATACGCCCGGTAGTGATCTTATTTCATTATGGTGAAAGTTGGAACCTCTTACGTGCCGATCAAGATCAAAGGATCTTCTTGAGATCCCCAGCTGGCAATTCCGACGTCTAAGAAACCATTA
TTATCATGACATTAACCTATAAAAATAGGCGTATCACGAGGCCCTTTCGTCTTCACCTCGAGGGGATCCCACCACAATTCAGCAAATTGTGAACATCATCACGTTCATCTTTCCCTGGTTGCCAAT
GGCCCATTTTCCTGTCA

prhaBAD-OmpF Δ GIGL

GTAACGAGAAGGTCGCGAATTCAGGCGCTTTTTAGACTGGTCGTAATGAACCTCTAGAAATAATTTTGTTTAACTTTAAGAAGGAGATATACATATGAAAAAGAGCACTCTGGCATTAGTGGTG
ATGGGCATTGTGGCATCTGCATCTGTACAGGCTGCAGAAATCTATAACAAAGATGGCAACAAAGTAGATCTGTACGGTAAAGCTGTTGGTCTGCATTATTTTTCCAAGGGTAACGGTGAAAACA
GTTACGGTGGCAATGGCGACATGACCTATGCCCGTCTTGGTTTTAAAGGGGAAACTCAAATCAATTCCGATCTGACCATCTATCTTCAGTGGGAATATAACTTCCAGGGTAACAACTCTGAAGG
CGCTGACGCTCAAACTGGTAACAAAACGCGTCTGGCATTCGCGGGTCTTAAATACGCTGACGTTGGTTCTTTCGATTACGGCCGTAACTACGGTGTGGTTTATGATGCACTGCCAGAATTTGGT
GGTGATACTGCATACAGCGATGACTTCTTCGTTGGTCGTGTTGGCGGCGTTGCTACCTATCGTAACTCCAACTTCTTTGGTCTGGTTGATGGCCTGAACTTCGCTGTTCAGTACCTGGGTAAAAA
CGAGCGTGACACTGCACGCCGTTCTAACGGCGACGGTGTTGGCGGTTCTATCAGCTACGAATACGAAGGCTTTGGTATCGTTGGTGCTTATGGTGCAGCTGACCGTACCAACCTGCAAGAAGC
TCAACCTCTTGGCAACGGTAAAAAAGCTGAACAGTGGGCTACTGGTCTGAAGTACGACGCGAACAACATCTACCTGGCAGCGAACTACGGTGAAACCCGTAACGCTACGCCGATCACTAATAA
ATTTACAAACACCAGCGGCTTCGCCAACAAAACGCAAGACGTTCTGTTAGTTGCGCAATACCAGTTCGATTTCGGTCTGCGTCCGTCCATCGCTTACACCAAATCTAAAGCGAAAGACGTAGAA
GGTATCGGTGATGTTGATCTGGTGAACTACTTTGAAGTGGGCGCAACCTACTACTTCAACAAAAACATGTCCACCTATGTTGACTACATCATCAACCAGATCGATTCTGACAACAAACTGGGCGT
AGGTTCAGACGACACCGTTGCTGTGGGTATCGTTTACCAGTTCTAAATGTCCAGACCTGCAGGCATGCAAGCTCTAGAGGCATCAAATAAAACGAAAGGCTCAGTCGAAAGACTGGGCCTTTC
GTTTTATCTGTTGTTTGTCGGTGAACGCTCTCCTGAGTAGGACAAATCCGCCGCCCTAGACCTAGGGTACGGGTTTTGCTGCCCGCAAACGGGCTGTTCTGGTGTTGCTAGTTTGTTATCAGAAT
CGCAGATCCGGCTTCAGCCGGTTTGCCGGCTGAAAGCGCTATTTCTTCCAGAATTGCCATGATTTTTTCCCCACGGGAGGCGTCACTGGCTCCCGTGTTGTCGGCAGCTTTGATTCGATAAGCAG
CATCGCCTGTTTCAGGCTGTCTATGTGTGACTGTTGAGCTGTAACAAGTTGTCTCAGGTGTTCAATTTCATGTTCTAGTTGCTTTGTTTTACTGGTTTCACCTGTTCTATTAGGTGTTACATGCTGTT
CATCTGTTACATTGTCGATCTGTTCATGGTGAACAGCTTTGAATGCACCAAAAACTCGTAAAAGCTCTGATGTATCTATCTTTTTTACACCGTTTTCATCTGTGCATATGGACAGTTTTCCCTTTGA
TATGTAACGGTGAACAGTTGTTCTACTTTTGTTTGTTAGTCTTGATGCTTCACTGATAGATACAAGAGCCATAAGAACCTCAGATCCTTCCGTATTTAGCCAGTATGTTCTCTAGTGTGGTTCGTT
GTTTTTGCGTGAGCCATGAGAACGAACCATTGAGATCATACTTACTTTGCATGTCACTCAAAAATTTTGCCTCAAAACTGGTGAGCTGAATTTTTGCAGTTAAAGCATCGTGTAGTGTTTTTCTTA
GTCCGTTATGTAGGTAGGAATCTGATGTAATGGTTGTTGGTATTTTGTCACCATTCATTTTTATCTGGTTGTTCTCAAGTTCGGTTACGAGATCCATTTGTCTATCTAGTTCAACTTGGAAAATCAA
CGTATCAGTCGGGCGGCCTCGCTTATCAACCACCAATTTCATATTGCTGTAAGTGTTTAAATCTTTACTTATTGGTTTCAAAACCCATTGGTTAAGCCTTTTAAACTCATGGTAGTTATTTTCAAGC
ATTAACATGAACTTAAATTCATCAAGGCTAATCTCTATATTTGCCTTGTGAGTTTTCTTTTGTGTTAGTTCTTTTAATAACCACTCATAAATCCTCATAGAGTATTTGTTTTCAAAAGACTTAACATG
TTCCAGATTATATTTTATGAATTTTTTTAACTGGAAAAGATAAGGCAATATCTCTTCACTAAAAACTAATTCTAATTTTTCGCTTGAGAACTTGGCATAGTTTGTCCACTGGAAAATCTCAAAGCCT
TTAACCAAAGGATTCCTGATTTCCACAGTTCTCGTCATCAGCTCTCTGGTTGCTTTAGCTAATACACCATAAGCATTTTCCCTACTGATGTTCATCATCTGAGCGTATTGGTTATAAGTGAACGATA
CCGTCCGTTCTTTCCTTGTAGGGTTTTCAATCGTGGGGTTGAGTAGTGCCACACAGCATAAAATTAGCTTGGTTTCATGCTCCGTTAAGTCATAGCGACTAATCGCTAGTTCATTTGCTTTGAAAA
CAACTAATTCAGACATACATCTCAATTGGTCTAGGTGATTTTAATCACTATACCAATTGAGATGGGCTAGTCAATGATAATTACTAGTCCTTTTCCTTTGAGTTGTGGGTATCTGTAAATTCTGCTA
GACCTTTGCTGGAAAACTTGTAAATTCTGCTAGACCCTCTGTAAATTCCGCTAGACCTTTGTGTGTTTTTTTTGTTTATATTCAAGTGGTTATAATTTATAGAATAAAGAAAGAATAAAAAAAGAT
AAAAAGAATAGATCCCAGCCCTGTGTATAACTCACTACTTTAGTCAGTTCCGCAGTATTACAAAAGGATGTCGCAAACGCTGTTTGCTCCTCTACAAAACAGACCTTAAAACCCTAAAGGCTTAA
GTAGCGCCGTCGCAAGCTCCGGCAAATCGCTGAATATTCCTTTTGTCTCCGACCATCAGGCACCTGAGTCGCTGTCTTTTTCGTCACATTCAGTTCGCTGCGCTCACGGCTCTGGCAGTGAATGG
GGGTAAATGGCACTACAGGCGCCTTTTATGGATTCATGCAAGGAAACTACCCATAATACAAGAAAAGCCCGTCACGGGCTTCTCAGGGCGTTTTATGGCGGGTCTGCTATGTGGTGCTATCTGA
CTTTTTGCTGTTCAGCAGTTCCTGCCCTCTGATTTTCCAGTCTGACCACTTCGGATTATCCCGTGACAGGTCATTCAGACTGGCTAATGCACCCAGTAAGGCAGCGGTATCATCAACAGGCTTACC
CGTCTTACTGTCCCTAGTGCTTGGATTCTCACCAATAAAAAACGCCCGGCGGCAACCGAGCGTTCTGAACAAATCCAGATGGAGTTCTGAGGTCATTACTGGATCTATCAACAGGAGTCCAAGC
GAGCTCTCGAACCCCAGAGTCCCGCTATGTATCCGCTCATGAATTAATTCTTATTACGCCCCGCCCTGCCACTCATCGCAGTACTGTTGTAATTCATTAAGCATTCTGCCGACATGGAAGCCATCA
CAAACGGCATGATGAACCTGAATCGCCAGCGGCATCAGCACCTTGTCGCCTTGCGTATAATATTTGCCCATGGTGAAAACGGGGGCGAAGAAGTTGTCCATATTGGCCACGTTTAAATCAAAAC
TGGTGAAACTCACCCAGGGATTGGCTGAGACGAAAAACATATTCTCAATAAACCCTTTAGGGAAATAGGCCAGGTTTTCACCGTAACACGCCACATCTTGCGAATATATGTGTAGAAACTGCCG
GAAATCGTCGTGGTATTCACTCCAGAGCGATGAAAACGTTTCAGTTTGCTCATGGAAAACGGTGTAACAAGGGTGAACACTATCCCATATCACCAGCTCACCGTCTTTCATTGCCATACGAAATT
CCGGATGAGCATTCATCAGGCGGGCAAGAATGTGAATAAAGGCCGGATAAAACTTGTGCTTATTTTTCTTTACGGTCTTTAAAAAGGCCGTAATATCCAGCTGAACGGTCTGGTTATAGGTACA
TTGAGCAACTGACTGAAATGCCTCAAAATGTTCTTTACGATGCCATTGGGATATATCAACGGTGGTATATCCAGTGATTTTTTTCTCCATTTTAGCTTCCTTAGCTCCTGAAAATCTCGATAACTCA
AAAAATACGCCCGGTAGTGATCTTATTTCATTATGGTGAAAGTTGGAACCTCTTACGTGCCGATCAAGATCAAAGGATCTTCTTGAGATCCCCAGCTGGCAATTCCGACGTCTAAGAAACCATTA
TTATCATGACATTAACCTATAAAAATAGGCGTATCACGAGGCCCTTTCGTCTTCACCTCGAGGGGATCCCACCACAATTCAGCAAATTGTGAACATCATCACGTTCATCTTTCCCTGGTTGCCAAT
GGCCCATTTTCCTGTCA

pSAV S112M K121R

TGGCGAATGGGACGCGCCCTGTAGCGGCGCATTAAGCGCGGCGGGTGTGGTGGTTACGCGCAGCGTGACCGCTACACTTGCCAGCGCCCTAGCGCCCGCTCCTTTCGCTTTCTTCCCTTCCTTT
CTCGCCACGTTCGCCGGCTTTCCCCGTCAAGCTCTAAATCGGGGGCTCCCTTTAGGGTTCCGATTTAGTGCTTTACGGCACCTCGACCCCAAAAAACTTGATTAGGGTGATGGTTCACGTAGTGG
GCCATCGCCCTGATAGACGGTTTTTCGCCCTTTGACGTTGGAGTCCACGTTCTTTAATAGTGGACTCTTGTTCCAAACTGGAACAACACTCAACCCTATCTCGGTCTATTCTTTTGATTTATAAGGG
ATTTTGCCGATTTCGGCCTATTGGTTAAAAAATGAGCTGATTTAACAAAAATTTAACGCGAATTTTAACAAAATATTAACGTTTACAATTTCAGGTGGCACTTTTCGGGGAAATGTGCGCGGAAC
CCCTATTTGTTTATTTTTCTAAATACATTCAAATATGTATCCGCTCATGAATTAATTCTTAGAAAAACTCATCGAGCATCAAATGAAACTGCAATTTATTCATATCAGGATTATCAATACCATATTTT
TGAAAAAGCCGTTTCTGTAATGAAGGAGAAAACTCACCGAGGCAGTTCCATAGGATGGCAAGATCCTGGTATCGGTCTGCGATTCCGACTCGTCCAACATCAATACAACCTATTAATTTCCCCTC
GTCAAAAATAAGGTTATCAAGTGAGAAATCACCATGAGTGACGACTGAATCCGGTGAGAATGGCAAAAGTTTATGCATTTCTTTCCAGACTTGTTCAACAGGCCAGCCATTACGCTCGTCATCA
AAATCACTCGCATCAACCAAACCGTTATTCATTCGTGATTGCGCCTGAGCGAGACGAAATACGCGATCGCTGTTAAAAGGACAATTACAAACAGGAATCGAATGCAACCGGCGCAGGAACACT
GCCAGCGCATCAACAATATTTTCACCTGAATCAGGATATTCTTCTAATACCTGGAATGCTGTTTTCCCGGGGATCGCAGTGGTGAGTAACCATGCATCATCAGGAGTACGGATAAAATGCTTGAT
GGTCGGAAGAGGCATAAATTCCGTCAGCCAGTTTAGTCTGACCATCTCATCTGTAACATCATTGGCAACGCTACCTTTGCCATGTTTCAGAAACAACTCTGGCGCATCGGGCTTCCCATACAATC
GATAGATTGTCGCACCTGATTGCCCGACATTATCGCGAGCCCATTTATACCCATATAAATCAGCATCCATGTTGGAATTTAATCGCGGCCTAGAGCAAGACGTTTCCCGTTGAATATGGCTCATA
ACACCCCTTGTATTACTGTTTATGTAAGCAGACAGTTTTATTGTTCATGACCAAAATCCCTTAACGTGAGTTTTCGTTCCACTGAGCGTCAGACCCCGTAGAAAAGATCAAAGGATCTTCTTGAGA
TCCTTTTTTTCTGCGCGTAATCTGCTGCTTGCAAACAAAAAAACCACCGCTACCAGCGGTGGTTTGTTTGCCGGATCAAGAGCTACCAACTCTTTTTCCGAAGGTAACTGGCTTCAGCAGAGCGC
AGATACCAAATACTGTCCTTCTAGTGTAGCCGTAGTTAGGCCACCACTTCAAGAACTCTGTAGCACCGCCTACATACCTCGCTCTGCTAATCCTGTTACCAGTGGCTGCTGCCAGTGGCGATAAG
TCGTGTCTTACCGGGTTGGACTCAAGACGATAGTTACCGGATAAGGCGCAGCGGTCGGGCTGAACGGGGGGTTCGTGCACACAGCCCAGCTTGGAGCGAACGACCTACACCGAACTGAGATA
CCTACAGCGTGAGCTATGAGAAAGCGCCACGCTTCCCGAAGGGAGAAAGGCGGACAGGTATCCGGTAAGCGGCAGGGTCGGAACAGGAGAGCGCACGAGGGAGCTTCCAGGGGGAAACGC
CTGGTATCTTTATAGTCCTGTCGGGTTTCGCCACCTCTGACTTGAGCGTCGATTTTTGTGATGCTCGTCAGGGGGGCGGAGCCTATGGAAAAACGCCAGCAACGCGGCCTTTTTACGGTTCCTG
GCCTTTTGCTGGCCTTTTGCTCACATGTTCTTTCCTGCGTTATCCCCTGATTCTGTGGATAACCGTATTACCGCCTTTGAGTGAGCTGATACCGCTCGCCGCAGCCGAACGACCGAGCGCAGCGA
GTCAGTGAGCGAGGAAGCGGAAGAGCGCCTGATGCGGTATTTTCTCCTTACGCATCTGTGCGGTATTTCACACCGCATATATGGTGCACTCTCAGTACAATCTGCTCTGATGCCGCATAGTTAA
GCCAGTATACACTCCGCTATCGCTACGTGACTGGGTCATGGCTGCGCCCCGACACCCGCCAACACCCGCTGACGCGCCCTGACGGGCTTGTCTGCTCCCGGCATCCGCTTACAGACAAGCTGTG
ACCGTCTCCGGGAGCTGCATGTGTCAGAGGTTTTCACCGTCATCACCGAAACGCGCGAGGCAGCTGCGGTAAAGCTCATCAGCGTGGTCGTGAAGCGATTCACAGATGTCTGCCTGTTCATCC
GCGTCCAGCTCGTTGAGTTTCTCCAGAAGCGTTAATGTCTGGCTTCTGATAAAGCGGGCCATGTTAAGGGCGGTTTTTTCCTGTTTGGTCACTGATGCCTCCGTGTAAGGGGGATTTCTGTTCAT
GGGGGTAATGATACCGATGAAACGAGAGAGGATGCTCACGATACGGGTTACTGATGATGAACATGCCCGGTTACTGGAACGTTGTGAGGGTAAACAACTGGCGGTATGGATGCGGCGGGAC
CAGAGAAAAATCACTCAGGGTCAATGCCAGCGCTTCGTTAATACAGATGTAGGTGTTCCACAGGGTAGCCAGCAGCATCCTGCGATGCAGATCCGGAACATAATGGTGCAGGGCGCTGACTTC
CGCGTTTCCAGACTTTACGAAACACGGAAACCGAAGACCATTCATGTTGTTGCTCAGGTCGCAGACGTTTTGCAGCAGCAGTCGCTTCACGTTCGCTCGCGTATCGGTGATTCATTCTGCTAACC
AGTAAGGCAACCCCGCCAGCCTAGCCGGGTCCTCAACGACAGGAGCACGATCATGCGCACCCGTGGGGCCGCCATGCCGGCGATAATGGCCTGCTTCTCGCCGAAACGTTTGGTGGCGGGAC
CAGTGACGAAGGCTTGAGCGAGGGCGTGCAAGATTCCGAATACCGCAAGCGACAGGCCGATCATCGTCGCGCTCCAGCGAAAGCGGTCCTCGCCGAAAATGACCCAGAGCGCTGCCGGCAC
CTGTCCTACGAGTTGCATGATAAAGAAGACAGTCATAAGTGCGGCGACGATAGTCATGCCCCGCGCCCACCGGAAGGAGCTGACTGGGTTGAAGGCTCTCAAGGGCATCGGTCGAGATCCCG
GTGCCTAATGAGTGAGCTAACTTACATTAATTGCGTTGCGCTCACTGCCCGCTTTCCAGTCGGGAAACCTGTCGTGCCAGCTGCATTAATGAATCGGCCAACGCGCGGGGAGAGGCGGTTTGC
GTATTGGGCGCCAGGGTGGTTTTTCTTTTCACCAGTGAGACGGGCAACAGCTGATTGCCCTTCACCGCCTGGCCCTGAGAGAGTTGCAGCAAGCGGTCCACGCTGGTTTGCCCCAGCAGGCGA
AAATCCTGTTTGATGGTGGTTAACGGCGGGATATAACATGAGCTGTCTTCGGTATCGTCGTATCCCACTACCGAGATGTCCGCACCAACGCGCAGCCCGGACTCGGTAATGGCGCGCATTGCGC
CCAGCGCCATCTGATCGTTGGCAACCAGCATCGCAGTGGGAACGATGCCCTCATTCAGCATTTGCATGGTTTGTTGAAAACCGGACATGGCACTCCAGTCGCCTTCCCGTTCCGCTATCGGCTG
AATTTGATTGCGAGTGAGATATTTATGCCAGCCAGCCAGACGCAGACGCGCCGAGACAGAACTTAATGGGCCCGCTAACAGCGCGATTTGCTGGTGACCCAATGCGACCAGATGCTCCACGCC
CAGTCGCGTACCGTCTTCATGGGAGAAAATAATACTGTTGATGGGTGTCTGGTCAGAGACATCAAGAAATAACGCCGGAACATTAGTGCAGGCAGCTTCCACAGCAATGGCATCCTGGTCATC
CAGCGGATAGTTAATGATCAGCCCACTGACGCGTTGCGCGAGAAGATTGTGCACCGCCGCTTTACAGGCTTCGACGCCGCTTCGTTCTACCATCGACACCACCACGCTGGCACCCAGTTGATCG
GCGCGAGATTTAATCGCCGCGACAATTTGCGACGGCGCGTGCAGGGCCAGACTGGAGGTGGCAACGCCAATCAGCAACGACTGTTTGCCCGCCAGTTGTTGTGCCACGCGGTTGGGAATGTA
ATTCAGCTCCGCCATCGCCGCTTCCACTTTTTCCCGCGTTTTCGCAGAAACGTGGCTGGCCTGGTTCACCACGCGGGAAACGGTCTGATAAGAGACACCGGCATACTCTGCGACATCGTATAACG
TTACTGGTTTCACATTCACCACCCTGAATTGACTCTCTTCCGGGCGCTATCATGCCATACCGCGAAAGGTTTTGCGCCATTCGATGGTGTCCGGGATCTCGACGCTCTCCCTTATGCGACTCCTGC
ATTAGGAAGCAGCCCAGTAGTAGGTTGAGGCCGTTGAGCACCGCCGCCGCAAGGAATGGTGCATGCAAGGAGATGGCGCCCAACAGTCCCCCGGCCACGGGGCCTGCCACCATACCCACGCC
GAAACAAGCGCTCATGAGCCCGAAGTGGCGAGCCCGATCTTCCCCATCGGTGATGTCGGCGATATAGGCGCCAGCAACCGCACCTGTGGCGCCGGTGATGCCGGCCACGATGCGTCCGGCGT
AGAGGATCGAGATCGATCTCGATCCCGCGAAATTAATACGACTCACTATAGGGGAATTGTGAGCGGATAACAATTCCCCTCTAGAAATAATTTTGTTTAACTTTAAGAAGGAGATATACATATG
AAAAAGACAGCTATCGCGATTGCAGTGGCACTGGCTGGTTTCGCTACCGTAGCGCAGGCCGCTAGCATGACTGGTGGACAGCAAATGGGTCGGGATCAGGCCGGCATCACCGGCACCTGGTA
CAACCAGCTCGGCTCGACCTTCATCGTGACCGCGGGCGCCGACGGCGCCCTGACCGGAACCTACGAGTCGGCCGTCGGCAACGCCGAGAGCCGCTACGTCCTGACCGGTCGTTACGACAGCG
CCCCGGCCACCGACGGCAGCGGCACCGCCCTCGGTTGGACGGTGGCCTGGAAGAATAACTACCGCAACGCCCACTCCGCGACCACGTGGAGCGGCCAGTACGTCGGCGGCGCCGAGGCGAG
GATCAACACCCAGTGGCTGCTGACCATGGGCACCACCGAGGCCAACGCCTGGCGCTCCACGCTGGTCGGCCACGACACCTTCACCAAGGTGAAGCCGTCCGCCGCCTCCATCGACGCGGCGAA
GAAGGCCGGCGTCAACAACGGCAACCCGCTCGACGCCGTTCAGCAGTAATAAGGATCCGAATTCGAGCTCCGTCGACAAGCTTGCGGCCGCACTCGAGCACCACCACCACCACCACTGAGATC
CGGCTGCTAACAAAGCCCGAAAGGAAGCTGAGTTGGCTGCTGCCACCGCTGAGCAATAACTAGCATAACCCCTTGGGGCCTCTAAACGGGTCTTGAGGGGTTTTTTGCTGAAAGGAGGAACTA
TATCCGGAT

prhaBAD-MBP-HaloTag

CCAGCTGTCTAGGGCGGCGGATTTGTCCTACTCAGGAGAGCGTTCACCGACAAACAACAGATAAAACGAAAGGCCCAGTCTTTCGACTGAGCCTTTCGTTTTATTTGATGCCTTTAATTAAAGC
GGATAACCACCACAATTCAGCAAATTGTGAACATCATCACGTTCATCTTTCCCTGGTTGCCAATGGCCCATTTTCCTGTCAGTAACGAGAAGGTCGCGAATTCAGGCGCTTTTTAGACTGGTCGT
AATGAAGGGTACCTCTAGAAATAATTTTGTTTAACTTTAAGAAGGAGATATACATATGAAAATAAAAACAGGTGCACGCATCCTCGCATTATCCGCATTAACGACGATGATGTTTTCCGCCTCGG
CTCTCGCCAAAATCGAAGAAGGTAAACTGGTAATCTGGATTAACGGCGATAAAGGCTATAACGGTCTCGCTGAAGTCGGTAAGAAATTCGAGAAAGATACCGGAATTAAAGTCACCGTTGAGC
ATCCGGATAAACTGGAAGAGAAATTCCCACAGGTTGCGGCAACTGGCGATGGCCCTGACATTATCTTCTGGGCACACGACCGCTTTGGTGGCTACGCTCAATCTGGCCTGTTGGCTGAAATCAC
CCCGGACAAAGCGTTCCAGGACAAGCTGTATCCGTTTACCTGGGATGCCGTACGTTACAACGGCAAGCTGATTGCTTACCCGATCGCTGTTGAAGCGTTATCGCTGATTTATAACAAAGATCTG
CTGCCGAACCCGCCAAAAACCTGGGAAGAGATCCCGGCGCTGGATAAAGAACTGAAAGCGAAAGGTAAGAGCGCGCTGATGTTCAACCTGCAAGAACCGTACTTCACCTGGCCGCTGATTGC
TGCTGACGGGGGTTATGCGTTCAAGTATGAAAACGGCAAGTACGACATTAAAGACGTGGGCGTGGATAACGCTGGCGCGAAAGCGGGTCTGACCTTCCTGGTTGACCTGATTAAAAACAAAC
ACATGAATGCAGACACCGATTACTCCATCGCAGAAGCTGCCTTTAATAAAGGCGAAACAGCGATGACCATCAACGGCCCGTGGGCATGGTCCAACATCGACACCAGCAAAGTGAATTATGGTG
TAACGGTACTGCCGACCTTCAAGGGTCAACCATCCAAACCGTTCGTTGGCGTGCTGAGCGCAGGTATTAACGCCGCCAGTCCGAACAAAGAGCTGGCGAAAGAGTTCCTCGAAAACTATCTGC
TGACTGATGAAGGTCTGGAAGCGGTTAATAAAGACAAACCGCTGGGTGCCGTAGCGCTGAAGTCTTACGAGGAAGAGTTGGCGAAAGATCCACGTATTGCCGCCACCATGGAAAACGCCCAG
AAAGGTGAAATCATGCCGAACATCCCGCAGATGTCCGCTTTCTGGTATGCCGTGCGTACTGCGGTGATCAACGCCGCCAGCGGTCGTCAGACTGTCGATGAAGCCCTGAAAGACGCGCAGACT
CGTATCACCAAGCTCGAGCCAACCACTGAGGATCTGTACTTTCAGAGCGATAACGATGGATCCGAAATCGGTACTGGCTTTCCATTCGACCCCCATTATGTGGAAGTCCTGGGCGAGCGCATGC
ACTACGTCGATGTTGGTCCGCGCGATGGCACCCCTGTGCTGTTCCTGCACGGTAACCCGACCTCCTCCTACGTGTGGCGCAACATCATCCCGCATGTTGCACCGACCCATCGCTGCATTGCTCCA
GACCTGATCGGTATGGGCAAATCCGACAAACCAGACCTGGGTTATTTCTTCGACGACCACGTCCGCTTCATGGATGCCTTCATCGAAGCCCTGGGTCTGGAAGAGGTCGTCCTGGTCATTCACG
ACTGGGGCTCCGCTCTGGGTTTCCACTGGGCCAAGCGCAATCCAGAGCGCGTCAAAGGTATTGCATTTATGGAGTTCATCCGCCCTATCCCGACCTGGGACGAATGGCCAGAATTTGCCCGCG
AGACCTTCCAGGCCTTCCGCACCACCGACGTCGGCCGCAAGCTGATCATCGATCAGAACGTTTTTATCGAGGGTACGCTGCCGATGGGTGTCGTCCGCCCGCTGACTGAAGTCGAGATGGACC
ATTACCGCGAGCCGTTCCTGAATCCTGTTGACCGCGAGCCACTGTGGCGCTTCCCAAACGAGCTGCCAATCGCCGGTGAGCCAGCGAACATCGTCGCGCTGGTCGAAGAATACATGGACTGGC
TGCACCAGTCCCCTGTCCCGAAGCTGCTGTTCTGGGGCACCCCAGGCGTTCTGATCCCACCGGCCGAAGCCGCTCGCCTGGCCAAAAGCCTGCCTAACTGCAAGGCTGTGGACATCGGCCCGG
GTCTGAATCTGCTGCAAGAAGACAACCCGGACCTGATCGGCAGCGAGATCGCGCGCTGGCTGTCTACTCTGGAGATTTCCGGTCATCATCACCATCACCACTAACGGCCTCCTGTGTGAAATTG
TTATCCGCTCGACTAGTCTTGGACTCCTGTTGATAGATCCAGTAATGACCTCAGAACTCCATCTGGATTTGTTCAGAACGCTCGGTTGCCGCCGGGCGTTTTTTATTGGTGAGAATCCAGGGGTC
CCCAATAATTACGATTTAAATGATCGGCACGTAAGAGGTTCCAACTTTCACCATAATGAAATAAGATCACTACCGGGCGTATTTTTTGAGTTATCGAGATTTTCAGGAGCTAAGGAAGCTAAAAT
GGAGAAAAAAATCACTGGATATACCACCGTTGATATATCCCAATGGCATCGTAAAGAACATTTTGAGGCATTTCAGTCAGTTGCTCAATGTACCTATAACCAGACCGTTCAGCTGGATATTACG
GCCTTTTTAAAGACCGTAAAGAAAAATAAGCACAAGTTTTATCCGGCCTTTATTCACATTCTTGCCCGCCTGATGAATGCTCATCCGGAATTTCGTATGGCAATGAAAGACGGTGAGCTGGTGAT
ATGGGATAGTGTTCACCCTTGTTACACCGTTTTCCATGAGCAAACTGAAACGTTTTCATCGCTCTGGAGTGAATACCACGACGATTTCCGGCAGTTTCTACACATATATTCGCAAGATGTGGCGT
GTTACGGTGAAAACCTGGCCTATTTCCCTAAAGGGTTTATTGAGAATATGTTTTTCGTCTCAGCCAATCCCTGGGTGAGTTTCACCAGTTTTGATTTAAACGTGGCCAATATGGACAACTTCTTCG
CCCCCGTTTTCACCATGGGCAAATATTATACGCAAGGCGACAAGGTGCTGATGCCGCTGGCGATTCAGGTTCATCATGCCGTTTGTGATGGCTTCCATGTCGGCAGAATGCTTAATGAATTACA
ACAGTACTGCGATGAGTGGCAGGGCGGGGCGTAATAATTAATTGGACAAGGGTCCTTTTCCGCTGCATAACCCTGCTTCGGGGTCATTATAGCGATTTTTTCGGTATATCCATCCTTTTTCGCAC
GATATACAGGATTTTGCCAAAGGGTTCGTGTAGACTTTCCTTGGTGTATCCAACGGCGTCAGCCGGGCAGGATAGGTGAAGTAGGCCCACCCGCGAGCGGGTGTTCCTTCTTCACTGTCCCTTA
TTCGCACCTGGCGGTGCTCAACGGGAATCCTGCTCTGCGAGGCTGGCCGTAGGCCGGCCGGGCAGGATAGGTGAAGTAGGCCCACCCGCGAGCGGGTGTTCCTTCTTCACTGTCCCTTATTCG
CACCTGGCGGTGCTCAACGGGAATCCTGCTCTGCGAGGCTGGCCGTAGGCCGGCCCTAGAAATATTTTATCTGATTAATAAGATGATCTTCTTGAGATCGTTTTGGTCTGCGCGTAATCTCTTGC
TCTGAAAACGAAAAAACCGCCTTGCAGGGCGGTTTTTCGAAGGTTCTCTGAGCTACCAACTCTTTGAACCGAGGTAACTGGCTTGGAGGAGCGCAGTCACCAAAACTTGTCCTTTCAGTTTAGC
CTTAACCGGCGCATGACTTCAAGACTAACTCCTCTAAATCAATTACCAGTGGCTGCTGCCAGTGGTGCTTTTGCATGTCTTTCCGGGTTGGACTCAAGACGATAGTTACCGGATAAGGCGCAGC
GGTCGGACTGAACGGGGGGTTCGTGCATACAGTCCAGCTTGGAGCGAACTGCCTACCCGGAACTGAGTGTCAGGCGTGGAATGAGACAAACGCGGCCATAACAGCGGAATGACACCGGTAA
ACCGAAAGGCAGGAACAGGAGAGCGCACGAGGGAGCCGCCAGGGGGAAACGCCTGGTATCTTTATAGTCCTGTCGGGTTTCGCCACCACTGATTTGAGCGTCAGATTTCGTGATGCTTGTCA
GGGGGGCGGAGCCTATGGAAAAACGGCTTTGCCGCGGCCCTCTCACTTCCCTGTTAAGTATCTTCCTGGCATCTTCCAGGAAATCTCCGCCCCGTTCGTAAGCCATTTCCGCTCGCCGCAGTCGA
ACGACCGAGCGTAGCGAGTCAGTGAGCGAGGAAGCGGAATATATCCGGCGCGCCGGCGCGC

pKD4

AGATTGCAGCATTACACGTCTTGAGCGATTGTGTAGGCTGGAGCTGCTTCGAAGTTCCTATACTTTCTAGAGAATAGGAACTTCGGAATAGGAACTTCAAGATCCCCTCACGCTGCCGCAAGCA
CTCAGGGCGCAAGGGCTGCTAAAGGAAGCGGAACACGTAGAAAGCCAGTCCGCAGAAACGGTGCTGACCCCGGATGAATGTCAGCTACTGGGCTATCTGGACAAGGGAAAACGCAAGCGCA
AAGAGAAAGCAGGTAGCTTGCAGTGGGCTTACATGGCGATAGCTAGACTGGGCGGTTTTATGGACAGCAAGCGAACCGGAATTGCCAGCTGGGGCGCCCTCTGGTAAGGTTGGGAAGCCCT
GCAAAGTAAACTGGATGGCTTTCTTGCCGCCAAGGATCTGATGGCGCAGGGGATCAAGATCTGATCAAGAGACAGGATGAGGATCGTTTCGCATGATTGAACAAGATGGATTGCACGCAGGT
TCTCCGGCCGCTTGGGTGGAGAGGCTATTCGGCTATGACTGGGCACAACAGACAATCGGCTGCTCTGATGCCGCCGTGTTCCGGCTGTCAGCGCAGGGGCGCCCGGTTCTTTTTGTCAAGACC
GACCTGTCCGGTGCCCTGAATGAACTGCAGGACGAGGCAGCGCGGCTATCGTGGCTGGCCACGACGGGCGTTCCTTGCGCAGCTGTGCTCGACGTTGTCACTGAAGCGGGAAGGGACTGGCT
GCTATTGGGCGAAGTGCCGGGGCAGGATCTCCTGTCATCTCACCTTGCTCCTGCCGAGAAAGTATCCATCATGGCTGATGCAATGCGGCGGCTGCATACGCTTGATCCGGCTACCTGCCCATTC
GACCACCAAGCGAAACATCGCATCGAGCGAGCACGTACTCGGATGGAAGCCGGTCTTGTCGATCAGGATGATCTGGACGAAGAGCATCAGGGGCTCGCGCCAGCCGAACTGTTCGCCAGGCT
CAAGGCGCGCATGCCCGACGGCGAGGATCTCGTCGTGACCCATGGCGATGCCTGCTTGCCGAATATCATGGTGGAAAATGGCCGCTTTTCTGGATTCATCGACTGTGGCCGGCTGGGTGTGGC
GGACCGCTATCAGGACATAGCGTTGGCTACCCGTGATATTGCTGAAGAGCTTGGCGGCGAATGGGCTGACCGCTTCCTCGTGCTTTACGGTATCGCCGCTCCCGATTCGCAGCGCATCGCCTTC
TATCGCCTTCTTGACGAGTTCTTCTGAGCGGGACTCTGGGGTTCGAAATGACCGACCAAGCGACGCCCAACCTGCCATCACGAGATTTCGATTCCACCGCCGCCTTCTATGAAAGGTTGGGCTTC
GGAATCGTTTTCCGGGACGCCGGCTGGATGATCCTCCAGCGCGGGGATCTCATGCTGGAGTTCTTCGCCCACCCCAGCTTCAAAAGCGCTCTGAAGTTCCTATACTTTCTAGAGAATAGGAACT
TCGGAATAGGAACTAAGGAGGATATTCATATGGACCATGGCTAATTCCCATGTCAGCCGTTAAGTGTTCCTGTGTCACTGAAAATTGCTTTGAGAGGCTCTAAGGGCTTCTCAGTGCGTTACAT
CCCTGGCTTGTTGTCCACAACCGTTAAACCTTAAAAGCTTTAAAAGCCTTATATATTCTTTTTTTTCTTATAAAACTTAAAACCTTAGAGGCTATTTAAGTTGCTGATTTATATTAATTTTATTGTTCA
AACATGAGAGCTTAGTACGTGAAACATGAGAGCTTAGTACGTTAGCCATGAGAGCTTAGTACGTTAGCCATGAGGGTTTAGTTCGTTAAACATGAGAGCTTAGTACGTTAAACATGAGAGCTT
AGTACGTGAAACATGAGAGCTTAGTACGTACTATCAACAGGTTGAACTGCGGATCTTGCGGCCGCAAAAATTAAAAATGAAGTTTTAAATCAATCTAAAGTATATATGAGTAAACTTGGTCTGA
CAGTTACCAATGCTTAATCAGTGAGGCACCTATCTCAGCGATCTGTCTATTTCGTTCATCCATAGTTGCCTGACTCCCCGTCGTGTAGATAACTACGATACGGGAGGGCTTACCATCTGGCCCCA
GTGCTGCAATGATACCGCGAGACCCACGCTCACCGGCTCCAGATTTATCAGCAATAAACCAGCCAGCCGGAAGGGCCGAGCGCAGAAGTGGTCCTGCAACTTTATCCGCCTCCATCCAGTCTAT
TAATTGTTGCCGGGAAGCTAGAGTAAGTAGTTCGCCAGTTAATAGTTTGCGCAACGTTGTTGCCATTGCTACAGGCATCGTGGTGTCACGCTCGTCGTTTGGTATGGCTTCATTCAGCTCCGGTT
CCCAACGATCAAGGCGAGTTACATGATCCCCCATGTTGTGCAAAAAAGCGGTTAGCTCCTTCGGTCCTCCGATCGTTGTCAGAAGTAAGTTGGCCGCAGTGTTATCACTCATGGTTATGGCAGC
ACTGCATAATTCTCTTACTGTCATGCCATCCGTAAGATGCTTTTCTGTGACTGGTGAGTACTCAACCAAGTCATTCTGAGAATAGTGTATGCGGCGACCGAGTTGCTCTTGCCCGGCGTCAATAC
GGGATAATACCGCGCCACATAGCAGAACTTTAAAAGTGCTCATCATTGGAAAACGTTCTTCGGGGCGAAAACTCTCAAGGATCTTACCGCTGTTGAGATCCAGTTCGATGTAACCCACTCGTGC
ACCCAACTGATCTTCAGCATCTTTTACTTTCACCAGCGTTTCTGGGTGAGCAAAAACAGGAAGGCAAAATGCCGCAAAAAAGGGAATAAGGGCGACACGGAAATGTTGAATACTCATACTCTTC
CTTTTTCAATATTATTGAAGCATTTATCAGGGTTATTGTCTCATGAGCGGATACATATTTGAATGTATTTAGAAAAATAAACAAATAGGGGTTCCGCGCACATTTCCCCGAAAAGTGCCACCTGCA
TCGATGGCCCCCCGATGGTAGTGTGGGGTCTCCCCATGCGAGAGTAGGGAACTGCCAGGCATCAAATAAAACGAAAGGCTCAGTCGAAAGACTGGGCCTTTCGTTTTATCTGTTGTTTGTCGG
TGAACGCTCTCCTGAGTAGGACAAATCCGCCGGGAGCGGATTTGAACGTTGCGAAGCAACGGCCCGGAGGGTGGCGGGCAGGACGCCCGCCATAAACTGCCAGGCATCAAATTAAGCAGAA
GGCCATCCTGACGGATGGCCTTTTTGCGTGGCCAGTGCCAAGCTTGCATGC

pKD46

CATCGATTTATTATGACAACTTGACGGCTACATCATTCACTTTTTCTTCACAACCGGCACGGAACTCGCTCGGGCTGGCCCCGGTGCATTTTTTAAATACCCGCGAGAAATAGAGTTGATCGTCAA
AACCAACATTGCGACCGACGGTGGCGATAGGCATCCGGGTGGTGCTCAAAAGCAGCTTCGCCTGGCTGATACGTTGGTCCTCGCGCCAGCTTAAGACGCTAATCCCTAACTGCTGGCGGAAAA
GATGTGACAGACGCGACGGCGACAAGCAAACATGCTGTGCGACGCTGGCGATATCAAAATTGCTGTCTGCCAGGTGATCGCTGATGTACTGACAAGCCTCGCGTACCCGATTATCCATCGGTG
GATGGAGCGACTCGTTAATCGCTTCCATGCGCCGCAGTAACAATTGCTCAAGCAGATTTATCGCCAGCAGCTCCGAATAGCGCCCTTCCCCTTGCCCGGCGTTAATGATTTGCCCAAACAGGTCG
CTGAAATGCGGCTGGTGCGCTTCATCCGGGCGAAAGAACCCCGTATTGGCAAATATTGACGGCCAGTTAAGCCATTCATGCCAGTAGGCGCGCGGACGAAAGTAAACCCACTGGTGATACCAT
TCGCGAGCCTCCGGATGACGACCGTAGTGATGAATCTCTCCTGGCGGGAACAGCAAAATATCACCCGGTCGGCAAACAAATTCTCGTCCCTGATTTTTCACCACCCCCTGACCGCGAATGGTGA
GATTGAGAATATAACCTTTCATTCCCAGCGGTCGGTCGATAAAAAAATCGAGATAACCGTTGGCCTCAATCGGCGTTAAACCCGCCACCAGATGGGCATTAAACGAGTATCCCGGCAGCAGGG
GATCATTTTGCGCTTCAGCCATACTTTTCATACTCCCGCCATTCAGAGAAGAAACCAATTGTCCATATTGCATCAGACATTGCCGTCACTGCGTCTTTTACTGGCTCTTCTCGCTAACCAAACCGGT
AACCCCGCTTATTAAAAGCATTCTGTAACAAAGCGGGACCAAAGCCATGACAAAAACGCGTAACAAAAGTGTCTATAATCACGGCAGAAAAGTCCACATTGATTATTTGCACGGCGTCACACTT
TGCTATGCCATAGCATTTTTATCCATAAGATTAGCGGATCCTACCTGACGCTTTTTATCGCAACTCTCTACTGTTTCTCCATACCCGTTTTTTTGGGAATTCGAGCTCTAAGGAGGTTATAAAAAAT
GGATATTAATACTGAAACTGAGATCAAGCAAAAGCATTCACTAACCCCCTTTCCTGTTTTCCTAATCAGCCCGGCATTTCGCGGGCGATATTTTCACAGCTATTTCAGGAGTTCAGCCATGAACGC
TTATTACATTCAGGATCGTCTTGAGGCTCAGAGCTGGGCGCGTCACTACCAGCAGCTCGCCCGTGAAGAGAAAGAGGCAGAACTGGCAGACGACATGGAAAAAGGCCTGCCCCAGCACCTGT
TTGAATCGCTATGCATCGATCATTTGCAACGCCACGGGGCCAGCAAAAAATCCATTACCCGTGCGTTTGATGACGATGTTGAGTTTCAGGAGCGCATGGCAGAACACATCCGGTACATGGTTGA
AACCATTGCTCACCACCAGGTTGATATTGATTCAGAGGTATAAAACGAATGAGTACTGCACTCGCAACGCTGGCTGGGAAGCTGGCTGAACGTGTCGGCATGGATTCTGTCGACCCACAGGAA
CTGATCACCACTCTTCGCCAGACGGCATTTAAAGGTGATGCCAGCGATGCGCAGTTCATCGCATTACTGATCGTTGCCAACCAGTACGGCCTTAATCCGTGGACGAAAGAAATTTACGCCTTTCC
TGATAAGCAGAATGGCATCGTTCCGGTGGTGGGCGTTGATGGCTGGTCCCGCATCATCAATGAAAACCAGCAGTTTGATGGCATGGACTTTGAGCAGGACAATGAATCCTGTACATGCCGGAT
TTACCGCAAGGACCGTAATCATCCGATCTGCGTTACCGAATGGATGGATGAATGCCGCCGCGAACCATTCAAAACTCGCGAAGGCAGAGAAATCACGGGGCCGTGGCAGTCGCATCCCAAAC
GGATGTTACGTCATAAAGCCATGATTCAGTGTGCCCGTCTGGCCTTCGGATTTGCTGGTATCTATGACAAGGATGAAGCCGAGCGCATTGTCGAAAATACTGCATACACTGCAGAACGTCAGCC
GGAACGCGACATCACTCCGGTTAACGATGAAACCATGCAGGAGATTAACACTCTGCTGATCGCCCTGGATAAAACATGGGATGACGACTTATTGCCGCTCTGTTCCCAGATATTTCGCCGCGAC
ATTCGTGCATCGTCAGAACTGACACAGGCCGAAGCAGTAAAAGCTCTTGGATTCCTGAAACAGAAAGCCGCAGAGCAGAAGGTGGCAGCATGACACCGGACATTATCCTGCAGCGTACCGGG
ATCGATGTGAGAGCTGTCGAACAGGGGGATGATGCGTGGCACAAATTACGGCTCGGCGTCATCACCGCTTCAGAAGTTCACAACGTGATAGCAAAACCCCGCTCCGGAAAGAAGTGGCCTGA
CATGAAAATGTCCTACTTCCACACCCTGCTTGCTGAGGTTTGCACCGGTGTGGCTCCGGAAGTTAACGCTAAAGCACTGGCCTGGGGAAAACAGTACGAGAACGACGCCAGAACCCTGTTTGA
ATTCACTTCCGGCGTGAATGTTACTGAATCCCCGATCATCTATCGCGACGAAAGTATGCGTACCGCCTGCTCTCCCGATGGTTTATGCAGTGACGGCAACGGCCTTGAACTGAAATGCCCGTTTA
CCTCCCGGGATTTCATGAAGTTCCGGCTCGGTGGTTTCGAGGCCATAAAGTCAGCTTACATGGCCCAGGTGCAGTACAGCATGTGGGTGACGCGAAAAAATGCCTGGTACTTTGCCAACTATG
ACCCGCGTATGAAGCGTGAAGGCCTGCATTATGTCGTGATTGAGCGGGATGAAAAGTACATGGCGAGTTTTGACGAGATCGTGCCGGAGTTCATCGAAAAAATGGACGAGGCACTGGCTGAA
ATTGGTTTTGTATTTGGGGAGCAATGGCGATGACGCATCCTCACGATAATATCCGGGTAGGCGCAATCACTTTCGTCTACTCCGTTACAAAGCGAGGCTGGGTATTTCCCGGCCTTTCTGTTATC
CGAAATCCACTGAAAGCACAGCGGCTGGCTGAGGAGATAAATAATAAACGAGGGGCTGTATGCACAAAGCATCTTCTGTTGAGTTAAGAACGAGTATCGAGATGGCACATAGCCTTGCTCAA
ATTGGAATCAGGTTTGTGCCAATACCAGTAGAAACAGACGAAGAATCCATGGGTATGGACAGTTTTCCCTTTGATATGTAACGGTGAACAGTTGTTCTACTTTTGTTTGTTAGTCTTGATGCTTC
ACTGATAGATACAAGAGCCATAAGAACCTCAGATCCTTCCGTATTTAGCCAGTATGTTCTCTAGTGTGGTTCGTTGTTTTTGCGTGAGCCATGAGAACGAACCATTGAGATCATACTTACTTTGCA
TGTCACTCAAAAATTTTGCCTCAAAACTGGTGAGCTGAATTTTTGCAGTTAAAGCATCGTGTAGTGTTTTTCTTAGTCCGTTACGTAGGTAGGAATCTGATGTAATGGTTGTTGGTATTTTGTCAC
CATTCATTTTTATCTGGTTGTTCTCAAGTTCGGTTACGAGATCCATTTGTCTATCTAGTTCAACTTGGAAAATCAACGTATCAGTCGGGCGGCCTCGCTTATCAACCACCAATTTCATATTGCTGTA
AGTGTTTAAATCTTTACTTATTGGTTTCAAAACCCATTGGTTAAGCCTTTTAAACTCATGGTAGTTATTTTCAAGCATTAACATGAACTTAAATTCATCAAGGCTAATCTCTATATTTGCCTTGTGAG
TTTTCTTTTGTGTTAGTTCTTTTAATAACCACTCATAAATCCTCATAGAGTATTTGTTTTCAAAAGACTTAACATGTTCCAGATTATATTTTATGAATTTTTTTAACTGGAAAAGATAAGGCAATATC
TCTTCACTAAAAACTAATTCTAATTTTTCGCTTGAGAACTTGGCATAGTTTGTCCACTGGAAAATCTCAAAGCCTTTAACCAAAGGATTCCTGATTTCCACAGTTCTCGTCATCAGCTCTCTGGTTG
CTTTAGCTAATACACCATAAGCATTTTCCCTACTGATGTTCATCATCTGAGCGTATTGGTTATAAGTGAACGATACCGTCCGTTCTTTCCTTGTAGGGTTTTCAATCGTGGGGTTGAGTAGTGCCA
CACAGCATAAAATTAGCTTGGTTTCATGCTCCGTTAAGTCATAGCGACTAATCGCTAGTTCATTTGCTTTGAAAACAACTAATTCAGACATACATCTCAATTGGTCTAGGTGATTTTAATCACTATA
CCAATTGAGATGGGCTAGTCAATGATAATTACTAGTCCTTTTCCTTTGAGTTGTGGGTATCTGTAAATTCTGCTAGACCTTTGCTGGAAAACTTGTAAATTCTGCTAGACCCTCTGTAAATTCCGC
TAGACCTTTGTGTGTTTTTTTTGTTTATATTCAAGTGGTTATAATTTATAGAATAAAGAAAGAATAAAAAAAGATAAAAAGAATAGATCCCAGCCCTGTGTATAACTCACTACTTTAGTCAGTTCC
GCAGTATTACAAAAGGATGTCGCAAACGCTGTTTGCTCCTCTACAAAACAGACCTTAAAACCCTAAAGGCTTAAGTAGCACCCTCGCAAGCTCGGTTGCGGCCGCAATCGGGCAAATCGCTGAA
TATTCCTTTTGTCTCCGACCATCAGGCACCTGAGTCGCTGTCTTTTTCGTGACATTCAGTTCGCTGCGCTCACGGCTCTGGCAGTGAATGGGGGTAAATGGCACTACAGGCGCCTTTTATGGATTC
ATGCAAGGAAACTACCCATAATACAAGAAAAGCCCGTCACGGGCTTCTCAGGGCGTTTTATGGCGGGTCTGCTATGTGGTGCTATCTGACTTTTTGCTGTTCAGCAGTTCCTGCCCTCTGATTTT
CCAGTCTGACCACTTCGGATTATCCCGTGACAGGTCATTCAGACTGGCTAATGCACCCAGTAAGGCAGCGGTATCATCAACGGGGTCTGACGCTCAGTGGAACGAAAACTCACGTTAAGGGAT
TTTGGTCATGAGATTATCAAAAAGGATCTTCACCTAGATCCTTTTAAATTAAAAATGAAGTTTTAAATCAATCTAAAGTATATATGAGTAAACTTGGTCTGACAGTTACCAATGCTTAATCAGTGA
GGCACCTATCTCAGCGATCTGTCTATTTCGTTCATCCATAGTTGCCTGACTCCCCGTCGTGTAGATAACTACGATACGGGAGGGCTTACCATCTGGCCCCAGTGCTGCAATGATACCGCGAGACC
CACGCTCACCGGCTCCAGATTTATCAGCAATAAACCAGCCAGCCGGAAGGGCCGAGCGCAGAAGTGGTCCTGCAACTTTATCCGCCTCCATCCAGTCTATTAATTGTTGCCGGGAAGCTAGAGT
AAGTAGTTCGCCAGTTAATAGTTTGCGCAACGTTGTTGCCATTGCTACAGGCATCGTGGTGTCACGCTCGTCGTTTGGTATGGCTTCATTCAGCTCCGGTTCCCAACGATCAAGGCGAGTTACAT
GATCCCCCATGTTGTGCAAAAAAGCGGTTAGCTCCTTCGGTCCTCCGATCGTTGTCAGAAGTAAGTTGGCCGCAGTGTTATCACTCATGGTTATGGCAGCACTGCATAATTCTCTTACTGTCATG
CCATCCGTAAGATGCTTTTCTGTGACTGGTGAGTACTCAACCAAGTCATTCTGAGAATAGTGTATGCGGCGACCGAGTTGCTCTTGCCCGGCGTCAATACGGGATAATACCGCGCCACATAGCA
GAACTTTAAAAGTGCTCATCATTGGAAAACGTTCTTCGGGGCGAAAACTCTCAAGGATCTTACCGCTGTTGAGATCCAGTTCGATGTAACCCACTCGTGCACCCAACTGATCTTCAGCATCTTTTA
CTTTCACCAGCGTTTCTGGGTGAGCAAAAACAGGAAGGCAAAATGCCGCAAAAAAGGGAATAAGGGCGACACGGAAATGTTGAATACTCATACTCTTCCTTTTTCAATATTATTGAAGCATTTA
TCAGGGTTATTGTCTCATGAGCGGATACATATTTGAATGTATTTAGAAAAATAAACAAATAGGGGTTCCGCGCACATTTCCCCGAAAAGTGCCACCTG

pCP20

GAATTCCGGATGAGCATTCATCAGGCGGGCAAGAATGTGAATAAAGGCCGGATAAAACTTGTGCTTATTTTTCTTTACGGTCTTTAAAAAGGCCGTAATATCCAGCTGAACGGTCTGGTTATAG
GTACATTGAGCAACTGACTGAAATGCCTCAAAATGTTCTTTACGATGCCATTGGGATATATCAACGGTGGTATATCCAGTGATTTTTTTCTCCATTTTAGCTTCCTTAGCTCCTGAAAATCTCGATA
ACTCAAAAAATACGCCCGGTAGTGATCTTATTTCATTATGGTGAAAGTTGGAACCTCTTACGTGCCGATCAACGTCTCATTTTCGCCAAAAGTTGGCCCAGGGCTTCCCGGTATCAACAGGGACA
CCAGGATTTATTTATTCTGCGAAGTGATCTTCCGTCACAGGTATTTATTCGGCGCAAAGTGCGTCGGGTGATGCTGCCAACTTACTGATTTAGTGTATGATGGTGTTTTTGAGGTGCTCCAGTGG
CTTCTGTTTCTATCAGCTGTCCCTCCTGTTCAGCTACTGACGGGGTGGTGCGTAACGGCAAAAGCACCGCCGGACATCAGCGCCTGTAGTGCCATTTACCCCCATTCACTGCCAGAGCCGTGAGC
GCAGCGAACTGAATGTCACGAAAAAGACAGCGACTCAGGTGCCTGATGGTCGGAGACAAAAGGAATATTCAGCGATTTGCCCGAGCTTGCGAGGGTGCTACTTAAGCCTTTAGGGTTTTAAG
GTCTGTTTTGTAGAGGAGCAAACAGCGTTTGCGACATCCTTTTGTAATACTGCGGAACTGACTAAAGTAGTGAGTTATACACAGGGCTGGGATCTATTCTTTTTATCTTTTTTTATTCTTTCTTTAT
TCTATAAATTATAACCACTTGAATATAAACAAAAAAAACACACAAAGGTCTAGCGGAATTTACAGAGGGTCTAGCAGAATTTACAAGTTTTCCAGCAAAGGTCTAGCAGAATTTACAGATACCC
ACAACTCAAAGGAAAAGGACTAGTAATTATCATTGACTAGCCCATCTCAATTGGTATAGTGATTAAAATCACCTAGACCAATTGAGATGTATGTCTGAATTAGTTGTTTTCAAAGCAAATGAACT
AGCGATTAGTCGCTATGACTTAACGGAGCATGAAACCAAGCTAATTTTATGCTGTGTGGCACTACTCAACCCCACGATTGAAAACCCTACAAGGAAAGAACGGACGGTATCGTTCACTTATAAC
CAATACGTTCAGATGATGAACATCAGTAGGGAAAATGCTTATGGTGTATTAGCTAAAGCAACCAGAGAGCTGATGACGAGAACTGTGGAAATCAGGAATCCTTTGGTTAAAGGCTTTGAGATT
TTCCAGTGGACAAACTATGCCAAGTTCTCAAGCGAAAAATTAGAATTAGTTTTTAGTGAAGAGATATTGCCTTATCTTTTCCAGTTAAAAAAATTCATAAAATATAATCTGGAACATGTTAAGTCT
TTTGAAAACAAATACTCTATGAGGATTTATGAGTGGTTATTAAAAGAACTAACACAAAAGAAAACTCACAAGGCAAATATAGAGATTAGCCTTGATGAATTTAAGTTCATGTTAATGCTTGAAA
ATAACTACCATGAGTTTAAAAGGCTTAACCAATGGGTTTTGAAACCAATAAGTAAAGATTTAAACACTTACAGCAATATGAAATTGGTGGTTGATAAGCGAGGCCGCCCGACTGATACGTTGAT
TTTCCAAGTTGAACTAGATAGACAAATGGATCTCGTAACCGAACTTGAGAACAACCAGATAAAAATGAATGGTGACAAAATACCAACAACCATTACATCAGATTCCTACCTACGTAACGGACTA
AGAAAAACACTACACGATGCTTTAACTGCAAAAATTCAGCTCACCAGTTTTGAGGCAAAATTTTTGAGTGACATGCAAAGTAAGCATGATCTCAATGGTTCGTTCTCATGGCTCACGCAAAAACA
ACGAACCACACTAGAGAACATACTGGCTAAATACGGAAGGATCTGAGGTTCTTATGGCTCTTGTATCTATCAGTGAAGCATCAAGACTAACAAACAAAAGTAGAACAACTGTTCACCGTTAGAT
ATCAAAGGGAAAACTGTCCATATGCACAGATGAAAACGGTGTAAAAAAGATAGATACATCAGAGCTTTTACGAGTTTTTGGTGCATTTAAAGCTGTTCACCATGAACAGATCGACAATGTAACA
GATGAACAGCATGTAACACCTAATAGAACAGGTGAAACCAGTAAAACAAAGCAACTAGAACATGAAATTGAACACCTGAGACAACTTGTTACAGCTCAACAGTCACACATAGACAGCCTGAAA
CAGGCGATGCTGCTTATCGAATCAAAGCTGCCGACAACACGGGAGCCAGTGACGCCTCCCGTGGGGAAAAAATCATGGCAATTCTGGAAGAAAATAGCGCCTGTTTCGTTTCAGGCAGGTTAT
CAGGGAGTGTCAGCGTCCTGCGGTTCTCCGGGGCGTTCGGGTCATGCAGCCCGTAATGGTGATTTAACAGCGTCTGCCAAGCATCAATTCTAGGCCTGTCTGCGCGGTCGTAGTACGGCTGGA
GGCGTTTTCCGGTCTGTAGCTCCATGTTCGGAATGACAAAATTCAGCTCAAGCCGTCCCTTGTCCTGGTGCTCCACCCACAGGATGCTGTACTGATTTTTTTCGAGACCGGGCATCAGTACACGC
TCAAAGCTCGCCATCACTTTTTCACGTCCTCCCGGCGGCAGCTCCTTCTCCGCGAACGACAGAACACCGGACGTGTATTTCTTCGCAAATGGCGTGGCATCGATGAGTTCCCGGACTTCTTCCGG
ATTACCCTGAAGCACCGTTGCGCCTTCGCGGTTACGCTCCCTCCCCAGCAGGTAATCAACCGGACCACTGCCACCACCTTTTCCCCTGGCATGAAATTTAACTATCATCCCGCGCCCCCTGTTCCC
TGACAGCCAGACGCAGCCGGCGCAGCTCATCCCCGATGGCCATCAGTGCGGCCACCACCTGAACCCGGTCACCGGAAGACCACTGCCCGCTGTTCACCTTACGGGCTGTCTGATTCAGGTTATT
TCCGATGGCGGCCAGCTGACGCAGTAACGGCGGTGCCAGTGTCGGCAGTTTTCCGGAACGGGCAACCGGCTCCCCCAGGCAGACCCGCCGCATCCATACCGCCAGTTGTTTACCCTCACAGCG
TTCAAGTAACCGGGCATGTTCATCATCAGTAACCCGTATTGTGAGCATCCTCTCGCGTTTCATCGGTATCATTACCCCCATGAACAGAAATCCCCCTTACACGGAGGCATCAGTGACTAAACGGGG
TCTGACGCTCAGTGGAACGAAAACTCACGTTAAGGGATTTTGGTCATGAGATTATCAAAAAGGATCTTCACCTAGATCCTTTTAAATTAAAAATGAAGTTTTAAATCAATCTAAAGTATATATGA
GTAAACTTGGTCTGACAGTTACCAATGCTTAATCAGTGAGGCACCTATCTCAGCGATCTGTCTATTTCGTTCATCCATAGTTGCCTGACTCCCCGTCGTGTAGATAACTACGATACGGGAGGGCT
TACCATCTGGCCCCAGTGCTGCAATGATACCGCGAGACCCACGCTCACCGGCTCCAGATTTATCAGCAATAAACCAGCCAGCCGGAAGGGCCGAGCGCAGAAGTGGTCCTGCAACTTTATCCG
CCTCCATCCAGTCTATTAATTGTTGCCGGGAAGCTAGAGTAAGTAGTTCGCCAGTTAATAGTTTGCGCAACGTTGTTGCCATTGCTGCAGGCATCGTGGTGTCACGCTCGTCGTTTGGTATGGCT
TCATTCAGCTCCGGTTCCCAACGATCAAGGCGAGTTACATGATCCCCCATGTTGTGCAAAAAAGCGGTTAGCTCCTTCGGTCCTCCGATCGTTGTCAGAAGTAAGTTGGCCGCAGTGTTATCACT
CATGGTTATGGCAGCACTGCATAATTCTCTTACTGTCATGCCATCCGTAAGATGCTTTTCTGTGACTGGTGAGTACTCAACCAAGTCATTCTGAGAATAGTGTATGCGGCGACCGAGTTGCTCTT
GCCCGGCGTCAACACGGGATAATACCGCGCCACATAGCAGAACTTTAAAAGTGCTCATCATTGGAAAACGTTCTTCGGGGCGAAAACTCTCAAGGATCTTACCGCTGTTGAGATCCAGTTCGAT
GTAACCCACTCGTGCACCCAACTGATCTTCAGCATCTTTTACTTTCACCAGCGTTTCTGGGTGAGCAAAAACAGGAAGGCAAAATGCCGCAAAAAAGGGAATAAGGGCGACACGGAAATGTTG
AATACTCATACTCTTCCTTTTTCAATATTATTGAAGCATTTATCAGGGTTATTGTCTCATGAGCGGATACATATTTGAATGTATTTAGAAAAATAAACAAATAGGGGTTCCGCGCACATTTCCCCG
AAAAGTGCCACCTGACGTCTAAGAAACCATTATTATCATGACATTAACCTATAAAAATAGGCGTATCACGAGGCCCTTTCGTCTTCAAGAATTTTATAAACCGTGGAGCGGGCAATACTGAGCT
GATGAGCAATTTCCGTTGCACCAGTGCCCTTCTGATGAAGCGTCAGCACGACGTTCCTGTCCACGGTACGCCTGCGGCCAAATTTGATTCCTTTCAGCTTTGCTTCCTGTCGGCCCTCATTCGTGC
GCTCTAGGATCCTCTACGCCGGACGCATCGTGGCCGGCATCACCGGCGCTGAGGTCTGCCTCGTGAAGAAGGTGTTGCTGACTCATACCAGGCCTGAATCGCCCCATCATCCAGCCAGAAAGT
GAGGGAGCCACGGTTGATGAGAGCTTTGTTGTAGGTGGACCAGTTGGTGATTTTGAACTTTTGCTTTGCCACGGAACGGTCTGCGTTGTCGGGAAGATGCGTGATCTGATCCTTCAACTCAGCA
AAAGTTCGATTTATTCAACAAAGCCGCCGTCCCGTCAAGTCAGCGTAATGCTCTGCCAGTGTTACAACCAATTAACCAATTCTGATTAGAAAAACTCATCGAGCATCAAATGAAACTGCAATTTA
TTCATATCAGGATTATCAATACCATATTTTTGAAAAAGCCGTTTCTGTAATGAAGGAGAAAACTCACCGAGGCAGTTCCATAGGATGGCAAGATCCTGGTATCGGTCTGCGATTCCGACTCGTCC
AACATCAATACAACCTATTAATTTCCCCTCGTCAAAAATAAGGTTATCAAGTGAGAAATCACCATGAGTGACGACTGAATCCGGTGAGAATGGCAAAAGCTTCTAGAGGATCCCCCTAGAGAAT
AGGAACTTCGGAATAGGAACTTCAAAGCGTTTCCGAAAACGAGCGCTTCCGAAAATGCAACGCGAGCTGCGCACATACAGCTCACTGTTCACGTCGCACCTATATCTGCGTGTTGCCTGTATAT
ATATATACATGAGAAGAACGGCATAGTGCGTGTTTATGCTTAAATGCGTACTTATATGCGTCTATTTATGTAGGATGAAAGGTAGTCTAGTACCTCCTGTGATATTATCCCATTCCATGCGGGGT
ATCGTATGCTTCCTTCAGCACTACCCTTTAGCTGTTCTATATGCTGCCACTCCTCAATTGGATTAGTCTCATCCTTCAATGCTATCATTTCCTTTGATATTGGATCATATGCATAGTACCGAGAAACT
AGTGCGAAGTAGTGATCAGGTATTGCTGTTATCTGATGAGTATACGTTGTCCTGGCCACGGCAGAAGCACGCTTATCGCTCCAATTTCCCACAACATTAGTCAACTCCGTTAGGCCCTTCATTGA
AAGAAATGAGGTCATCAAATGTCTTCCAATGTGAGATTTTGGGCCATTTTTTATAGCAAAGATTGAATAAGGCGCATTTTTCTTCAAAGCTTTATTGTACGATCTGACTAAGTTATCTTTTAATAA
TTGGTATTCCTGTTTATTGCTTGAAGAATTGCCGGTCCTATTTACTCGTTTTAGGACTGGTTCAGAATTCCTCAAAAATTCATCCAAATATACAAGTGGATCGATCCTACCCCTTGCGCTAAAGAA
GTATATGTGCCTACTAACGCTTGTCTTTGTCTCTGTCACTAAACACTGGATTATTACTCCCAGATACTTATTTTGGACTAATTTAAATGATTTCGGATCAACGTTCTTAATATCGCTGAATCTTCCAC
AATTGATGAAAGTAGCTAGGAAGAGGAATTGGTATAAAGTTTTTGTTTTTGTAAATCTCGAAGTATACTCAAACGAATTTAGTATTTTCTCAGTGATCTCCCAGATGCTTTCACCCTCACTTAGAA
GTGCTTTAAGCATTTTTTTACTGTGGCTATTTCCCTTATCTGCTTCTTCCGATGATTCGAACTGTAATTGCAAACTACTTACAATATCAGTGATATCAGATTGATGTTTTTGTCCATAGTAAGGAATA
ATTGTAAATTCCCAAGCAGGAATCAATTTCTTTAATGAGGCTTCCAGAATTGTTGCTTTTTGCGTCTTGTATTTAAACTGGAGTGATTTATTGACAATATCGAAACTCAGCGAATTGCTTATGATA
GTATTATAGCTCATGAATGTGGCTCTCTTGATTGCTGTTCCGTTATGTGTAATCATCCAACATAAATAGGTTAGTTCAGCAGCACATAATGCTATTTTCTCACCTGAAGGTCTTTCAAACCTTTCCA
CAAACTGACGAACAAGCACCTTAGGTGGTGTTTTACATAATATACCAAATTGTGGCATACAACCTCCTTAGTACATGCAACCATTATCACCGCCAGAGGTAAAATAGTCAACACGCACGGTGTTA
GATATTTATCCCTTGCGGTGATAGATTTAACGTATGAGCACAAAAAAGAAACCATTAACACAAGAGCAGCTTGAGGACGCACGTCGCCTTAAAGCAATTTATGAAAAAAAGAAAAATGAACTT
GGCTTATCCCAGGAATCTGTCGCAGACAAGATGGGGATGGGGCAGTCAGGCGTTGGTGCTTTATTTAATGGCATCAATGCATTAAATGCTTATAACGCCGCATTGCTTACAAAAATTCTCAAAG
TTAGCGTTGAAGAATTTAGCCCTTCAATCGCCAGAGAAATCTACGAGATGTATGAAGCGGTTAGTATGCAGCCGTCACTTAGAAGTGAGTATGAGTACCCTGTTTTTTCTCATGTTCAGGCAGG
GATGTTCTCACCTAAGCTTAGAACCTTTACCAAAGGTGATGCGGAGAGATGGGTAAGCACAACCAAAAAAGCCAGTGATTCTGCATTCTGGCTTGAGGTTGAAGGTAATTCCATGACCGCACC
AACAGGCTCCAAGCCAAGCTTTCCTGACGGAATGTTAATTCTCGTTGACCCTGAGCAGGCTGTTGAGCCAGGTGATTTCTGCATAGCCAGACTTGGGGGTGATGAGTTTACCTTCAAGAAACTG
ATCAGGGATAGCGGTCAGGTGTTTTTACAACCACTAAACCCACAGTACCCAATGATCCCATGCAATGAGAGTTGTTCCGTTGTGGGGAAAGTTATCGCTAGTCAGTGGCCTGAAGAGACGTTTG
GCTGATCGGCAAGGTGTTCTGGTCGGCGCATAGCTGATAACAATTGAGCAAGAATCTTCATCGAATTAGGGGAATTTTCACTCCCCTCAGAACATAACATAGTAAATGGATTGAATTATGAAGA
ATGGTTTTTATGCGACTTACCGCAGCAAAAATAAAGGGAAAGATAAGCGCTCAATAAACCTGTCTGTTTTCCTTAATTCTCTGCTGGCTGATAATCATCACCTGCAGGTTGGCTCCAATTATTTGT
ATATTCATAAAATCGAGCTTATGCATTTCTTTCCAGACTTGTTCAACAGGCCAGCCATTACGCTCGTCATCAAAATCACTCGCATCAACCAAACCGTTATTCATTCGTGATTGCGCCTGAGCGAGA
CGAAATACGCGATCGCTGTTAAAAGGACAATTACAAACAGGAATCGAATGCAACCGGCGCAGGAACACTGCCAGCGCATCAACAATATTTTCACCTGAATCAGGATATTCTTCTAATACCTGGA
ATGCTGTTTTCCCGGGGATCGCAGTGGTGAGTAACCATGCATCATCAGGAGTACGGATAAAATGCTTGATGGTCGGAAGAGGCATAAATTCCGTCAGCCAGTTTAGTCTGACCATCTCATCTGT
AACATCATTGGCAACGCTACCTTTGCCATGTTTCAGAAACAACTCTGGCGCATCGGGCTTCCCATACAATCGATAGATTGTCGCACCTGATTGCCCGACATTATCGCGAGCCCATTTATACCCATA
TAAATCAGCATCCATGTTGGAATTTAATCGCGGCCTCGAGCAAGACGTTTCCCGTTGAATATGGCTCATAACACCCCTTGTATTACTGTTTATGTAAGCAGACAGTTTTATTGTTCATGATGATAT
ATTTTTATCTTGTGCAATGTAACATCAGAGATTTTGAGACACAACGTGGCTTTGTTGAATAAATCGAACTTTTGCTGAGTTGAAGGATCAGATCACGCATCTTCCCGACAACGCAGACCGTTCCG
TGGCAAAGCAAAAGTTCAAAATCACCAACTGGTCCACCTACAACAAAGCTCTCATCAACCGTGGCTCCCTCACTTTCTGGCTGGATGATGGGGCGATTCAGGCCTGGTATGAGTCAGCAACACC
TTCTTCACGAGGCAGACCTCAGCGCTAACCGTTTTTATCAGGCTCTGGGAGGCAGAATAAATGATCATATCGTCAATTATTACCTCCACGGGGAGAGCCTGAGCAAACTGGCCTCAGGCATTTG
AGAAGCACACGGTCACACTGCTTCCGGTAGTCAATAAACCGGTAAACCAGCAATAGACATAAGCGGCTATTTAACGACCCTGCCCTGAACCGACGACCGGGTCGAATTTGCTTTCGAATTTCTG
CCATTCATCCGCTTATTATCACTTATTCAGGCGTAGCACCAGGCGTTTAAGGGCACCAATAACTGCCTTAAAAAAATTACGCCCCGCCCTGCCACTCATCGCAGTACTGTTGTAATTCATTAAGCA
TTCTGCCGACATGGAAGCCATCACAGACGGCATGATGAACCTGAATCGCCAGCGGCATCAGCACCTTGTCGCCTTGCGTATAATATTTGCCCATGGTGAAAACGGGGGCGAAGAAGTTGTCCA
TATTGGCCACGTTTAAATCAAAACTGGTGAAACTCACCCAGGGATTGGCTGAGACGAAAAACATATTCTCAATAAACCCTTTAGGGAAATAGGCCAGGTTTTCACCGTAACACGCCACATCTTG
CGAATATATGTGTAGAAACTGCCGGAAATCGTCGTGGTATTCACTCCAGAGCGATGAAAACGTTTCAGTTTGCTCATGGAAAACGGTGTAACAAGGGTGAACACTATCCCATATCACCAGCTCA
CCGTCTTTCATTGCCATACG

Supplementary Data 2. Protein sequences of the OMPs used in this study:

FhuA

MARSKTAQPKHSLRKIAVVVATAVSGMSVYAQAAVEPKEDTITVTAAPAPQESAWGPAATIA
ARQSATGTKTDTPIQKVPQSISVVTAEEMALHQPKSVKEALSYTPGVSVGTRGASNTYDHLI
IRGFAAEGQSQNNYLNGLKLQGNFYNDAVIDPYMLERAEIMRGPVSVLYGKSSPGGLLNMVS
KRPTTEPLKEVQFKAGTDSLFQTGFDFSDSLDDDGVYSYRLTGLARSANAQQKGSEEQRYAI
APAFTWRPDDKTNFTFLSYFQNEPETGYYGWLPKEGTVEPLPNGKRLPTDFNEGAKNNTYSR
NEKMVGYSFDHEFNDTFTVRQNLRFAENKTSQNSVYGYGVCSDPANAYSKQCAALAPADKGH
YLARKYVVDDEKLQNFSVDTQLQSKFATGDIDHTLLTGVDFMRMRNDINAWFGYDDSVPLLN
LYNPVNTDFDFNAKDPANSGPYRILNKQKQTGVYVQDQAQWDKVLVTLGGRYDWADQESLNR
VAGTTDKRDDKQFTWRGGVNYLFDNGVTPYFSYSESFEPSSQVGKDGNIFAPSKGKQYEVGV
KYVPEDRPIVVTGAVYNLTKTNNLMADPEGSFFSVEGGEIRARGVEIEAKAALSASVNVVGS
YTYTDAEYTTDTTYKGNTPAQVPKHMASLWADYTFFDGPLSGLTLGTGGRYTGSSYGDPANS
FKVGSYTVVDALVRYDLARVGMAGSNVALHVNNLFDREYVASCFNTYGCFWGAERQVVATAT
FRF

FhuA ΔC

MARSKTAQPKHSLRKIAVVVATAVSGMSVYAQA-----------------------------
----

-------MKEVQFKAGTDSLFQTGFDFSDSLDDDGVYSYRLTGLARSANAQQKGSEEQRYAI
APAFTWRPDDKTNFTFLSYFQNEPETGYYGWLPKEGTVEPLPNGKRLPTDFNEGAKNNTYSR
NEKMVGYSFDHEFNDTFTVRQNLRFAENKTSQNSVYGYGVCSDPANAYSKQCAALAPADKGH
YLARKYVVDDEKLQNFSVDTQLQSKFATGDIDHTLLTGVDFMRMRNDINAWFGYDDSVPLLN
LYNPVNTDFDFNAKDPANSGPYRILNKQKQTGVYVQDQAQWDKVLVTLGGRYDWADQESLNR
VAGTTDKRDDKQFTWRGGVNYLFDNGVTPYFSYSESFFPSSQVGKDGNIFAPSKGKQYEVGV
KYVPEDRPIVVTGAVYNLTKTNNLMADPEGSFFSVEGGEIRARGVEIEAKAALSASVNVVGS
YTYTDAEYTTDTTYKGNTPAQVPKHMASLWADYTFFDGPLSGLTLGTGGRYTGSSYGDPANS
FKVGSYTVVDALVRYDLARVGMAGSNVALHVNNLFDREYVASCFNTYGCFWGAERQVVATAT
FRF

FhuA ΔC/Δ4L NSEGS

MARSKTAQPKHSLRKIAVVVATAVSGMSVYAQA-----------------------------
----

-------LKEVQFKAGTDSLFQTGFDFSDSLDDDGVYSYRLTGLARSANAQQKGSEEQRYAI
APAFTWRPDDKTNFTFLSYFQNEPETG-------------NSEGS-------------TYSR
NEKMVGYSFDHEFNDTFTVRQNLRFAENKTSQNSVYG------------NSEGS--------
---RKYVVDDEKLQNFSVDTQLQSKFATGDIDHTLLTGVDFMRMRNDINAWFGY--------
----NSEGS---------SGPYRILNKQKQTGVYVQDQAQWDKVLVTLGGRYDWADQESLNR
VAGTTDKRDDKQFTWRGGVNYLFDNGVTPYFSYSESFEPSSQVGKDGNIFAPSKGKQYEVGV
KYVPEDRPIVVTGAVYNLTKTNNLMADPEGSFFSVEGGEIRARGVEIEAKAALSASVNVVGS
YTYTDAEYTTDTTYKGNTPAQVPKHMASLWADYTFFDGPLSGLTLGTGGRYTGSSYGDPANS
FKVGSYTVVDALVRYDLARVGMAGSNVALHV----------NSEGS---------QVVATAT
FRF

FhuA ΔC/Δ5L NSEGS

MARSKTAQPKHSLRKIAVVVATAVSGMSVYAQA-----------------------------
----

-------LKEVQFKAGTDSLFQTGFDFSDSLDDDGVYSYRLTGLARSANAQQKGSEEQRYAI
APAFTWRPDDKTNFTFLSYFQNEPETG-------------NSEGS-------------TYSR
NEKMVGYSFDHEFNDTFTVRQNLRFAENKTSQNSVYG------------NSEGS--------
---RKYVVDDEKLQNFSVDTQLQSKFATGDIDHTLLTGVDFMRMRNDINAWFGY--------
----NSEGS---------SGPYRILNKQKQTGVYVQDQAQWDKVLVTLGGRYDWADQESLNR
VAGTTDKRDDKQFTWRGGVNYLFDNGVTPYFSYSESFEPSSQVGKDGNIFAPSKGKQYEVGV
KYVPEDRPIVVTGAVYNLTKTNNLMADPEGSFFSVEGGEIRARGVEIEAKAALSASVNVVGS
YTYTDAEYTTDTTYKGNTPAQVPKHMASLWADYTFFDGPLSGLTLGTGGRYT-----NSEGS
-----YTVVDALVRYDLARVGMAGSNVALHV----------NSEGS---------QVVATAT
FRF

FepA
MNKKIHSLALLVNLGIYGVAQAQEPTDTPVSHDDTIVVTAAEQNLQAPGVSTITADEIRKNP
VARDVSKIIRTMPGVNLTGNSTSGQRGNNRQIDIRGMGPENTLILIDGKPVSSRNSVRQGWR
GERDTRGDTSWVPPEMIERIEVLRGPAAARYGNGAAGGVVNIITKKGSGEWHGSWDAYFNAP
EHKEEGATKRTNFSLTGPLGDEFSFRLYGNLDKTQADAWDINQGHQSARAGTYATTLPAGRE
GVINKDINGVVRWDFAPLQSLELEAGYSRQGNLYAGDTQNTNSDSYTRSKYGDETNRLYRQN
YALTWNGGWDNGVTTSNWVQYEHTRNSRIPEGLAGGTEGKFNEKATQDFVDIDLDDVMLHSE
VNLPIDFLVNQTLTLGTEWNQQRMKDLSSNTQALTGTNTGGAIDGVSTTDRSPYSKAEIFSL
FAENNMELTDSTIVTPGLRFDHHSIVGNNWSPALNISQGLGDDFTLKMGIARAYKAPSLYQT
NPNYILYSKGQGCYASAGGCYLQGNDDLKAETSINKEIGLEFKRDGWLAGVTWFRNDYRNKI
EAGYVAVGQNAVGTDLYQWDNVPKAVVEGLEGSLNVPVSETVMWTNNITYMLKSENKTTGDR
LSIIPEYTLNSTLSWQAREDLSMQTTFTWYGKQQPKKYNYKGQPAVGPETKEISPYSIVGLS
ATWDVTKNVSLTGGVDNLFDKRLWRAGNAQTTGDLAGANYIAGAGAYTYNEPGRTWYMSVNT
HF

FepA ΔC128
MNKKIHSLALLVNLGIYGVAQA--

--------------------------AAARYGNGAAGGVVNIITKKGSGEWHGSWDAYFNAP
EHKEEGATKRTNFSLTGPLGDEFSFRLYGNLDKTQADAWDINQGHQSARAGTYATTLPAGRE
GVINKDINGVVRWDFAPLQSLELEAGYSRQGNLYAGDTQNTNSDSYTRSKYGDETNRLYRQN
YALTWNGGWDNGVTTSNWVQYEHTRNSRIPEGLAGGTEGKFNEKATQDFVDIDLDDVMLHSE
VNLPIDFLVNQTLTLGTEWNQQRMKDLSSNTQALTGTNTGGAIDGVSTTDRSPYSKAEIFSL
FAENNMELTDSTIVTPGLRFDHHSIVGNNWSPALNISQGLGDDFTLKMGIARAYKAPSLYQT
NPNYILYSKGQGCYASAGGCYLQGNDDLKAETSINKEIGLEFKRDGWLAGVTWFRNDYRNKI
EAGYVAVGQNAVGTDLYQWDNVPKAVVEGLEGSLNVPVSETVMWTNNITYMLKSENKTTGDR
LSIIPEYTLNSTLSWQAREDLSMQTTFTWYGKQQPKKYNYKGQPAVGPETKEISPYSIVGLS
ATWDVTKNVSLTGGVDNLFDKRLWRAGNAQTTGDLAGANYIAGAGAYTYNEPGRTWYMSVNT
HF

FepA ΔC128/ΔL
MNKKIHSLALLVNLGIYGVAQA--

--------------------------AAARYGNGAAGGVVNIITKKGSGEWHGSWDAYFNAP
EH---GATKRTNFSLTGPLGDEFSFRLYGNLDKTQ-------------------------RE
GVINKDINGVVRWDFAPLQSLELEAGYSRQGNLYA------------------ETNRLYRQN
YALTWNGGWDNGVTTSNWVQYEHTRNSRIP-----------------DFVDIDLDDVMLHSE
VNLPIDFLVNQTLTLGTEWNQQRMKDL--------------------------YSKAEIFSL
FAENNMELTDSTIVTPGLRFDHHSIVGNNWSPALNISQGLGDDFTLKMGIARAYKAP-----
----------------------------KAETSINKEIGLEFKRDGWLAGVTWFRNDYRNKI

EAGY---------TDLYQWDNVPKAVVEGLEGSLNVPVSETVMWTNNITYMLKSENKT----
----PEYTLNSTLSWQAREDLSMQTTFTWYGK---------------------SPYSIVGLS
ATWDVTKNVSLTGGVDNLFDKR------------------------------GRTWYMSVNT
HF

FepA ΔC
MNKKIHSLALLVNLGIYGVAQA--

--GEWHGSWDAYFNAP
EHKEEGATKRTNFSLTGPLGDEFSFRLYGNLDKTQADAWDINQGHQSARAGTYATTLPAGRE
GVINKDINGVVRWDFAPLQSLELEAGYSRQGNLYAGDTQNTNSDSYTRSKYGDETNRLYRQN
YALTWNGGWDNGVTTSNWVQYEHTRNSRIPEGLAGGTEGKFNEKATQDFVDIDLDDVMLHSE
VNLPIDFLVNQTLTLGTEWNQQRMKDLSSNTQALTGTNTGGAIDGVSTTDRSPYSKAEIFSL
FAENNMELTDSTIVTPGLRFDHHSIVGNNWSPALNISQGLGDDFTLKMGIARAYKAPSLYQT
NPNYILYSKGQGCYASAGGCYLQGNDDLKAETSINKEIGLEFKRDGWLAGVTWFRNDYRNKI
EAGYVAVGQNAVGTDLYQWDNVPKAVVEGLEGSLNVPVSETVMWTNNITYMLKSENKTTGDR
LSIIPEYTLNSTLSWQAREDLSMQTTFTWYGKQQPKKYNYKGQPAVGPETKEISPYSIVGLS
ATWDVTKNVSLTGGVDNLFDKRLWRAGNAQTTGDLAGANYIAGAGAYTYNEPGRTWYMSVNT
HF

FepA ΔC/Δ4L
MNKKIHSLALLVNLGIYGVAQA--

--GEWHGSWDAYFNAP
EHKEEGATKRTNFSLTGPLGDEFSFRLYGNLDKTQ-------------------------RE
GVINKDINGVVRWDFAPLQSLELEAGYSRQGNLYAGDTQNTNSDSYTRSKYGDETNRLYRQN
YALTWNGGWDNGVTTSNWVQYEHTRNSRIPEGLAGGTEGKFNEKATQDFVDIDLDDVMLHSE
VNLPIDFLVNQTLTLGTEWNQQRMKDL--------------------------YSKAEIFSL
FAENNMELTDSTIVTPGLRFDHHSIVGNNWSPALNISQGLGDDFTLKMGIARAYKAP-----
----------------------------KAETSINKEIGLEFKRDGWLAGVTWFRNDYRNKI
EAGYVAVGQNAVGTDLYQWDNVPKAVVEGLEGSLNVPVSETVMWTNNITYMLKSENKTTGDR
LSIIPEYTLNSTLSWQAREDLSMQTTFTWYGKQQPKKYNYKGQPAVGPETKEISPYSIVGLS
ATWDVTKNVSLTGGVDNLFDKR------------------------------GRTWYMSVNT
HF

FepA ΔC/Δ4L NSEGS

```
MNKKIHSLALLVNLGIYGVAQA----------------------------------------
--------------------------------------------------------------
------------------------------------------------GEWHGSWDAYFNAP
EHKEEGATKRTNFSLTGPLGDEFSFRLYGNLDKTQ---------NSEGS-----------RE
GVINKDINGVVRWDFAPLQSLELEAGYSRQGNLYAGDTQNTNSDSYTRSKYGDETNRLYRQN
YALTWNGGWDNGVTTSNWVQYEHTRNSRIPEGLAGGTEGKFNEKATQDFVDIDLDDVMLHSE
VNLPIDFLVNQTLTLGTEWNQQRMKDL---------NSEGS------------YSKAEIFSL
FAENNMELTDSTIVTPGLRFDHHSIVGNNWSPALNISQGLGDDFTLKMGIARAYKAP-----
---------NSEGS--------------KAETSINKEIGLEFKRDGWLAGVTWFRNDYRNKI
EAGYVAVGQNAVGTDLYQWDNVPKAVVEGLEGSLNVPVSETVMWTNNITYMLKSENKTTGDR
LSIIPEYTLNSTLSWQAREDLSMQTTFTWYGKQQPKKYNYKGQPAVGPETKEISPYSIVGLS
ATWDVTKNVSLTGGVDNLFDKR------------NSEGS-------------GRTWYMSVNT
HF
```

FepA ΔC/ΔL NSEGS

```
MNKKIHSLALLVNLGIYGVAQA----------------------------------------
--------------------------------------------------------------
------------------------------------------------GEWHGSWDAYFNAP
EH---GATKRTNFSLTGPLGDEFSFRLYGNLDKTQA--------NSEGS-----------RE
GVINKDINGVVRWDFAPLQSLELEAGYSRQGNLYA------NSEGS-------ETNRLYRQN
YALTWNGGWDNGVTTSNWVQYEHTRNSRIP------NSEGS------DFVDIDLDDVMLHSE
VNLPIDFLVNQTLTLGTEWNQQRMKDL----------NSEGS-----------YSKAEIFSL
FAENNMELTDSTIVTPGLRFDHHSIVGNNWSPALNISQGLGDDFTLKMGIARAYKAP-----
-------NSEGS----------------KAETSINKEIGLEFKRDGWLAGVTWFRNDYRNKI
EAGY--NSEGS--TDLYQWDNVPKAVVEGLEGSLNVPVSETVMWTNNITYMLKSENKT-NSE
GS--PEYTLNSTLSWQAREDLSMQTTFTWYGK-----NSEGS-----------SPYSIVGLS
ATWDVTKNVSLTGGVDNLFDKR-----------NSEGS--------------GRTWYMSVNT
HF
```

OmpF

```
MKKSTLALVVMGIVASASVQAAEIYNKDGNKVDLYGKAVGLHYFSKGNGENSYGGNGDMTYA
RLGFKGETQINSDLTGYGQWEYNFQGNNSEGADAQTGNKTRLAFAGLKYADVGSFDYGRNYG
VVYDALGYTDMLPEFGGDTAYSDDFFVGRVGGVATYRNSNFFGLVDGLNFAVQYLGKNERDT
ARRSNGDGVGGSISYEYEGFGIVGAYGAADRTNLQEAQPLGNGKKAEQWATGLKYDANNIYL
AANYGETRNATPITNKFTNTSGFANKTQDVLLVAQYQFDFGLRPSIAYTKSKAKDVEGIGDV
DLVNYFEVGATYYFNKNMSTYVDYIINQIDSDNKLGVGSDDTVAVGIVYQF
```

OmpF Δ

MKKSTLALVVMGIVASASVQAAEIYNKDGNKVDLYGKAVGLHYFSKGNGENSYGGNGDMTYA
RLGFKGETQINSDLTGYGQWEYNFQGNNSEGADAQTGNKTRLAFAGLKYADVGSFDYGRNYG
VVYDAL------PEFGGDTAYSDDFFVGRVGGVATYRNSNFFGLVDGLNFAVQYLGKNERDT
ARRSNGDGVGGSISYEYEGFGIVGAYGAADRTNLQEAQPLGNGKKAEQWATGLKYDANNIYL
AANYGETRNATPITNKFTNTSGFANKTQDVLLVAQYQFDFGLRPSIAYTKSKAKDVEGIGDV
DLVNYFEVGATYYFNKNMSTYVDYIINQIDSDNKLGVGSDDTVAVGIVYQF

OmpF ΔGI/GL

MKKSTLALVVMGIVASASVQAAEIYNKDGNKVDLYGKAVGLHYFSKGNGENSYGGNGDMTYA
RLGFKGETQINSDLTIYLQWEYNFQGNNSEGADAQTGNKTRLAFAGLKYADVGSFDYGRNYG
VVYDAL------PEFGGDTAYSDDFFVGRVGGVATYRNSNFFGLVDGLNFAVQYLGKNERDT
ARRSNGDGVGGSISYEYEGFGIVGAYGAADRTNLQEAQPLGNGKKAEQWATGLKYDANNIYL
AANYGETRNATPITNKFTNTSGFANKTQDVLLVAQYQFDFGLRPSIAYTKSKAKDVEGIGDV
DLVNYFEVGATYYFNKNMSTYVDYIINQIDSDNKLGVGSDDTVAVGIVYQF
